# Antiproliferative Activity of (−)‐Isopulegol‐based 1,3‐Oxazine, 1,3‐Thiazine and 2,4‐Diaminopyrimidine Derivatives

**DOI:** 10.1002/open.202200169

**Published:** 2022-10-06

**Authors:** Fatima Z. Bamou, Tam M. Le, Bizhar A. Tayeb, Seyyed A. S. Tahaei, Renáta Minorics, István Zupkó, Zsolt Szakonyi

**Affiliations:** ^1^ Institute of Pharmaceutical Chemistry and MTA-SZTE Stereochemistry Research Group Hungarian Academy of Sciences University of Szeged Eötvös u. 6 6720 Szeged Hungary; ^2^ Department of Pharmacodynamics and Biopharmacy University of Szeged Eötvös u. 6 6720 Szeged Hungary

**Keywords:** (−)-isopulegol, (+)-neoisopulegol, 1,3-oxazines, 1,3-thiazines, 2,4-diaminopyrimidines

## Abstract

A series of novel heterocyclic structures, namely 1,3‐oxazines, 1,3‐thiazines and 2,4‐diaminopyrimidines, were designed and synthesised. The bioassay tests demonstrated that, among these analogues, 2,4‐diaminopyridine derivatives showed significant antiproliferative activity against different human cancer cell lines (A2780, SiHa, HeLa, MCF‐7 and MDA‐MB‐231). Pyrimidines substituted with *N* ^2^‐(*p*‐trifluoromethyl)aniline, in particular, displayed a potent inhibitory effect on the growth of cancer cells. Structure–activity relationships were also studied from the aspects of stereochemistry on the aminodiol moiety as well as exploring the effects of substituents on the pyrimidine scaffold.

## Graphical abstract

**A series of novel heterocyclic structures**, namely 1,3‐oxazines, 1,3‐thiazines and 2,4‐diaminopyrimidines, were synthesized by an efficient methodology starting from (−)‐isopulegol. Bioassay tests demonstrated that *N* ^2^‐(*p*‐trifluoromethyl)aniline‐substituted 2,4‐diaminopyridine displayed a significantly more antiproliferative activity against different human cancer cell lines (HeLa, Siha, MDA‐MB‐231, MCF‐7 and A2780) than the positive control, cisplatin. The in vitro values demonstrated that these compounds have the potential to be developed into clinically important therapeutic choices for the treatment of cancer.

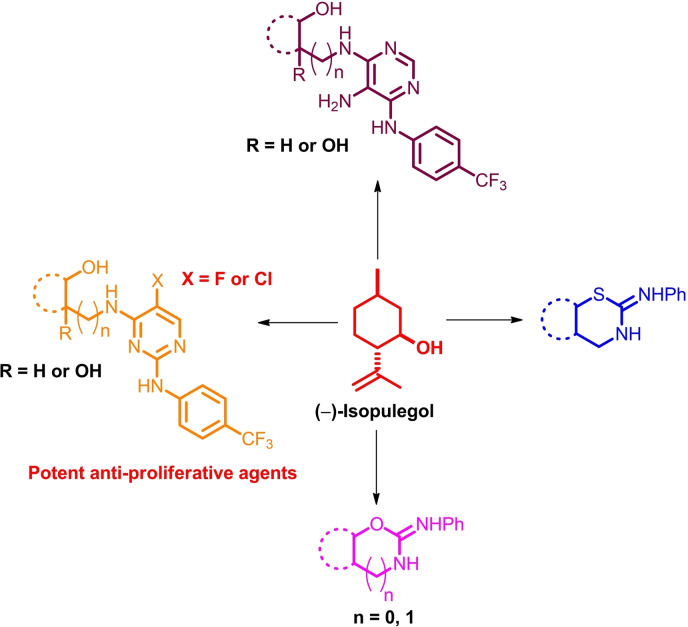

## Introduction

2‐Amino‐1,3‐diol derivatives, important functional motifs, are found in a diverse range of bioactive natural products. Sphingoid bases,^[1]^ sphinganines and clavaminol derivatives,[^2^, ^3^] among others, play unique and crucial roles in many physiological processes. In particular, sphingolipids have been reported to be involved in cell recognition and signal transduction and exhibit prominent antitumor, immune‐modulatory and immunosuppressive activities.^[4]^ In addition, many synthetic compounds have 3‐amino‐1,2‐diol moieties in their backbones, including the antitumor agent aminocyclopentitol pactamycin, the proteasome inhibitor TMC‐95 A, the immunosuppressant antibiotic myriocin, riboflavin (vitamin B2) and the hydrogenase coenzyme F420.^[5]^ Besides being of pharmacological interest, 3‐amino‐1,2‐diols have proven to be excellent building blocks for the synthesis of various heterocyclic compounds such as 1,3‐oxazine,[^6^, ^7^] 1,3‐thiazine^[8]^ and pyrimidines.^[9]^

Pyrimidines belong to an important class of heterocyclic structures found in many synthetic and naturally occurring products with a remarkable spectrum of biological activities.[^10^, ^11^, ^12^, ^13^, ^14^, ^15^] Several valuable reviews illustrate the medicinal and therapeutic properties of pyrimidine derivatives.[^16^, ^17^, ^18^] Among the existing large numbers of structurally diverse pyrimidine derivatives, 2,4‐diaminopyrimidines have attracted considerable attention due to their important chemopreventive and chemotherapeutic effects.[^19^, ^20^, ^21^, ^22^, ^23^, ^24^, ^25^, ^26^, ^27^, ^28^, ^29^, ^30^, ^31^, ^32^, ^33^, ^34^, ^35^, ^36^, ^37^] This structural motif is involved in numerous biological activities – mainly concerning cancer – with action mechanisms related to folate metabolism inhibition,^[38]^ kinase inhibitor activity[^26^, ^39^, ^40^, ^41^, ^42^, ^43^, ^44^] and apoptosis induction.[^22^, ^45^, ^46^] For example, Ceritinib (Zykadia™),^[47]^ Alectinib (Alecensa^TM^),^[48]^ Brigatinib (Alunbrig™),^[49]^ Lorlatinib, (Lorbrena^TM^),^[50]^ small‐molecule antineoplastic anaplastic lymphoma kinase (ALK) inhibitors, and others are used for the treatment of patients with non‐small cell lung cancer. More interestingly, a number of pyrimidine derivatives have recently been identified to have the capacity to inhibit growth of the Aurora kinase‐associated tumors, including VX‐680 (tozasertib)^[51]^ and AZD‐1152 (barasertib).[^52^, ^53^]

Furthermore, 2,4‐diaminopyrimidine‐derived drugs with extensive biological activities, such as anti‐obesity,^[54]^ antiviral,^[55]^ antiparasitics,[^56^, ^57^, ^58^, ^59^] antibacterial,[^60^, ^61^] analgesic,^[62]^ and anti‐inflammatory[^63^, ^64^, ^65^] effects have also received consideration in the field of drug design and development in recent years.

On the other hand, 1,3‐thiazine derivatives, an important class of heterocyclic compounds, have a wide range of biological properties[^66^, ^67^] including antiproliferative,[^68^, ^69^, ^70^, ^71^, ^72^] analgesic,^[73]^ anticonvulsant,[^74^, ^75^] anti‐inflammatory,^[76]^ antibiotic,^[77]^ antimicrobial,[^75^, ^78^, ^79^, ^80^] antimalarial,^[81]^ and antihypertensive^[82]^ properties. A literature survey revealed that also 1,3‐oxazine moieties exhibit a broad range of pharmacological activities,[^83^, ^84^] such as anticancer,[^85^, ^86^, ^87^, ^88^, ^89^] antimicrobial[^90^, ^91^] and anti‐inflammatory[^92^, ^93^] effects, which showed their potential value in developing new therapeutic agents.

Based on the above consideration and in a continuation of our interest in the synthesis of heterocyclic compounds with anticancer activity,[^68^, ^94^, ^95^] starting from isopulegol‐derived aminodiols, a new series of 1,3‐oxazines, oxazoles, 1,3‐thiazines, thiazoles and 2,4‐diaminopyrimidine derivatives was designed, synthesised and trsted for their antiproliferative activities using in vitro assay against different cancer cell lines.

## Results and Discussion

**Scheme 1:**
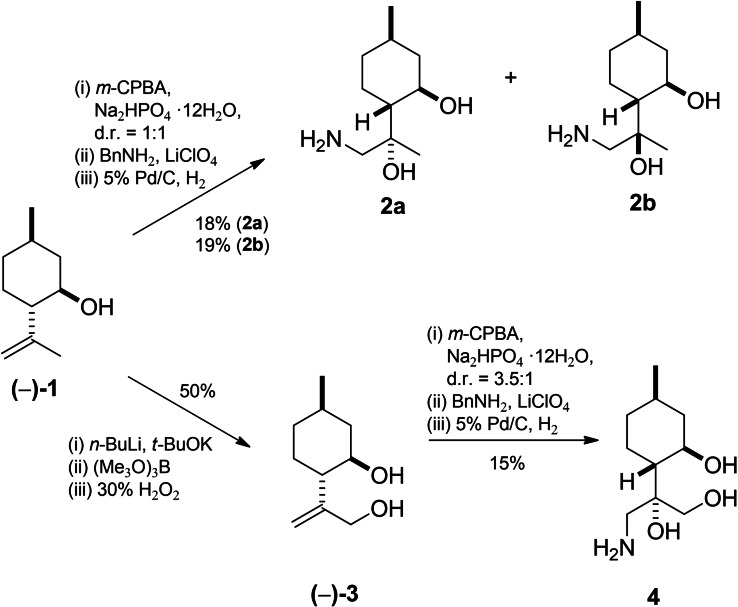
Preparation of (−)‐isopulegol‐based aminodiols **2 a**, **b** and aminotriol **4**.

The synthetic routes started with the preparation of building blocks such as aminodiols, aminotriols and amino alcohols as key intermediates. In the first step, aminodiols **2 a**, **b** were obtained from commercially available (−)‐isopulegol **1** by a three‐step sequence including epoxidation with *m*‐CPBA followed by ring‐opening of the corresponding oxiranes with benzylamine and subsequent hydrogenolysis on 5 % Pd/C.^[96]^ Moreover, regioselective oxidation of **1** gave diol **3**,^[97]^ which was transformed to primary aminotriol **4** according to a literature method (Scheme 1).^[96]^

**Scheme 2:**
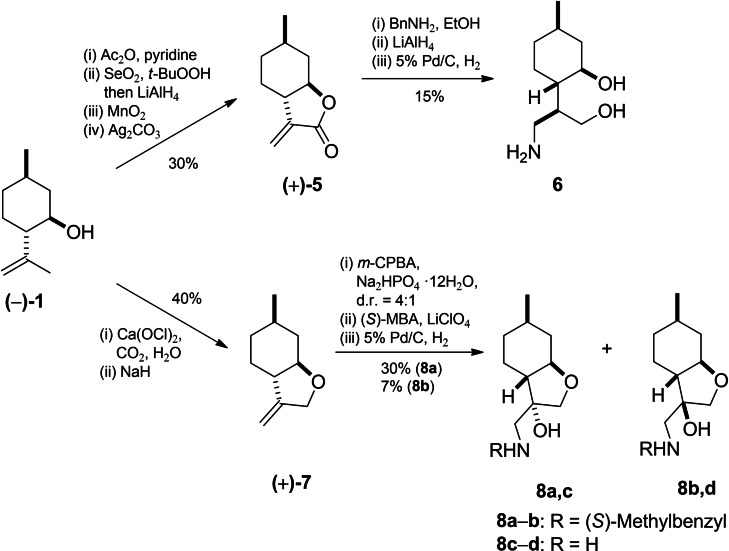
Preparation of (−)‐isopulegol‐based aminodiol **6** and aminoalcohols **8 a**, **b**.

Furthermore, diol **3** was subsequently converted into (+)‐*α*‐methylene‐*γ*‐butyrolactone **5** through a two‐step oxidation and ring closure of the *γ*‐hydroxy‐substituted *α*,*β*‐unsaturated carboxylic acid thus obtained.^[98]^ Reduction of the *β*‐amino lactone, produced by nucleophilic addition of benzylamine to **5**, with LiAlH_4_ followed by debenzylation of the resulting secondary aminodiol over 5 % Pd/C gave primary aminodiol **6**.^[96]^ On the other hand, allylic chlorination of (−)‐**1** and subsequent cyclisation produced *exo*‐methylene tetrahydrofuran **7**.^[99]^ The epoxidation of **7** with *m*‐CPBA delivered a 4 : 1 mixture of epoxides, which was then treated with (*S*)‐methylbenzylamine [(*S*)‐MBA] to provide primary aminoalcohols **8 c**–**d** after debenzylation via hydrogenolysis of corresponding secondary aminoalcohols **8 a**, **b** over 5 % Pd/C (Scheme 2).^[100]^

**Scheme 3:**
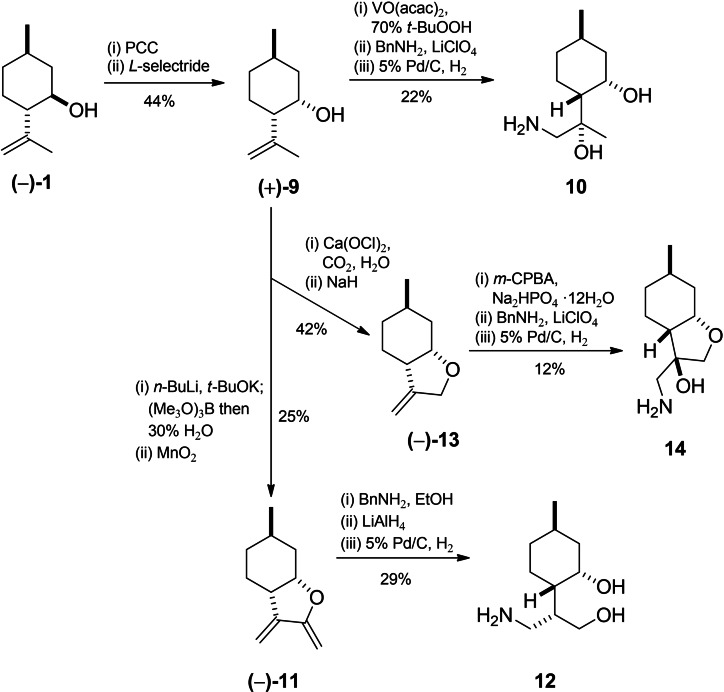
Preparation of (+)‐neoisopulegol‐based aminodiols **10**–**12** and aminoalcohol **14**.

In the same manner, (+)‐neoisopulegol‐based aminodiols^[101]^ and an aminoalcohol^[100]^ were also prepared from (+)‐neoisopulegol **9**, obtained from (−)‐**1** in two steps by Jones oxidation of the hydroxy group followed by stereospecific reduction of (*S*)‐isopulegone over a stoichiometric amount of l‐selectride at −78 °C in THF into the desired *cis* diastereoisomer (Scheme 3).^[98]^

**Scheme 4:**
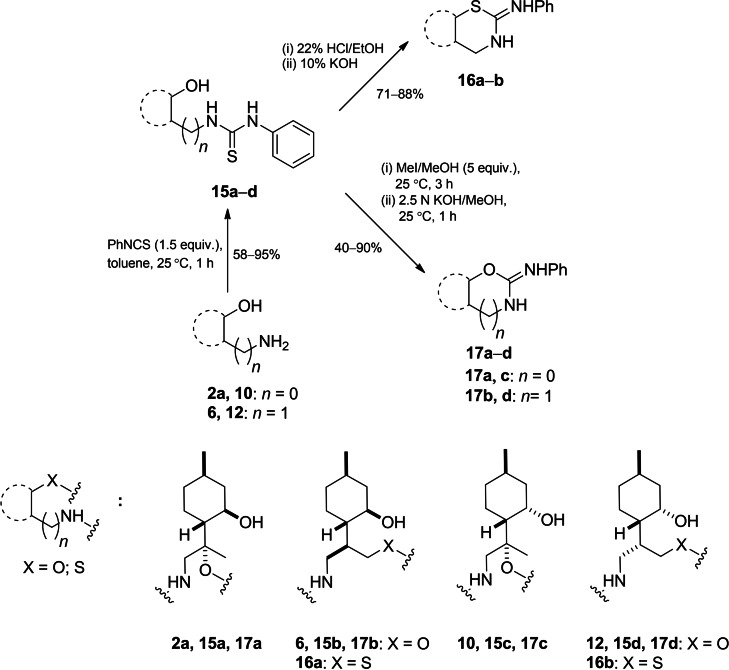
Preparation of (<M‐)‐isopulegol‐based thiazines **16 a**, **b** and oxazines **17 a**–**d**.

With key intermediates in hand, the synthesis of 1,3‐oxazine^[94]^ and 1,3‐thiazine^[68]^ derivatives was carried out by different transformations.^[102]^ Rapid conversion of aminodiols **2 a**, **6**, **10** and **12** with phenyl isothiocyanate in toluene at room temperature provided the corresponding thiourea adducts **15 a**–**d** in moderate yields. The transformation of **2 b**, however, was an exception since no product formation was observed under the applied conditions. This is probably due to steric hindrance exerted by both the methyl substituent at the *α* position and the neighboring hydroxy group of the aminodiol moiety in **2 b**.^[96]^ In the next step, the acid‐catalysed cyclisation of thiourea derivatives **15 a**–**d** could be carried out in one step to yield 1,3‐thiazines. Interestingly, during the ring‐closure process with 22 % HCl in EtOH under the applied conditions, thiourea adducts **15 b**, **d** were preferentially transformed into 2‐phenylimino‐1,3‐thiazines **16 a**, **b**. In contrast, **15 a**, **c** did not react as a result of steric hindrance of the methyl group at the *α* position. We also noticed in our previous work that the cyclisation could be conveniently carried out in the case of thioureas with sterically less hindered structures.^[68]^ On the other hand, treatment of **15 a**–**d** with MeI gave thioethers, which were easily transformed in alkaline medium to 2‐phenylimino‐oxazoles (**17 a,c**) or 2‐phenylimino‐1,3‐oxazines (**17 b**, **d**) (Scheme 4).

**Scheme 5:**
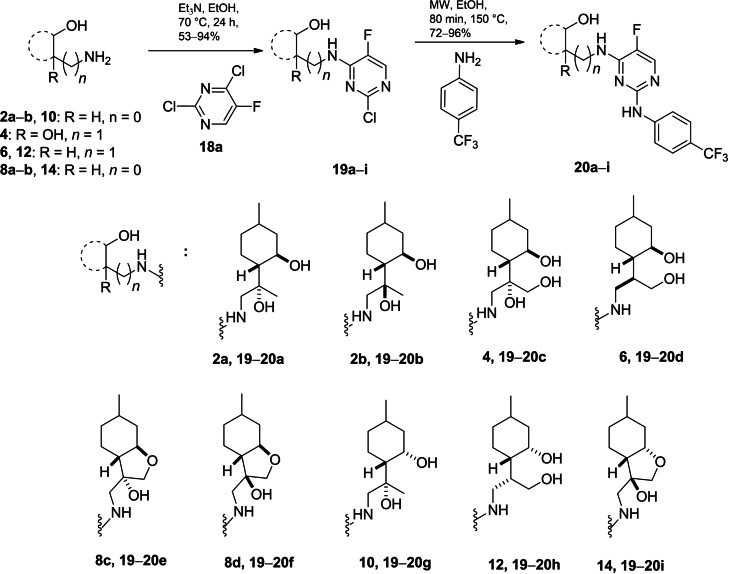
Preparation of (−)‐isopulegol‐based pyrimidine derivatives **19**–**20**.

In addition to the importance of monoterpene‐fused 2‐phenylimino‐1,3‐oxazines and 1,3‐thiazines as antiproliferative agents, a recent report also highlight the anticancer potential of the pyrimidine‐based structures.^[17]^ Consequently, we decided to convert primary aminodiols **2 a**, **b**, **6**, **10**, **12** and aminotriol **4** as well as aminoalcohols **8 a**, **b** and **14** into their pyrimidine scaffolds. Addition of 2,4‐dichloro‐5‐fluoropyrimidine **18 a** to these building blocks in the presence of Et_3_N in EtOH provided 5‐fluoro analogues **19 a**–**i**.^[103]^ These were then applied in microwave‐assisted S_N_Ar coupling reactions with 4‐aminobenzotrifluoride in EtOH at 150 °C to produce **20 a**–**i** as solid precipitates in good yields (Scheme 5).^[104]^

**Scheme 6:**
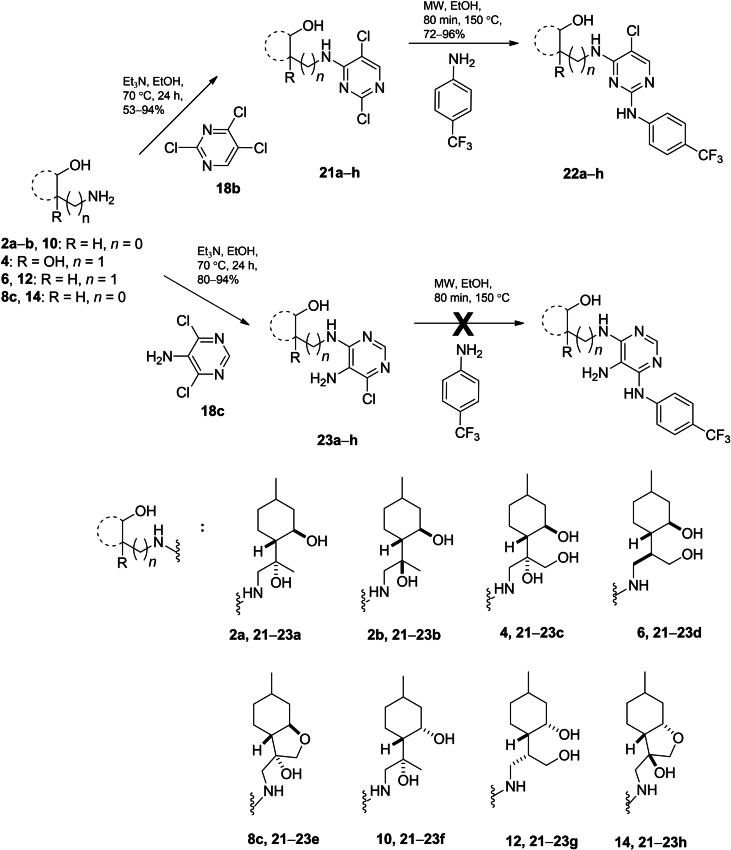
Preparation of (−)‐isopulegol‐based pyrimidine derivatives **21**–**23**.

Due to the anticancer potential of pyrimidines substituted at various positions as well as fused with other heterocyclic rings,^[105]^ coupling reactions were performed by utilising 2,4,5‐trichloropyrimidine **18 b** and 5‐amino‐4,6‐dichloropyrimidine **18 c** as reagents. The desired (−)‐isopulegol‐based pyrimidines **21 a**–**h** and **23 a**–**h** were formed in good yields. In the next step, conversion of **21 a**–**g** with 4‐aminobenzotrifluoride smoothly provided analogues **22 a**–**h** in excellent yields. The next couplings at the remaining chlorine at position 6 in adducts **23 a**–**h**, in turn, were unsuccessful either under standard heating or microwave‐assisted conditions. This is probably due to the steric effect of the amino group at the *ortho* position, making **23 a**–**h** unable to establish the desired interactions (Scheme 6).[^35^, ^106^]

**Figure 1:**
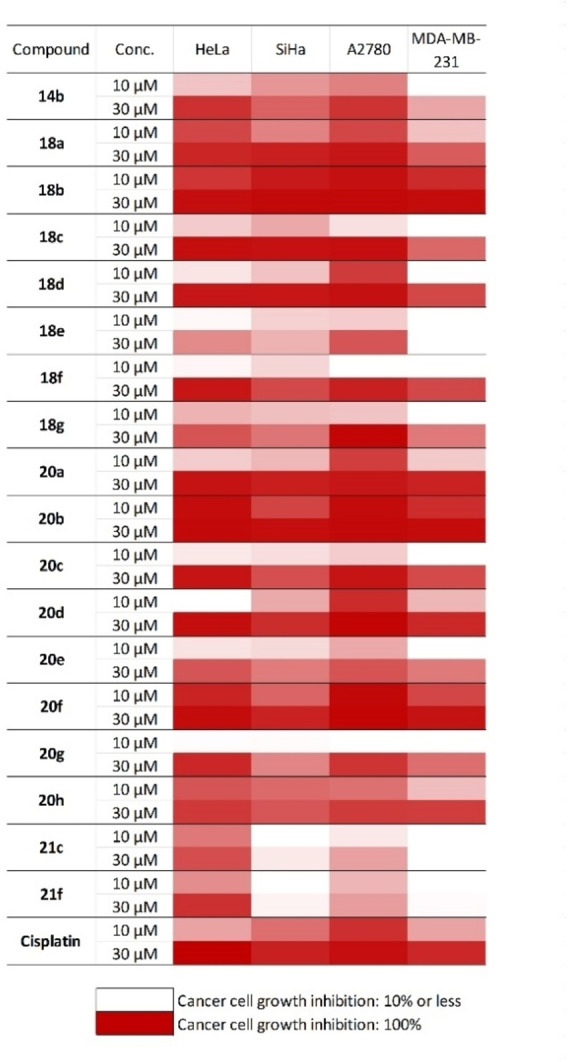
Antiproliferative properties of the selected isopulegol‐based pyrimidine derivatives.

The novel heterocyclic compounds, namely 1,3‐thiazines **16 a**,–**b**, 1,3‐oxazines **17 a**–**d** and diaminopyrimidine analogues **19**–**23** were investigated for their in vitro antiproliferative properties on a panel of different human cancer cell lines (HeLa, SiHa, A2780, and MDA‐MB‐231). The results of MTT assays are presented in Table S1 in the Supporting Information, while selected results (over 70 % inhibition) of the best compounds are given in Figure 1. HeLa and A2780 cells were generally more sensitive to the tested substances than the other two cell lines. (−)‐Isopulegol‐based 2,4‐diaminopyrimidines **19**–**22** were found to show substantial effects of the tested compounds, exhibiting cell growth‐inhibiting capacities comparable to that of the reference agent cisplatin. Among them, 2,4‐diaminopyrimidines **20 a**–**i** and **22 a**–**h** containing the *N* ^2^‐(trifluoromethyl)phenyl group favoured the action, while the absence of this substituent on **19 a**–**i** and **21 a**–**h** led to a generally lesser potency. These results indicate that the introduction of a *N* ^2^‐aryl function into the 2,4‐diaminopyrimidine skeleton has a significant impact on their efficacy. Since no substantial difference was observed between the effects of **20 b** and **22 b**, the type of halogen substituent at the 5 position on the pyrimidine scaffold also seems irrelevant. On the other hand, pyrimidine analogues **22 a**, **b**, **22 d**, **22 f**, **22 g** were highly effective, indicating that the aminodiol system is an essential part of the molecule. Among the series of pyrimidine derivatives derived from aminodiols, antiproliferative activities were influenced by the aminodiol structure. Overall, aminodiols with the presence of a methyl group (**22 a**, **b**, **22 f**) resulted in higher effects against all tested cell lines than the corresponding compounds without a methyl group in the aminodiol moiety (**22 d**, **22 g**). This illustrates that the methyl group may be helpful to increase the cytotoxicity of this compound class. Replacement of the aminodiol system of **22 a** by an aminotriol moiety (**22 c**) was detrimental for antiproliferative inhibitory activity owing to the introduction of water‐solubilising groups to reduce cell permeability,^[104]^ while replacing both aminodiol moieties in **22 a** with an aminoalcohol, such as **22 e**, produced a similar effect, demonstrating the crucial role of a building block for the design and synthesis of novel antiproliferative agents. Furthermore, the comparison of the antiproliferative activities of **22 a** and **22 f** as well as those of **22 a** and **22 b** confirmed that both the stereochemistry of the aminodiol and the presence of the hydroxy moiety on the cyclohexane ring have influence on antiproliferative activity. Considering the effect of the stereochemistry of the OH group on the alkyl chain to the antiproliferative activity, aminodiol **22 b** with *R* configuration was found to be more effective compared to its corresponding isomer (**22 a**), whereas the stereochemistry of the hydroxy substituent on the cyclohexane ring in the aminodiol function did not contribute to activity improvement.

The effects of the most promising analogues (**20 b** and **22 b**) are comparable to that of the reference agent cisplatin, as reflected in their calculated IC_50_ values (Figure 1 and Table S1 in the Supporting Information).

Although the interactions between 2,4‐diaminopyrimidine scaffolds containing the *N* ^2^‐(*p*‐trifluoromethyl)phenyl group and Aurora A have already been studied on the molecular level using X‐ray crystallography,^[104]^ molecular docking simulations could also enable us to comprehend the binding pattern between ligand and protein intuitively. These key interactions contribute to the high in vitro potency of compound **20 b**. A docking study using Discovery Studio 2.5 was thus conducted investigate the possible interactions between compound **20 b** and Aurora A kinase PDB (Code: 4DEE).

**Figure 2:**
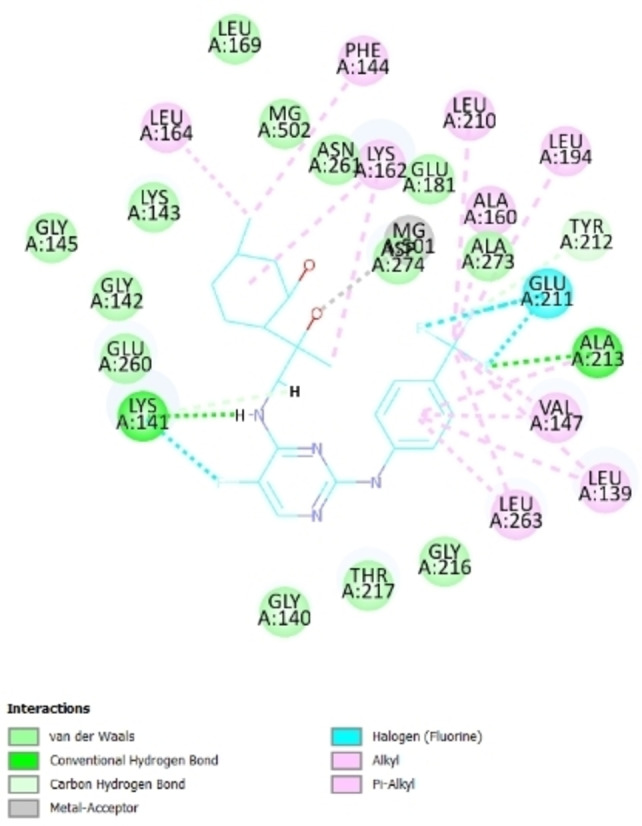
2D diagram of key binding interactions of hit (compound **20 b**) with Aurora A.

The 2D diagram of key binding interactions of hit (compound 20b) with Aurora A (Figure 2) shows that the methylcyclohexyl moiety forms hydrophobic alkyl interaction with a hydrophobic pocket formed by Phe144, Leu164 and Lys162, while the aromatic ring has a variety of pi‐alkyl interaction with another hydrophobic pocket Leu139, Val147 and Leu263. The amine group of aminodiol also creates a significant hydrogen bond with Lys141. These key interactions contribute to the high in vitro potency of compound 20b.

The molecular properties of **20 b** were determined and the results show that compound **20 b** meets Lipinski's rules of five. Furthermore, an in‐silico ADMET study confirmed that this compound has good absorption through human intestinal. Additionally, it is proposed to exhibit low ability to penetrate the blood–brain barrier (BBB), as shown in the Supporting Information.

## Conclusion

A library of heterocylic compounds such as 1,3‐oxazines, oxazoles, 1,3‐thiazines, thiazoles and 2,4‐diaminopyrimidines, was prepared from commercially available (−)‐isopulegol.

The in vitro pharmacological studies showed that 2,4‐diaminopyrimidines exerted antiproliferative action on different human cancer cell lines. Among them, aminodiols based on *N* ^*2*^‐(*p*‐trifluorophenyl)amino and *N* ^4^‐(−)‐isopulegol, simultaneously incorporating diaminopyrimidines, proved to be more potent than the clinically used anticancer agent cisplatin.

Furthermore, the in vitro experiments also clearly demonstrated that the stereochemistry of the hydroxy substituent on the cyclohexane ring in the aminodiol moiety has no influence on the antiproliferative effect, whereas the inhibitory activity was found to be affected by the stereochemistry of the alkyl chain. *R*‐isomers were more potent than the corresponding *S*‐isomers against different cancer cell lines

Preliminary exploration indicated that compounds **20 b** and **22 b** have great promise against different cancer cell lines. To our delight, molecular docking results exemplified that **20 b** could foster potent affinity by forming significant hydrogen and hydrophobic interactions with Aurora A kinase (PDB Code: 4DEE), a target for anticancer drugs in preclinical models.^[104]^ In summary, compounds **20 b** and **22 b** can be regarded as new potential inhibitors. Further studies will be performed and reported in the future. Therefore, in the next stage of our project, we plan to obtain *N* ^*2*^‐substituted aryl analogues, preferably with different substitutions on *N*‐phenyl systems, to improve their antiproliferative activities on a panel of different cancer cell lines. Additionally, docking studies and molecular dynamics studies with the optimised derivatives will also be performed to get an insight into the dynamics of ligand interaction.

## Experimental Section

**General methods**: ^1^H and ^13^C NMR spectra were recorded on a Bruker Avance DRX 500 spectrometer (500 and 125 MHz, respectively, *δ*=0 ppm (TMS)). Chemical shifts (*δ*) are expressed in [ppm] relative to TMS as internal reference. *J* values are given in [Hz]. HRMS flow injection analysis was performed with a Thermo Scientific Q Exactive Plus hybrid quadrupole‐Orbitrap (Thermo Fisher Scientific, Waltham, MA, USA) mass spectrometer coupled to a Waters Acquity I–Class UPLC™ (Waters, Manchester, UK). Optical rotations were determined with a Perkin–Elmer 341 polarimeter. Melting points were determined on a Kofler apparatus and they are uncorrected. Chromatographic separations were carried out on Merck Kieselgel 60 (230–400 mesh ASTM). Reactions were monitored with Merck Kieselgel 60 F_254_‐precoated TLC plates (0.25 mm thickness). Commercially available reagents were used as obtained from suppliers (Molar Chemicals Ltd., Halásztelek, Hungary; Merck Ltd., Budapest, Hungary and VWR International Ltd., Debrecen, Hungary), while solvents were dried according to standard procedures.

**Starting materials**: (−)‐Isopulegol (**1**) is commercially available from Merck Co with *ee*=95 %, ([*α*]20 D=−22.0, neat) and its enatimomer (+)‐**1** (*ee*=90 %, [*α*]20 D=+22.0, neat). (+)‐Neoisopulegol (**2**) ([*α*]20 D=+28.7, c=17.2, CHCl_3_) and its enatimomer (−)‐**2** ([*α*]20 D=−22.2, c=2.0, CHCl_3_) were synthesized from (−)‐**1** and its isomer (+)‐**1** following a reported procedure, respectively.^[107]^ (−)‐Isopulegol‐ based aminodiols **2 a**, **b** and **6**,^[96]^ (+)‐neoisopulegol‐based aminodiols **10** and **12** ^[101]^ together with aminotriol **4** ^[96]^ as well as aminoalcohols **14** ^[100]^ were prepared according to literature procedures. All spectroscopic data were similar to those described therein.

**Docking Study**: Aurora A kinase crystal structure was obtained from PDB (protein data bank). ChemBioDraw Ultra 11.0 was used to design the compound for the docking study. The docking study and in silico ADMET predictions were performed by Accelrys discovery studio 2.5 software.

**Choosing the template crystal structure**: Aiming to choose the most valid crystal structure to be used in our study, we first downloaded the available Aurora A crystal structure from PDB.

**Preparation of the crystal structure of Aurora A**: It is well known that the extracted crystal structure from PDB does not have hydrogen atoms, so firstly, hydrogen atoms must be added by applying several force fields (CHARMm). Adding hydrogen atoms leads to steric hindrance and subsequently to high energy and unstable molecule, which should be minimised. Minimisation of the crystal structure was performed by using adopted basis minimisation aiming at finding the most stable and least energy structure and reducing H−H interactions without affecting the basic protein skeleton atoms. Then, the active site was determined and the sphere surrounded.^[108]^

**Docking study (CDocker)**: By using CDocker method, we can generate all the possible conformations of the compound in the protein active site. Then the results can be assessed by both the CDocker energy and the number of interactions between the ligand and active site. This method requires preparing the crystal structure (as mentioned before) and preparing the designed compound by using Accelrys Discovery Studio protocol and applying force field.^[109]^

Before starting this study, it is important to make sure that the used method is valid by comparing the conformation of the reference compound with its conformations generated by the docking method, where RMSD (Root Mean Square Deviation) should not exceed 2 Å.

*(3S,3aR,6R,7aR)‐6‐Methyl‐3‐((((S)‐1‐phenylethyl)amino)methyl)octahydrobenzofuran‐3‐ol* (**8 a**) *and (3R,3aR,6R,7aR)‐6‐Methyl‐3‐((((S)‐1‐phenylethyl)amino)methyl)octahydrobenzofuran‐3‐ol* (**8 b**)

*m*‐CPBA (70 % purity, 5.87 g, 23.8 mmol) was added at 0 °C to a solution of **7** (11.9 mmol) in CH_2_Cl_2_ (50 mL) and Na_2_HPO_4_ ⋅ 12H_2_O (6.35 g, 35.7 mmol) in water (130 mL), and the mixture was stirred at room temperature. When the reaction was complete, as indicated by TLC (2 h), the mixture was separated and the aqueous phase was extracted with CH_2_Cl_2_ (100 mL). The organic layer was washed with a 5 % KOH solution (3×50 mL), then dried (Na_2_SO_4_) and evaporated to provide a 4 : 1 mixture of epoxides as a pale‐yellow oil, which was added to the solution of (*S*)‐methylbenzylamine (0.80 mL, 6.20 mmol) in MeCN (30 mL) and LiClO_4_ (0.31 g, 2.94 mmol). The mixture was kept at reflux temperature for 6 h. When the reaction was completed (indicated by TLC), the mixture was evaporated to dryness, and the residue was dissolved in water (15 mL) and then extracted with CH_2_Cl_2_ (3×50 mL). The combined organic phase was dried (Na_2_SO_4_), filtered, and concentrated. The crude product was purified by column chromatography on silica gel with an appropriate solvent mixture (CHCl_3_:MeOH=19 : 1) to provide **8 a** and **8 b**.

**8 a**: Yellow crystals (33 %); m. p. 54–55 °C; [*α*]20 D=−51.0 (c 0.1275, MeOH). All spectroscopic data (^1^H and ^13^C NMR together with HRMS) can be found in the Supporting Information.

**8 b**: White crystals (8 %); m. p. 68–69 °C; [*α*]20 D=−46.0 (c 0.21, MeOH). All spectroscopic data (^1^H and ^13^C NMR together with HRMS) can be found in the Supporting Information.

*(3S,3aR,6R,7aR)‐3‐(Aminomethyl)‐6‐methyloctahydrobenzofuran‐3‐ol* (**8 c**) *and (3R,3aR,6R,7aR)‐3‐(Aminomethyl)‐6‐methyloctahydrobenzofuran‐3‐ol* (**8 d**)

Aminoalcohols **8 a**–**b** (14.0 mmol) in MeOH (100 mL) were added to a suspension of palladium‐on‐carbon (5 % Pd, 0.22 g) in MeOH (50 mL), and the mixture was stirred under an H_2_ atmosphere (1 atm) at room temperature. After completion of the reaction (as monitored by TLC, 24 h), the mixture was filtered through a Celite pad, and the solution was evaporated to dryness. The crude product was recrystallised in Et_2_O, resulting in primary aminoalcohols **8 c**–**d** as white crystals.

**8 c**: White crystals (90 %); m. p. 190–192 °C; [*α*]20 D=−3.0 (c 0.26, MeOH). All spectroscopic data (^1^H and ^13^C NMR together with HRMS) can be found in the Supporting Information.

**8 d**: White crystals (87 %); m. p. 193–196 °C; [*α*]20 D=−12.0 (c 0.14, MeOH). All spectroscopic data (^1^H and ^13^C NMR together with HRMS) can be found in the Supporting Information.

**General procedure for the preparation of thioureas (15 a**–**d)**: Aminodiols **2 a, 6, 10** and **12** (0.53 mmol) and the appropriate phenylisothiocyanate (0.79 mmol) were dissolved in toluene (40 mL), and the mixture was stirred at room temperature for 1 h, except that in the case of **6** when a treatment at reflux temperature for 3 h was carried out. The resulting mixtures were then evaporated then the residue was purified by column chromatography on silica gel (eluted with CHCl_3_:MeOH=19 : 1).

*1‐((S)‐2‐Hydroxy‐2‐((1R,2R,4R)‐2‐hydroxy‐4‐methylcyclohexyl)propyl)‐3‐phenylthiourea* (**15 a**): Prepared from **2 a**. White crystals (95 %); m. p. 162–163 °C; [*α*]20 D=−33.0 (c 0.28, MeOH). All spectroscopic data (^1^H and ^13^C NMR together with HRMS) can be found in the Supporting Information.

*1‐((R)‐3‐Hydroxy‐2‐((1S,2R,4R)‐2‐hydroxy‐4‐methylcyclohexyl)propyl)‐3‐phenylthiourea* (**15 b**): Prepared from **6**. White crystals (58 %); m. p. 97–98 °C; [*α*]20 D=−22.0 (c 0.28, MeOH). All spectroscopic data (^1^H and ^13^C NMR together with HRMS) can be found in the Supporting Information.

*1‐((S)‐2‐Hydroxy‐2‐((1R,2S,4R)‐2‐hydroxy‐4‐methylcyclohexyl)propyl)‐3‐phenylthiourea* (**15 c**): Prepared from **10**. Yellow oil (61 %); [*α*]20 D=−5.0 (c 0.295, MeOH). All spectroscopic data (^1^H and ^13^C NMR together with HRMS) can be found in the Supporting Information.

*1‐((S)‐3‐Hydroxy‐2‐((1S,2S,4R)‐2‐hydroxy‐4‐methylcyclohexyl)propyl)‐3‐phenylthiourea* (**15 d**): Prepared from **12**. White crystals (94 %); m. p. 50–52 °C; [*α*]20 D=+25.0 (c 0.265, MeOH). All spectroscopic data (^1^H‐ and ^13^C NMR together with HRMS) can be found in the Supporting Information.

**General procedure for the preparation of 1,3**–**thiazines** (**16 a, b**): A solution of thioureas **15 b** or **15 d** (0.31 mmol) in dry EtOH (1 mL) was added 22 % HCl in EtOH (5 mL) and the mixture was stirred at room temperature for 4 h and then concentrated under vacuum. The residue was treated with 10 % KOH in MeOH (20 mL) followed by evaporation, and the crude product was again dissolved in water (10 mL) and extracted with CHCl_3_ (3×20 mL). The combined organic layer was washed with saturated NaCl aqueous solution (15 mL), dried (Na_2_SO_4_) and concentrated under vacuum. The crude product was purified by column chromatography on silica gel with CHCl_3_:MeOH=19 : 1.

*(1R,2S,5R)‐5‐Methyl‐2‐((R)‐2‐(phenylimino)‐1,3‐thiazinan‐5‐yl)cyclohexanol* (**16 a**): Prepared from **15 b**. White crystals (71 %); m. p. 73–75 °C; [*α*]20 D=−46.0 (c 0.9, MeOH). All spectroscopic data (^1^H and ^13^C NMR together with HRMS) can be found in the Supporting Information.

*(1S,2S,5R)‐5‐Methyl‐2‐((S)‐2‐(phenylimino)‐1,3‐thiazinan‐5‐yl)cyclohexanol* (**16 b**): Prepared from **15 d**. White crystals (88 %); m. p. 224–225 °C; [*α*]20 D=+9.0 (c 0.29, MeOH). All spectroscopic data (^1^H and ^13^C NMR together with HRMS) can be found in the Supporting Information.

**General procedure for the synthesis of 1.3‐oxazines** (**17 a**–**d**): To a solution of **15 a**–**d** (0.31 mmol) in MeOH (4 mL), MeI (1.50 mmol) was added. After 3 h stirring at room temperature, the mixture was evaporated, followed by adding 2.5 m KOH in MeOH (20 mL) and subsequently stirred for 1 h before evaporation. The residue was dissolved in water (20 mL) and extracted with CHCl_3_ (3×20 mL). The organic phase was then dried with Na_2_SO_4_ and evaporated to dryness. The crude product was purified by column chromatography on silica gel (CHCl_3_:MeOH=19 : 1).

*(1R,2R,5R)‐5‐Methyl‐2‐((S)‐5‐methyl‐2‐(phenylimino)oxazolidin‐5‐yl)cyclohexanol* (**17 a**): Prepared from **15 a**. White crystals (76 %); m. p. 94–96 °C; [*α*]20 D=−5.0 (c 0.28, MeOH). All spectroscopic data (^1^H and ^13^C NMR together with HRMS) can be found in the Supporting Information.

*(1R,2S,5R)‐5‐Methyl‐2‐((R)‐2‐(phenylimino)‐1,3‐oxazinan‐5‐yl)cyclohexanol* (**17 b**): Prepared from **15 b**. White crystals (50 %); m. p. 166–168 °C; [*α*]20 D=−37.0 (c 0.25, MeOH). All spectroscopic data (^1^H and ^13^C NMR together with HRMS) can be found in the Supporting Information.

*(1S,2R,5R)‐5‐Methyl‐2‐((S)‐5‐methyl‐2‐(phenylimino)oxazolidin‐5‐yl)cyclohexanol (* **17 c**): Prepared from **15 c**. White crystals (90 %); m. p. 94–95 °C; [*α*]20 D=−16.0 (c 0.25, MeOH). All spectroscopic data (^1^H and ^13^C NMR together with HRMS) can be found in the Supporting Information.

*(1S,2S,5R)‐5‐Methyl‐2‐((S)‐2‐(phenylimino)‐1,3‐oxazinan‐5‐yl)cyclohexanol* (**17 d**): Prepared from **15 d**. White crystals (83 %); m. p. 171–172 °C; [*α*]20 D=+49.0 (c 0.27, MeOH). All spectroscopic data (^1^H and ^13^C NMR together with HRMS) can be found in the Supporting Information.

**General procedure for the preparation of pyrimidine analogues** (**19 a**–**h**), (**21 a**–**g**) and (**23 a**–**g**): To a solution of aminodiols **2 a**, **b**, **6**, **10**, and **12** together with aminotriol **4** as well as aminoalcohols **8 a**, **b**, **14** (0.6 mmol) in EtOH (2 mL), 2,4‐dichloro‐5‐fluoropyrimidine **18 a**, 2,4,5‐trichloropyrimidine **18 b** or 4,6‐dichloropyrimidine‐5‐amine **18 c** (0.6 mmol) and Et_3_N (1.8 mmol, 182 mg) were added. After a treatment at reflux temperature for 24 h, the reaction mixture was cooled to room temperature and concentrated under reduced pressure. The crude product was dissolved in EtOAc (15 mL) and washed with H_2_O (3×15 mL). The organic layer was dried (Na_2_SO_4_) and concentrated under reduced pressure. The residue was purified by column chromatography on silica gel and eluted with CHCl_3_:MeOH=19 : 1.

*(1R,2R,5R)‐2‐((S)‐1‐((2‐Chloro‐5‐fluoropyrimidin‐4‐yl)amino)‐2‐hydroxypropan‐2‐yl)‐5‐methylcyclohexanol* (**19 a**): Prepared from **2 a** and **18 a**. Brown crystals (94 %); m. p. 66–69 °C; [*α*]20 D=+63.0 (c 0.13, MeOH). All spectroscopic data (^1^H and ^13^C NMR together with HRMS) can be found in the Supporting Information.

*(1R,2R,5R)‐2‐((R)‐1‐((2‐Chloro‐5‐fluoropyrimidin‐4‐yl)amino)‐2‐hydroxypropan‐2‐yl)‐5‐methylcyclohexanol* (**19 b**): Prepared from **2 b** and **18 a**. White crystals (88 %); m. p. 100–104 °C; [*α*]20 D=+4.0 (c 0.13, MeOH). All spectroscopic data (^1^H and ^13^C NMR together with HRMS) can be found in the Supporting Information.

(*S*)‐3‐((2‐Chloro‐5‐fluoropyrimidin‐4‐yl)amino)‐2‐((1*R*,2*R*,4*R*)‐2‐hydroxy‐4‐methylcyclohexyl)propane‐1,2‐diol (**19 c**): Prepared from **4** and **18 a**. Yellow oil (94 %); [*α*]20 D =+8.0 (c 0.1375, MeOH). All spectroscopic data (^1^H and ^13^C NMR together with HRMS) can be found in the Supporting Information.

*(1R,2S,5R)‐2‐((R)‐1‐((2‐Chloro‐5‐fluoropyrimidin‐4‐yl)amino)‐3‐hydroxypropan‐2‐yl)‐5‐methylcyclohexanol (* **19 d**): Prepared from **6** and **18 a**. White crystals (84 %); m. p. 172–174 °C; [*α*]20 D=−55.0 (c 0.13, MeOH). All spectroscopic data (^1^H and ^13^C NMR together with HRMS) can be found in the Supporting Information.

*(3S,3aR,6R,7aR)‐3‐(((2‐Chloro‐5‐fluoropyrimidin‐4‐yl)amino)methyl)‐6‐methyloctahydrobenzofuran‐3‐ol (* **19 e**): Prepared from **8 a** and **18 a**. White crystals (53 %); m. p. 175–177 °C; [*α*]20 D=−3.0 (c 0.1150, MeOH). All spectroscopic data (^1^H and ^13^C NMR together with HRMS) can be found in the Supporting Information.

*(3R,3aR,6R,7aR)‐3‐(((2‐Chloro‐5‐fluoropyrimidin‐4‐yl)amino)methyl)‐6‐methyloctahydrobenzofuran‐3‐ol* (**19 f**): Prepared from **8 b** and **18 a**. Colorless oil (70 %); [*α*]20 D=−3.0 (c 0.1425, MeOH). All spectroscopic data (^1^H and ^13^C NMR together with HRMS) can be found in the Supporting Information.

*(1S,2R,5R)‐2‐((S)‐1‐((2‐Chloro‐5‐fluoropyrimidin‐4‐yl)amino)‐2‐hydroxypropan‐2‐yl)‐5‐methylcyclohexanol* (**19 g**): Prepared from **10** and **18 a**. White crystals (75 %); m. p. 158–160 °C; [*α*]20 D=+36.0 (c 0.1450, MeOH). All spectroscopic data (^1^H and ^13^C NMR together with HRMS) can be found in the Supporting Information.

*(1S,2S,5R)‐2‐((S)‐1‐((2‐Chloro‐5‐fluoropyrimidin‐4‐yl)amino)‐3‐hydroxypropan‐2‐yl)‐5‐methylcyclohexanol* (**19 h**): Prepared from **12** and **18 a**. White crystals (88 %); m. p. 142–144 °C; [*α*]20 D=+26.0 (c 0.1250, MeOH). All spectroscopic data (^1^H and ^13^C NMR together with HRMS) can be found in the Supporting Information.

*(3R,3aR,6R,7aS)‐3‐(((2‐Chloro‐5‐fluoropyrimidin‐4‐yl)amino)methyl)‐6‐methyloctahydrobenzofuran‐3‐ol* (**19 i**): Prepared from **14** and **18 a**. White crystals (72 %); m. p. 156–158 °C; [*α*]20 D=−7.0 (c 0.1375, MeOH). All spectroscopic data (^1^H and ^13^C NMR together with HRMS) can be found in the Supporting Information.

*(1R,2R,5R)‐2‐((S)‐1‐((2,5‐Dichloropyrimidin‐4‐yl)amino)‐2‐hydroxypropan‐2‐yl)‐5‐methylcyclohexanol* (**21 a**): Prepared from **2 a** and **18 b**. White crystals (73 %); m. p. 110–113 °C; [*α*]20 D=+66.0 (c 0.15, MeOH). All spectroscopic data (^1^H and ^13^C NMR together with HRMS) can be found in the Supporting Information.

*(1R,2R,5R)‐2‐((R)‐1‐((2,5‐Dichloropyrimidin‐4‐yl)amino)‐2‐hydroxypropan‐2‐yl)‐5‐methylcyclohexanol* (**21 b**): Prepared from **2 b** and **18 b**. White crystals (75 %); m. p. 94–96 °C; [*α*]20 D=+3.0 (c 0.1350, MeOH). All spectroscopic data (^1^H and ^13^C NMR together with HRMS) can be found in the Supporting Information.

*(S)‐3‐((2,5‐Dichloropyrimidin‐4‐yl)amino)‐2‐((1R,2R,4R)‐2‐hydroxy‐4‐methylcyclohexyl)propane‐1,2‐diol* (**21 c**): Prepared from **4** and **18 b**. White crystals (70 %); m. p. 118–120 °C; [*α*]20 D=+1.0 (c 0.1150, MeOH). All spectroscopic data (^1^H and ^13^C NMR together with HRMS) can be found in the Supporting Information.

*(1R,2S,5R)‐2‐((R)‐1‐((2,5‐Dichloropyrimidin‐4‐yl)amino)‐3‐hydroxypropan‐2‐yl)‐5‐methylcyclohexanol (* **21 d**): Prepared from **6** and **18 b**. White crystals (84 %); m. p. 138–140 °C; [*α*]20 D=−45.0 (c 0.1150, MeOH). All spectroscopic data (^1^H and ^13^C NMR together with HRMS) can be found in the Supporting Information.

*(3S,3aR,6R,7aR)‐3‐(((2,5‐Dichloropyrimidin‐4‐yl)amino)methyl)‐6‐methyloctahydrobenzofuran‐3‐ol* (**21 e**): Prepared from **8 a** and **18 b**. Yellow crystals (88 %); m. p. 90–91 °C; [*α*]20 D=−6.0 (c 0.13, MeOH). All spectroscopic data (^1^H and ^13^C NMR together with HRMS) can be found in the Supporting Information.

*(1S,2R,5R)‐2‐((S)‐1‐((2,5‐Dichloropyrimidin‐4‐yl)amino)‐2‐hydroxypropan‐2‐yl)‐5‐methylcyclohexanol* (**21 f**): Prepared from **10** and **18 b**. Yellow oil (84 %); [*α*]20 D=+17.0 (c 0.1375, MeOH). All spectroscopic data (^1^H and ^13^C NMR together with HRMS) can be found in the Supporting Information.

*(1S,2S,5R)‐2‐((S)‐1‐((2,5‐Dichloropyrimidin‐4‐yl)amino)‐3‐hydroxypropan‐2‐yl)‐5‐methylcyclohexanol* (**21 g**): Prepared from **12** and **18 b**. White crystals (85 %); m. p. 184–186 °C; [*α*]20 D=+6.0 (c 0.1425, MeOH). All spectroscopic data (^1^H and ^13^C NMR together with HRMS) can be found in the Supporting Information.

*(3R,3aR,6R,7aS)‐3‐(((2,5‐Dichloropyrimidin‐4‐yl)amino)methyl)‐6‐methyloctahydrobenzofuran‐3‐ol* (**21 h**): Prepared from **14** and **18 b**. White crystals (80 %); m. p. 185–186 °C; [*α*]20 D=+3.0 (c 0.13, MeOH). All spectroscopic data (^1^H and ^13^C NMR together with HRMS) can be found in the Supporting Information.

*(1R,2R,5R)‐2‐((S)‐1‐((5‐Amino‐6‐chloropyrimidin‐4‐yl)amino)‐2‐hydroxypropan‐2‐yl)‐5‐methylcyclohexanol* (**23 a**): Prepared from **2 a** and **18 c**. Yellow oil (82 %); [*α*]20 D=−10.0 (c 0.13, MeOH). All spectroscopic data (^1^H and ^13^C NMR together with HRMS) can be found in the Supporting Information.

*(1R,2R,5R)‐2‐((R)‐1‐((5‐Amino‐6‐chloropyrimidin‐4‐yl)amino)‐2‐hydroxypropan‐2‐yl)‐5‐methylcyclohexanol* (**23 b**): Prepared from **2 b** and **18 c**. White crystals (80 %); m. p. 213–214 °C; [*α*]20 D=−20.0 (c 0.12, MeOH). All spectroscopic data (^1^H and ^13^C NMR together with HRMS) can be found in the Supporting Information.

*(S)‐3‐((5‐Amino‐6‐chloropyrimidin‐4‐yl)amino)‐2‐((1R,2R,4R)‐2‐hydroxy‐4‐methylcyclohexyl)propane‐1,2‐diol* (**23 c**): Prepared from **4** and **18 c**. Yellow crystals (79 %); m. p. 160–162 °C; [*α*]20 D=−6.0 (c 0.1325, MeOH). All spectroscopic data (^1^H and ^13^C NMR together with HRMS) can be found in the Supporting Information.

*(1R,2S,5R)‐2‐((R)‐1‐((5‐Amino‐6‐chloropyrimidin‐4‐yl)amino)‐3‐hydroxypropan‐2‐yl)‐5‐methylcyclohexanol* (**23 d**): Prepared from **6** and **18 c**. Yellow crystals (88 %); m. p. 187–189 °C; [*α*]20 D=−32.0 (c 0.1375, MeOH). All spectroscopic data (^1^H and ^13^C NMR together with HRMS) can be found in the Supporting Information.

*(3S,3aR,6R,7aR)‐3‐(((5‐Amino‐6‐chloropyrimidin‐4‐yl)amino)methyl)‐6‐methyloctahydrobenzofuran‐3‐ol* (**23 e**): Prepared from **8 a** and **18 c**. White crystals (88 %); m. p. 171–172 °C; [*α*]20 D=−30.0 (c 0.1425, MeOH). All spectroscopic data (^1^H and ^13^C NMR together with HRMS) can be found in the Supporting Information.

*(1S,2R,5R)‐2‐((S)‐1‐((5‐Amino‐6‐chloropyrimidin‐4‐yl)amino)‐2‐hydroxypropan‐2‐yl)‐5‐methylcyclohexanol* (**23 f**): Prepared from **10** and **18 c**. Brown oil (83 %); [*α*]20 D=−7.0 (c 0.1825, MeOH). All spectroscopic data (^1^H and ^13^C NMR together with HRMS) can be found in the Supporting Information.

*(1S,2S,5R)‐2‐((S)‐1‐((5‐Amino‐6‐chloropyrimidin‐4‐yl)amino)‐3‐hydroxypropan‐2‐yl)‐5‐methylcyclohexanol* (**23 g**): Prepared from **12** and **18 c**. Yellow crystals (90 %); m. p. 182–183 °C; [*α*]20 D=+19.0 (c 0.1150, MeOH). All spectroscopic data (^1^H and ^13^C NMR together with HRMS) can be found in the Supporting Information.

*(3R,3aR,6R,7aS)‐3‐(((5‐Amino‐6‐chloropyrimidin‐4‐yl)amino)methyl)‐6‐methyloctahydrobenzofuran‐3‐ol* (**23 h**): Prepared from **14** and **18 c**. Brown oil (86 %); [*α*]20 D=−6.0 (c 0.1250, MeOH). All spectroscopic data (^1^H and ^13^C NMR together with HRMS) can be found in the Supporting Information.

**General procedure for the preparation of** ***N*** ^**2**^ **‐(*p–*trifluoromethyl)aniline substituted pyrimidines** (**20 a**–**i**) and (**22 a**–**h**): A mixture of pyrimidines **19 a**–**i** or **21 a**–**h** (0.16 mmol) and 4‐trifluoromethylaniline (0.24 mmol) in EtOH (200 μL) was heated in microwave reactor at 150 °C, 200 W, 19 bar for 80 min. The formed precipitate was filtered off and washed with CH_2_Cl_2_ to afford the desired product in pure form without further purification.

*(1R,2R,5R)‐2‐((S)‐1‐((5‐Fluoro‐2‐((4‐(trifluoromethyl)phenyl)amino)pyrimidin‐4‐yl)amino)‐2‐hydroxypropan‐2‐yl)‐5‐methylcyclohexan‐1‐ol hydrochloride* (**20 a**): Prepared from **19 a**. White crystals (72 %); m. p. 154–156 °C; [α]20 D=+43.0 (c 0.1150, MeOH). All spectroscopic data (^1^H and ^13^C NMR together with HRMS) can be found in the Supporting Information.

*(1R,2R,5R)‐2‐((R)‐1‐((5‐Fluoro‐2‐((4‐(trifluoromethyl)phenyl)amino)pyrimidin‐4‐yl)amino)‐2‐hydroxypropan‐2‐yl)‐5‐methylcyclohexan‐1‐ol hydrochloride* (**20 b**): Prepared from **19 b**. Yellow crystals (87 %); m. p. 154–156 °C; [α]20 D=+16.0 (c 0.14, MeOH). All spectroscopic data (^1^H and ^13^C NMR together with HRMS) can be found in the Supporting Information.

*(S)‐3‐((5‐Fluoro‐2‐((4‐(trifluoromethyl)phenyl)amino)pyrimidin‐4‐yl)amino)‐2‐((1R,2R,4R)‐2‐hydroxy‐4‐methylcyclohexyl)propane‐1,2‐diol hydrochloride* (**20 c**): Prepared from **19 c**. White crystals (84 %); m. p. 158–160 °C; [α]20 D=+18.0 (c 0.1075, MeOH). All spectroscopic data (^1^H and ^13^C NMR together with HRMS) can be found in the Supporting Information.

*(1R,2S,5R)‐2‐((R)‐1‐((5‐Fluoro‐2‐((4‐(trifluoromethyl)phenyl)amino)pyrimidin‐4‐yl)amino)‐3‐hydroxypropan‐2‐yl)‐5‐methylcyclohexan‐1‐ol hydrochloride* (**20 d**): Prepared from **19 d**. Yellow crystals (94 %); m. p. 179–181 °C; [α]20 D=−23.0 (c 0.1375, MeOH). All spectroscopic data (^1^H and ^13^C NMR together with HRMS) can be found in the Supporting Information.

*(3S,3aR,6R,7aR)‐3‐(((5‐Fluoro‐2‐((4‐(trifluoromethyl)phenyl)amino)pyrimidin‐4‐yl)amino)methyl)‐6‐methyloctahydrobenzofuran‐3‐ol hydrochloride* (**20 e**): Prepared from **19 e**. White crystals (87 %); m. p. 150–152 °C; [α]20 D=−33.0 (c 0.1350, MeOH). All spectroscopic data (^1^H and ^13^C NMR together with HRMS) can be found in the Supporting Information.

*(3R,3aR,6R,7aR)‐3‐(((5‐Fluoro‐2‐((4‐(trifluoromethyl)phenyl)amino)pyrimidin‐4‐yl)amino)methyl)‐6‐methyloctahydrobenzofuran‐3‐ol hydrochloride* (**20 f**): Prepared from **19 f**. Yellow crystals (90 %); m. p. 156–158 °C; [α]20 D=−40.0 (c 0.1450, MeOH). All spectroscopic data (^1^H and ^13^C NMR together with HRMS) can be found in the Supporting Information.

*(1S,2R,5R)‐2‐((S)‐1‐((5‐Fluoro‐2‐((4‐(trifluoromethyl)phenyl)amino)pyrimidin‐4‐yl)amino)‐2‐hydroxypropan‐2‐yl)‐5‐methylcyclohexan‐1‐ol hydrochloride* (**20 g**): Prepared from **19 g**. White crystals (80 %); m. p. 166–168 °C; [α]20 D=+18.0 (c 0.1350, MeOH). All spectroscopic data (^1^H and ^13^C NMR together with HRMS) can be found in the Supporting Information.

*(1S,2S,5R)‐2‐((S)‐1‐((5‐Fluoro‐2‐((4‐(trifluoromethyl)phenyl)amino)pyrimidin‐4‐yl)amino)‐3‐hydroxypropan‐2‐yl)‐5‐methylcyclohexan‐1‐ol hydrochloride* (**20 h**): Prepared from **20 h**. Yellow crystals (96 %); m. p. 145–147 °C; [α]20 D=+15.0 (c 0.13, MeOH). All spectroscopic data (^1^H and ^13^C NMR together with HRMS) can be found in the Supporting Information.

*(3R,3aR,6R,7aS)‐3‐(((5‐Fluoro‐2‐((4‐(trifluoromethyl)phenyl)amino)pyrimidin‐4‐yl)amino)methyl)‐6‐methyloctahydrobenzofuran‐3‐ol hydrochloride* (**20 i**): Prepared from **19 i**. Yellow crystals (87 %); m. p. 238–240 °C; [α]20 D=−61.0 (c 0.1250, MeOH). All spectroscopic data (^1^H and ^13^C NMR together with HRMS) can be found in the Supporting Information.

*(1R,2R,5R)‐2‐((S)‐1‐((5‐Chloro‐2‐((4‐(trifluoromethyl)phenyl)amino)pyrimidin‐4‐yl)amino)‐2‐hydroxypropan‐2‐yl)‐5‐methylcyclohexan‐1‐ol hydrochloride* (**22 a**): Prepared from **21 a**. White crystals (80 %); m. p. 167–169 °C; [α]20 D=+54.0 (c 0.1225, MeOH). All spectroscopic data (^1^H and ^13^C NMR together with HRMS) can be found in the Supporting Information.

*((1R,2R,5R)‐2‐((R)‐1‐((5‐Chloro‐2‐((4‐(trifluoromethyl)phenyl)amino)pyrimidin‐4‐yl)amino)‐2‐hydroxypropan‐2‐yl)‐5‐methylcyclohexan‐1‐ol hydrochloride* (**22 b**): Prepared from **21 b**. White crystals (82 %); m. p. 82–84 °C; [α]20 D=+12.0 (c 0.1325, MeOH). All spectroscopic data (^1^H and ^13^C NMR together with HRMS) can be found in the Supporting Information.

*(S)‐3‐((5‐Chloro‐2‐((4‐(trifluoromethyl)phenyl)amino)pyrimidin‐4‐yl)amino)‐2‐((1R,2R,4R)‐2‐hydroxy‐4‐methylcyclohexyl)propane‐1,2‐diol hydrochloride* (**22 c**): Prepared from **21 c**. Yellow crystals (90 %); m. p. 163–165 °C; [α]20 D=−4.0 (c 0.1300, MeOH). All spectroscopic data (^1^H and ^13^C NMR together with HRMS) can be found in the Supporting Information.

*(1R,2S,5R)‐2‐((R)‐1‐((5‐Chloro‐2‐((4‐(trifluoromethyl)phenyl)amino)pyrimidin‐4‐yl)amino)‐3‐hydroxypropan‐2‐yl)‐5‐methylcyclohexan‐1‐ol hydrochloride* (**22 d**): Prepared from **21 d**. Yellow crystals (98 %); m. p. 173–175 °C; [α]20 D=29.0 (c 0.1250, MeOH). All spectroscopic data (^1^H and ^13^C NMR together with HRMS) can be found in the Supporting Information.

*(3S,3aR,6R,7aR)‐3‐(((5‐Chloro‐2‐((4‐(trifluoromethyl)phenyl)amino)pyrimidin‐4‐yl)amino)methyl)‐6‐methyloctahydrobenzofuran‐3‐ol hydrochloride* (**22 e**): Prepared from **21 e**. White crystals (85 %); m. p. 188–190 °C; [α]20 D=−23.0 (c 0.1350, MeOH). All spectroscopic data (^1^H and ^13^C NMR together with HRMS) can be found in the Supporting Information.

*(1S,2R,5R)‐2‐((S)‐1‐((5‐Chloro‐2‐((4‐(trifluoromethyl)phenyl)amino)pyrimidin‐4‐yl)amino)‐2‐hydroxypropan‐2‐yl)‐5‐methylcyclohexan‐1‐ol hydrochloride* (**22 f**): Prepared from **21 f**. White crystals (87 %); m. p. 137–140 °C; [α]20 D=+29.0 (c 0.1325, MeOH). All spectroscopic data (^1^H and ^13^C NMR together with HRMS) can be found in the Supporting Information.

*(1S,2S,5R)‐2‐((S)‐1‐((5‐Chloro‐2‐((4‐(trifluoromethyl)phenyl)amino)pyrimidin‐4‐yl)amino)‐3‐hydroxypropan‐2‐yl)‐5‐methylcyclohexan‐1‐ol hydrochloride* (**22 g**): Prepared from **21 g**. White crystals (95 %); m. p. 162–164 °C; [α]20 D=+2.0 (c 0.1150, MeOH). All spectroscopic data (^1^H and ^13^C NMR together with HRMS) can be found in the Supporting Information.

*(3R,3aR,6R,7aS)‐3‐(((5‐Chloro‐2‐((4‐(trifluoromethyl)phenyl)amino)pyrimidin‐4‐yl)amino)methyl)‐6‐methyloctahydrobenzofuran‐3‐ol hydrochloride* (**22 h**): Prepared from **21 h**. Yellow crystals (85 %); m. p. 223–225 °C; [[α]20 D=−30.0 (c 0.1350, MeOH). All spectroscopic data (^1^H and ^13^C NMR together with HRMS) can be found in the Supporting Information.

**Determination of antiproliferative effect**: The growth‐inhibitory effects of the presented heterocyclic compounds were determined by a standard MTT (3‐(4,5‐dimethylthiazol‐2‐yl)‐2,5‐diphenyltetrazolium bromide) assay on a panel of human adherent cancer cell lines of gynecological origin containing HeLa and SiHa (cervical cancers), A2780 (ovarian cancer) and MDA‐MB‐231 (breast cancer) cells.^[110]^ All cell lines were purchased from the European Collection of Cell Cultures (Salisbury, UK) except the SiHa, obtained from the American Tissue Culture Collection (Manassas, VA, USA). The cells were cultivated in minimal essential medium (MEM) supplemented with fetal bovine serum (10 %), non‐essential amino acids, and penicillin‐streptomycin (1 % each) at 37 °C in a humidified atmosphere containing 5 % CO_2_. All media and supplements were obtained from Lonza Group Ltd. (Basel, Switzerland). Cancer cells were plated into 96‐well plates at the density of 5000 cells/well. After overnight incubation, the test compound was added in two concentrations (10 μm and 30 μm) and incubated for 72 h under cell‐culturing conditions. Then, MTT solution (5 mg mL^−1^, 20 μL) was added to each well and incubated for 4 h. Finally, the medium was removed, and the precipitated formazan was dissolved in DMSO during 60 min of shaking at 37 °C. The absorbance was measured at 545 nm using a microplate reader (SpectoStarNano, BMG Labtech, Ortenberg, Germany). Two independent experiments were carried out with five wells for each condition. Cisplatin (Ebewe GmbH, Unterach, Austria) was used as a positive control. Calculations were performed utilising the GraphPad Prism 5.01 software (GraphPad Software Inc., San Diego, CA, USA).

## Conflict of interest

The authors declare no conflict of interest.

1

## Supporting information

As a service to our authors and readers, this journal provides supporting information supplied by the authors. Such materials are peer reviewed and may be re‐organized for online delivery, but are not copy‐edited or typeset. Technical support issues arising from supporting information (other than missing files) should be addressed to the authors.

Supporting Information

## Data Availability

The data that support the findings of this study are available in the supplementary material of this article.
