# Supplementary material for: Antiproliferative Activity of (−)‐Isopulegol‐based 1,3‐Oxazine, 1,3‐Thiazine and 2,4‐Diaminopyrimidine Derivatives
Source: ChemistryOpen. 2022 Oct 6;11(10):e202200169. doi: 10.1002/open.202200169 (PMC9535514; doi:10.1002/open.202200169)
Supplement: Supplementary file 1 — Supporting Information [file OPEN-11-e202200169-s001.pdf]

ChemistryOpen

Supporting Information

Antiproliferative Activity of (−)-Isopulegol-based 1,3-Oxazine, 1,3-Thiazine and 2,4-Diaminopyrimidine Derivatives

Fatima Z. Bamou, Tam M. Le, Bizhar A. Tayeb, Seyyed A. S. Tahaei, Renáta Minorics, István Zupkó, and Zsolt Szakonyi*

Contents

1. Experimental section .... S1 – S16
2. Investigation of anti-proliferative activity of (–)-isopulegol-based heterocyclic derivatives .... S17 – S28

2. ^{1}H-, ^{13}C- NMR spectra of new heterocyclic compounds ....S29 – S84

3. Docking study ....S85 – S86

4. References S87

1. Experimental Section

1.2. General methods: ^{1}H– and ^{13}C–NMR spectra were recorded on a Bruker Avance DRX 500 spectrometer (500 and 125 MHz, respectively, δ=0 ppm (TMS)). Chemical shifts (δ) are expressed in [ppm] relative to TMS as internal reference. J values are given in [Hz]. HRMS flow injection analysis was performed with a Thermo Scientific Q Exactive Plus hybrid quadrupole-Orbitrap (Thermo Fisher Scientific, Waltham, MA, USA) mass spectrometer coupled to a Waters Acquity I-Class UPLC™ (Waters, Manchester, UK). Optical rotations were determined with a Perkin–Elmer 341 polarimeter. Melting points were determined on a Kofler apparatus and they are uncorrected. Chromatographic separations were carried out on Merck Kieselgel 60 (230–400 mesh ASTM). Reactions were monitored with Merck Kieselgel 60 F_{254}-precoated TLC plates (0.25 mm thickness). Commercially available reagents were used as obtained from suppliers (Molar Chemicals Ltd., Halásztelek, Hungary; Merck Ltd., Budapest, Hungary and VWR International Ltd., Debrecen, Hungary), while solvents were dried according to standard procedures.

1.3. Starting materials: (–)-Isopulegol (**1**) is commercially available from Merck Co with *ee* = 95%, ($[\alpha]_D^{20}$ = –22.0, neat) and its enatimomer (+)-**1** (*ee* = 90%, $[\alpha]_D^{20}$ = +22.0, neat). (+)-Neoisopulegol (**2**) ($[\alpha]_D^{20}$ = +28.7, c = 17.2, $CHCl_3$) and its enatimomer (–)-**2** ($[\alpha]_D^{20}$ = –22.2, c = 2.0, $CHCl_3$) were synthesized from (–)-**1** and its isomer (+)-**1** following a reported procedure, respectively.[1] (–)-Isopulegol- based aminodiols **2a–b** and **6**,[2] (+)-neoisopulegol-based aminodiols **10** and **12**[3] together with aminotriol **4**[2] as well as aminoalcohols **14**[4] were prepared according to literature procedures. All spectroscopic data were similar to those described therein.

1.4. Docking Study: Aurora A kinase crystal structure was obtained from PDB (protein data bank). ChemBioDraw Ultra 11.0 was used to design the compound for the docking study. Docking study and In-Silico ADMET prediction were performed by Accelrys discovery studio 2.5 software.

1.4.1. Choosing the template crystal structure: Aiming to choose the most valid crystal structure to be used in our study, we first downloaded the available Aurora A crystal structure from PDB.

1.4.2. Preparation of the crystal structure of Aurora A: It is well known that the extracted crystal structure from PDB does not have hydrogen atoms, so firstly, hydrogen atoms must be added by applying several force fields (CHARMm). Adding hydrogen atoms lead to steric hindrance and subsequently to high energy and unstable molecule, which should be minimised. Minimisation of the crystal structure was performed by using adopted basis minimisation aiming at finding the most stable and less energy structure and reducing H-H interactions without affecting the basic protein skeleton atoms. Then, the active site was determined and the sphere surrounded. [5]

1.4.3. Docking study (CDocker): By using CDocker method, we can generate all the possible conformations of the compound in the protein active site. Then the results can be assessed by both the CDocker energy and the number of interactions between the ligand and active site. This method requires preparing the crystal structure (as mentioned before) and preparing the designed compound by using Accelrys Discovery Studio protocol and applying

force field.[6] Before starting this study, it is important to make sure that the used method is valid by comparing the conformation of the reference compound with its conformations generated by the docking method, where RMSD (Root Mean Square Deviation) should not exceed 2A°.

1.5. Experimental section and compound characterisations

(3S,3aR,6R,7aR)-6-Methyl-3-((((S)-1-phenylethyl)amino)methyl)octahydrobenzofuran-3-ol (**8a**) *and (3R,3aR,6R,7aR)-6-Methyl-3-((((S)-1-phenylethyl)amino)methyl)octahydrobenzofuran-3-ol* (**8b**)

m-CPBA (70% purity, 5.87 g, 23.8 mmol) was added at 0 °C to a solution of **7** (11.9 mmol) in CH_2Cl_2 (50 mL) and $Na_2HPO_4 \cdot 12H_2O$ (6.35 g, 35.7 mmol) in water (130 mL), and the mixture was stirred at room temperature. When the reaction was complete, as indicated by TLC (2 h), the mixture was separated and the aqueous phase was extracted with CH_2Cl_2 (100 mL). The organic layer was washed with a 5% KOH solution (3 × 50 mL), then dried (Na_2SO_4) and evaporated to provide a 4:1 mixture of epoxides as a pale-yellow oil, which was added to the solution of (*S*)-methylbenzylamine (0.80 mL, 6.20 mmol) in MeCN (30 mL) and $LiClO_4$ (0.31 g, 2.94 mmol). The mixture was kept at reflux temperature for 6 h. When the reaction was completed (indicated by TLC), the mixture was evaporated to dryness, and the residue was dissolved in water (15 mL) and then extracted with CH_2Cl_2 (3 × 50 mL). The combined organic phase was dried (Na_2SO_4), filtered, and concentrated. The crude product was purified by column chromatography on silica gel with an appropriate solvent mixture ($CHCl_3$: MeOH = 19:1) to provide **8a** and **8b**.

8a: Yellow crystals (33%); m.p. 54–55 °C; $[\alpha]_D^{20}$ = –51.0 (c 0.1275, MeOH); ^{1}H NMR (500 MHz, $CDCl_3$): δ (ppm) = 7.33-7.22 (m, 5H), 3.80-3.69 (m, 3H), 3.57-3.52 (m, 1H), 3.08 (brs, 1H), 2.60 (q, *J* = 12.0 Hz, 2H), 2.11-2.08 (m, 1H), 1.76-1.73 (m, 1H), 1.68-1.63 (m, 1H), 1.52-1.35 (m, 3H), 1.36 (d, *J* = 6.5 Hz, 3H), 1.06-1.00 (m, 1H), 0.96 (d, *J* = 6.6 Hz, 3H), 0.94-0.86 (m, 1H); ^{13}C NMR (125 MHz, $CDCl_3$): δ (ppm) = 144.9 (C_q), 128.6 (CH), 127.2 (CH), 126.5 (CH), 81.4 (CH), 79.7 (CH_2), 78.3 (C_q), 58.7 (CH), 54.8 (CH_2), 53.3 (CH), 40.1 (CH_2), 34.3 (CH_2), 30.9 (CH), 24.4 (CH_3), 22.8 (CH_2), 22.0 (CH_3); HRMS (ESI): *m*/z $[M+H]^+$ calculated for $C_{18}H_{28}NO_2$ 290.2042, found 290.2116.

8b: White crystals (8%); m.p. 68–69 °C; $[\alpha]_D^{20}$ = –46.0 (c 0.21, MeOH); ^{1}H NMR (500 MHz, $CDCl_3$): δ (ppm) = 7.23-7.34 (m, 5H), 3.88-3.86 (m, 1H), 3.75-3.71 (q, *J* = 6.6 Hz, 1H), 3.64 (d, *J* = 9.5 Hz, 1H), 3.14 (td, *J* = 4.0, 11.1 Hz, 1H), 2.56 (q, *J* = 12.3 Hz, 2H), 2.05-2.02 (m, 1H), 1.82-1.77 (m, 1H), 1.70-1.68 (m, 1H), 1.59-1.54 (m, 1H), 1.43-1.31 (m, 1H), 1.36 (d, *J* = 6.6 Hz, 3H), 1.10 (q, *J* = 11.4 Hz, 1H), 0.99-0.86 (m, 2H), 0.95 (d, *J* = 6.6 Hz, 3H); ^{13}C NMR (125 MHz, $CDCl_3$): δ (ppm) = 145.1 (C_q), 128.6 (CH), 127.1 (CH), 126.2 (CH), 81.9 (CH), 78.4 (CH_2), 78.2 (CH_2), 58.8 (CH), 55.1 (CH), 51.9 (CH_2), 40.3 (CH_2), 34.6 (CH_2), 31.0 (CH), 24.1 (CH_3), 23.4 (CH_2), 21.8 (CH_3); HRMS (ESI): *m*/z $[M+H]^+$ calculated for $C_{18}H_{28}NO_2$ 290.2042, found 290.2116.

*(3S,3aR,6R,7aR)-3-(Aminomethyl)-6-methyloctahydrobenzofuran-3-ol (**8c**) and (3R,3aR,6R,7aR)-3-(Aminomethyl)-6-methyloctahydrobenzofuran-3-ol (**8d**)*

Aminoalcohols **8a**–**b** (14.0 mmol) in MeOH (100 mL) were added to a suspension of palladium-on-carbon (5% Pd, 0.22 g) in MeOH (50 mL), and the mixture was stirred under an

H_2 atmosphere (1 atm) at room temperature. After completion of the reaction (as monitored by TLC, 24 h), the mixture was filtered through a Celite pad, and the solution was evaporated to dryness. The crude product was recrystallised in Et_2O, resulting in primary aminoalcohols **8c–d** as white crystals.

8c: White crystals (90%); m.p. 190–192 °C; $[\alpha]_D^{20}$ = –3.0 (c 0.26, MeOH); ^{1}H NMR (500 MHz, DMSO-*d*$_6$): δ (ppm) = 7.88 (brs, 2H), 5.28 (brs, 1H), 3.98 (d, *J* = 9.8 Hz, 1H), 3.60 (d, *J* = 9.9 Hz, 1H), 3.48-3.43 (m, 1H), 2.95-2.85 (m, 2H), 2.00-1.97 (m, 1H), 1.69-1.66 (m, 2H), 1.44-1.37 (m, 1H), 1.29-1.21 (m, 1H), 1.14-1.09 (m, 1H), 0.97-0.85 (m, 5H); ^{13}C NMR (125 MHz, DMSO-*d*$_6$): δ (ppm) = 80.8 (CH), 78.6 (CH_2) , 77.1 (C_q), 52.6 (CH), 46.3 (CH_2), 40.3 (CH_2), 34.3 (CH_2), 30.9 (CH) , 22.4 (CH_2), 22.4 (CH_3); HRMS (ESI): *m*/z $[M+H]^+$ calculated for $C_{10}H_{20}NO_2$ 186.1416, found 186.1491.

8d: White crystals (87%); m.p. 193–196 °C; $[\alpha]_D^{20}$ = –12.0 (c 0.14, MeOH); ^{1}H NMR (500 MHz, DMSO-*d*$_6$): δ (ppm) = 7.97 (brs, 2H), 5.62 (brs, 1H), 3.83 (d, *J* = 9.6 Hz, 1H), 3.61 (d, *J* = 9.6 Hz, 1H), 3.23-3.20 (m, 1H), 2.92 (d, *J* = 13.2 Hz, 1H), 2.81 (d, *J* =12.7 Hz, 1H), 1.98-1.94 (m, 1H), 1.83-1.80 (m, 1H), 1.71-1.68 (m, 1H), 1.55-1.49 (m, 1H), 1.44-1.36 (m, 1H), 1.11-1.03 (m, 1H), 1.01 (q, *J* = 11.2 Hz, 1H), 0.93 (d, *J* = 6.6 Hz, 3H), 0.88-0.81 (m, 1H); ^{13}C NMR (125 MHz, DMSO-*d*$_6$): δ (ppm) = 81.2 (CH), 77.2 (C_q), 76.7 (CH_2), 56.1 (CH), 44.1 (CH_2), 40.5 (CH_2), 34.7 (CH_2), 30.8 (CH), 22.5 (CH_2), 22.2 (CH_3); HRMS (ESI): *m*/z $[M+H]^+$ calculated for $C_{10}H_{20}NO_2$ 186.1416, found 186.1487.

1.5.1. General procedure for the preparation of thioureas (15a–d)

Aminodiols **2a, 6, 10** and **12** (0.53 mmol) and the appropriate phenylisothiocyanate (0.79 mmol) were dissolved in toluene (40 mL), and the mixture was stirred at room temperature for 1 h, except that in the case of **6** when a treatment at reflux temperature for 3 h was carried out. The resulting mixtures were then evaporated then the residue was purified by column chromatography on silica gel (eluted with $CHCl_3$: MeOH = 19:1).

1-((S)-2-Hydroxy-2-((1R,2R,4R)-2-hydroxy-4-methylcyclohexyl)propyl)-3-phenylthiourea (**15a**): Prepared from **2a**. White crystals (95%); m.p. 162–163 °C; $[\alpha]_D^{20}$ = –33.0 (c 0.28, MeOH); ^{1}H NMR (500 MHz, DMSO-*d*$_6$): δ (ppm) = 9.77 (s, 1H), 7.51 (d, *J* =7.7 Hz, 2H), 7.45 (brs, 1H), 7.30 (t, *J* = 5.7 Hz, 2H), 7.10-7.06 (m, 1H), 5.74 (s, 1H), 5.62 (s, 1H), 3.65-3.63 (m, 2H), 3.30 (d, *J* = 3.9 Hz, 1H), 1.83 (d, *J* = 12.0 Hz, 1H), 1.68 (d, *J* = 11.2 Hz, 1H), 1.58 (d, *J* = 12.1 Hz, 1H), 1.40-1.35 (m, 2H), 1.12 (s, 3H), 0.96-0.81 (m, 6H); ^{13}C NMR (125 MHz, DMSO-*d*$_6$): δ (ppm) = 128.9 (CH), 124.4 (CH), 123.0 (CH), 71.5 (CH), 53.1 (CH_2), 48.9 (CH), 45.3 (CH_2), 34.6 (CH_2), 31.1 (CH), 26.2 (CH_2), 22.4 (CH_3), 21.8 (CH_3); HRMS (ESI): *m*/z $[M+H]^+$ calculated for $C_{17}H_{27}N_2O_2S$ 323.1715, found 323.1788.

1-((R)-3-Hydroxy-2-((1S,2R,4R)-2-hydroxy-4-methylcyclohexyl)propyl)-3-phenylthiourea (**15b**): Prepared from **6**. White crystals (58%); m.p. 97–98 °C; $[\alpha]_D^{20}$ = –22.0 (c 0.28, MeOH); ^{1}H NMR (500 MHz, $CDCl_3$): δ (ppm) = 7.77 (brs, 1H), 7.41 (t, *J* = 7.7 Hz, 2H), 7.28 (t, *J* = 7.1 Hz, 1H), 7.22 (d, *J* = 7.1 Hz, 2H), 6.98-6.92 (m, 1H), 4.08-3.99 (m, 1H), 3.72-3.63 (m, 2H), 3.59-3.47 (m, 2H), 3.01 (s, 1H), 2.05-2.04 (m, 1H), 1.93-1.91 (m, 1H), 1.69 (brs, 1H), 1.65-1.61 (m, 2H), 1.46-1.37 (m, 1H), 1.32-1.27 (m, 1H), 1.11-1.04 (m, 1H), 1.00-0.93 (m, 1H), 0.90 (d, *J* = 6.56 Hz, 3H), 0.88-0.80 (m, 1H); ^{13}C NMR (125 MHz, $CDCl_3$): δ (ppm) = 180.4 (C_q), 130.0 (CH), 127.4 (CH), 125.5(CH), 71.0 (CH), 63.9 (CH_2), 46.4 (CH_2), 45.8 (CH), 44.3 (CH_2),

42.4 (CH), 34.4 (CH_2), 31.5 (CH), 27.8 (CH_2), 22.0 (CH_3); HRMS (ESI): *m*/z $[M+H]^+$ calculated for $C_{17}H_{27}N_2O_2S$ 323.1715, found 323.1784.

1-((S)-2-Hydroxy-2-((1R,2S,4R)-2-hydroxy-4-methylcyclohexyl)propyl)-3-phenylthiourea (**15c**): Prepared from **10**. Yellow oil (61%); $[\alpha]_D^{20}$ = –5.0 (c 0.295, MeOH); ^{1}H NMR (500 MHz, $CDCl_3$): δ (ppm) = 7.69 (brs, 1H), 7.42 (t, *J* = 7.5 Hz, 2H), 7.30-7.25 (m, 2H), 6.70 (brs, 1H), 4.39 (s, 1H), 3.79 (dd, *J* = 4.2, 13.8 Hz, 1H), 3.72-3.52 (m, 1H), 2.05 (brs, 1H), 1.79-1.61 (m, 5H), 1.28 (s, 3H), 1.25-1.24 (m, 1H), 1.15-1.09 (t, *J* = 13.0 Hz, 1H); 0.95-0.86 (m, 1H), 0.86-0.88 (d, *J* = 6.1 Hz, 3H); ^{13}C NMR (125 MHz, $CDCl_3$): δ (ppm) = 130.1 (CH), 127.3 (CH), 125.2 (CH), 68.1 (CH), 52.9 (CH_2), 47.2 (CH), 42.8 (CH_2), 34.6 (CH_2), 25.7 (CH_3), 24.9 (CH), 22.0 (CH_3), 20.4 (CH_2); HRMS (ESI): *m*/z $[M+H]^+$ calculated for $C_{17}H_{27}N_2O_2S$ 323.1715, found 323.1788.

1-((S)-3-Hydroxy-2-((1S,2S,4R)-2-hydroxy-4-methylcyclohexyl)propyl)-3-phenylthiourea (**15d**): Prepared from **12**. White crystals (94%); m.p. 50–52 °C; $[\alpha]_D^{20}$ = +25.0 (c 0.265, MeOH); ^{1}H NMR (500 MHz, $CDCl_3$): δ (ppm) = 7.81 (brs, 1H), 7.40 (t, *J* = 7.8 Hz, 2H), 7.30-7.27 (t, *J* = 7.4 Hz, 1H), 7.21 (d, *J* = 7.7 Hz, 2H), 4.00 (s, 1H), 3.95-3.92 (m, 1H), 3.67-3.58 (m, 3H), 2.34 (brs, 2H), 1.82-1.68 (m, 4H), 1.52-1.37 (m, 3H), 1.15-1.10 (t, *J* = 12.4 Hz, 1H), 0.94-0.84 (m, 1H), 0.85 (d, *J* = 6.2 Hz, 3H); ^{13}C NMR (125 MHz, $CDCl_3$): δ (ppm) = 136.2 (Cq), 130.0 (CH), 127.4 (CH), 125.6 (CH), 67.4 (CH), 61.7 (CH_2), 45.0 (C_q), 42.9 (CH), 42.6 (CH_2), 40.5 (CH), 34.8 (CH_2), 25.8 (CH), 24.0 (CH_2), 22.1 (CH_3); HRMS (ESI): *m*/z $[M+H]^+$ calculated for $C_{17}H_{27}N_2O_2S$ 323.1715, found 323.1793.

1.5.2. General procedure for the preparation of 1,3–thiazines (16a–b)

A solution of thioureas **15b** or **15d** (0.31 mmol) in dry EtOH (1 mL) was added 22% HCl in EtOH (5 mL) and the mixture was stirred at room temperature for 4 h and then concentrated under vacuum. The residue was treated with 10% KOH in MeOH (20 mL) followed by evaporation, and the crude product was again dissolved in water (10 mL) and extracted with $CHCl_3$ (3 × 20 mL). The combined organic layer was washed with saturated NaCl aqueous solution (15 mL), dried (Na_2SO_4) and concentrated under vacuum. The crude product was purified by column chromatography on silica gel with $CHCl_3$:MeOH = 19:1.

(1R,2S,5R)-5-Methyl-2-((R)-2-(phenylimino)-1,3-thiazinan-5-yl)cyclohexanol (**16a**): Prepared from **15b**. White crystals (71%); m.p. 73–75 °C; $[\alpha]_D^{20}$ = –46.0 (c 0.9, MeOH); ^{1}H NMR (500 MHz, $CDCl_3$): δ (ppm) = 7.31-7.28 (m, 2H), 7.16-7.09 (m, 3H), 3.58-3.55 (m, 1H), 3.43 (td, *J* = 4.1, 10.4 Hz, 1H), 3.38-3.33 (m, 1H), 3.13 (t, *J* = 11.6 Hz, 1H), 2.87-2.84 (m, 1H), 2.48-2.43 (m, 1H), 1.97-1.95 (m, 1H), 1.70-1.66 (m, 2H), 1.49-1.35 (m, 2H), 1.12-1.04 (m, 1H), 1.00 (q, *J* = 12.0 Hz, 1H), 0.91 (d, *J* = 6.6 Hz, 3H), 0.89-0.84 (m, 1H); ^{13}C NMR (125 MHz, $CDCl_3$): δ (ppm) = 129.0 (CH), 124.6 (CH), 123.2 (CH), 71.3 (CH), 47.5 (CH), 45.3 (CH_2), 45.0 (CH_2), 34.2 (CH_2), 34.1 (CH), 31.3 (CH), 30.4 (CH_2), 26.3 (CH_2), 21.9 (CH_3); HRMS (ESI): *m*/z $[M+H]^+$ calculated for $C_{17}H_{25}N_2OS$ 305.1609, found 305.1679.

(1S,2S,5R)-5-Methyl-2-((S)-2-(phenylimino)-1,3-thiazinan-5-yl)cyclohexanol (**16b**): Prepared from **15d**. White crystals (88%); m.p. 224–225 °C; $[\alpha]_D^{20}$ = +9.0 (c 0.29, MeOH); ^{1}H NMR (500 MHz, DMSO-d_6): δ (ppm) = 7.56 (brs, 1H), 7.15 (t, *J* = 7.6 Hz, 2H), 6.83-6.60 (m, 1H), 4.33 (d, *J* = 7.8 Hz, 1H), 3.89 (s, 1H), 3.70 (brs, 1H), 3.11 (brs, 1H), 2.80 (brs, 1H), 1.75-1.51 (m, 5H), 1.38-1.35 (m, 1H), 1.20 (brs, 1H), 1.02-0.97 (m, 1H), 0.84-0.80 (m, 1H), 0.81 (d, 3H, *J* = 6.4 Hz); ^{13}C NMR (125 MHz, DMSO-d_6): δ (ppm) = 128.8 (CH), 128.7 (CH), 65.7

(CH), 43.1 (CH_2), 41.8 (CH_2), 35.1 (CH_2), 30.1 (CH_2), 25.7 (CH), 23.6 (CH_2), 22.8 (CH_3); HRMS (ESI): *m*/z $[M+H]^+$ calculated for $C_{17}H_{25}N_2OS$ 305.1609, found 305.1686.

1.5.3. General procedure for the synthesis of 1.3-oxazines (17a–d)

To a solution of **15a–d** (0.31 mmol) in MeOH (4 mL), MeI (1.50 mmol) was added. After 3 h stirring at room temperature, the mixture was evaporated, followed by adding 2.5 M KOH in MeOH (20 mL) and subsequently stirred for 1 h before evaporation. The residue was dissolved in water (20 mL) and extracted with $CHCl_3$ (3 × 20 mL). The organic phase was then dried with Na_2SO_4 and evaporated to dryness. The crude product was purified by column chromatography on silica gel ($CHCl_3$:MeOH = 19:1).

(1R,2R,5R)-5-Methyl-2-((S)-5-methyl-2-(phenylimino)oxazolidin-5-yl)cyclohexanol (**17a**): Prepared from **15a**. White crystals (76%); m.p. 94–96 °C; $[\alpha]_D^{20}$ = −5.0 (c 0.28, MeOH); ^1H NMR (500 MHz, $CDCl_3$): δ (ppm) = 7.32-7.27 (m, 4H), 7.00-6.96 (m, 1H), 3.76-3.69 (m, 2H), 3.54 (d, *J* = 11.4 Hz, 1H), 3.22 (brs, 2H), 2.05-2.01 (m, 1H), 1.70-1.56 (m, 3H), 1.50-1.44 (m, 1H), 1.43 (s, 3H), 1.07-0.98 (m, 2H), 0.95-0.84 (m, 1H), 0.93 (d, *J* = 6.5 Hz, 3H); ^{13}C NMR (125 MHz, $CDCl_3$): δ (ppm) = 155.0 (C_q), 129.0 (CH), 122.7 (CH), 119.3 (CH), 89.6 (C_q), 71.1 (CH), 51.9 (CH), 43.6 (CH_2), 34.0 (CH_2), 31.1 (CH), 26.3 (CH_2), 21.9 (CH_3), 20.4 (CH_3); HRMS (ESI): *m*/z $[M+H]^+$ calculated for $C_{17}H_{25}N_2O_2$ 289.1838, found 289.1909.

(1R,2S,5R)-5-Methyl-2-((R)-2-(phenylimino)-1,3-oxazinan-5-yl)cyclohexanol (**17b**): Prepared from **15b**. White crystals (50%); m.p. 166–168 °C; $[\alpha]_D^{20}$ = −37.0 (c 0.25, MeOH); ^1H NMR (500 MHz, $CDCl_3$): δ (ppm) = 7.32-7.26 (m, 4H), 7.10-7.07 (m, 1H), 6.75 (s, 1H), 5.06 (t, *J* = 5.4 Hz, 1H), 3.99 (t, *J* = 8.7 Hz, 1H), 3.61 (t, *J* = 7.9 Hz, 1H), 3.42-3.37 (m, 1H), 3.28-3.23 (m, 1H), 3.19-3.15 (m, 1H), 2.16-2.03 (m, 2H), 1.92-1.89 (m, 1H), 1.71-1.68 (m, 1H), 1.51-1.41 (m, 1H), 1.15-1.00 (m, 3H), 0.96 (d, *J* = 6.6 Hz, 3H), 0.94-0.83 (m, 1H); ^{13}C NMR (125 MHz, $CDCl_3$): δ (ppm) = 155.9 (C_q), 138.4 (C_q), 129.3 (CH), 124.0 (CH), 121.2 (CH), 84.0 (CH), 71.0 (CH_2), 49.0 (CH), 43.8 (CH), 42.1 (CH_2), 39.7 (CH_2), 34.6 (CH_2), 31.2 (CH), 27.3 (CH_2), 22.0 (CH_3); HRMS (ESI): *m*/z $[M+H]^+$ calculated for $C_{17}H_{25}N_2O_2$ 289.1838, found 289.1909.

(1S,2R,5R)-5-Methyl-2-((S)-5-methyl-2-(phenylimino)oxazolidin-5-yl)cyclohexanol (**17c**): Prepared from **15c**. White crystals (90%); m.p. 94–95 °C; $[\alpha]_D^{20}$ = −16.0 (c 0.25, MeOH); ^1H NMR (500 MHz, $CDCl_3$): δ (ppm) = 7.31 (brs, 1H), 7.27 (t, *J* = 7.4 Hz, 2H), 7.00-6.96 (m, 1H), 4.30 (s, 1H), 3.89 (d, *J* = 11.4 Hz, 1H), 3.52-3.48 (m, 1H), 1.90-1.78 (m, 3H), 1.71-1.59 (m, 2H), 1.52 (s, 3H), 1.47-1.45 (m, 1H), 1.09-1.04 (m, 1H), 0.98-0.90 (m, 1H), 0.88-0.87 (d, *J* = 6.1 Hz, 3H); ^{13}C NMR (125 MHz, $CDCl_3$): δ (ppm) = 129.0 (CH), 122.4 (CH), 66.6 (CH), 49.0 (CH), 42.3 (CH_2), 34.7 (CH_2), 25.7 (CH), 24.8 (CH_3), 22.1 (CH_3), 20.3 (CH_2); HRMS (ESI): *m*/z $[M+H]^+$ calculated for $C_{17}H_{25}N_2O_2$ 289.1838, found 289.1913.

(1S,2S,5R)-5-Methyl-2-((S)-2-(phenylimino)-1,3-oxazinan-5-yl)cyclohexanol (**17d**): Prepared from **15d**. White crystals (83%); m.p. 171–172 °C; $[\alpha]_D^{20}$ = +49.0 (c 0.27, MeOH); ^1H NMR (500 MHz, DMSO-d_6): δ (ppm) = 8.13 (brs, 1H), 7.43 (s, 1H), 7.13 (t, *J* = 7.5 Hz, 2H), 6.78 (t, *J* = 7.1 Hz, 1H), 4.34 (d, *J* = 4.1 Hz, 1H), 4.30-4.27 (m, 1H), 3.86 (t, *J* = 10.0 Hz, 1H), 3.82 (s, 1H), 3.48-3.45 (m, 1H), 3.09-3.04 (m, 1H), 1.85-1.82 (m, 1H), 1.75-1.69 (m, 2H), 1.64-1.61 (m, 1H), 1.47-1.36 (m, 2H), 1.11-1.07 (m, 1H), 1.01-096 (m, 1H), 0.83-0.80 (m, 1H), 0.81 (d, *J* = 6.4 Hz, 3H); ^{13}C NMR (125 MHz, DMSO-d_6): δ = (ppm) 128.6 (CH), 120.4 (CH), 68.5 (CH_2), 65.4 (CH), 42.9 (CH_2), 41.4 (CH), 40.5 (CH_2), 34.9 (CH_2), 33.4 (CH), 25.7 (CH), 23.4

(CH_2), 22.89 (CH_3); HRMS (ESI): *m*/z $[M+H]^+$ calculated for $C_{17}H_{25}N_2O_2$ 289.1838, found 289.1912.

1.5.4. General procedure for the preparation of pyrimidine analogues (19a–h), (21a–g) and (23a–g)

To a solution of aminodiols **2a–b**, **6**, **10**, and **12** together with aminotriol **4** as well as aminoalcohols **8a–b**, **14** (0.6 mmol) in EtOH (2 mL), 2,4-dichloro-5-fluoropyrimidine **18a**, 2,4,5-trichloropyrimidine **18b** or 4,6-dichloropyrimidine-5-amine **18c** (0.6 mmol) and Et_3N (1.8 mmol, 182 mg) were added. After a treatment at reflux temperature for 24 h, the reaction mixture was cooled to room temperature and concentrated under reduced pressure. The crude product was dissolved in EtOAc (15 mL) and washed with H_2O (3 × 15 mL). The organic layer was dried (Na_2SO_4) and concentrated under reduced pressure. The residue was purified by column chromatography on silica gel and eluted with $CHCl_3$: MeOH = 19:1.

(1R,2R,5R)-2-((S)-1-((2-Chloro-5-fluoropyrimidin-4-yl)amino)-2-hydroxypropan-2-yl)-5-methylcyclohexanol (**19a**): Prepared from **2a** and **18a**. Brown crystals (94%); m.p. 66–69 °C; $[\alpha]_D^{20}$ = +63.0 (c 0.13, MeOH); ^{1}H NMR (500 MHz, $CDCl_3$): δ (ppm) = 7.86 (d, *J* = 2.7 Hz, 1H), 5.85 (s, 1H), 5.05 (brs, 1H), 3.85 (td, *J* = 4.1, 10.4 Hz, 1H), 3.70 (dd, *J* = 6.7, 13.4 Hz, 1H), 3.32 (dd, *J* = 4.4, 13.5 Hz, 1H), 2.31 (brs, 1H), 1.95-1.91 (m, 1H), 1.78-1.73 (m, 1H), 1.69-1.65 (m, 1H), 1.53-1.43 (m, 2H), 1.24 (s, 3H), 1.10 (q, *J* = 11.9 Hz, 1H), 1.02-0.94 (m, 1H), 0.92 (d, *J* = 6.5 Hz, 3H), 0.89-0.79 (m, 1H); ^{13}C NMR (125 MHz, $CDCl_3$): δ (ppm) = 154.1 (C_q), 146.3 (C_q), 144.3 (C_q), 139.4 (CH), 75.2 (C_q), 73.0 (CH), 49.1 (CH), 49.2 (CH_2), 45.3 (CH_2), 34.1 (CH_2), 31.3 (CH), 26.4 (CH_2), 21.8 (CH_3), 21.1 (CH_3); HRMS (ESI): *m*/z $[M+H]^+$ calculated for $C_{14}H_{22}ClFN_3O_2$ 318.1306, found 318.1376.

(1R,2R,5R)-2-((R)-1-((2-Chloro-5-fluoropyrimidin-4-yl)amino)-2-hydroxypropan-2-yl)-5-methylcyclohexanol (**19b**): Prepared from **2b** and **18a**. White crystals (88%); m.p. 100–104 °C; $[\alpha]_D^{20}$ = +4.0 (c 0.13, MeOH); ^{1}H NMR (500 MHz, $CDCl_3$): δ (ppm) = 7.85 (d, *J* = 2.7 Hz, 1H), 5.95 (s, 1H), 4.78 (s, 1H), 3.85-3.80 (m, 1H), 3.74 (dd, *J* = 7.7, 13.3 Hz, 1H), 3.51 (dd, *J* = 3.1, 13.3 Hz, 1H), 2.61 (s, 1H), 1.95-1.92 (m, 1H), 1.87-1.82 (m, 1H), 1.75-1.72 (m, 1H), 1.61-1.55 (m, 1H), 1.53-1.45 (m, 1H), 1.20 (s, 3H), 1.16-1.05 (m, 2H), 0.97-0.86 (m, 1H), 0.94 (d, *J* = 6.8 Hz, 3H); ^{13}C NMR (125 MHz, $CDCl_3$): δ (ppm) = 154.5 (C_q), 146.4 (C_q), 144.4 (C_q), 139.4 (CH), 75.2 (C_q), 73.1 (CH), 52.4 (CH), 46.8 (CH_2), 45.6 (CH_2), 34.4 (CH_2), 31.5 (CH), 26.3 (CH_2), 24.9 (CH_3), 21.8 (CH_3); HRMS (ESI): *m*/z $[M+H]^+$ calculated for $C_{14}H_{22}ClFN_3O_2$ 318.1306, found 318.1377.

(*S*)-3-((2-Chloro-5-fluoropyrimidin-4-yl)amino)-2-((1*R*,2*R*,4*R*)-2-hydroxy-4-methylcyclohexyl)propane-1,2-diol (**19c**): Prepared from **4** and **18a**. Yellow oil (94%); $[\alpha]_D^{20}$ = +8.0 (c 0.1375, MeOH); ^{1}H NMR (500 MHz, $CDCl_3$): δ (ppm) 7.86 (d, *J* = 2.6 Hz, 1H), 6.18 (s, 1H), 5.22 (s, 1H), 3.90-3.85 (m, 1H), 3.74-3.54 (m, 4H), 2.85 (s, 1H), 2.74 (s, 1H), 1.97-1.95 (m, 1H), 1.82-1.70 (m, 4H), 1.53-1.43 (m, 1H), 1.14-1.07 (m, 2H), 0.96-0.88 (m, 1H), 0.94 (d, *J* = 6.6 Hz, 3H); ^{13}C NMR (125 MHz, $CDCl_3$): δ (ppm) = 154.4 (C_q), 146.5 (C_q), 144.4 (C_q), 139.3 (CH), 76.0 (C_q), 72.4 (CH), 66.1 (CH_2), 48.0 (CH), 45.4 (CH_2), 43.9 (CH_2), 34.1 (CH_2), 31.2 (CH), 25.5 (CH_2), 21.8 (CH_3); HRMS (ESI): *m*/z $[M+H]^+$ calculated for $C_{14}H_{22}ClFN_3O_3$ 334.1255, found 334.1322.

*(1R,2S,5R)-2-((R)-1-((2-Chloro-5-fluoropyrimidin-4-yl)amino)-3-hydroxypropan-2-yl)-5-methylcyclohexanol (***19d**): Prepared from **6** and **18a**. White crystals (84%); m.p. 172–174 °C;

$[\alpha]_D^{20}$ = –55.0 (c 0.13, MeOH); ^{1}H NMR (500 MHz, DMSO-*d*$_6$): δ (ppm) = 8.03 (d, *J* = 3.2 Hz, 1H); 8.01-7.97 (m, 1H), 4.60 (t, *J* = 4.9 Hz, 1H), 4.56 (d, *J* = 5.1 Hz, 1H), 3.49-3.47 (m, 2H), 3.45-3.40 (m, 1H), 3.28-3.22 (m, 1H), 2.19-2.17 (m, 1H), 1.86-1.84 (m, 1H), 1.60-1.57 (m, 2H), 1.37-1.33 (m, 2H), 1.13-1.04 (m, 1H), 0.89-0.72 (m, 2H), 0.86 (d, *J* = 6.8 Hz, 3H); ^{13}C NMR (125MHz, DMSO-*d*$_6$): δ (ppm) = 154.0 (C_q), 146.7 (C_q), 144.7 (C_q), 139.6 (CH), 69.9 (CH), 62.1 (CH_2), 45.5 (CH_2), 45.2 (CH), 40.7 (CH_2), 40.6 (CH), 34.9 (CH_2), 31.4 (CH), 26.7 (CH_2), 22.7 (CH_3); HRMS (ESI): *m*/z $[M+H]^+$ calculated for $C_{14}H_{22}ClFN_3O_2$ 318.1306, found 318.1382.

*(3S,3aR,6R,7aR)-3-(((2-Chloro-5-fluoropyrimidin-4-yl)amino)methyl)-6-methyloctahydrobenzofuran-3-ol (***19e**): Prepared from **8a** and **18a**. White crystals (53%); m.p. 175–177 °C; $[\alpha]_D^{20}$ = –3.0 (c 0.1150, MeOH); ^{1}H NMR (500 MHz, DMSO-*d*$_6$): δ (ppm) = 8.09-8.07 (m, 2H), 4.86 (s, 1H), 3.93 (d, *J* = 9.7 Hz, 1H), 3.58 (dd, *J* = 6.5, 13.6 Hz, 1H), 3.51 (d, *J* = 9.6 Hz, 1H), 3.44-3.39 (m, 2H), 1.95-1.93 (m, 1H), 1.76-1.75 (m, 1H), 1.67-1.64 (m, 1H), 1.40-1.35 (m, 1H), 1.27-1.17 (m, 1H), 1.05-0.99 (m, 1H), 0.90 (d, *J* = 6.5 Hz, 3H), 0.88-0.84 (m, 1H), 0.83-0.74 (m, 1H); ^{13}C NMR (125 MHz, DMSO-*d*$_6$): δ (ppm) = 154.3 (C_q), 153.8 (C_q), 146.6 (C_q), 144.5 (C_q), 140.4 (CH), 80.5 (CH), 79.2 (CH_2), 77.9 (CH_2), 51.5 (CH), 45.1 (CH_2), 34.4 (CH_2), 31.0 (CH), 22.4 (CH_3), 22.3 (CH_2); HRMS (ESI): *m*/z $[M+H]^+$ calculated for $C_{14}H_{20}ClFN_3O_2$ 316.1149, found 316.1221.

*(3R,3aR,6R,7aR)-3-(((2-Chloro-5-fluoropyrimidin-4-yl)amino)methyl)-6-methyloctahydrobenzofuran-3-ol (***19f**): Prepared from **8b** and **18a**. Colorless oil (70%); $[\alpha]_D^{20}$ = –3.0 (c 0.1425, MeOH); ^{1}H NMR (500 MHz, $CDCl_3$): δ (ppm) = 7.90 (s, 1H), 5.72 (s, 1H), 3.87-3.79 (m, 2H), 3.70-3.60 (m, 2H), 3.35 (td, *J* = 3.8, 10.9 Hz, 1H), 2.67 (s, 1H), 2.17-2.11 (m, 1H), 1.95-1.91 (m, 1H), 180-1.76 (m, 1H), 1.61-1.56 (m, 1H), 1.52-1.43 (m, 1H), 1.21-1.11 (m, 2H), 0.99 (d, *J* = 6.6 Hz, 3H), 0.98-0.89 (m, 1H); ^{13}C NMR (125 MHz, $CDCl_3$): δ (ppm) = 154.5 (C_q), 146.3 (C_q), 144.3 (C_q), 139.8 (CH), 82.2 (CH), 80.1 (C_q), 78.3 (CH_2), 56.8 (CH), 45.4 (CH_2), 40.1 (CH_2), 34.6 (CH_2), 30.9 (CH), 23.09 (CH_2), 21.9 (CH_3); HRMS (ESI): *m*/z $[M+H]^+$ calculated for $C_{14}H_{20}ClFN_3O_2$ 316.1149, found 316.1220.

(1S,2R,5R)-2-((S)-1-((2-Chloro-5-fluoropyrimidin-4-yl)amino)-2-hydroxypropan-2-yl)-5-methylcyclohexanol (**19g**): Prepared from **10** and **18a**. White crystals (75%); m.p. 158–160 °C; $[\alpha]_D^{20}$ = +36.0 (c 0.1450, MeOH); ^{1}H NMR (500 MHz, $CDCl_3$): δ (ppm) = 7.85 (d, 1H), 5.93 (brs, 1H), 4.46 (s, 1H), 3.61-3.57 (m, 2H), 3.52-3.49 (m, 1H), 2.11 (s, 1H), 1.83-1.65 (m, 4H), 1.36-1.35 (m, 1H), 1.33 (s, 3H), 1.20-1.14 (m, 1H), 0.97-0.92 (m, 1H), 0.90 (d, *J* = 6.2 Hz, 3H); ^{13}C NMR (125 MHz, $CDCl_3$): δ (ppm) = 154.5 (C_q), 146.4 (C_q), 144.4 (C_q), 139.4 (CH), 74.0 (C_q), 68.3 (CH), 48.3 (CH_2), 47.0 (CH), 42.9 (CH_2), 34.5 (CH_2), 25.7 (CH), 24.7 (CH_3), 22.1 (CH_3), 20.5 (CH_2); HRMS (ESI): *m*/z $[M+H]^+$ calculated for $C_{14}H_{22}ClFN_3O_2$ 318.1306, found 318.1378.

(1S,2S,5R)-2-((S)-1-((2-Chloro-5-fluoropyrimidin-4-yl)amino)-3-hydroxypropan-2-yl)-5-methylcyclohexanol (**19h**): Prepared from **12** and **18a**. White crystals (88%); m.p. 142–144 °C; $[\alpha]_D^{20}$ = +26.0 (c 0.1250, MeOH); ^{1}H NMR (500 MHz, $CDCl_3$): δ (ppm) = 7.84 (s, 1H), 6.26 (s, 1H), 4.13 (s, 1H), 3.80-3.57 (m, 4H), 2.24 (brs, 1H), 1.91-1.75 (m, 4H), 1.65-1.47 (m, 3H), 1.29-1.17 (m, 1H), 1.03-0.90 (m, 1H), 0.89 (d, *J* = 6.8 Hz, 3H); ^{13}C NMR (125 MHz, $CDCl_3$): δ (ppm) = 146.4 (C_q), 144.3 (C_q), 139.2 (CH), 67.7 (CH), 62.0 (CH_2), 42.9 (CH_2), 42.7 (CH), 40.6 (CH_2), 40.4 (CH), 34.8 (CH_2), 25.8 (CH), 23.9 (CH_2), 22.1 (CH_3); HRMS (ESI): m/z $[M+H]^+$ calculated for $C_{14}H_{22}ClFN_3O_2$ 318.1306, found 318.1372.

(3R,3aR,6R,7aS)-3-(((2-Chloro-5-fluoropyrimidin-4-yl)amino)methyl)-6-methyloctahydrobenzofuran-3-ol (**19i**): Prepared from **14** and **18a**. White crystals (72%); m.p. 156–158 °C; $[\alpha]_D^{20}$ = –7.0 (c 0.1375, MeOH); ^{1}H NMR (500 MHz, $CDCl_3$): δ (ppm) = 7.90 (d, *J* = 2.6 Hz, 1H), 5.65 (brs, 1H), 4.42-4.41 (m, 1H), 4.03 (d, *J* = 9.9 Hz, 1H), 3.78-3.73 (m, 2H), 3.66 (dd, *J* = 5.1, 13.9 Hz, 1H), 2.36 (s, 1H), 2.09-2.05 (m, 1H), 1.85-1.81 (m, 1H), 1.69-1.60 (m, 3H), 1.21-1.12 (m, 2H), 0.90 (d, *J* = 6.4 Hz, 3H), 0.86-0.81 (m, 1H); ^{13}C NMR (125 MHz, $CDCl_3$): δ (ppm) = 154.5 (C_q), 146.3 (C_q), 144.3 (C_q), 140.0 (CH), 84.0 (C_q), 77.6 (CH), 76.6 (CH_2), 46.9 (CH), 44.0 (CH_2), 36.8 (CH_2), 32.7 (CH_2), 26.3 (CH), 24.2 (CH_2), 22.1 (CH_3); HRMS (ESI): *m*/z $[M+H]^+$ calculated for $C_{14}H_{20}ClFN_3O_2$ 316.1149, found 316.1220.

(1R,2R,5R)-2-((S)-1-((2,5-Dichloropyrimidin-4-yl)amino)-2-hydroxypropan-2-yl)-5-methylcyclohexanol (**21a**): Prepared from **2a** and **18b**. White crystals (73%); m.p. 110–113 °C; $[\alpha]_D^{20}$ = +66.0 (c 0.15, MeOH); ^{1}H NMR (500 MHz, $CDCl_3$): δ (ppm) = 8.00 (s, 1H), 6.16 (s, 1H), 5.09 (brs, 1H), 3.85 (td, *J* = 4.3, 10.5 Hz, 1H), 3.70 (dd, *J* = 6.5, 13.4 Hz, 1H), 3.34 (dd, *J* = 4.3, 13.5 Hz, 1H), 2.43 (brs, 1H), 1.94-1.91 (m, 1H), 1.77-1.72 (m, 1H), 1.69-1.65 (m, 1H), 1.51-1.43 (m, 2H), 1.24 (s, 3H), 1.10 (q, *J* = 11.8 Hz, 1H), 1.02-0.94 (m, 1H), 0.92 (d, *J* = 6.5 Hz, 3H), 0.89-0.79 (m, 1H); ^{13}C NMR (125MHz, $CDCl_3$): δ (ppm) = 159.1 (C_q), 158.3 (C_q), 153.4 (CH), 113.4 (C_q), 75.2 (C_q), 72.9 (CH), 49.3 (CH_2), 49.3 (CH), 45.2 (CH_2), 34.1 (CH_2), 31.3 (CH), 26.4 (CH_2), 21.8 (CH_3), 21.1 (CH_3); HRMS (ESI): *m*/z $[M+H]^+$ calculated for $C_{14}H_{21}Cl_2N_3O_2$ 334.1011, found 334.1081.

(1R,2R,5R)-2-((R)-1-((2,5-Dichloropyrimidin-4-yl)amino)-2-hydroxypropan-2-yl)-5-methylcyclohexanol (**21b**): Prepared from **2b** and **18b**. White crystals (75%); m.p. 94–96 °C; $[\alpha]_D^{20}$ = +3.0 (c 0.1350, MeOH); ^{1}H NMR (500 MHz, $CDCl_3$): δ (ppm) = 8.00 (s, 1H), 6.25 (s, 1H), 4.71 (s, 1H), 3.84-3.80 (m, 1H), 3.77-3.69 (m, 1H), 3.54-3.48 (m, 1H), 2.38 (m, 1H), 1.94-1.92 (m, 1H), 1.87-1.82 (m, 1H), 1.75-1.72 (m, 1H), 1.61-1.56 (m, 1H), 1.53-1.45 (m, 1H), 1.20 (s, 3H), 1.14-1.06 (m, 2H), 0.99-0.88 (m, 1H), 0.94 (d, *J* = 6.4 Hz, 3H); ^{13}C NMR (125 MHz, $CDCl_3$): δ (ppm) = 159.2 (C_q), 158.3 (C_q), 153.3 (CH), 113.5 (C_q), 75.1 (C_q), 73.2 (CH), 52.4 (CH), 47.2 (CH_2), 45.6 (CH_2), 34.4 (CH_2), 31.5 (CH), 26.3 (CH_2), 25.0 (CH_3), 21.8 (CH_3); HRMS (ESI): *m*/z $[M+H]^+$ calculated for $C_{14}H_{21}Cl_2N_3O_2$ 334.1011, found 334.1082.

(S)-3-((2,5-Dichloropyrimidin-4-yl)amino)-2-((1R,2R,4R)-2-hydroxy-4-methylcyclohexyl)propane-1,2-diol (**21c**): Prepared from **4** and **18b**. White crystals (70%); m.p. 118–120 °C; $[\alpha]_D^{20}$ = +1.0 (c 0.1150, MeOH); ^{1}H NMR (500 MHz, $CDCl_3$): δ (ppm) = 8.00 (s, 1H), 6.46 (s, 1H), 5.15 (s, 1H), 3.90-3.86 (m, , 1H), 3.66-3.65 (m, 2H), 3.59-3.55 (m, 2H), 2.57 (t, *J* = 6.3 Hz, 1H), 2.49 (s, 1H), 1.97-1.95 (m, 1H), 1.82-1.69 (m, 3H), 1.52-1.43 (m, 1H), 1.15-1.06 (m, 2H), 0.97-0.88 (m, 1H), 0.94 (d, *J* = 6.6 Hz, 3H); ^{13}C NMR (125 MHz, $CDCl_3$): δ (ppm) = 159.2 (C_q), 158.2 (C_q), 153.3 (CH), 113.63 (C_q), 75.9 (C_q), 72.5 (CH), 66.2 (CH_2), 48.1 (CH), 45.4 (CH_2), 44.4 (CH_2), 34.1 (CH_2), 31.2 (CH), 25.5 (CH_2), 21.8 (CH_3); HRMS (ESI): *m*/z $[M+H]^+$ calculated for $C_{14}H_{21}Cl_2N_3O_3$ 350.0960, found 350.1026.

*(1R,2S,5R)-2-((R)-1-((2,5-Dichloropyrimidin-4-yl)amino)-3-hydroxypropan-2-yl)-5-methylcyclohexanol (***21d**): Prepared from **6** and **18b**. White crystals (84%); m.p. 138–140 °C; $[\alpha]_D^{20}$ = –45.0 (c 0.1150, MeOH); ^{1}H NMR (500 MHz, DMSO-d_6): δ (ppm) = 8.14 (s, 1H), 7.95 (s, 1H), 5.75 (s, 1H), 4.80-4.78 (m, 1H), 4.68 (d, *J* = 5.0 Hz, 1H), 3.52-3.48 (m, 3H), 3.28-3.27 (m, 1H), 2.19-2.18 (m, 1H), 1.87-1.85 (m, 1H), 1.58-1.54 (m, 2H), 1.38-1.32 (m, 2H), 1.14-1.06 (m, 1H), 0.90-0.75 (m, 2H), 0.86 (d, *J* = 6.4 Hz, 3H); ^{13}C NMR (125 MHz, DMSO-d_6): δ

(ppm) = 158.9 (C_q), 157.9 (C_q), 153.7 (CH), 113.4 (C_q), 69.8 (CH), 62.9 (CH_2), 45.6 (CH), 45.3 (CH_2), 41.8 (CH_2), 40.4 (CH), 34.9 (CH_2), 31.3 (CH), 26.8 (CH_2), 22.6 (CH_3); HRMS (ESI): *m*/z $[M+H]^+$ calculated for $C_{14}H_{21}Cl_2N_3O_2$ 334.1011, found 334.1084.

(3S,3aR,6R,7aR)-3-(((2,5-Dichloropyrimidin-4-yl)amino)methyl)-6-methyloctahydrobenzofuran-3-ol (**21e**): Prepared from **8a** and **18b**. Yellow crystals (88%); m.p. 90–91 °C; $[\alpha]_D^{20}$ = –6.0 (c 0.13, MeOH); ^1H NMR (500 MHz, $CDCl_3$): δ (ppm) = 8.05 (s, 1H), 5.94 (s, 1H), 4.05 (d, *J* = 10.4 Hz, 1H), 3.80-3.76 (m, 2H), 3.71 (dd, *J* = 5.1, 13.9 Hz, 1H), 3.57 (td, *J* = 3.9, 10.9 Hz, 1H), 2.48 (s, 1H), 2.16-2.13 (m, 1H), 1.80-1.69 (m, 2H), 1.55-1.44 (m, 1H), 1.42-1.33 (m, 1H), 1.30-1.25 (m, 1H), 1.08 (q, *J* = 11.4 Hz, 1H), 0.99 (d, *J* = 6.6 Hz, 3H), 0.97-0.91 (m, 1H); ^{13}C NMR (125 MHz, $CDCl_3$): δ (ppm) = 159.2 (C_q) , 158.3 (C_q), 153.8 (CH), 113.4 (C_q), 81.4 (CH), 79.8 (CH_2), 79.4 (CH_2), 53.1 (CH), 48.6 (CH_2), 39.9 (CH_2), 34.1 (CH_2), 30.9 (CH), 22.4 (CH_2), 21.9 (CH_3); HRMS (ESI): *m*/z $[M+H]^+$ calculated for $C_{14}H_{19}Cl_2N_3O_2$ 332.0854, found 332.0927.

(1S,2R,5R)-2-((S)-1-((2,5-Dichloropyrimidin-4-yl)amino)-2-hydroxypropan-2-yl)-5-methylcyclohexanol (**21f**): Prepared from **10** and **18b**. Yellow oil (84%); $[\alpha]_D^{20}$ = +17.0 (c 0.1375, MeOH); ^1H NMR (500 MHz, $CDCl_3$): δ (ppm) = 8.00 (s, 1H), 6.17 (brs, 1H), 4.46 (s, 1H), 3.62-3.48 (m, 3H), 2.08 (brs, 1H), 1.83-1.67 (m, 5H), 1.37-1.34 (m, 1H), 1.33 (s, 3H), 1.20-1.14 (m, 1H), 0.97-0.86 (m, 1H), 0.90 (d, *J* = 6.1 Hz, 3H); ^{13}C NMR (125 MHz, $CDCl_3$): δ (ppm) = 159.2 (Cq), 158.3 (Cq), 153.4 (CH), 113.4 (Cq), 74.0 (Cq), 68.3 (CH), 48.7 (CH_2), 46.9 (CH), 42.9 (CH_2), 34.5 (CH_2), 25.7 (CH), 24.7 (CH_3), 22.1 (CH_3), 20.5 (CH_2); HRMS (ESI): *m*/z $[M+H]^+$ calculated for $C_{14}H_{21}Cl_2N_3O_2$ 334.1011, found 334.1083.

(1S,2S,5R)-2-((S)-1-((2,5-Dichloropyrimidin-4-yl)amino)-3-hydroxypropan-2-yl)-5-methylcyclohexanol (**21g**): Prepared from **12** and **18b**. White crystals (85%); m.p. 184–186 °C; $[\alpha]_D^{20}$ = +6.0 (c 0.1425, MeOH); ^1H NMR (500 MHz, $CDCl_3$): δ (ppm) = 7.99 (s, 1H), 6.51 (s, 1H), 4.11 (s, 1H), 3.75-3.58 (m, 4H), 2.29 (brs, 1H), 1.88-1.85 (m, 2H), 1.79-1.75 (m, 2H), 1.60-1.46 (m, 3H), 1.26-1.17 (m, 1H), 0.98-0.84 (m, 1H), 0.98 (d, *J* = 6.3 Hz, 3H); ^{13}C NMR (125 MHz, $CDCl_3$): δ (ppm) = 159.2 (C_q) , 158.4 (C_q), 153.3 (CH), 113.4 (C_q), 67.7 (CH), 62.1 (CH_2), 42.7 (CH), 42.7 (CH_2), 41.1 (CH_2), 40.4 (CH), 34.8 (CH_2), 25.8 (CH), 23.9 (CH_2), 22.14 (CH_3); HRMS (ESI): m/z $[M+H]^+$ calculated for $C_{14}H_{21}Cl_2N_3O_2$ 334.1011, found 334.1077.

(3R,3aR,6R,7aS)-3-(((2,5-Dichloropyrimidin-4-yl)amino)methyl)-6-methyloctahydrobenzofuran-3-ol (**21h**): Prepared from **14** and **18b**. White crystals (80%); m.p. 185–186 °C; $[\alpha]_D^{20}$ = +3.0 (c 0.13, MeOH); ^1H NMR (500 MHz, $CDCl_3$): δ (ppm) = 8.04 (s, 1H), 5.95 (s, 1H), 4.42-4.41 (m, 1H), 4.03 (d, *J* = 10.0 Hz, 1H), 3.79-3.73 (m, 2H), 3.69-3.65 (dd, *J* = 5.0, 13.9 Hz, 1H), 2.39 (s, 1H), 2.08-2.05 (m, 1H), 1.86-1.81 (m, 1H), 1.69-1.60 (m, 3H), 1.21-1.11 (m, 2H), 0.90 (d, *J* = 6.58 Hz, 3H), 0.87-0.81 (m, 1H); ^{13}C NMR (125 MHz, $CDCl_3$): δ (ppm) = 159.0 (C_q), 158.4 (C_q), 153.8 (CH), 113.4 (C_q), 84.0 (C_q), 77.6 (CH), 76.6 (CH_2), 46.8 (CH), 44.4 (CH_2), 36.8 (CH_2), 32.8 (CH_2), 26.3 (CH), 24.2 (CH_2), 22.1 (CH_3); HRMS (ESI): *m*/z $[M+H]^+$ calculated for $C_{14}H_{19}Cl_2N_3O_2$ 332.0854, found 332.0926.

(1R,2R,5R)-2-((S)-1-((5-Amino-6-chloropyrimidin-4-yl)amino)-2-hydroxypropan-2-yl)-5-methylcyclohexanol (**23a**): Prepared from **2a** and **18c**. Yellow oil (82%); $[\alpha]_D^{20}$ = –10.0 (c 0.13, MeOH); ^1H NMR (500 MHz, $CDCl_3$): δ (ppm) = 8.00 (s, 1H), 5.72 (brs, 1H), 5.50-5.48 (m, 1H), 3.83 (td, *J* = 4.3, 10.4 Hz, 1H), 3.68 (dd, *J* = 6.6, 13.7 Hz, 1H), 3.41 (dd, *J* = 4.5, 13.8 Hz, 1H), 1.96-1.94 (m, 1H), 1.72-1.62 (m, 4H), 1.57-1.51 (m, 1H), 1.49-1.41 (m, 1H), 1.24 (s, 3H), 1.09 (q, *J* = 11.7 Hz, 1H), 1.02-0.93 (m, 1H), 0.91 (d, *J* = 5.7 Hz, 3H), 0.88-0.78 (m, 1H); ^{13}C

NMR (125 MHz, $CDCl_3$): δ (ppm) = 155.1 (C_q), 148.9 (CH), 142.7 (C_q), 122.1 (C_q), 76.5 (C_q), 72.5 (CH), 50.3 (CH_2), 49.0 (CH), 44.8 (CH_2), 34.2 (CH_2), 31.2 (CH), 26.6 (CH_2), 21.9 (CH_3), 21.1 (CH_3); HRMS (ESI): *m*/z $[M+H]^+$ calculated for $C_{14}H_{24}ClN_4O_2$ 315.1509, found 315.1578.

(1R,2R,5R)-2-((R)-1-((5-Amino-6-chloropyrimidin-4-yl)amino)-2-hydroxypropan-2-yl)-5-methylcyclohexanol (**23b**): Prepared from **2b** and **18c**. White crystals (80%); m.p. 213–214 °C; $[\alpha]_D^{20}$ = –20.0 (c 0.12, MeOH); ^{1}H NMR (500 MHz, DMSO-*d6*): δ (ppm) = 7.69 (s, 1H), 6.58-6.56 (m, 1H), 5.41 (d, *J* = 4.1 Hz, 1H), 5.30 (s, 1H), 5.13 (s, 2H), 3.88 (dd, *J* = 7.3, 13.6 Hz, 1H), 3.61-3.55 (m, 1H), 3.41 (dd, *J* = 4.0, 13.5 Hz, 1H), 1.82-1.77 (m, 2H), 1.63-1.60 (m, 1H), 1.40-1.32 (m, 2H), 1.03 (s, 3H), 0.93-0.89 (m, 1H), 0.86 (d, *J* = 6.3 Hz, 3H), 0.83-0.75 (m, 1H); ^{13}C NMR (125 MHz, DMSO-*d6*): δ (ppm) = 153.3 (C_q), 145.9 (CH), 137.4 (C_q), 124.0 (C_q), 75.3 (C_q), 71.6 (CH), 52.6 (CH), 48.6 (CH_2), 45.8 (CH_2), 34.9 (CH_2), 31.4 (CH), 26.3 (CH_2), 23.9 (CH_3), 22.5 (CH_3); HRMS (ESI): *m*/z $[M+H]^+$ calculated for $C_{14}H_{24}ClN_4O_2$ 315,1509, found 315.1579.

(S)-3-((5-Amino-6-chloropyrimidin-4-yl)amino)-2-((1R,2R,4R)-2-hydroxy-4-methylcyclohexyl)propane-1,2-diol (**23c**): Prepared from **4** and **18c**. Yellow crystals (79%); m.p. 160–162 °C; $[\alpha]_D^{20}$ = –6.0 (c 0.1325, MeOH); ^{1}H NMR (500 MHz, $CDCl_3$): δ (ppm) = 8.02 (s, 1H), 5.64 (s, 1H), 5.16 (s, 1H), 3.87-3.82 (m, 1H), 3.75-3.65 (m, 2H), 3.61-3.51 (m, 4H), 3.03-2.98 (m, 2H), 1.97-1.95 (m, 1H), 1.88-1.84 (m, 1H), 1.77-1.68 (m, 2H), 1.50-1.42 (m, 1H), 1.15-1.04 (m, 2H), 0.93-0.88 (m, 1H), 0.92 (d, *J* = 6.4 Hz, 3H); ^{13}C NMR (125 MHz, $CDCl_3$): δ (ppm) = 155.2 (C_q), 149.1 (CH), 76.5 (C_q), 72.4 (CH), 66.2 (CH_2), 48.6 (CH), 45.4 (CH_2), 44.9 (CH_2), 34.2 (CH_2), 31.3 (CH), 25.5 (CH_2), 21.8 (CH_3); HRMS (ESI): *m*/z $[M+H]^+$ calculated for $C_{14}H_{24}ClN_4O_3$ 331.1458, found 331.1526.

(1R,2S,5R)-2-((R)-1-((5-Amino-6-chloropyrimidin-4-yl)amino)-3-hydroxypropan-2-yl)-5-methylcyclohexanol (**23d**): Prepared from **6** and **18c**. Yellow crystals (88%); m.p. 187–189 °C; $[\alpha]_D^{20}$ = –32.0 (c 0.1375, MeOH); ^{1}H NMR (500 MHz, DMSO-*d6*): δ (ppm) = 7.71 (s, 1H), 6.73-6.71 (m, 1H), 5.01 (s, 2H), 4.56 (d, *J* = 5.1 Hz, 1H), 4.47 (t, *J* = 5.1 Hz, 1H), 3.53-3.35 (m, 4H), 3.27-3.23 (m, 1H), 2.25-2.18 (m, 1H), 1.88-1.85 (m, 1H), 1.67-1.58 (m, 2H), 1.44-1.37 (m, 2H), 1.05-1.02 (m, 1H), 0.91-0.84 (m, 1H), 0.87 (d, *J* = 6.4 Hz, 3H), 0.80-0.77 (m, 1H); ^{13}C NMR (125 MHz, DMSO-*d6*): δ (ppm) = 152.7 (C_q), 146.1 (CH), 137.0 (C_q), 123.9 (C_q), 70.0 (CH), 61.7 (CH_2), 45.5 (CH_2), 44.6 (CH), 40.4 (CH_2), 40.3 (CH), 35.0 (CH_2), 31.5 (CH), 26.0 (CH_2), 22.7 (CH_3); HRMS (ESI): *m*/z $[M+H]^+$ calculated for $C_{14}H_{24}ClN_4O_2$ 315.1509, found 315.1581.

(3S,3aR,6R,7aR)-3-(((5-Amino-6-chloropyrimidin-4-yl)amino)methyl)-6-methyloctahydrobenzofuran-3-ol (**23e**): Prepared from **8a** and **18c**. White crystals (88%); m.p. 171–172 °C; $[\alpha]_D^{20}$ = –30.0 (c 0.1425, MeOH); ^{1}H NMR (500 MHz, $CDCl_3$): δ (ppm) = 8.03 (s, 1H), 5.42-5.40 (m, 1H), 4.15 (brs, 1H), 4.06 (d, *J* = 9.9 Hz, 1H), 3.77 (d, *J* = 10.1 Hz, 1H), 3.75-3.65 (m, 2H), 3.57 (td, *J* = 3.7, 10.8 Hz, 1H), 3.48 (s, 2H), 2.16-2.08 (m, 1H), 1.77-1.74 (m, 1H), 1.70-1.66 (m, 1H), 1.55-1.46 (m, 1H), 1.45-1.37 (m, 1H), 1.25-1.19 (m, 1H), 1.05 (q, *J* = 11.2 Hz, 1H), 0.98 (d, *J* = 6.5 Hz, 3H), 0.95-0.89 (m, 1H); ^{13}C NMR (125 MHz, $CDCl_3$): δ (ppm) = 155.6 (C_q), 149.1 (CH), 143.7 (C_q), 122.1 (C_q), 81.5 (CH), 80.2 (C_q), 79.7 (CH_2), 53.5 (CH), 50.2 (CH_2), 40.0 (CH_2), 34.2 (CH_2), 31.0 (CH), 22.6 (CH_2), 22.0 (CH_3); HRMS (ESI): *m*/z $[M+H]^+$ calculated for $C_{14}H_{22}ClN_4O_2$ 313.1353, found 313.1424.

(1S,2R,5R)-2-((S)-1-((5-Amino-6-chloropyrimidin-4-yl)amino)-2-hydroxypropan-2-yl)-5-methylcyclohexanol (**23f**): Prepared from **10** and **18c**. Brown oil (83%); $[\alpha]_D^{20}$ = −7.0 (c 0.1825, MeOH); ^{1}H NMR (500 MHz, $CDCl_3$): δ (ppm) = 8.00 (s, 1H), 5.63 (brs, 1H), 4.56 (brs, 1H), 4.52 (brs, 1H), 4.44 (s, 1H), 3.69 (dd, *J* = 4.0, 13.7 Hz, 1H), 3.57 (s, 2H), 3.49-3.41 (m, 1H), 3.02 (brs, 1H), 1.86-1.67 (m, 5H), 1.32-1.30 (m, 4H), 1.15-1.08 (m, 1H), 0.97-0.84 (m, 1H), 0.88 (d, *J* = 6.3 Hz, 3H); ^{13}C NMR (125 MHz, $CDCl_3$): δ (ppm) = 155.4 (C_q) , 149.0 (CH), 142.7 (C_q), 122.1 (C_q), 75.0 (C_q), 68.0 (CH), 49.8 (CH_2), 47.6 (CH), 42.6 (CH_2), 34.7 (CH_2), 25.7 (CH), 24.4 (CH_3), 22.1 (CH_3), 20.4 (CH_2); HRMS (ESI): *m*/z [M+H]$^+$ calculated for $C_{14}H_{24}ClN_4O_2$ 315.1509, found 315.1579.

(1S,2S,5R)-2-((S)-1-((5-Amino-6-chloropyrimidin-4-yl)amino)-3-hydroxypropan-2-yl)-5-methylcyclohexanol (**23g**): Prepared from **12** and **18c**. Yellow crystals (90%); m.p. 182−183 °C; $[\alpha]_D^{20}$ = +19.0 (c 0.1150, MeOH); ^{1}H NMR (500 MHz, DMSO-d_6): δ (ppm) = 7.70 (s, 1H), 6.71 (t, *J* = 5.1 Hz, 1H), 4.97 (s, 2H), 4.69 (t, *J* = 5.0 Hz, 1H), 4.56 (d, *J* = 3.4 Hz, 1H), 3.91 (s, 1H), 3.52-3.38 (m, 4H), 1.74-1.63 (m, 4H), 1.49-1.39 (m, 3H), 1.04-0.99 (m, 1H), 0.86-0.84 (m, 1H), 0.81 (d, *J* = 6.4 Hz, 3H); ^{13}C NMR (125 MHz, DMSO-d_6): δ (ppm) = 152.9 (C_q), 146.1 (CH), 137.2 (C_q), 123.9 (C_q), 67.1 (CH), 59.9 (CH_2), 43.0 (CH_2), 43.0 (CH), 40.7 (CH_2), 40.6 (CH), 35.4 (CH_2), 25.9 (CH), 23.2 (CH_2), 22.9 (CH_3); HRMS (ESI): m/z [M+H]$^+$ calculated for $C_{14}H_{24}ClN_4O_2$ 315.1509, found 315.1575.

(3R,3aR,6R,7aS)-3-(((5-Amino-6-chloropyrimidin-4-yl)amino)methyl)-6-methyloctahydrobenzofuran-3-ol (**23h**): Prepared from **14** and **18c**. Brown oil (86%); $[\alpha]_D^{20}$ = −6.0 (c 0.1250, MeOH); ^{1}H NMR (500 MHz, $CDCl_3$): δ (ppm) = 8.04 (s, 1H), 5.34-5.29 (m, 1H), 4.43 (m, 1H), 4.01 (d, *J* = 9.5 Hz, 1H), 3.75-3.71 (m, 3H), 3.50 (brs, 2H), 2.08-2.04 (m, 1H), 1.87-1.81 (m, 1H), 1.67-1.58 (m, 3H), 1.20-1.11 (m, 2H), 0.89 (d, *J* = 6.7 Hz, 3H), 0.86-0.81 (m, 1H); ^{13}C NMR (125 MHz, $CDCl_3$): δ (ppm) = 155.4 (C_q), 149.2 (CH), 143.5 (C_q), 122.1 (C_q), 84.4 (C_q), 77.6 (CH), 76.7 (CH_2), 47.1 (CH), 45.3 (CH_2), 36.8 (CH_2), 32.9 (CH_2), 26.3 (CH), 24.4 (CH_2), 22.1 (CH_3); HRMS (ESI): *m*/z [M+H]$^+$ calculated for $C_{14}H_{22}ClN_4O_2$ 313.1353, found 313.1422.

1.5.5. General procedure for the preparation of N^2-(p-trifluoromethyl)aniline substituted pyrimidines (20a–i) and (22a–h)

A mixture of pyrimidines **19a–i** or **21a–h** (0.16 mmol) and 4-trifluoromethylaniline (0.24 mmol) in EtOH (200 µL) was heated in microwave reactor at 150 °C, 200 W, 19 bar for 80 min. The formed precipitate was filtered off and washed with CH_2Cl_2 to afford the desired product in pure form without further purification.

(1R,2R,5R)-2-((S)-1-((5-Fluoro-2-((4-(trifluoromethyl)phenyl)amino)pyrimidin-4-yl)amino)-2-hydroxypropan-2-yl)-5-methylcyclohexan-1-ol hydrochloride (**20a**): Prepared from **19a**. White crystals (72%); m.p. 154–156 °C; $[\alpha]_D^{20}$ = +43.0 (c 0.1150, MeOH); ^{1}H NMR (500 MHz, DMSO-d_6): δ (ppm) = 9.82 (s, 1H), 8.04 (d, *J* = 4.1 Hz, 1H), 7.88 (d, *J* = 8.4 Hz, 2H), 7.60 (d, *J* = 8.7 Hz, 2H), 7.54 (brs, 1H), 3.66-3.61 (m, 1H), 3.54-3.45 (m, 2H), 1.84-1.82 (m, 1H), 1.72-1.69 (m, 1H), 1.53-1.51 (m, 1H), 1.41-1.36 (m, 2H), 1.12 (s, 3H), 1.00-0.88 (m, 2H), 0.85 (d, *J* = 6.4 Hz, 3H), 0.80-0.73 (m, 1H); ^{13}C NMR (125 MHz, DMSO-d_6): δ (ppm) = 153.8 (C_q), 143.9 (C_q), 141.9 (C_q), 140.0 (C_q), 135,4 (CH), 126.1 (CH), 119.5 (CH), 75.8 (Cq), 71.4 (CH), 49.6 (CH), 49.2 (CH_2), 45.2 (CH_2), 34.5 (CH_2), 31.1 (CH), 26.3 (CH_2), 22.4 (CH_3), 21.8 (CH_3); HRMS (ESI): *m*/z [M+H]$^+$ calculated for $C_{21}H_{27}F_4N_4O_2$ 443.1992, found 443.2061.

(1R,2R,5R)-2-((R)-1-((5-Fluoro-2-((4-(trifluoromethyl)phenyl)amino)pyrimidin-4-yl)amino)-2-hydroxypropan-2-yl)-5-methylcyclohexan-1-ol hydrochloride (**20b**): Prepared from **19b**. Yellow crystals (87%); m.p. 154–156 °C; $[\alpha]_D^{20}$ = +16.0 (c 0.14, MeOH); ^{1}H NMR (500 MHz, DMSO-*d6*): δ (ppm)= 10.38 (s, 1H), 8.29 (brs, 1H), 8.13 (d, *J* = 4.9 Hz, 1H), 7.87 (d, *J* = 8.5 Hz, 2H), 7.64 (d, *J* = 8.5 Hz, 2H), 3.92-3.88 (m, 1H), 3.58-3.49 (m, 2H), 1.86-1.84 (m, 1H), 1.79-1.76 (m, 1H), 1.61-1.59 (m, 1H), 1.41-1.33 (m, 2H), 1.03 (s, 3H), 0.97-0.90 (m, 1H), 0.87 (d, *J* = 6.4 Hz, 3H), 0.85-0.78 (m, 1H); ^{13}C NMR (125 MHz, DMSO-*d6*): δ (ppm) = 154.5 (C_q), 152.0 (C_q), 142.9 (C_q), 141.3 (C_q), 139.3 (C_q), 130,0 (CH), 126.3 (CH), 120.1 (CH), 75.3 (C_q), 71.4 (CH), 52.8 (CH), 48.8 (CH_2), 45.6 (CH_2), 34.8 (CH_2), 31.4 (CH), 26.1 (CH_2), 23.5 (CH_3), 22.4 (CH_3); HRMS (ESI): *m*/z $[M+H]^+$ calculated for $C_{21}H_{27}F_4N_4O_2$ 443.1992, found 443.2062.

(S)-3-((5-Fluoro-2-((4-(trifluoromethyl)phenyl)amino)pyrimidin-4-yl)amino)-2-((1R,2R,4R)-2-hydroxy-4-methylcyclohexyl)propane-1,2-diol hydrochloride (**20c**): Prepared from **19c**. White crystals (84%); m.p. 158–160 °C; $[\alpha]_D^{20}$ = +18.0 (c 0.1075, MeOH); ^{1}H NMR (500 MHz, DMSO-*d6*): δ (ppm) = 10.07 (1H, s), 8.07 (d, *J* = 4.4 Hz, 1H), 7.92 (brs, 1H), 7.87 (d, *J* = 8.5 Hz, 2H), 7.62 (d, *J* = 8.5 Hz, 2H), 3.78-3.74 (m, 1H), 3.69-3.64 (m, 1H), 3.62-3.58 (m, 1H), 3.41-3.54 (2H, m), 1.88-1.75 (m, 2H), 1.65-1.54 (m, 2H), 1.38-1.36 (m, 1H), 1.09-1.00 (m, 1H), 0.97-0.90 (m, 1H), 0.87 (d, *J* = 6.4 Hz, 3H), 0.83-0.75; ^{13}C NMR (125 MHz, DMSO-d6): δ (ppm) = 154.0 (C_q), 143.4 (C_q), 141.8 (C_q), 139.8 (C_q), 132.7 (CH), 126.3 (CH), 119.8 (CH), 77.2 (CH_2), 70.9 (CH), 65.2 (CH_2), 48.3 (CH), 45.7 (CH_2), 45.2 (CH_2), 34.8 (CH_2), 31.2 (CH), 25.8 (CH_2), 22.5 (CH_3); HRMS (ESI): *m*/z $[M+H]^+$ calculated for $C_{21}H_{27}F_4N_4O_3$ 459.1941, found 459.2004.

(1R,2S,5R)-2-((R)-1-((5-Fluoro-2-((4-(trifluoromethyl)phenyl)amino)pyrimidin-4-yl)amino)-3-hydroxypropan-2-yl)-5-methylcyclohexan-1-ol hydrochloride (**20d**): Prepared from **19d**. Yellow crystals (94%); m.p. 179–181 °C; $[\alpha]_D^{20}$ = –23.0 (c 0.1375, MeOH); ^{1}H NMR (500 MHz, DMSO-*d6*): δ (ppm) = 9.46 (s, 1H), 7.95 (d, *J* = 8.6 Hz, 2H), 7.90 (d, *J* = 3.6 Hz, 1H), 7.54 (d, *J* = 8.8 Hz, 2H), 7.36-7.34 (m, 1H), 4.62-4.60 (m, 1H), 4.54 (d, *J* = 5.6 Hz, 1H), 3.54-3.49 (m, 3H), 3.40-3.35 (m, 2H), 2.22-2.20 (m, 1H), 1.87-1.84 (m, 1H), 1.61-1.57 (m, 2H), 1.40-1.33 (m, 2H), 1.16-1.13 (m, 1H), 0.91-0.89 (m, 1H), 0.87 (d, *J* = 6.5 Hz, 3H), 0.91-0.78 (m, 1H); ^{13}C NMR (125 MHz, DMSO-*d6*): δ (ppm) = 155.8 (C_q), 152.7 (C_q), 145.4 (C_q), 142.8 (Cq), 140.8 (C_q), 138.6 (CH), 126.0 (CH), 120.4 (Cq), 118.0 (CH), 70.0 (CH), 62.4 (CH_2), 45.6 (CH_2), 45.3 (CH), 40.9 (CH), 40.6 (CH_2), 34.9 (CH_2), 31.5 (CH), 27.0 (CH_2), 22.7 (CH_3); HRMS (ESI): *m*/z $[M+H]^+$ calculated for $C_{21}H_{27}F_4N_4O_2$ 443.1992, found 443.2061.

(3S,3aR,6R,7aR)-3-(((5-Fluoro-2-((4-(trifluoromethyl)phenyl)amino)pyrimidin-4-yl)amino)methyl)-6-methyloctahydrobenzofuran-3-ol hydrochloride (**20e**): Prepared from **19e**. White crystals (87%); m.p. 150–152 °C; $[\alpha]_D^{20}$ = –33.0 (c 0.1350, MeOH); ^{1}H NMR (500 MHz, DMSO-*d6*): δ (ppm) = 10.58 (s, 1H), 8.71 (brs, 1H), 8.19 (d, *J* = 4.9 Hz, 1H), 7.84 (d, *J* = 8.4 Hz, 2H), 7.67 (d, *J* = 8.7 Hz, 2H), 3.98 (d, *J* = 9.3 Hz, 1H), 3.72 (dd, *J* = 6.6, 13.6 Hz, 1H), 3.55-3.50 (m, 2H), 3.46-3.41 (m, 1H), 1.95-1.93 (m, 1H), 1.59 (d, *J* = 11.1 Hz, 2H), 1.43-1.33 (m, 1H), 1.26-1.19 (m, 1H), 1.13-1.04 (m, 1H), 0.89 (d, *J* = 6.48 Hz, 3H), 0.85-0.72 (m, 2H); ^{13}C NMR (125 MHz, DMSO-*d6*): δ (ppm) = 154.6 (C_q), 151.8 (C_q) 142.6 (C_q), 141.2 (C_q), 139.2 (C_q), 131,0 (CH), 126.4 (CH), 120.3 (CH), 80.6 (CH), 79.1 (CH_2), 52.1 (CH), 46.7 (CH_2), 40.4 (CH_2), 34.4 (CH_2), 30.9 (CH), 22.4 (CH_3), 22.3 (CH_2); HRMS (ESI): *m*/z $[M+H]^+$ calculated for $C_{21}H_{25}F_4N_4O_2$ 441.1835, found 441.1906.

(3R,3aR,6R,7aR)-3-(((5-Fluoro-2-((4-(trifluoromethyl)phenyl)amino)pyrimidin-4-yl)amino)methyl)-6-methyloctahydrobenzofuran-3-ol hydrochloride (**20f**): Prepared from **19f**. Yellow crystals (90%); m.p. 156–158 °C; $[\alpha]_D^{20}$ = –40.0 (c 0.1450, MeOH); ^{1}H NMR (500 MHz, DMSO-*d*$_6$): δ (ppm) = 10.38 (s, 1H), 8.27 (brs, 1H), 8.15 (d, *J* = 4.6 Hz, 1H), 7.83 (d, *J* = 8.5 Hz, 2H), 7.61 (d, *J* = 8.5 Hz, 2H), 3.79-3.73 (m, 2H), 3.57 (d, *J* = 9.17 Hz, 1H), 3.44 (dd, *J* = 4.6, 13.6 Hz, 1H), 3.27-3.21 (m, 1H), 1.94-1.92 (m, 1H), 1.83-1.80 (m, 1H), 1.71-169 (m, 1H), 1.55-1.50 (m, 1H), 1.38-1.36 (m, 1H), 1.19-1.11 (m, 1H), 1.03 (q, *J* = 11.2 Hz, 1H), 0.94 (d, *J* = 6.5 Hz, 3H), 0.91-0.83 (m, 1H); ^{13}C NMR (125 MHz, DMSO-*d*$_6$): δ (ppm) = 154.5 (C$_q$), 143.0 (C$_q$), 141.4 (C$_q$), 139.4 (C$_q$), 131,9 (CH), 126.2 (CH), 120.4 (CH), 81.3 (CH), 79.3 (CH$_2$), 76.9 (CH$_2$), 55.9 (CH), 45.9 (CH$_2$), 34.7 (CH$_2$), 31.0 (CH), 22.7 (CH$_2$), 22.3 (CH$_3$); HRMS (ESI): *m*/z [M+H]$^+$ calculated for $C_{21}H_{25}F_4N_4O_2$ 441.1835, found 441.1908.

(1S,2R,5R)-2-((S)-1-((5-Fluoro-2-((4-(trifluoromethyl)phenyl)amino)pyrimidin-4-yl)amino)-2-hydroxypropan-2-yl)-5-methylcyclohexan-1-ol hydrochloride (**20g**): Prepared from **19g**. White crystals (80%); m.p. 166–168 °C; $[\alpha]_D^{20}$ = +18.0 (c 0.1350, MeOH); ^{1}H NMR (500 MHz, DMSO-*d*$_6$): δ (ppm) = 10.26 (brs, 1H), 8.44 (brs, 1H), 8.10 (d, *J* = 4.6 Hz, 1H), 7.87 (d, *J* = 8.3 Hz, 2H), 7.64 (d, *J* = 8.6 Hz, 2H), 4.18 (s, 1H), 3.54 (d, *J* = 5.7 Hz, 2H), 1.79-1.65 (m, 3H), 1.60-1.57 (m, 2H), 1.34-1.31 (m, 1H), 1.17 (s, 3H), 1.04-0.97 (m, 1H), 0.90-0.84 (m, 1H), 0.82 (d, *J* = 6.4 Hz, 3H); ^{13}C NMR (125 MHz, DMSO-*d*$_6$): δ (ppm) = 126.4 (CH), 119.8 (CH), 73.9 (C$_q$), 65.6 (CH), 49.0 (CH), 48.5 (CH$_2$), 43.2 (CH$_2$), 35.3 (CH$_2$), 25.8 (CH), 25.4 (CH$_3$), 22.8 (CH$_3$), 21.2 (CH$_2$); HRMS (ESI): *m*/z [M+H]$^+$ calculated for $C_{21}H_{27}F_4N_4O_2$ 443.1992, found 443.2062.

(1S,2S,5R)-2-((S)-1-((5-Fluoro-2-((4-(trifluoromethyl)phenyl)amino)pyrimidin-4-yl)amino)-3-hydroxypropan-2-yl)-5-methylcyclohexan-1-ol hydrochloride (**20h**): Prepared from **20h**. Yellow crystals (96%); m.p. 145–147 °C; $[\alpha]_D^{20}$ = +15.0 (c 0.13, MeOH); ^{1}H NMR (500 MHz, DMSO-*d*$_6$): δ (ppm) = 10.26 (s, 1H), 8.44 (s, 1H), 8.08 (d, *J* = 4.3 Hz, 1H), 7.89 (d, *J* = 8.4 Hz, 2H), 7.65 (d, *J* = 8.2 Hz, 2H), 3.83 (s, 1H), 3.57-3.52 (m, 2H), 3.46-3.43 (m, 1H), 3.17 (s, 1H), 1.86-1.85 (m, 1H), 1.76-1.62 (m, 3H), 1.49-1.39 (m, 3H), 1.05-0.99 (m, 1H), 0.88-0.83 (m, 1H), 0.81 (d, *J* = 6.3 Hz, 3H); ^{13}C NMR (125 MHz, DMSO-*d*$_6$): δ (ppm) = 153.9 (C$_q$), 143.2 (C$_q$), 141.5 (C$_q$), 139.5 (C$_q$), 126.3 (CH), 119.7 (CH), 67.2 (CH), 60.7 (CH$_2$), 43.0 (CH$_2$), 43.0 (CH), 41.8 (CH$_2$), 41.0 (CH), 35.4 (CH$_2$), 25.9 (CH), 23.2 (CH$_2$), 22.8 (CH$_3$); HRMS (ESI): m/z [M+H]$^+$ calculated for $C_{21}H_{27}F_4N_4O_2$ 443.1992, found 443.2058.

(3R,3aR,6R,7aS)-3-(((5-Fluoro-2-((4-(trifluoromethyl)phenyl)amino)pyrimidin-4-yl)amino)methyl)-6-methyloctahydrobenzofuran-3-ol hydrochloride (**20i**): Prepared from **19i**. Yellow crystals (87%); m.p. 238–240 °C; $[\alpha]_D^{20}$ = –61.0 (c 0.1250, MeOH); ^{1}H NMR (500 MHz, DMSO-*d*$_6$): δ (ppm) = 10.22 (s, 1H), 8.11 (d, *J* = 4.6 Hz, 1H), 8.05 (brs, 1H), 7.85 (d, *J* = 8.4 Hz, 2H), 7.63 (d, *J* = 8.7 Hz, 2H), 4.28-4.27 (m, 1H), 3.85 (d, *J* = 9.2 Hz, 1H), 3.81-3.77 (m, 1H), 3.54-3.46 (m, 2H), 1.87-1.84 (m, 1H), 1.79-1.75 (m, 1H), 1.65-1.62 (m, 1H), 1.55-1.52 (m, 1H), 1.45-1.43 (m, 1H), 1.12-0.94 (m, 2H), 0.83 (d, *J* = 6.5 Hz, 3H), 0.83-0.77 (m, 1H); ^{13}C NMR (125 MHz, DMSO-*d*$_6$): δ (ppm) = 154.0 (C$_q$), 143.3 (C$_q$), 139.7 (C$_q$), 133.4 (CH), 126.2 (CH), 120.0 (CH), 83.0 (C$_q$), 76.9 (CH), 75.8 (CH$_2$), 46.9 (CH), 44.9 (CH$_2$), 37.1 (CH$_2$), 33.1 (CH$_2$), 26.5 (CH), 24.2 (CH$_2$), 22.6 (CH$_3$); HRMS (ESI): *m*/z [M+H]$^+$ calculated for $C_{21}H_{25}F_4N_4O_2$ 441.1835, found 441.1909.

(1R,2R,5R)-2-((S)-1-((5-Chloro-2-((4-(trifluoromethyl)phenyl)amino)pyrimidin-4-yl)amino)-2-hydroxypropan-2-yl)-5-methylcyclohexan-1-ol hydrochloride (**22a**): Prepared from **21a**. White crystals (80%); m.p. 167–169 °C; $[\alpha]_D^{20}$ = +54.0 (c 0.1225, MeOH); ^{1}H NMR

(500 MHz, $CDCl_3$): δ (ppm) = 10.21 (s, 1H), 8.17 (s, 1H), 7.89 (d, *J* = 8.5 Hz, 2H), 7.64 (d, *J* = 8.6 Hz, 2H), 7.12 (s, 1H), 3.64 (td, *J* = 4.1, 10.3 Hz, 1H), 3.59-3.45 (m, 2H), 1.85 (d, *J* = 12.1 Hz, 1H), 1.69-1.66 (m, 1H), 1.54-1.52 (m, 1H), 1.38-1.31 (m, 2H), 1.13 (s, 3H), 0.98-0.91 (m, 2H), 0.85 (d, *J* = 6.5 Hz, 3H), 0.80-0.72 (m, 1H); ^{13}C NMR (125 MHz, $CDCl_3$): δ (ppm) = 158.6 (C_q) ,148,5 (CH) 143.4 (C_q), 126.2 (CH), 123.9 (C_q) 120.0 (CH), 105.3 (C_q), 75.4 (C_q), 71.3 (CH), 49.9 (CH), 49.6 (CH_2), 45.2 (CH_2), 34.5 (CH_2), 31.1 (CH), 26.1 (CH_2), 22.4 (CH_3), 22.0 (CH_3); HRMS (ESI): *m*/z $[M+H]^+$ calculated for $C_{21}H_{27}ClF_3N_4O_2$ 459.1696, found 459.1770.

((1R,2R,5R)-2-((R)-1-((5-Chloro-2-((4-(trifluoromethyl)phenyl)amino)pyrimidin-4-yl)amino)-2-hydroxypropan-2-yl)-5-methylcyclohexan-1-ol hydrochloride (**22b**): Prepared from **21b**. White crystals (82%); m.p. 82–84 °C; $[\alpha]_D^{20}$ = +12.0 (c 0.1325, MeOH); ^{1}H NMR (500 MHz, DMSO-*d*$_6$): δ (ppm) = 9.95 (s, 1H), 8.10 (s, 1H), 7.90 (d, *J* =8.7 Hz, 2H), 7.62 (d, *J* = 8.4 Hz, 2H), 7.20 (brs, 1H), 3.95 (dd, *J* = 7.3, 13.4 Hz, 1H), 3.55 (td, *J* = 3.9, 10.3 Hz, 1H), 3.47 (dd, *J* = 4.2, 13.3 Hz, 1H), 1.87-1.79 (m, 2H), 1.63-1.60 (m, 1H), 1.42-1.34 (m, 2H), 1.00 (s, 3H), 0.99-0.90 (m, 1H), 0.88 (d, *J* = 6.4 Hz, 3H), 0.86-0.78 (m, 1H); ^{13}C NMR (125 MHz, DMSO-*d*$_6$): δ (ppm) = 158.6 (C_q), 149,5 (CH), 143.7 (C_q), 126.2 (CH), 123,9 (C_q), 119.8 (CH), 105.3 (C_q), 74.9 (C_q), 71.3 (CH), 52.7 (CH), 49.3 (CH_2), 45.7 (CH_2), 34.8 (CH_2), 31.4 (CH), 26.0 (CH_2), 23.2 (CH_3), 22.4 (CH_3); HRMS (ESI): *m*/z $[M+H]^+$ calculated for $C_{21}H_{27}ClF_3N_4O_2$ 459.1696, found 459.1770.

(S)-3-((5-Chloro-2-((4-(trifluoromethyl)phenyl)amino)pyrimidin-4-yl)amino)-2-((1R,2R,4R)-2-hydroxy-4-methylcyclohexyl)propane-1,2-diol hydrochloride (**22c**): Prepared from **21c**. Yellow crystals (90%); m.p. 163–165 °C; $[\alpha]_D^{20}$ = –4.0 (c 0.1300, MeOH); ^{1}H NMR (500 MHz, DMSO-*d*$_6$): δ (ppm) = 10.10 (1H, s), 8.12 (s, 1H), 7.90 (d, *J* = 8.5 Hz, 2H), 7.63 (d, *J* = 8.7 Hz, 2H), 7.37 (s, 1H), 3.80-3.76 (m, 1H), 3.68-3.59 (m, 2H), 3.41 (s, 2H), 1.89-1.76 (m, 2H), 1.59-1.54 (m, 2H), 1.38-1.33 (m, 1H), 1.09-1.00 (m, 1H), 0.97-0.90 (m, 1H), 0.87 (d, *J* = 6.6 Hz, 3H), 0.83-0.75 (m, 1H); ^{13}C NMR (125 MHz, DMSO-*d*$_6$): δ (ppm) = 158.5 (C_q), 148,7 (CH), 143.6 (C_q), 126.2 (CH), 119.8 (CH), 105.4 (C_q), 76.5 (C_q), 70.9 (CH), 65.6 (CH_2), 48.6 (CH), 45.9 (CH_2), 45,7 (CH_2), 34.7 (CH_2), 31.3 (CH), 25.7 (CH_2), 22.5 (CH_3); HRMS (ESI): m/z $[M+H]^+$ calculated for $C_{21}H_{27}ClF_3N_4O_3$ 475.1645, found 475.1712.

(1R,2S,5R)-2-((R)-1-((5-Chloro-2-((4-(trifluoromethyl)phenyl)amino)pyrimidin-4-yl)amino)-3-hydroxypropan-2-yl)-5-methylcyclohexan-1-ol hydrochloride (**22d**): Prepared from **21d**. Yellow crystals (98%); m.p. 173–175 °C; $[\alpha]_D^{20}$ = –29.0 (c 0.1250, MeOH); ^{1}H NMR (500 MHz, DMSO-*d*$_6$): δ (ppm) = 10.48 (brs, 1H), 8.29 (brs, 1H), 8.17 (s, 1H), 7.89 (d, *J* = 8.4 Hz, 2H), 7.66 (d, *J* = 8.5 Hz, 2H), 3.65-3.61 (m, 1H), 3.53 (d, *J* = 6.7 Hz, 2H), 3.46-3.41 (m, 1H), 3.28 (td, *J* = 3.9, 10.3 Hz, 1H), 2.22-2.19 (m, 1H), 1.86-1.84 (m, 1H), 1.57-1.52 (m, 2H), 1.36-1.31 (m, 2H), 1.18-1.10 (m, 1H), 0.90-0.88 (m, 1H), 0.86 (d, *J* = 6.4 Hz, 3H), 0.82-0.74 (m, 1H); ^{13}C NMR (125MHz, DMSO-*d*$_6$): δ (ppm) = 158.2 (C_q), 153.9 (C_q), 145,6 (CH), 142.8 (C_q), 126.3 (CH), 123.8 (C_q), 120.3 (CH), 105.3 (C_q), 69.9 (CH), 62.4 (CH_2), 45.5 (CH), 45.5 (CH_2), 42.4 (CH_2), 40.8 (CH), 34.9 (CH_2), 31.4 (CH), 27.2 (CH_2), 22.6 (CH_3); HRMS (ESI): *m*/z $[M+H]^+$ calculated for $C_{21}H_{27}ClF_3N_4O_2$ 459.1696, found 459.1769.

(3S,3aR,6R,7aR)-3-(((5-Chloro-2-((4-(trifluoromethyl)phenyl)amino)pyrimidin-4-yl)amino)methyl)-6-methyloctahydrobenzofuran-3-ol hydrochloride (**22e**): Prepared from **21e**. White crystals (85%); m.p. 188–190 °C; $[\alpha]_D^{20}$ = –23.0 (c 0.1350, MeOH); ^{1}H NMR (500 MHz, DMSO-*d*$_6$): δ (ppm) = 10.46 (s, 1H), 8.20 (s, 1H), 7.87 (d, *J* = 8.5 Hz, 3H), 7.66 (d, *J* = 8.5 Hz,

2H), 3.98 (d, J = 9.6 Hz, 1H), 3.72 (dd, J = 6.3, 13.4 Hz, 1H), 3.59 (dd, J = 6.0, 13.4 Hz, 1H), 3.53 (d, J = 9.8 Hz, 1H), 3.46-3.40 (m, 1H), 1.96-1.93 (m, 1H), 1.61-1.59 (m, 2H), 138-1.37 (m, 1H), 1.27-1.17 (m, 1H), 1.12-1.08 (m, 1H), 0.89 (d, J = 6.5 Hz, 3H), 0.87-0.82 (m, 1H), 0.79-0.71 (m, 1H); ^{13}C NMR (125 MHz, DMSO-d_6): δ (ppm) = 158.9 (C_q), 154.2 (C_q), 147,8 (CH), 142.9 (C_q), 126.3 (CH), 123.8 (C_q), 120.3 (CH), 105.4 (C_q), 80.7 (CH), 79.2 (CH_2), 78.6 (CH_2), 52.5 (CH), 47.4 (CH_2), 40.4 (CH_2), 34.4 (CH_2), 30.9 (CH), 22.5 (CH_2), 22.4 (CH_3); HRMS (ESI): *m*/z $[M+H]^+$ calculated for $C_{21}H_{25}ClF_3N_4O_2$ 457.1540, found 457.1615.

(1S,2R,5R)-2-((S)-1-((5-Chloro-2-((4-(trifluoromethyl)phenyl)amino)pyrimidin-4-yl)amino)-2-hydroxypropan-2-yl)-5-methylcyclohexan-1-ol hydrochloride (**22f**): Prepared from **21f**. White crystals (87%); m.p. 137–140 °C; $[\alpha]_D^{20}$ = +29.0 (c 0.1325, MeOH); ^{1}H NMR (500 MHz, DMSO-d_6): δ (ppm) = 10.45 (s, 1H), 8.17 (s, 1H), 8.08 (brs, 1H), 7.89 (d, J = 8.8 Hz, 2H), 7.66 (d, J = 8.8 Hz, 2H), 4.19 (s, 1H), 3.61-3.50 (m, 2H), 1.80-1.55 (m, 5H), 1.33-1.23 (m, 1H), 1.16 (s, 3H), 1.03-0.98 (m, 1H), 0.91-0.82 (m, 1H), 0.83 (d, J = 6.3 Hz, 3H); ^{13}C NMR (125 MHz, DMSO-d_6): δ (ppm) = 158.6 (C_q), 154.1 (C_q), 145,6 (CH), 143.0 (C_q), 126.4 (CH), 120.1 (CH), 105.4 (C_q), 73.5 (C_q), 65.4 (CH), 49.9 (CH), 49.0 (CH_2), 43.2 (CH_2), 35.4 (CH_2), 31.2 (CH), 25.8 (CH_3), 22.7 (CH_3), 21.4 (CH_2); HRMS (ESI): *m*/z $[M+H]^+$ calculated for $C_{21}H_{27}ClF_3N_4O_2$ 459.1696, found 459.1769.

(1S,2S,5R)-2-((S)-1-((5-Chloro-2-((4-(trifluoromethyl)phenyl)amino)pyrimidin-4-yl)amino)-3-hydroxypropan-2-yl)-5-methylcyclohexan-1-ol hydrochloride (**22g**): Prepared from **21g**. White crystals (95%); m.p. 162–164 °C; $[\alpha]_D^{20}$ = +2.0 (c 0.1150, MeOH); ^{1}H NMR (500 MHz, DMSO-d_6): δ (ppm) = 10.15 (brs, 1H), 8.10 (s, 1H), 8.02 (brs, 1H), 7.91 (d, J = 8.2 Hz, 2H), 7.63 (d, J = 8.4 Hz, 2H), 3.83 (s, 1H), 3.62-3.56 (m, 3H), 3.49-3.45 (m, 1H), 1.84-1.63 (m, 4H), 1.50-1.45 (m, 3H), 1.07-0.98 (m, 1H), 0.89-0.84 (m, 1H), 0.81 (d, J = 6.4 Hz, 3H); ^{13}C NMR (125 MHz, DMSO-d_6): δ (ppm) = 158.2 (C_q), 143.5 (C_q), 148.5 (CH), 126.3 (CH), 119.7 (CH), 105.3 (C_q), 66.9 (CH), 61.6 (CH_2), 43.0 (CH_2), 42.8 (CH_2), 42.5 (CH), 40.9 (CH), 35.4 (CH_2), 25.8 (CH), 23.6 (CH_2), 22.8 (CH_3); HRMS (ESI): m/z $[M+H]^+$ calculated for $C_{21}H_{27}ClF_3N_4O_2$ 459.1696, found 459.1761.

(3R,3aR,6R,7aS)-3-(((5-Chloro-2-((4-(trifluoromethyl)phenyl)amino)pyrimidin-4-yl)amino)methyl)-6-methyloctahydrobenzofuran-3-ol hydrochloride (**22h**): Prepared from **21h**. Yellow crystals (85%); m.p. 223–225 °C; $[\alpha]_D^{20}$ = –30.0 (c 0.1350, MeOH); ^{1}H NMR (500 MHz, DMSO-d_6): δ (ppm) = 10.08 (s, 1H), 8.13 (s, 1H), 7.88 (d, J = 8.6 Hz, 2H), 7.63 (d, J = 8.5 Hz, 2H), 4.29-4.28 (m, 1H), 3.87 (d, J = 9.4 Hz, 1H), 3.75 (dd, J = 5.6, 13.4 Hz, 1H), 3.58 (dd, J = 5.3, 13.5 Hz, 1H), 3.49 (d, J = 9.4 Hz, 1H), 1.87-1.85 (m, 1H), 1.77-1.72 (m, 1H), 1.61-1.52 (m, 2H), 1.45-1.44 (m, 1H), 1.13-0.99 (m, 1H), 0.83 (d, J = 6.5 Hz, 3H), 0.83-0.77 (m, 1H); ^{13}C NMR (125 MHz, DMSO-d_6): δ (ppm) = 158.4 (C_q), 150,0 (CH),143.6 (C_q), 126.2 (CH), 119.9 (CH), 105.3 (C_q), 82.8 (CH_2), 76.8 (CH), 75.9 (CH_2), 46.9 (CH), 45.1 (CH_2), 37.0 (CH_2), 33.0 (CH_2), 26.5 (CH), 24.1 (CH_2), 22.6 (CH_3); HRMS (ESI): *m*/z $[M+H]^+$ calculated for $C_{21}H_{25}ClF_3N_4O_2$ 457.1540, found 457.1613.

1.6. Determination of antiproliferative effect

The growth-inhibitory effects of the presented heterocyclic compounds were determined by a standard MTT (3-(4,5-dimethylthiazol-2-yl)-2,5-diphenyltetrazolium bromide) assay on a panel of human adherent cancer cell lines of gynecological origin containing HeLa and SiHa (cervical cancers), A2780 (ovarian cancer) and MDA-MB-231 (breast cancer) cells.[7] All cell lines were purchased from the European Collection of Cell Cultures (Salisbury, UK) except the SiHa, obtained from the American Tissue Culture Collection (Manassas, VA, USA). The cells were cultivated in minimal essential medium (MEM) supplemented with fetal bovine serum

(10%), non-essential amino acids, and penicillin-streptomycin (1% each) at 37 °C in a humidified atmosphere containing 5% CO_2. All media and supplements were obtained from Lonza Group Ltd. (Basel, Switzerland). Cancer cells were plated into 96-well plates at the density of 5000 cells/well. After overnight incubation, the test compound was added in two concentrations (10 μM and 30 μM) and incubated for 72 h under cell-culturing conditions. Then, MTT solution (5 mg/mL, 20 μL) was added to each well and incubated for four h. Finally, the medium was removed, and the precipitated formazan was dissolved in DMSO during 60 min of shaking at 37 °C. The absorbance was measured at 545 nm using a microplate reader (SpectoStarNano, BMG Labtech, Ortenberg, Germany). Two independent experiments were carried out with five wells for each condition. Cisplatin (Ebewe GmbH, Unterach, Austria) was used as a positive control. Calculations were performed utilising the GraphPad Prism 5.01 software (GraphPad Software Inc., San Diego, CA, USA).

2. Investigation of antiproliferative activity of the (–)-isopulegol-based heterocyclic compounds

Table 1. Antiproliferative properties of the heterocyclic derivatives

Compound	Chemical structure	Conc. (μM)	Growth Inhibition (%) ± SEM			
			HeLa	SiHa	A2780	MDA-MB-231
**16a**	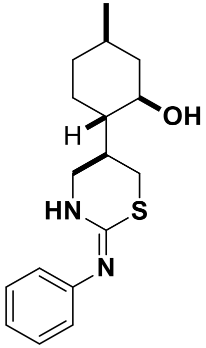	10	-	-	-	-
		30	18.49 ± 3.50	12.55 ± 11.74	-	-
**16b**	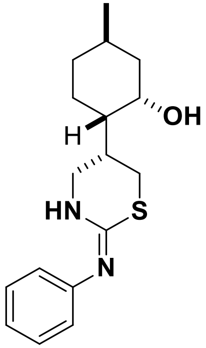	10	31.45 ± 1.55	47.12 ± 1.72	55.02 ± 1.27	-
		30	83.37 ± 0.46	65.69 ± 1.19	82.06 ± 0.28	41.58 ± 1.09

**17a**	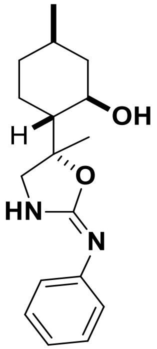	10	17.07 ± 1.74	22.41 ± 1.96	-	-
		30	19.03 ± 4.25	32.40 ± 1.49	12.01 ± 0.38	13.60 ± 4.66
**17b**	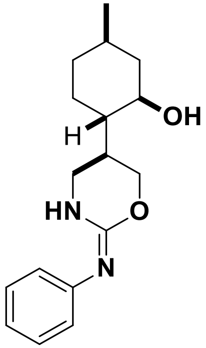	10	-	-	-	-
		30	-	12.06 ± 7.06	-	-
**17c**	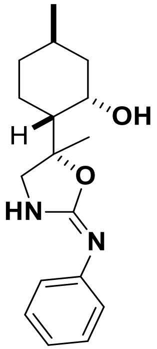	10	-	12.66 ± 2.97	25.21 ± 1.92	-
		30	19.22 ± 2.38	17.75 ± 1.58	37.98 ± 2.82	-

**17d**	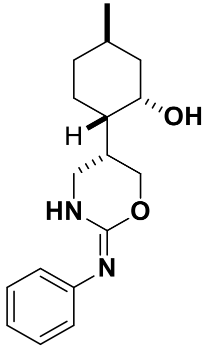	10	-	26.44 ± 1.24	-	-
		30	23.04 ± 1.82	43.07 ± 1.25	23.21 ± 2.43	-
**19a**	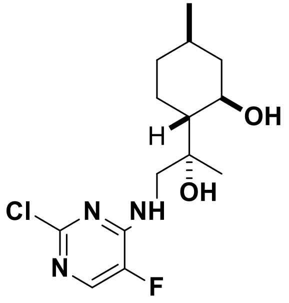	10	-	-	-	-
		30	26.06 ± 2.24	34.63 ± 1.55	-	-
**20a**	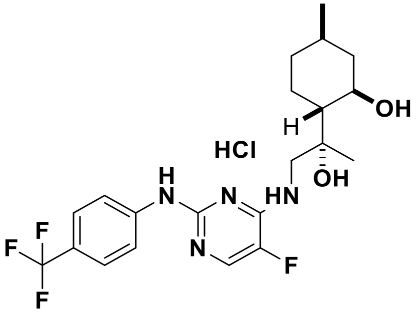	10	75.38 ± 0.53	54.14 ± 1.05	75.64 ± 2.25	32.06 ± 1.94
		30	86.75 ± 0.41	89.19 ± 0.81	92.14 ± 0.49	67.79 ± 1.09

**20b**	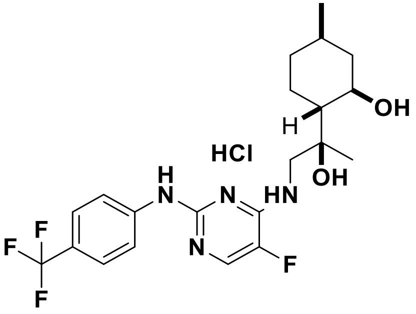	10	94.62 ± 0.35	84.94 ± 1.11	91.50 ± 0.36	55.65 ± 1.26
		30	96.90 ± 0.46 (IC_{50}: 7.20 μM)	95.89 ± 0.74 (IC_{50}: 2.62 μM)	96.58 ± 0.76 (IC_{50}: 4.98 μM)	99.82 ± 1.61 (IC_{50}: 9.91 μM)**
**20c**	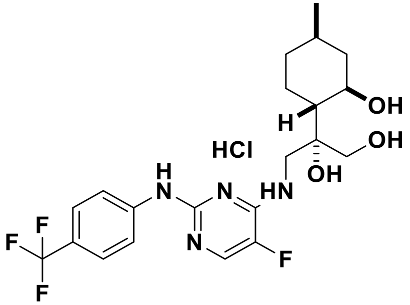	10	28.69 ± 1.54	41.01 ± 0.99	21.14 ± 1.22	-
		30	94.95 ± 0.45	84.07 ± 1.20	94.83 ± 0.10	63.52 ± 1.17
**20d**	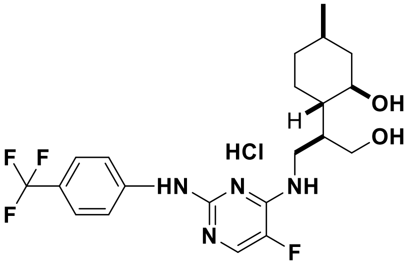	10	19.49 ± 3.01	31.56 ± 0.93	79.18 ± 0.49	-
		30	92.06 ± 0.36	92.45 ± 0.54	94.42 ± 0.30	74.72 ± 2.11

**20e**	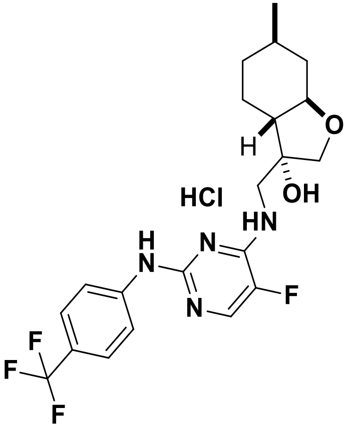	10	12.59 ± 1.39	26.37 ± 2.64	28.11 ± 3.60	-
		30	50.98 ± 3.52	37.05 ± 0.77	70.09 ± 1.19	-
**20f**	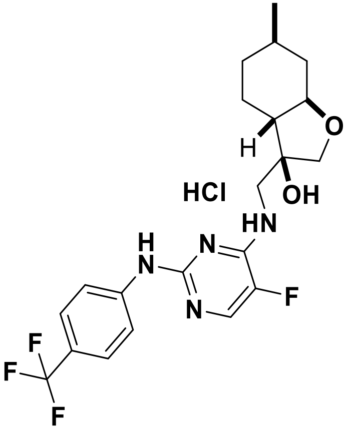	10	13.77 ± 2.29	24.96 ± 0.39	-	-
		30	92.44 ± 1.47	74.26 ± 3.53	89.04 ± 2.28	74.60 ± 2.78

**20g**	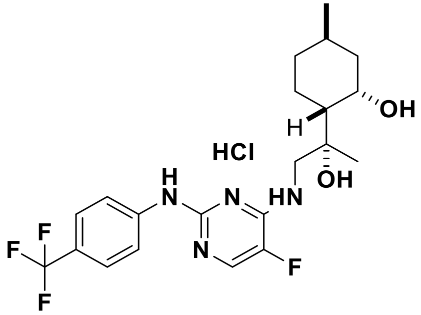	10	37.11 ± 3.39	32.92 ± 0.91	31.49 ±2 .40	-
		30	70.69 ± 1.19	58.90 ± 0.48	98.36 ± 0.29	56.98 ± 2.27
**20h**	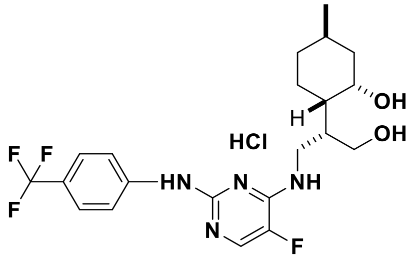	10	30.15 ± 1.99	11.05 ± 2.92	11.29 ± 1.11	-
		30	59.09 ± 2.27	37.91 ± 1.40	56.94 ± 1.11	34.49 ± 1.63
**20i**	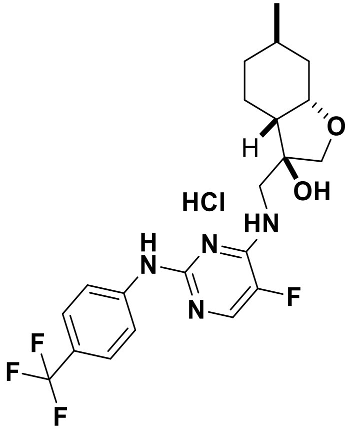	10	65.72 ± 1.07	61.55 ± 1.14	35.26 ± 1.38	22.45 ± 1.11
		30	68.08 ± 0.82	61.61 ± 1.77	53.75 ± 3.31	37.80 ± 1.32

**22a**	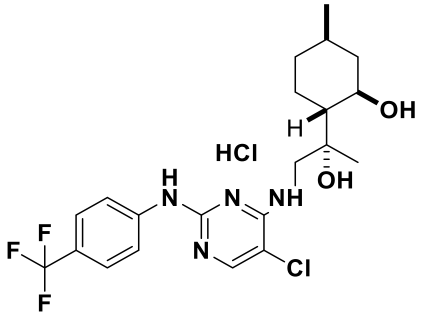	10	27.66 ± 0.60	35.35 ± 1.00	78.25 ± 0.75	29.03 ± 2.20
		30	93.40 ± 0.26	89.59 ± 0.54	92.12 ± 0.32	88.09 ± 0.54
**22b**	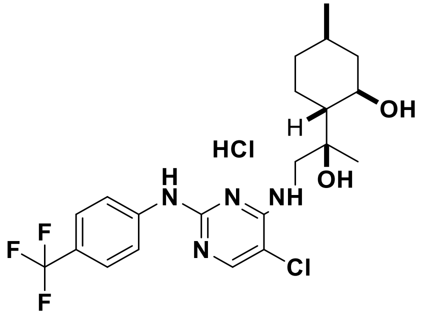	10	96.00 ± 0.39	76.02 ± 2.05	96.26 ± 0.58	40.32 ± 2.18
		30	96.55 ± 0.43 (IC_{50}: 9.03 μM)**	95.32 ± 0.43 (IC_{50}: 5.69 μM)	96.69 ± 0.23 (IC_{50}: 4.13 μM)	96.16 ± 1.09 (IC_{50}: 11.47 μM)
**22c**	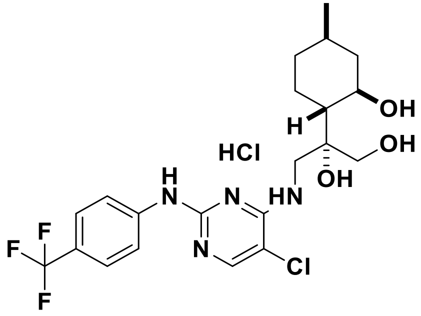	10	18.42 ± 3.17	21.84 ± 2.29	28.15 ± 2.32	-
		30	92.99 ± 0.27	72.33 ± 2.40	93.16 ± 0.40	74.01 ± 0.88

**22d**	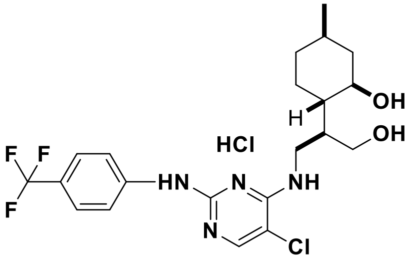	10	10.34 ± 3.63	40.16 ± 1.16	85.06 ± 0.76	35.89 ± 2.21
		30	95.36 ± 0.18	84.47 ± 1.88	98.38 ± 0.25	86.02 ± 1.99
**22e**	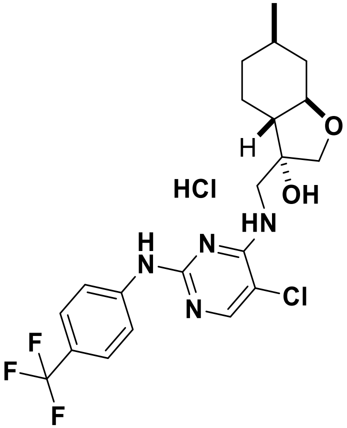	10	19.89 ± 1.35	23.15 ± 2.39	40.41 ± 3.41	-
		30	70.27 ± 1.10	56.35 ± 0.99	70.58 ± 0.58	57.28 ± 1.17
**22f**	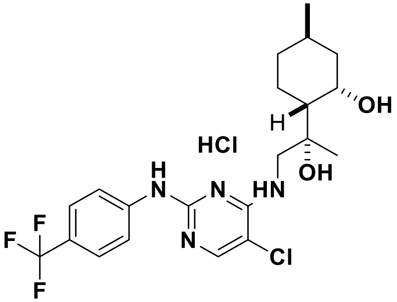	10	87.71 ± 1.54	64.44 ± 3.50	97.23 ± 0.31	75.56 ± 2.58
		30	96.06 ± 0.27	88.34 ± 0.46	98.33 ± 0.25	93.30 ± 1.16

**22g**	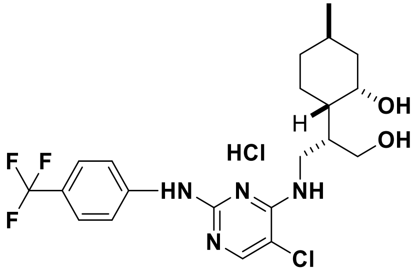	10	11.25 ± 0.71	11.59 ± 1.36	-	-
		30	86.55 ± 1.13	53.03 ± 1.51	81.97 ± 1.60	60.94 ± 1.82
**22h**	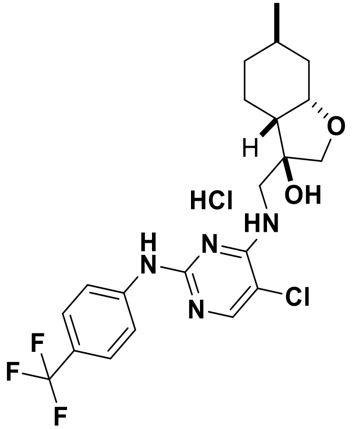	10	70.16 ± 0.56	62.63 ± 1.16	60.44 ± 1.63	33.56 ± 1.83
		30	80.14 ± 0.97	69.85 ± 2.19	79.28 ± 1.75	78.64 ± 0.97
**23a**	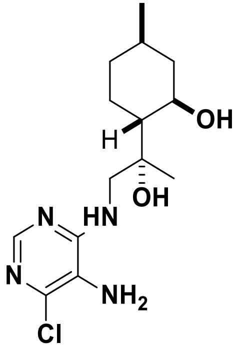	10	64.20 ± 0.58	23.18 ± 2.74	36.80 ± 2.37	-
		30	68.10 ± 2.44	29.79 ± 2.10	51.77 ± 1.29	-

**23b**	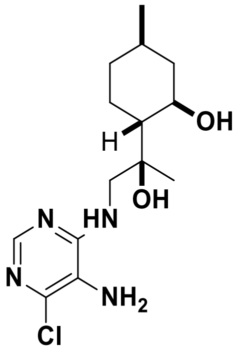	10	52.61 ± 1.56	-	-	10.68 ± 2.36
		30	58.33 ± 0.21	21.55 ± 2.37	36.23 ± 3.13	11.82 ± 3.71
**23c**	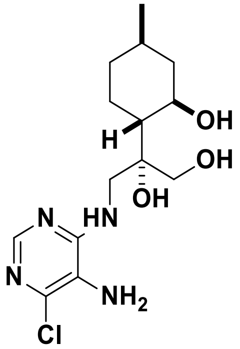	10	57.62 ± 1.48	-	18.39 ± 1.03	-
		30	72.53 ± 2.44	17.93 ± 2.00	43.50 ± 0.72	-
**23d**	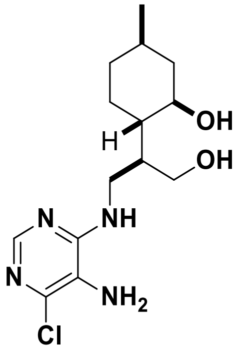	10	48.75 ± 3.01	-	35.06 ± 1.27	-
		30	69.46 ± 2.70	-	46.78 ± 2.75	-

**23e**	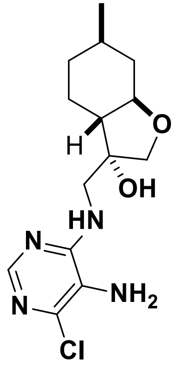	10	58.49 ± 1.05	20.33 ± 0.35	29.57 ± 0.56	-
		30	60.88 ± 4.23	31.47 ± 1.20	42.16 ± 1.12	11.47 ± 0.24
**23f**	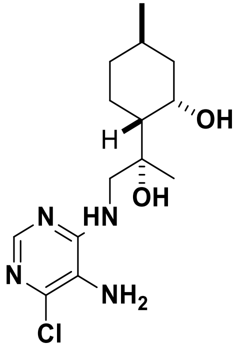	10	50.49 ± 1.19	-	36.18 ± 3.29	-
		30	83.37 ± 1.91	14.29 ± 3.03	44.75 ± 3.08	11.51 ± 1.94
**23h**	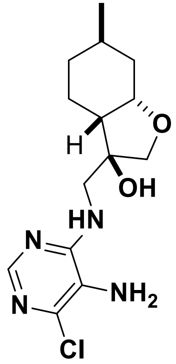	10	60.12 ± 1.61	-	20.71 ± 2.02	-
		30	63.35 ± 1.14	17.49 ± 4.25	42.90 ± 2.06	18.79 ± 1.76
**Cisplatin**		10	42.61 ± 2.33	60.98 ± 0.92	83.57 ± 2.21	42.72 ± 2.68
		30	99.93 ± 0.26	88.95 ± 0.53	95.02 ± 0.28	86.44 ± 0.42

			(IC$_{50}$: 12.43 μM)**	(IC$_{50}$: 4.29 μM)	(IC$_{50}$: 1.30 μM)	(IC$_{50}$: 10.17 μM)

* Cancer cell growth inhibition values less than 10% were considered insignificant and are not given numerically

** In the case of the most effective test compounds (**20b** and **22b**) and reference agent cisplatin, the viability assays were repeated with a set of dilutions (0.1–30 μM), and the IC$_{50}$ values were determined using GraphPad Prism 5.01 software (GraphPad Software Inc., San Diego, CA, USA).

During our antiproliferative work we intended to use a practical investigational approach, therefore, we focused on the present treatment guidelines of gynecological cancers to choose a long time available, well-characterised positive control molecule which is recommended for the therapy of all cancer types included in our experiment (namely, breast, cervical and ovarian cancer). According to the online available guidelines of the European Society of Medical Oncology (ESMO) platinum compounds, like cisplatin, are effective in the clinical treatment of different gynecological cancers.

Platinum-based therapy is still the first-line treatment option in local cervical cancer and it can be beneficial in combination with paclitaxel or topotecan in the therapy of advanced/metastatic cervical cancer.[8] Moreover, patients suffering from either primary (early stage or epithelial form) or recurrent ovarian cancer can be treated with platinum derivatives, in most cases as part of combination chemotherapy.[9] Among numerous different types of breast cancer according to their pathophysiological or developmental origin, for most triple negative breast cancer (defined by the absence of expression of estrogen and progesterone receptors and of overexpression of HER2 or amplification of HER2neu) chemotherapy included platinum derivatives remains the standard treatment.[10] MDA-MB-231 cell line used in our work is an experimentally applicable triple negative breast cancer cell line.

3. ^{1}H-, ^{13}C- NMR spectra of new heterocyclic compounds

S1: ^{1}H- and ^{13}C-NMR of compound (3*S*,3a*R*,6*R*,7a*R*)-6-methyl-3-((((*S*)-1-phenylethyl)amino)methyl)octahydrobenzofuran-3-ol (**8a**)

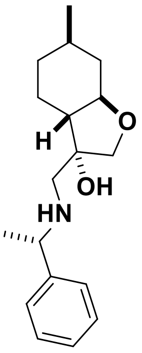

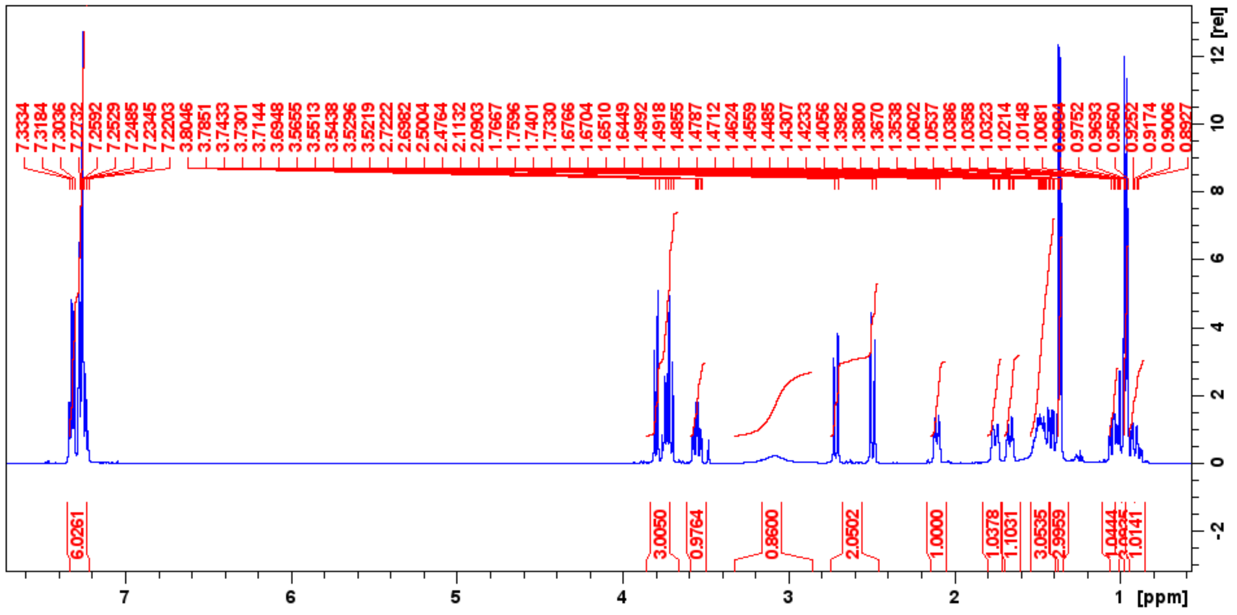

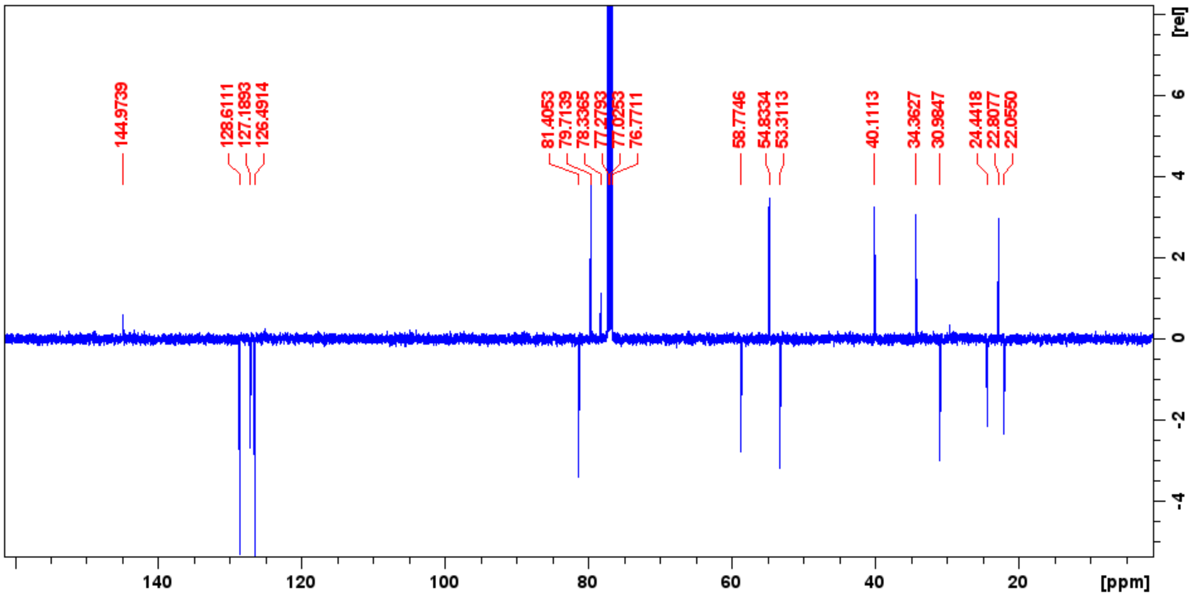

S2: ^{1}H- and ^{13}C-NMR of compound (3*R*,3a*R*,6*R*,7a*R*)-6-Methyl-3-((((S)-1-phenylethyl)amino)methyl)octahydrobenzofuran-3-ol (**8b**)

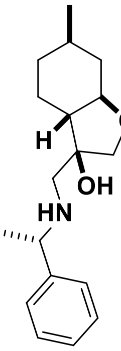

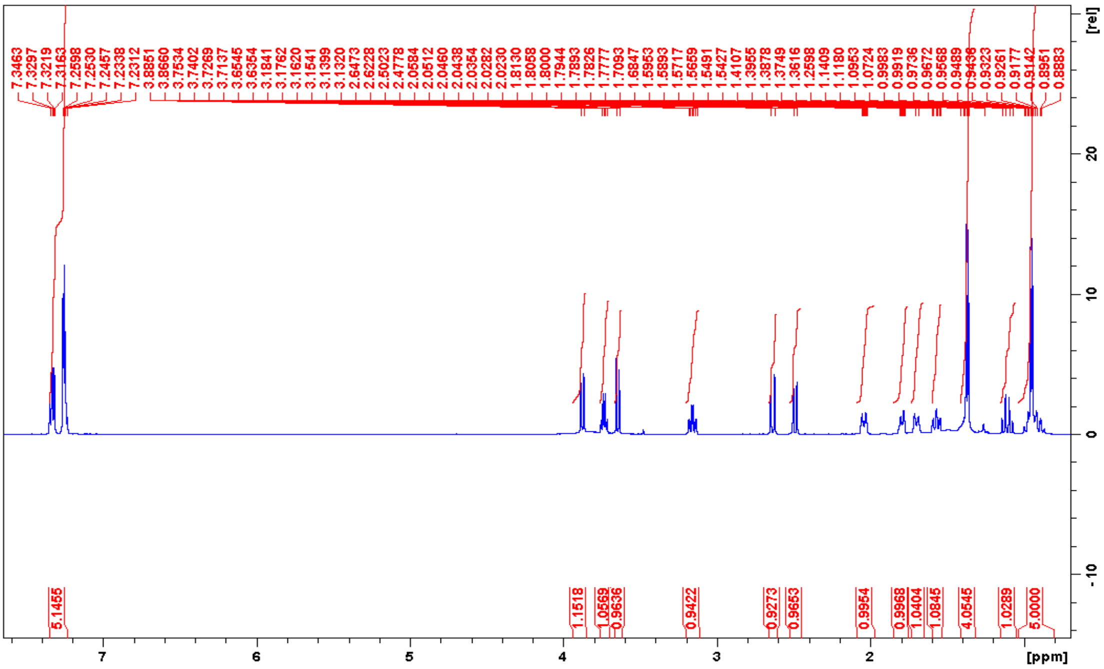

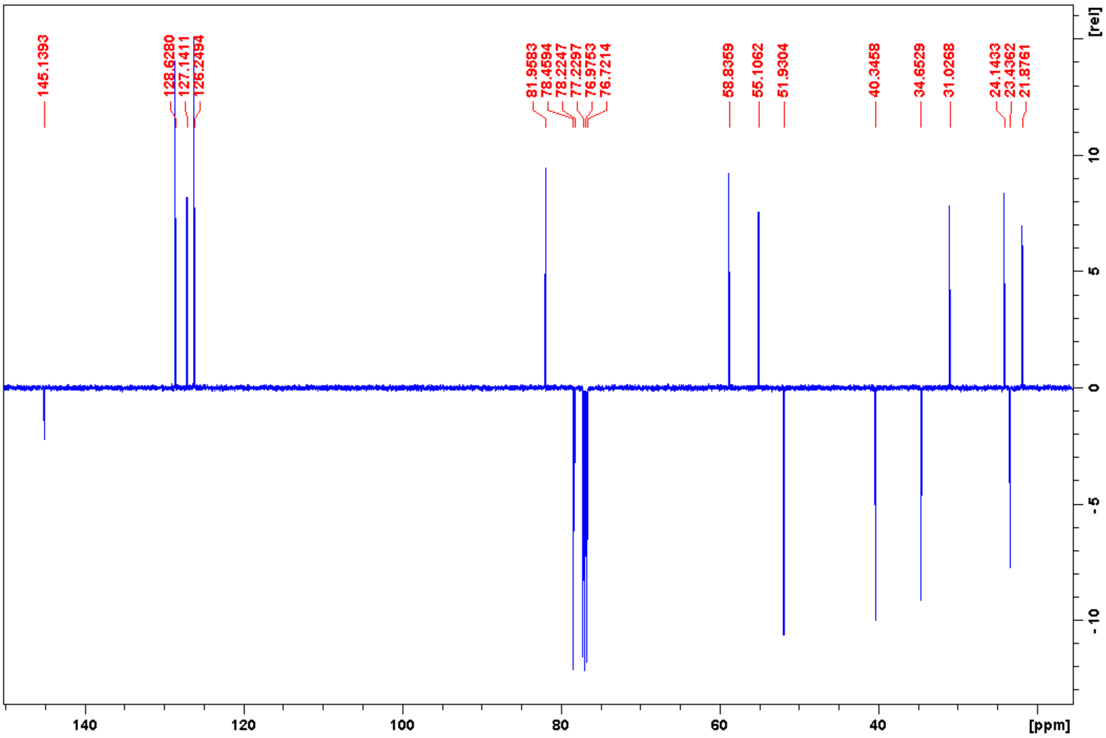

S3: ^{1}H- and ^{13}C-NMR of compound (3*S*,3a*R*,6*R*,7a*R*)-3-(aminomethyl)-6-methyloctahydrobenzofuran-3-ol (**8c**)

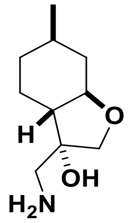

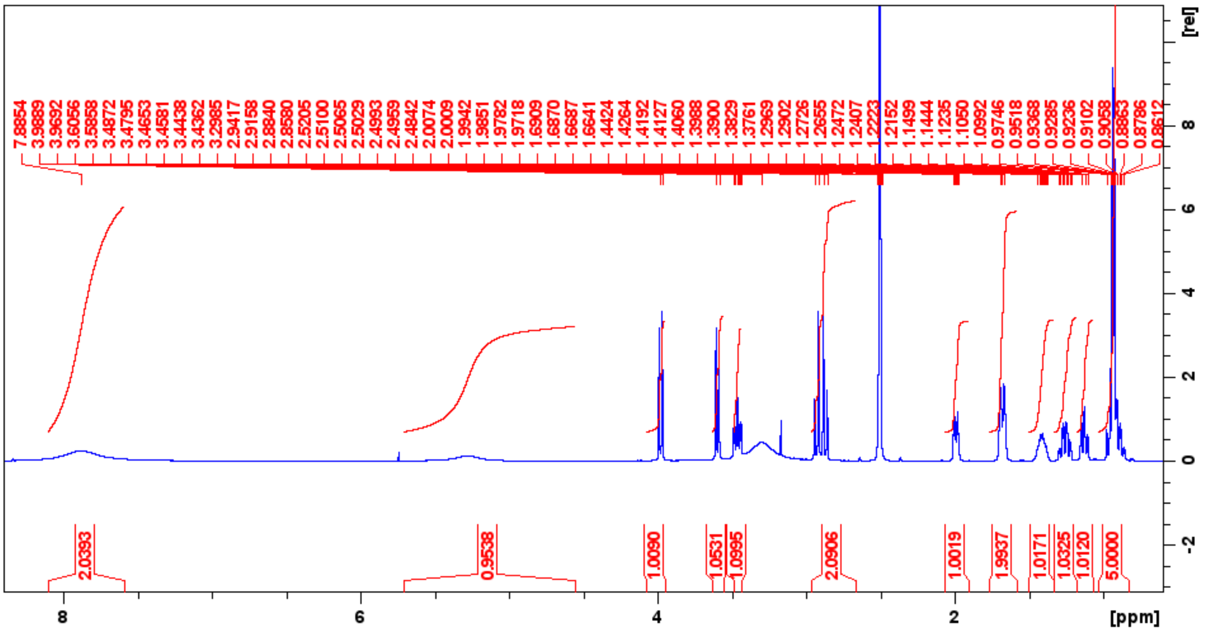

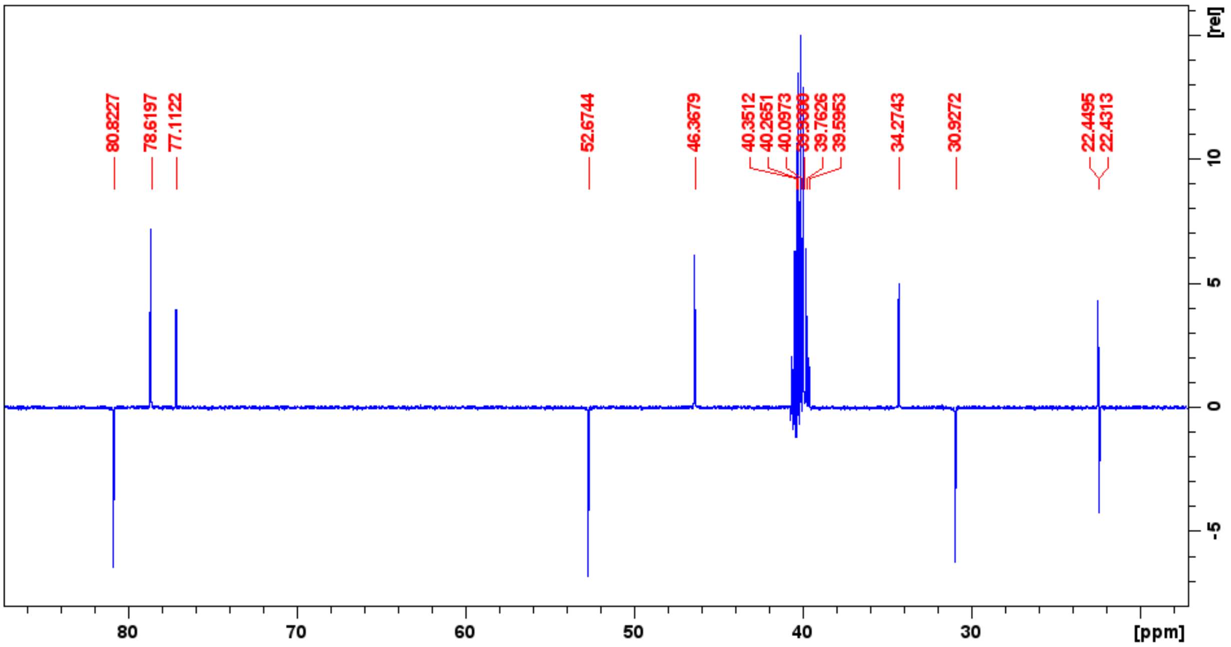

S4: ^{1}H- and ^{13}C-NMR of compound (3*R*,3a*R*,6*R*,7a*R*)-3-(aminomethyl)-6-methyloctahydrobenzofuran-3-ol (**8d**)

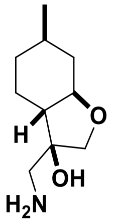

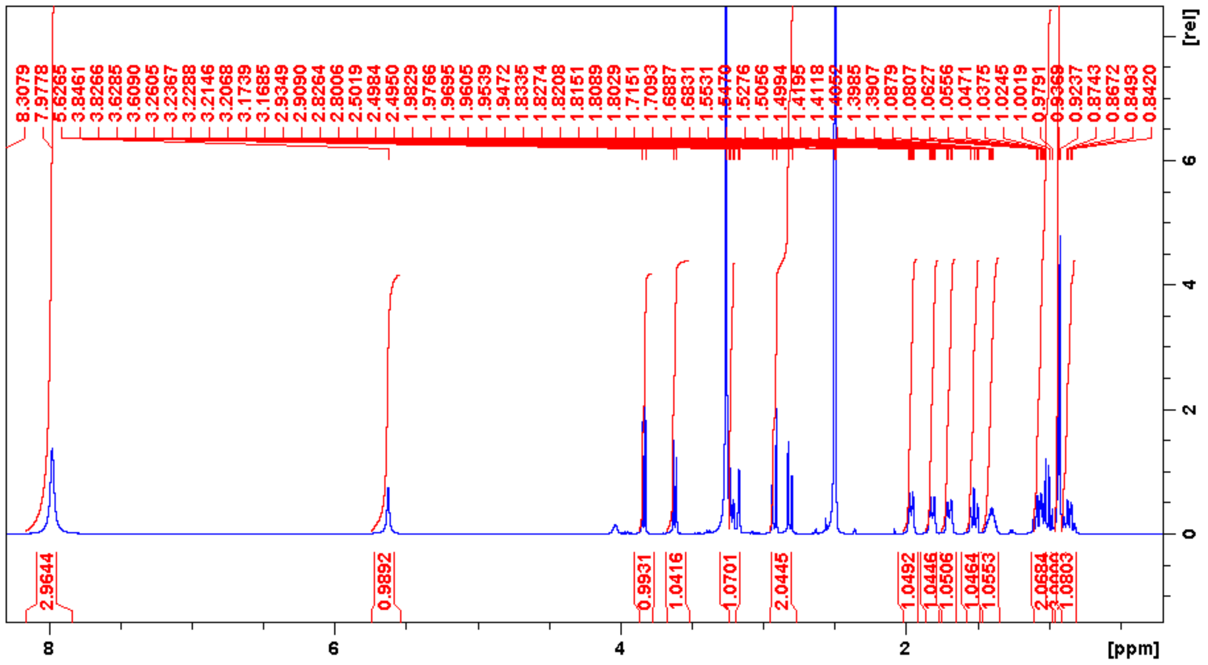

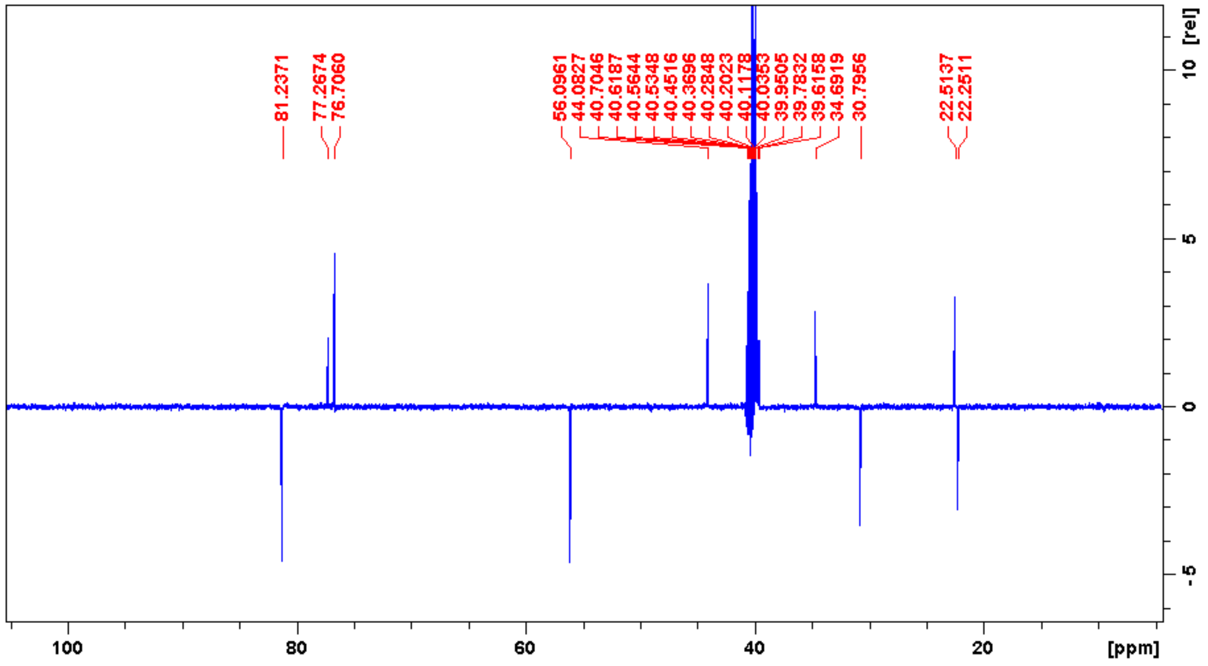

S5: ^{1}H- and ^{13}C-NMR of compound 1-((*S*)-2-hydroxy-2-((1*R*,2*R*,4*R*)-2-hydroxy-4-methylcyclohexyl)propyl)-3-phenylthiourea (**15a**)

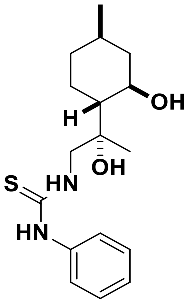

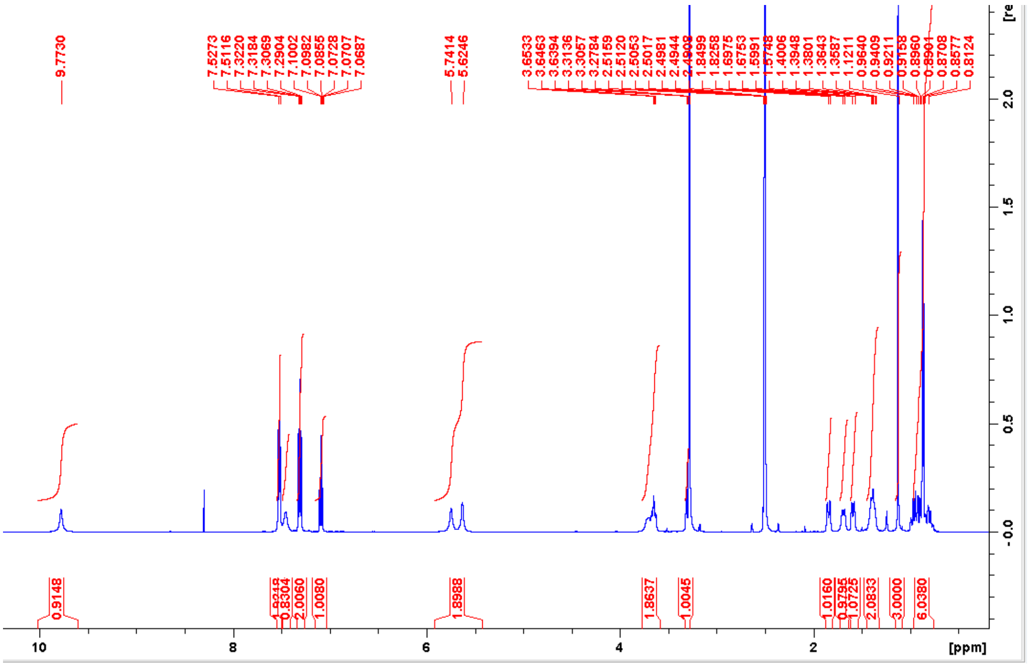

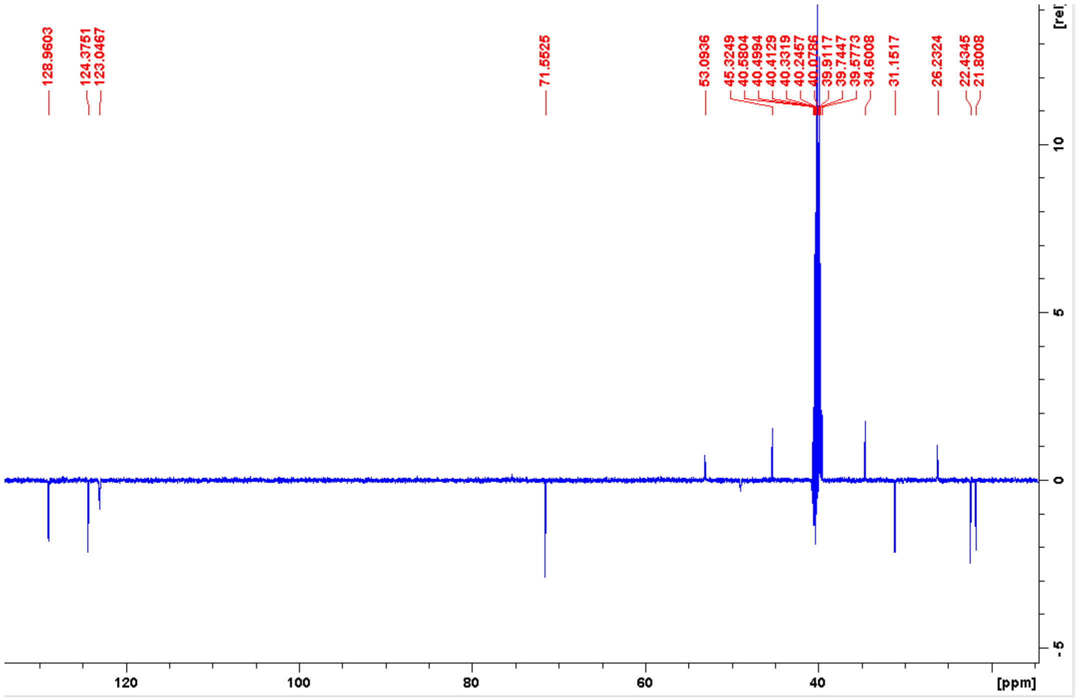

S6: ¹H- and ¹³C-NMR of compound 1-((*R*)-3-hydroxy-2-((1*S*,2*R*,4*R*)-2-hydroxy-4-methylcyclohexyl)propyl)-3-phenylthiourea (**15b**)

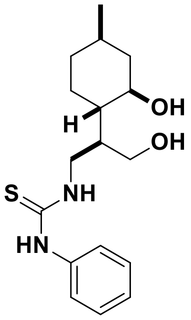

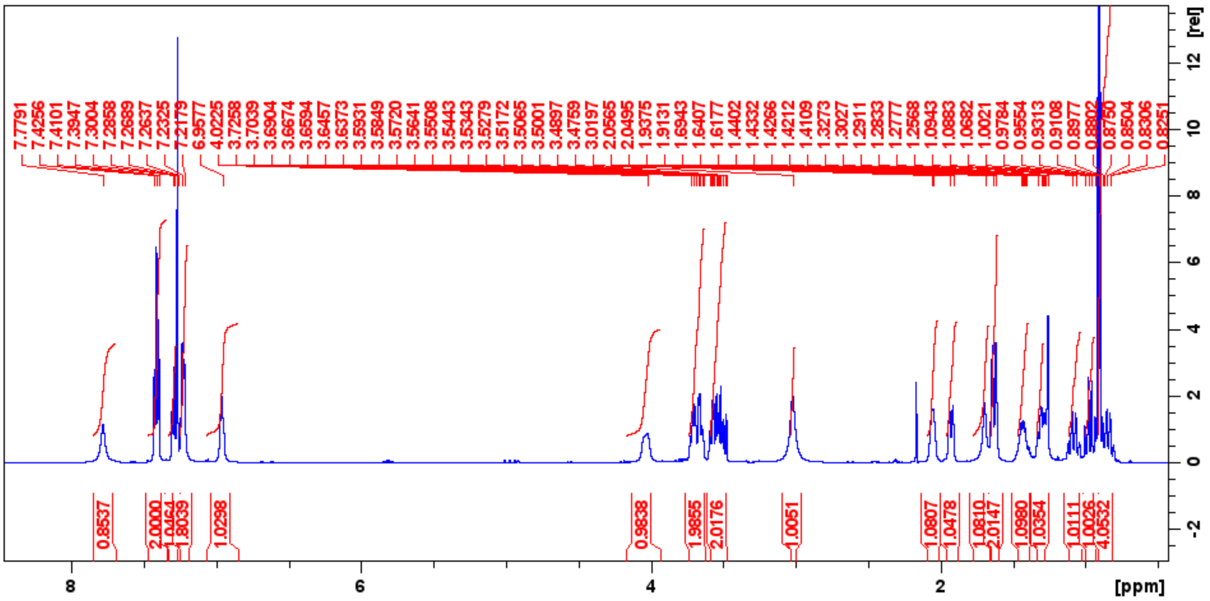

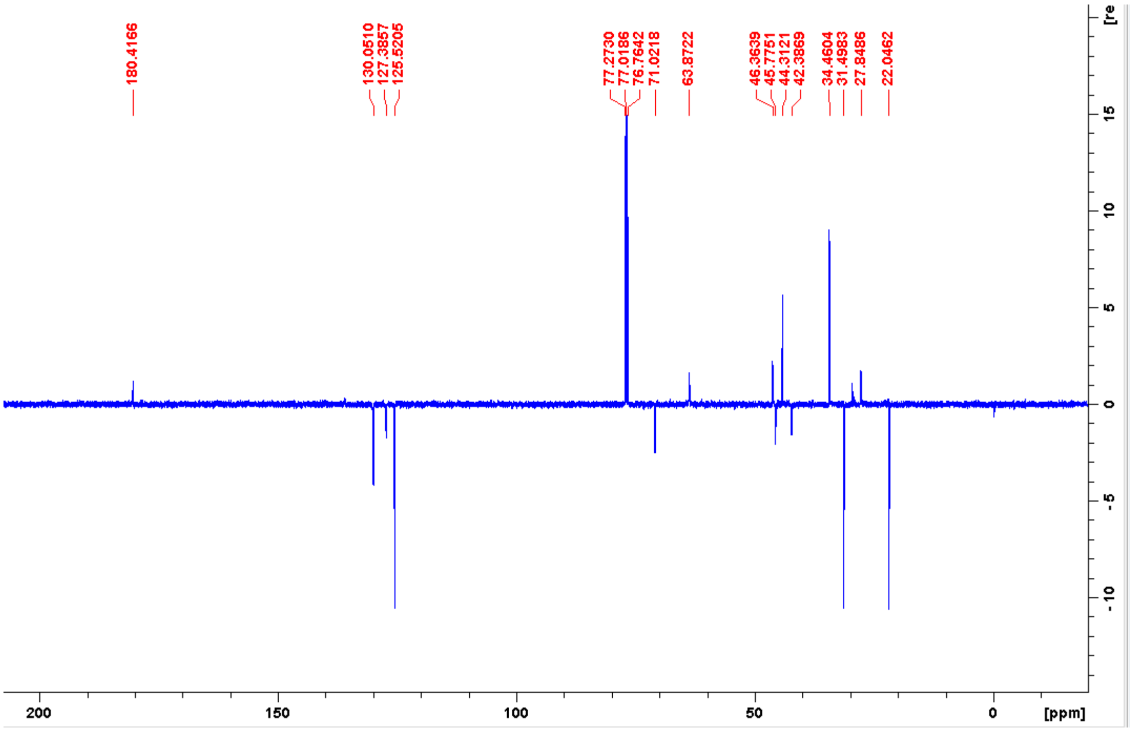

S7: ^{1}H- and ^{13}C-NMR of compound 1-((*S*)-2-hydroxy-2-((1*R*,2*S*,4*R*)-2-hydroxy-4-methylcyclohexyl)propyl)-3-phenylthiourea (**15c**)

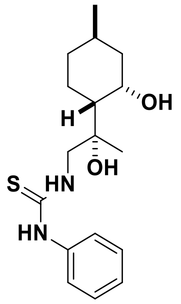

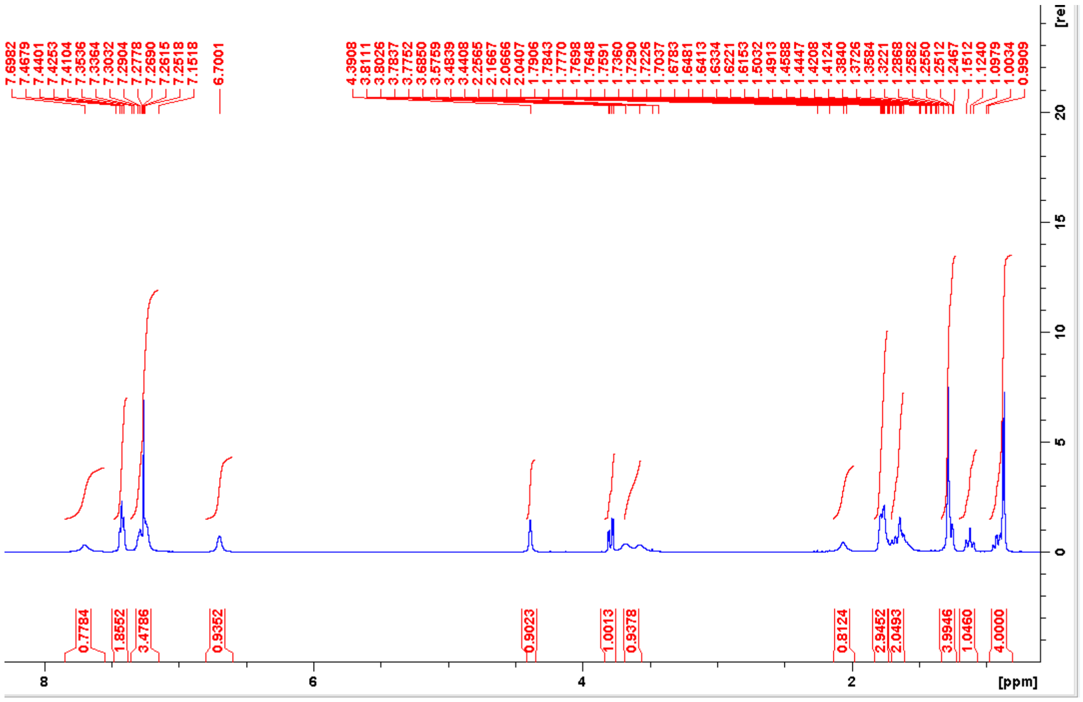

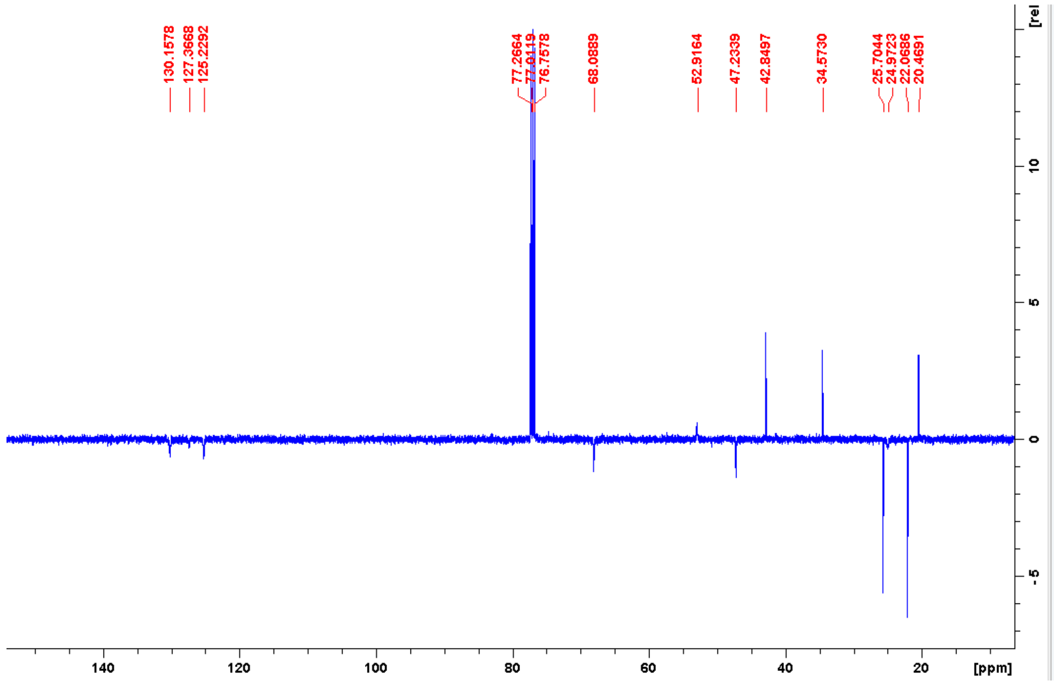

S8: ^{1}H- and ^{13}C-NMR of compound 1-((*S*)-3-hydroxy-2-((1*S*,2*S*,4*R*)-2-hydroxy-4-methylcyclohexyl)propyl)-3-phenylthiourea (**15d**)

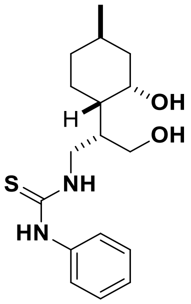

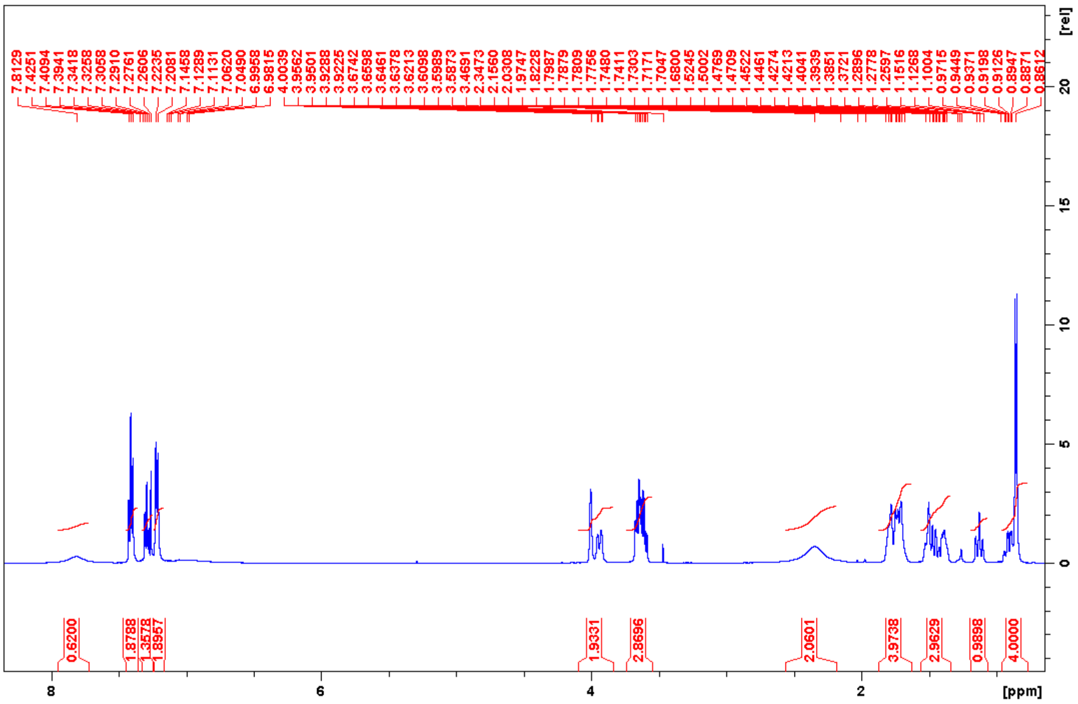

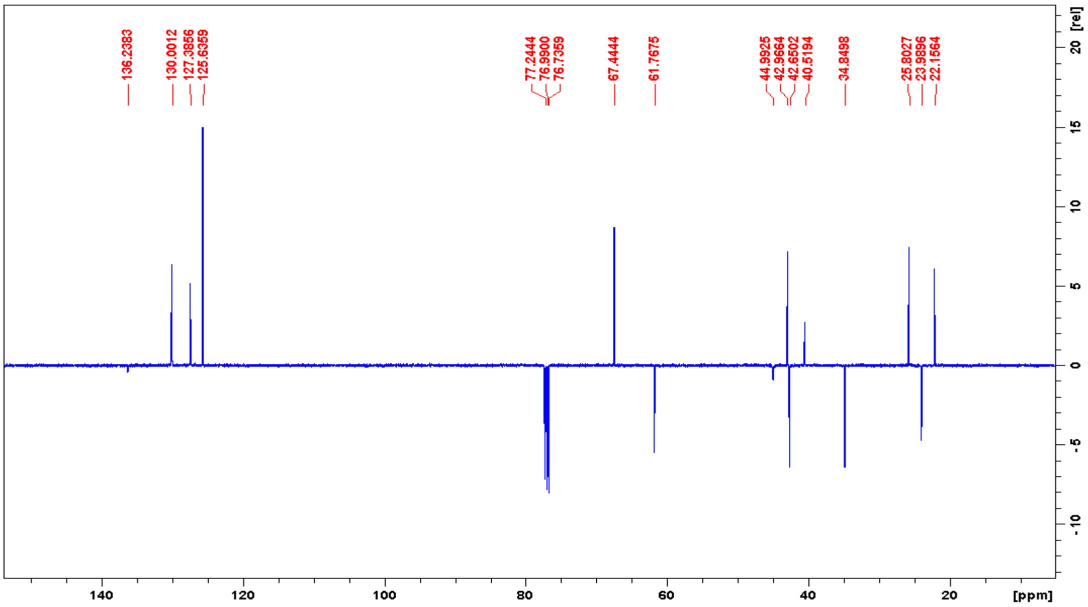

S9: ^{1}H- and ^{13}C-NMR of compound (1*R*,2*S*,5*R*)-5-methyl-2-((*R*)-2-(phenylimino)-1,3-thiazinan-5-yl)cyclohexanol (**16a**)

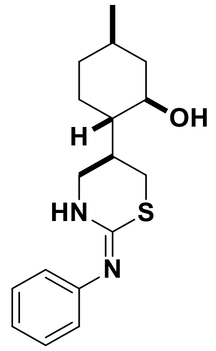

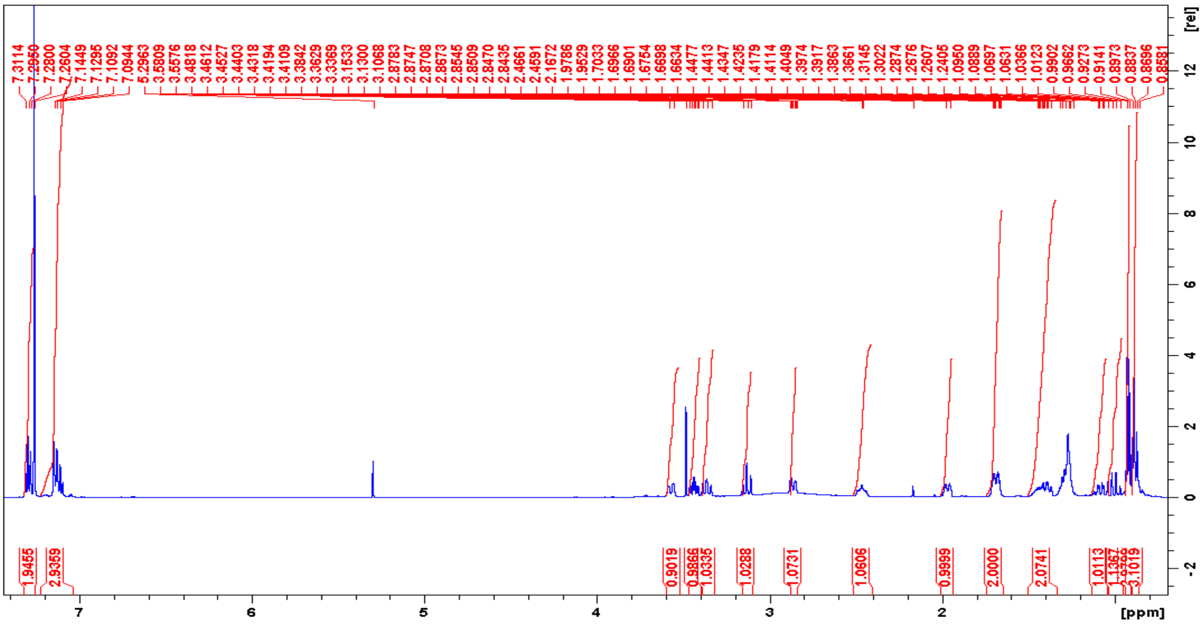

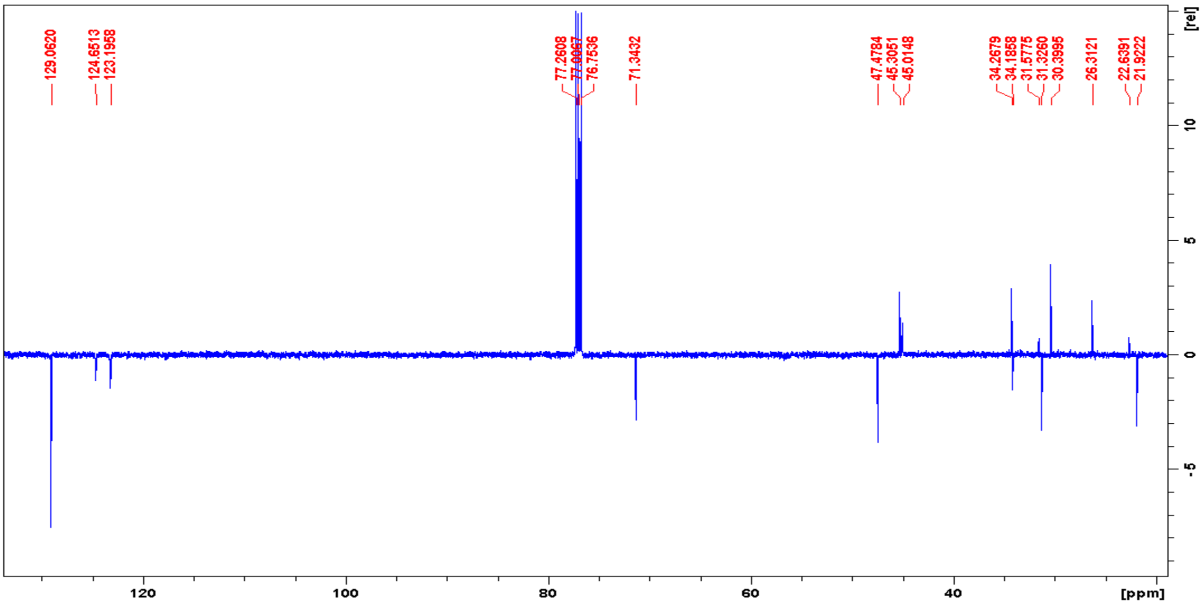

S10: ^{1}H- and ^{13}C-NMR of compound (1*S*,2*S*,5*R*)-5-methyl-2-((*S*)-2-(phenylimino)-1,3-thiazinan-5-yl)cyclohexanol (**16b**)

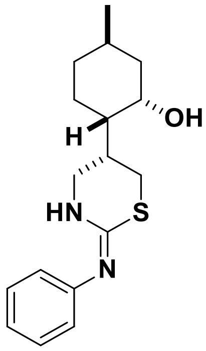

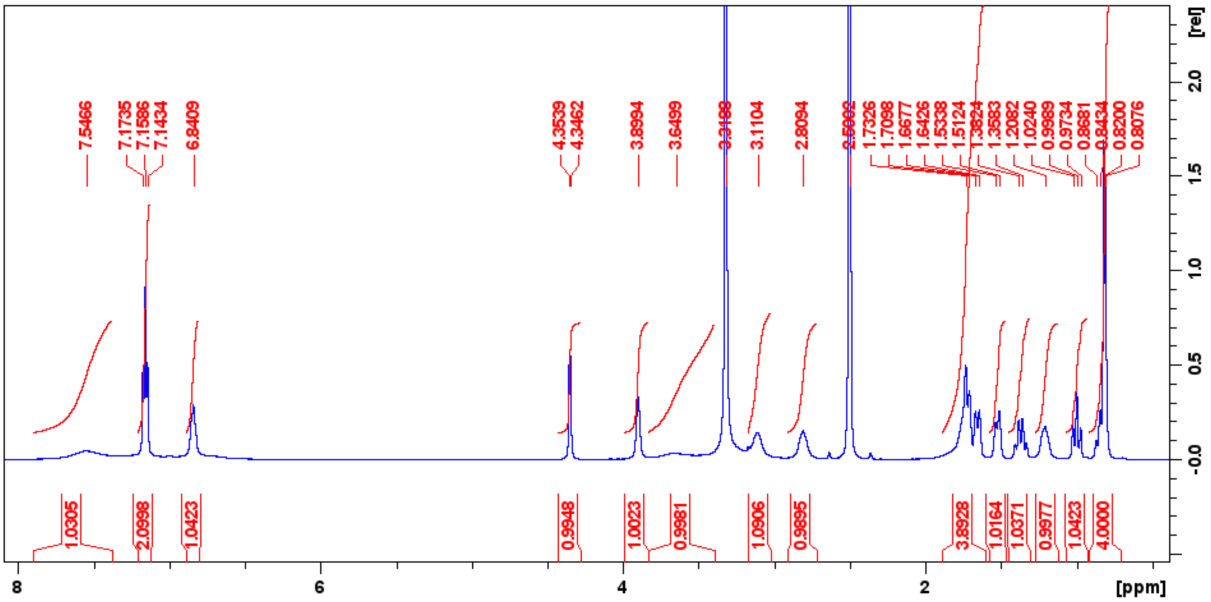

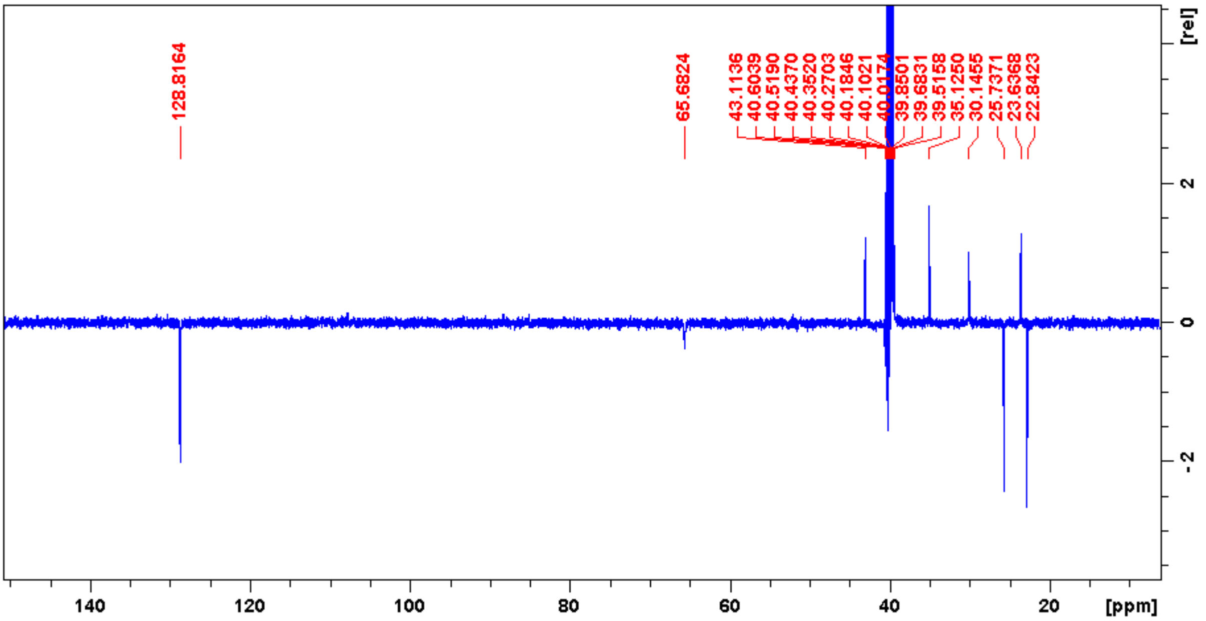

S11: ^{1}H- and ^{13}C-NMR of compound (1*R*,2*R*,5*R*)-5-methyl-2-((*S*)-5-methyl-2-(phenylimino)oxazolidin-5-yl)cyclohexanol (**17a**)

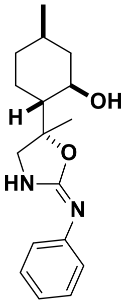

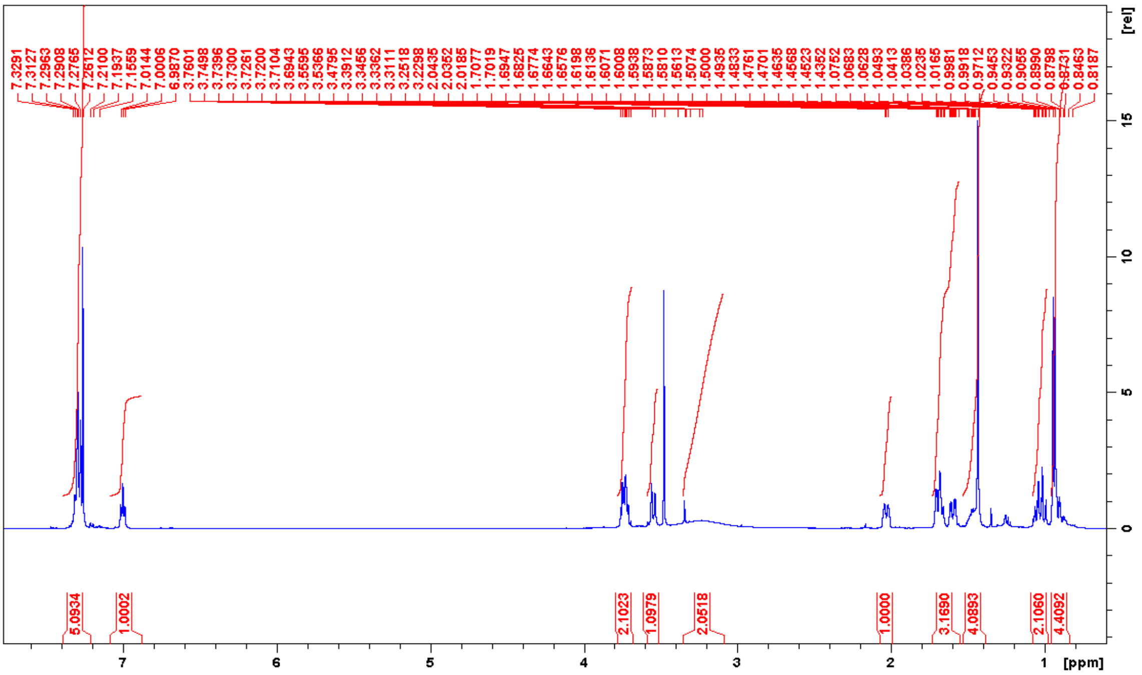

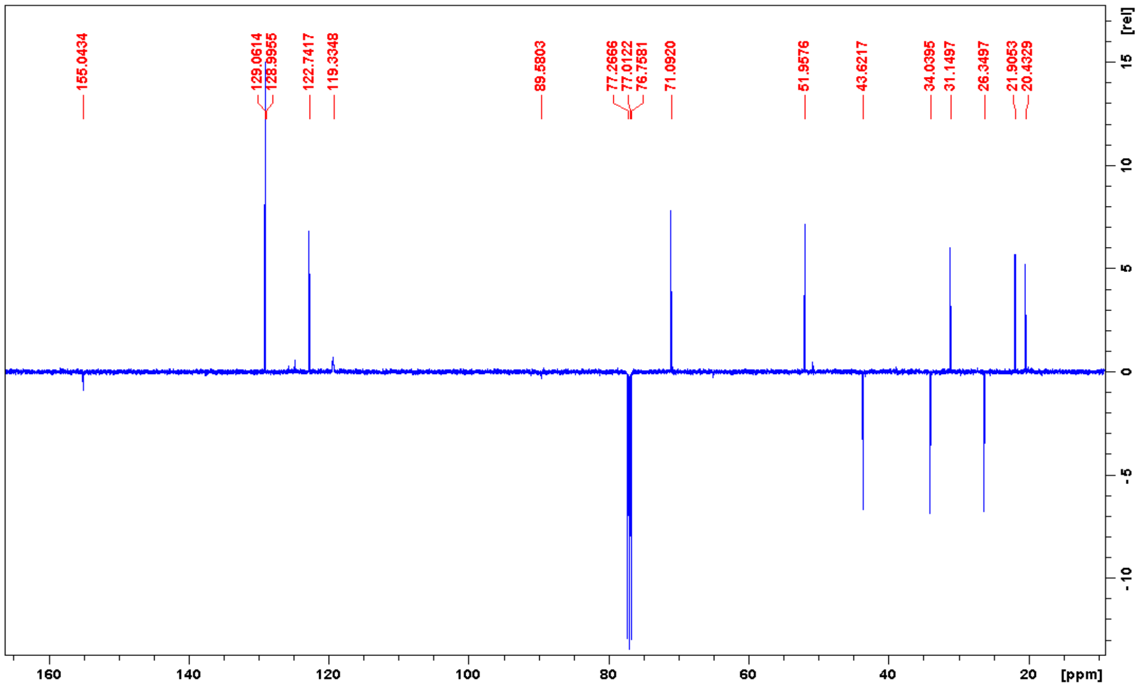

S12: ^{1}H- and ^{13}C-NMR of compound (1*R*,2*S*,5*R*)-5-methyl-2-((*R*)-2-(phenylimino)-1,3-oxazinan-5-yl)cyclohexanol (**17b**)

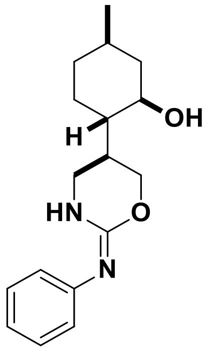

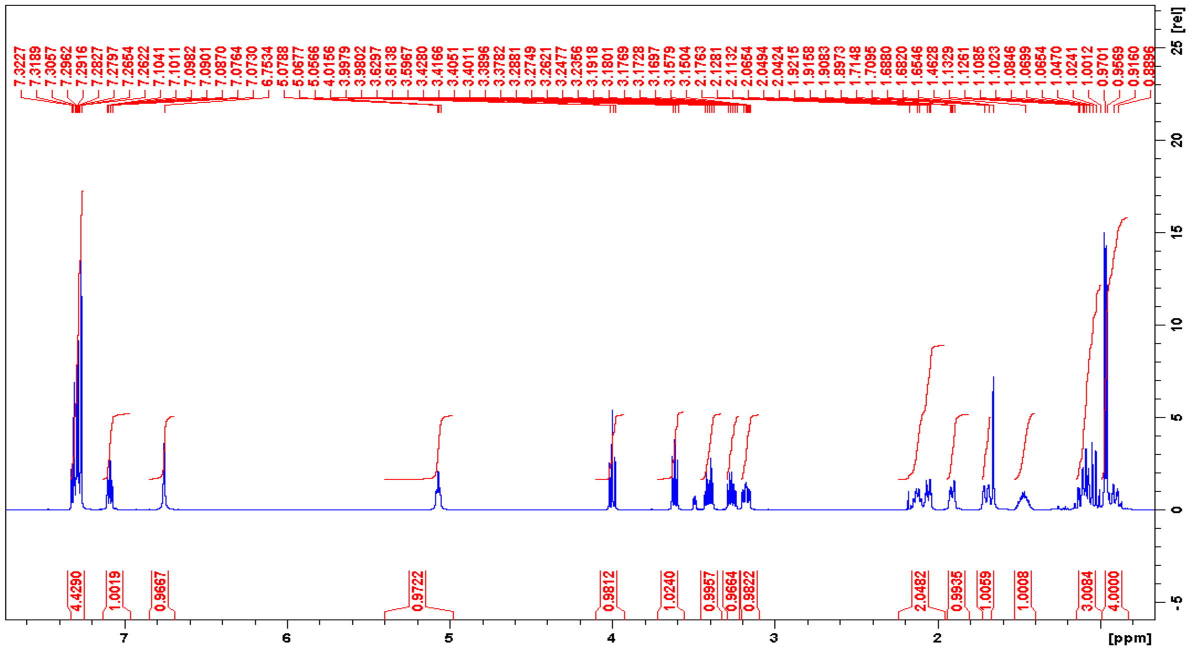

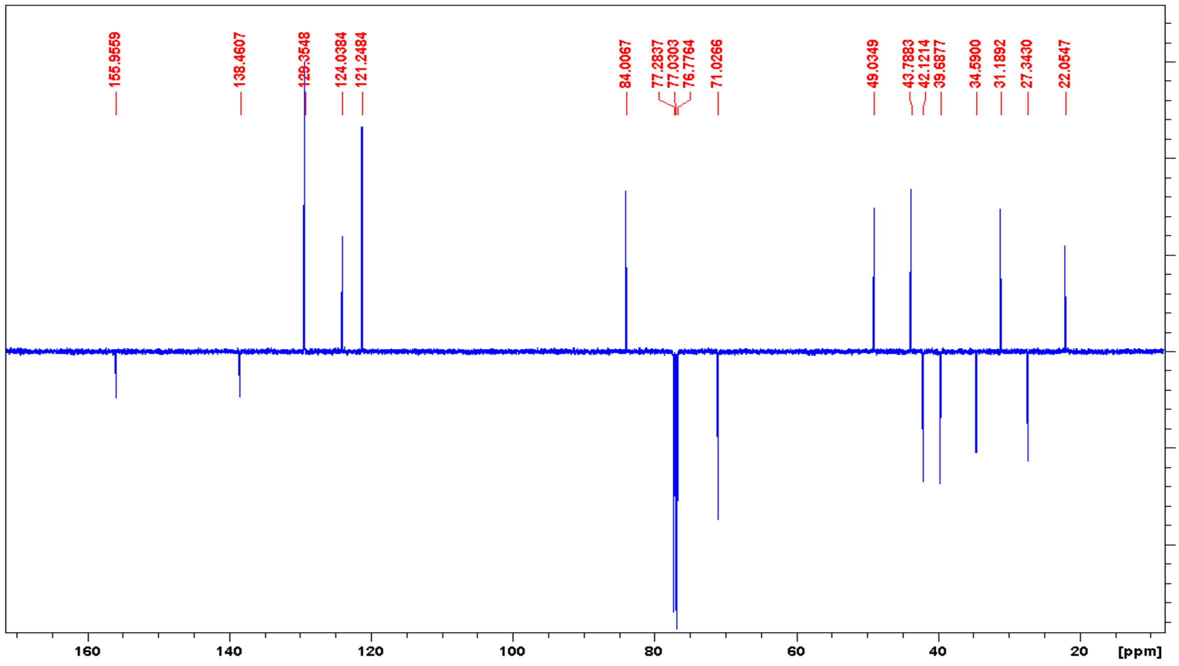

S13: ¹H- and ¹³C-NMR of compound (1*S*,2*R*,5*R*)-5-methyl-2-((*S*)-5-methyl-2-(phenylimino)oxazolidin-5-yl)cyclohexanol (**17c**)

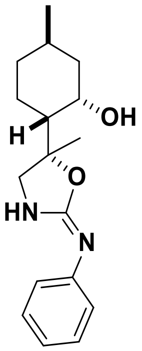

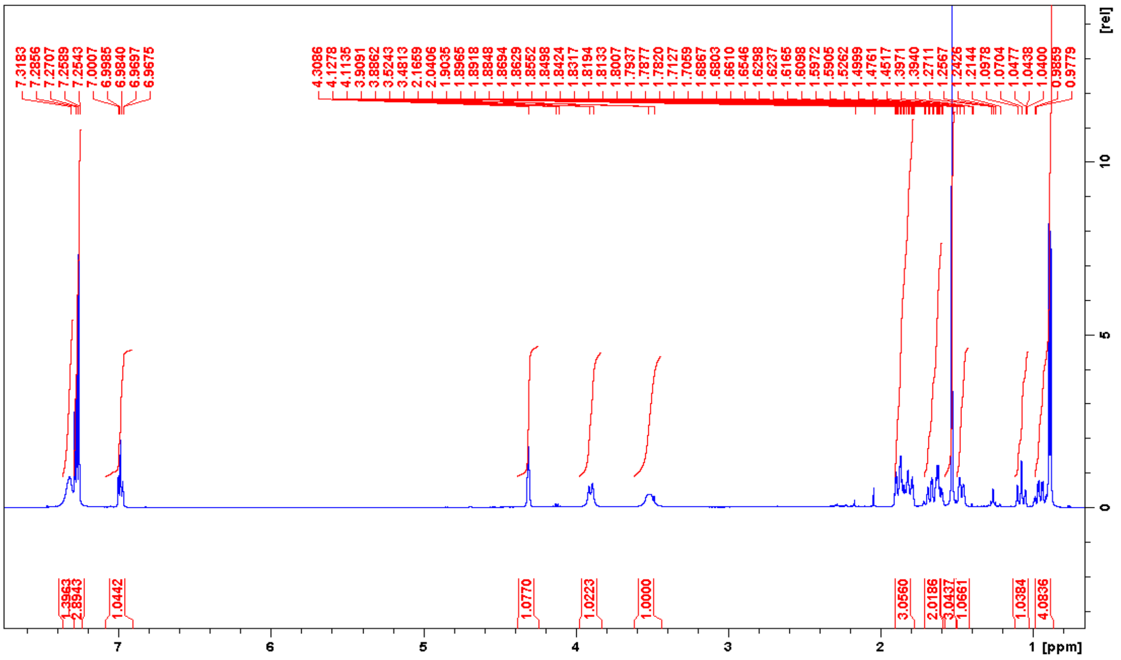

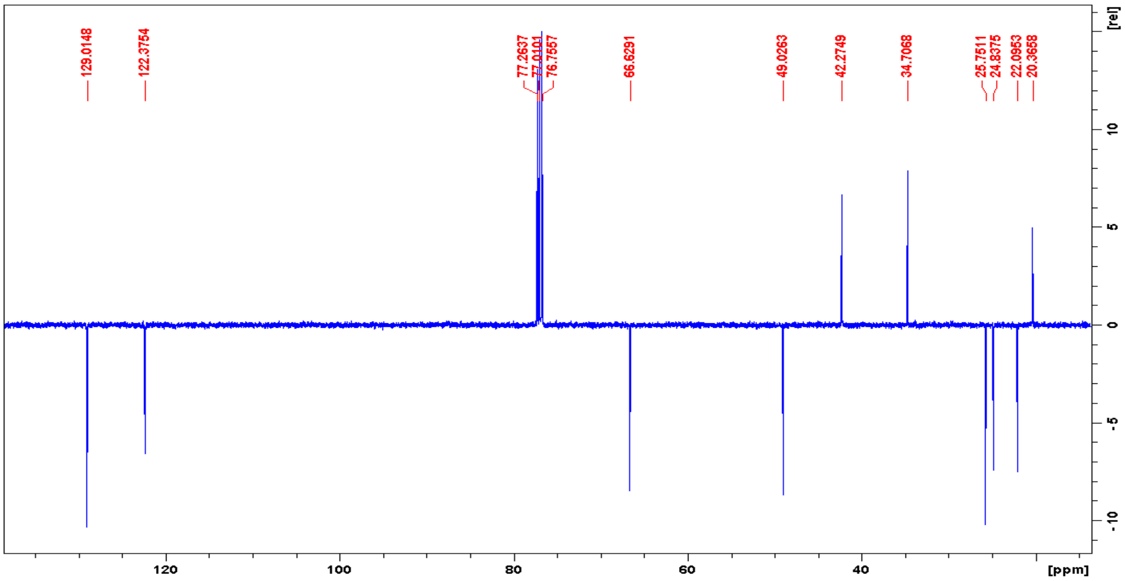

S14: ^{1}H- and ^{13}C-NMR of compound (1*S*,2*S*,5*R*)-5-methyl-2-((*S*)-2-(phenylimino)-1,3-oxazinan-5-yl)cyclohexanol (**17d**)

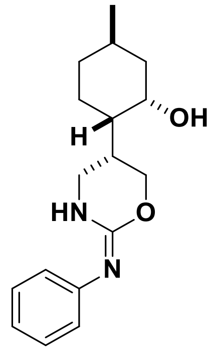

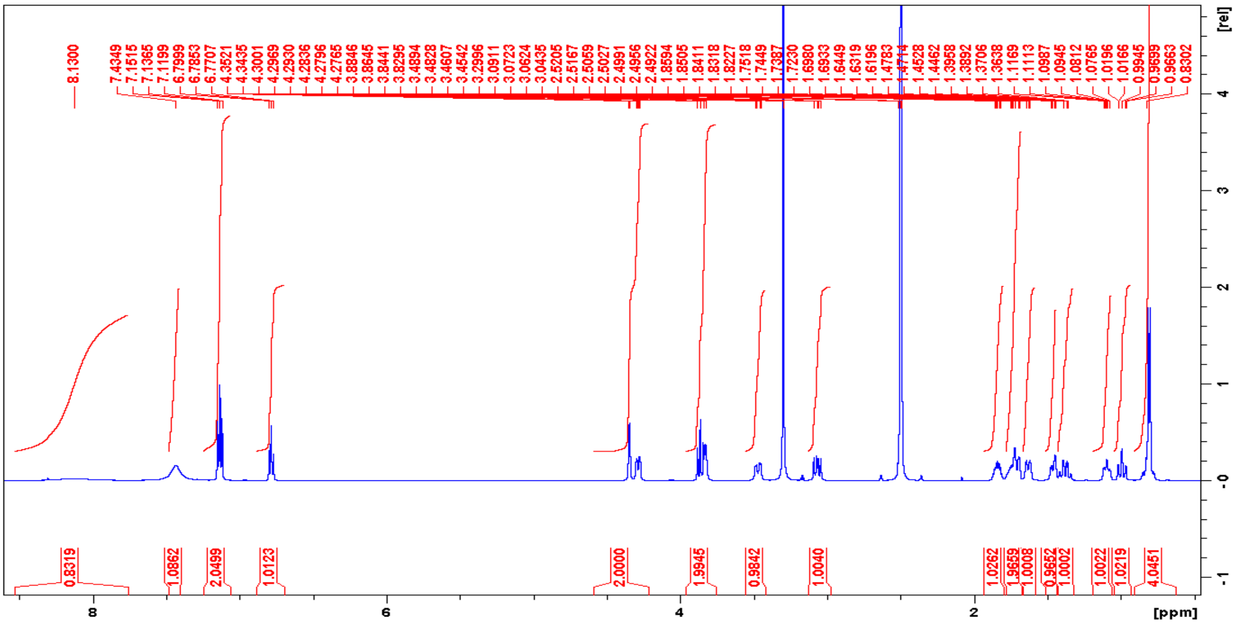

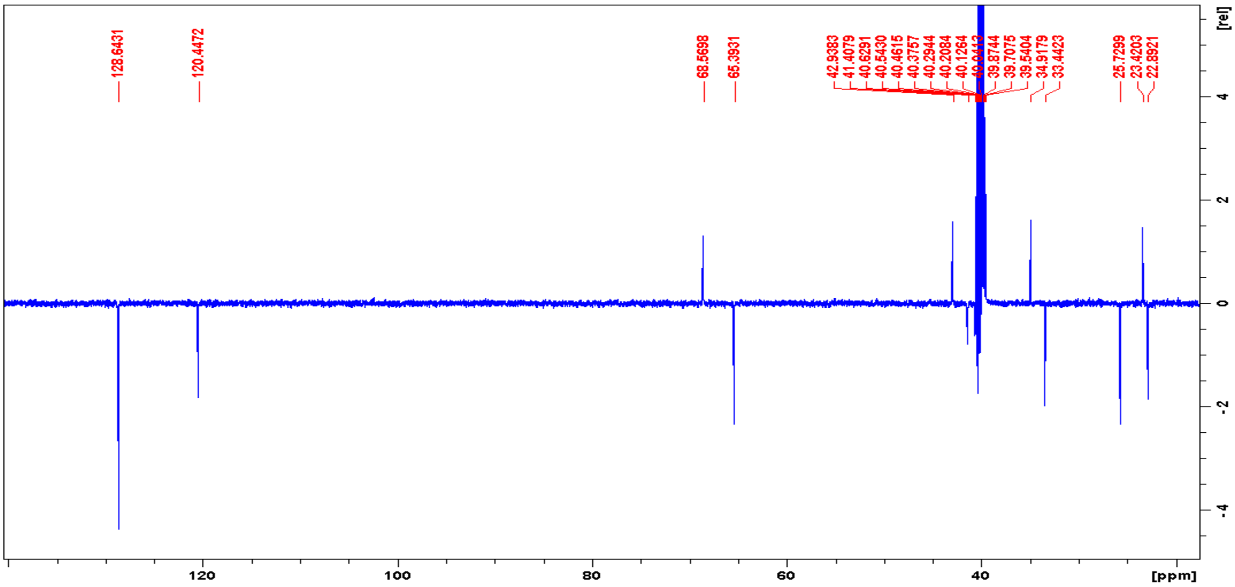

S15: ^{1}H- and ^{13}C-NMR of compound (1*R*,2*R*,5*R*)-2-((*S*)-1-((2-chloro-5-fluoropyrimidin-4-yl)amino)-2-hydroxypropan-2-yl)-5-methylcyclohexanol (**19a**)

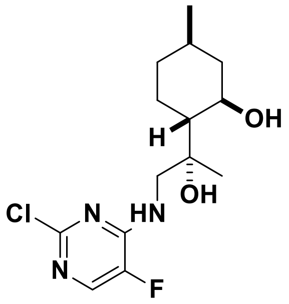

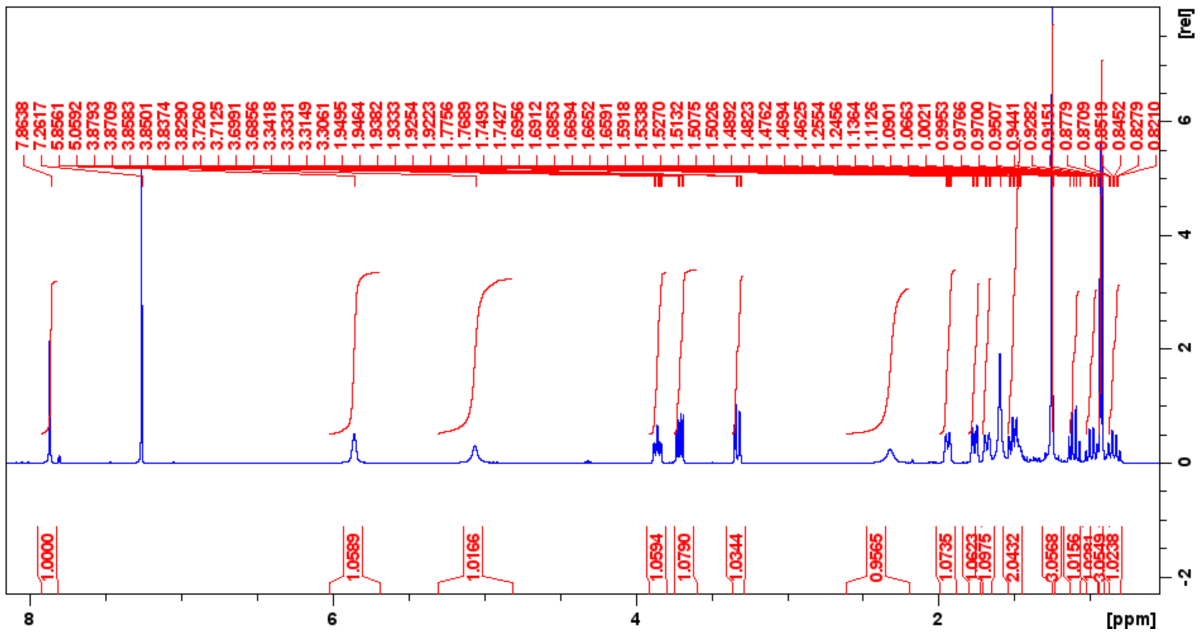

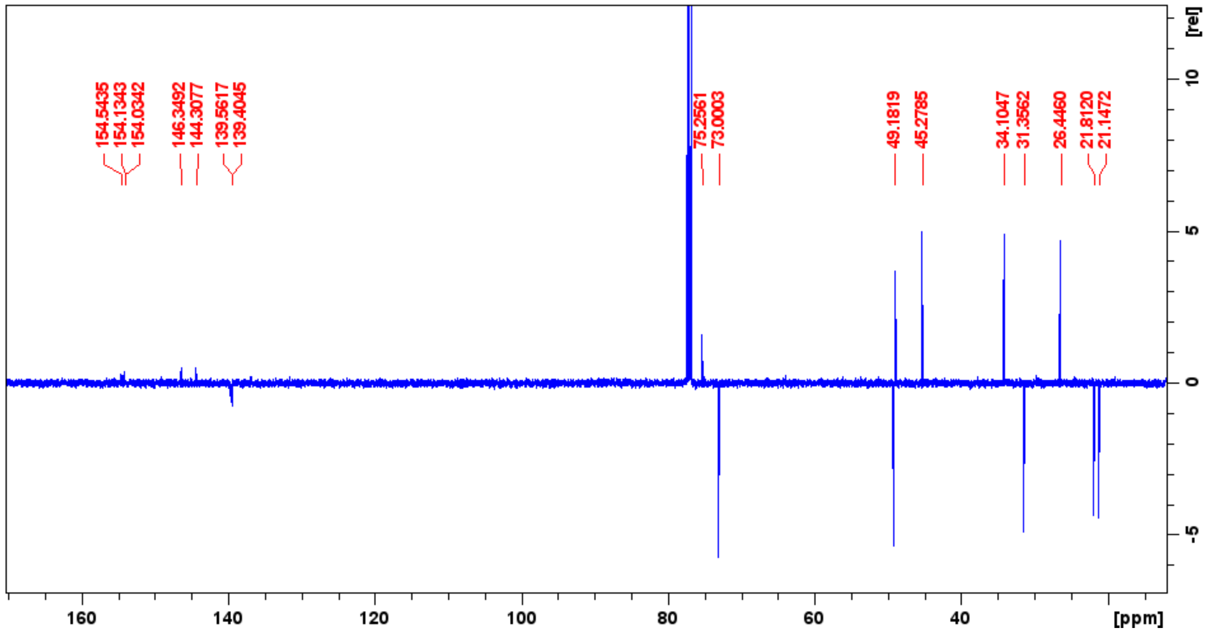

S16: ^{1}H- and ^{13}C-NMR of compound (1*R*,2*R*,5*R*)-2-((*R*)-1-((2-chloro-5-fluoropyrimidin-4-yl)amino)-2-hydroxypropan-2-yl)-5-methylcyclohexanol (**19b**)

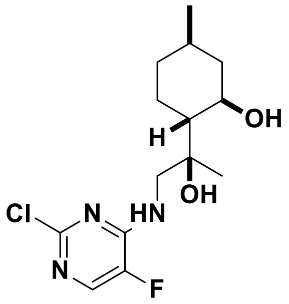

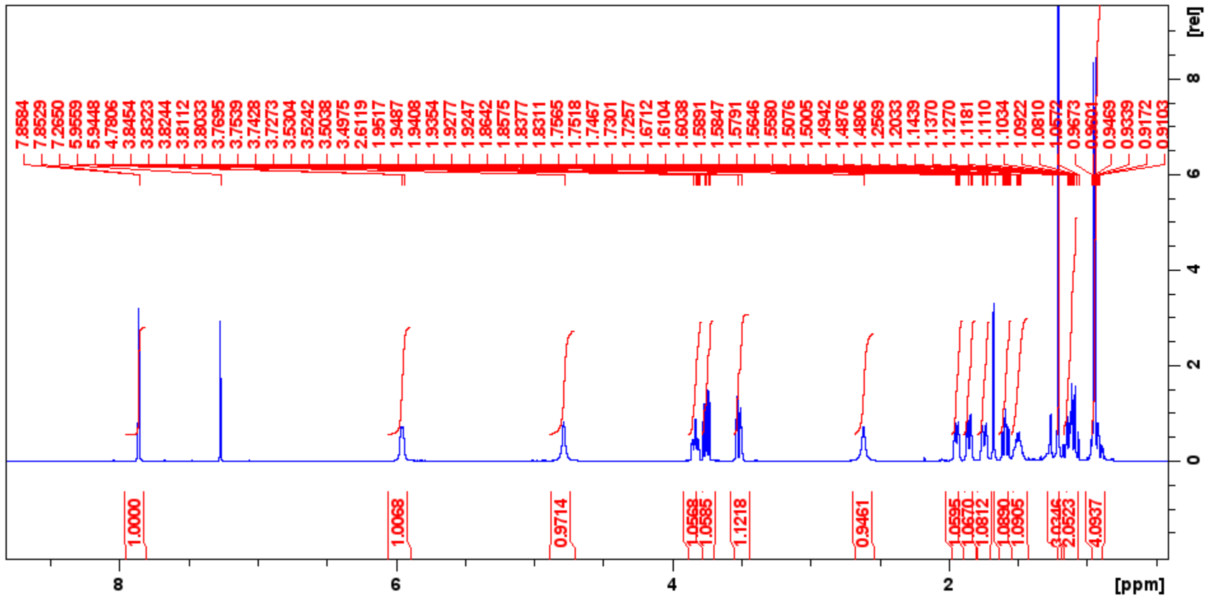

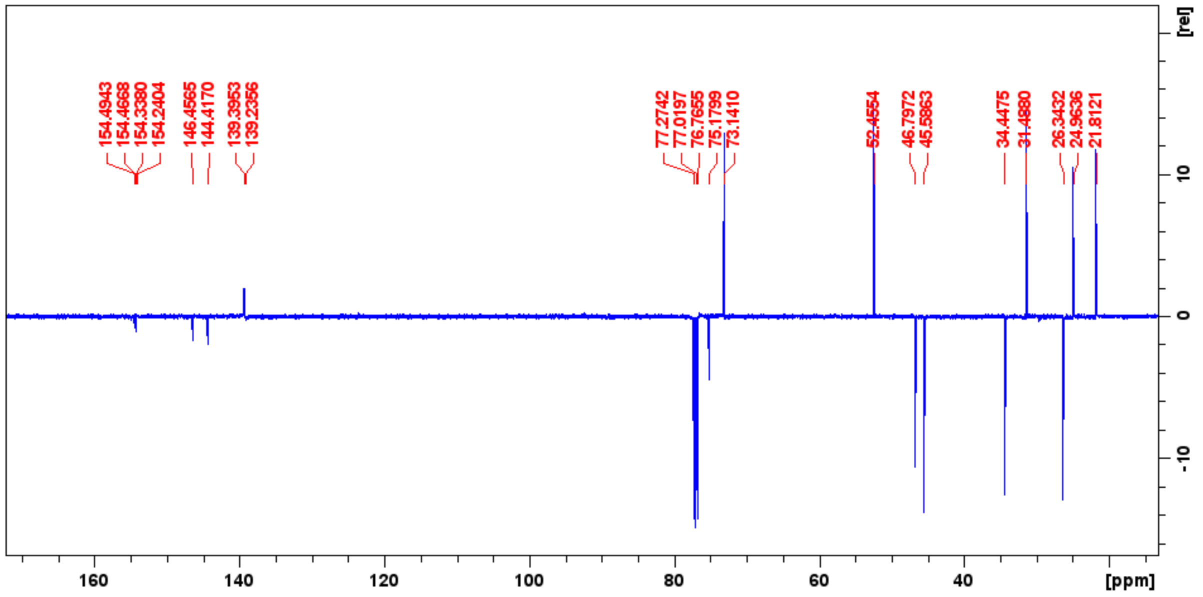

S17: ^{1}H- and ^{13}C-NMR of compound (*S*)-3-((2-chloro-5-fluoropyrimidin-4-yl)amino)-2-((1*R*,2*R*,4*R*)-2-hydroxy-4-methylcyclohexyl)propane-1,2-diol (**19c**)

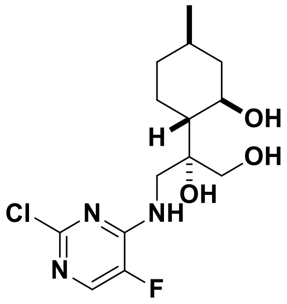

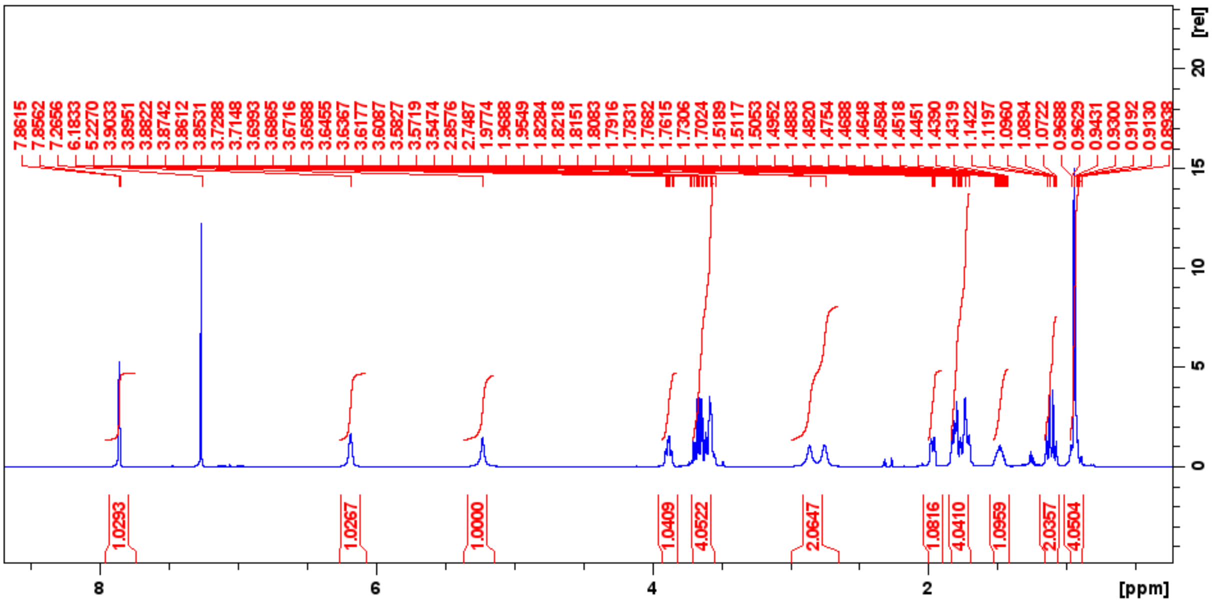

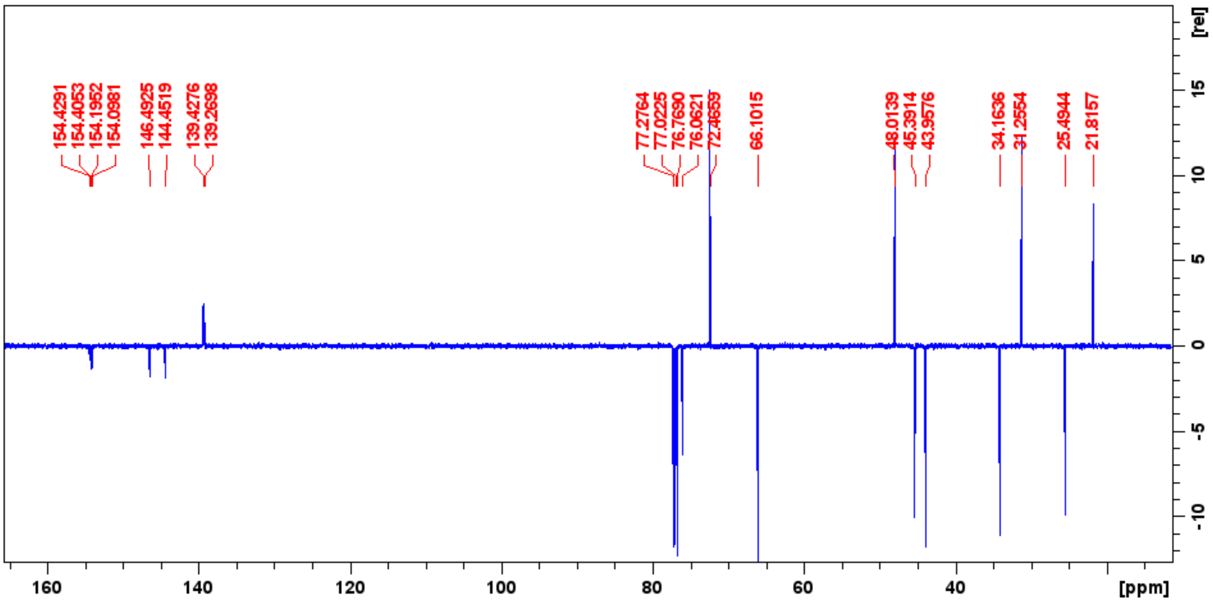

S18: ^{1}H- and ^{13}C-NMR of compound (1*R*,2*S*,5*R*)-2-((*R*)-1-((2-chloro-5-fluoropyrimidin-4-yl)amino)-3-hydroxypropan-2-yl)-5-methylcyclohexanol (**19d**)

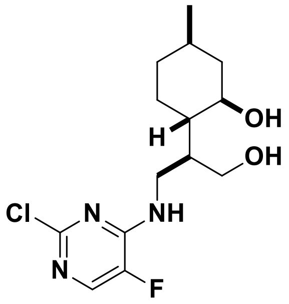

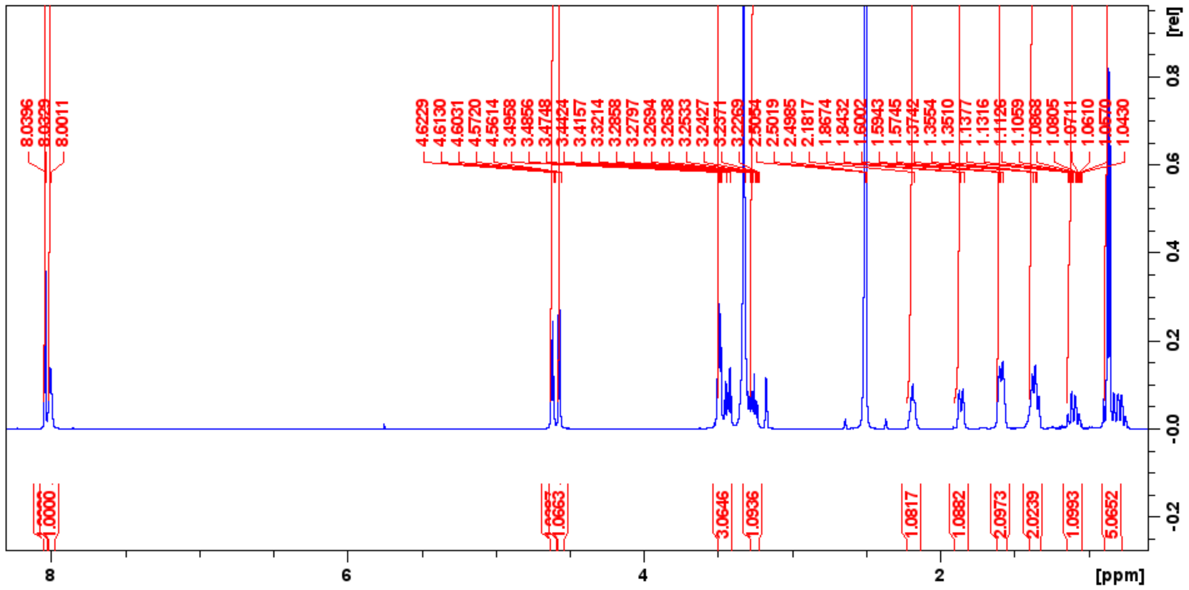

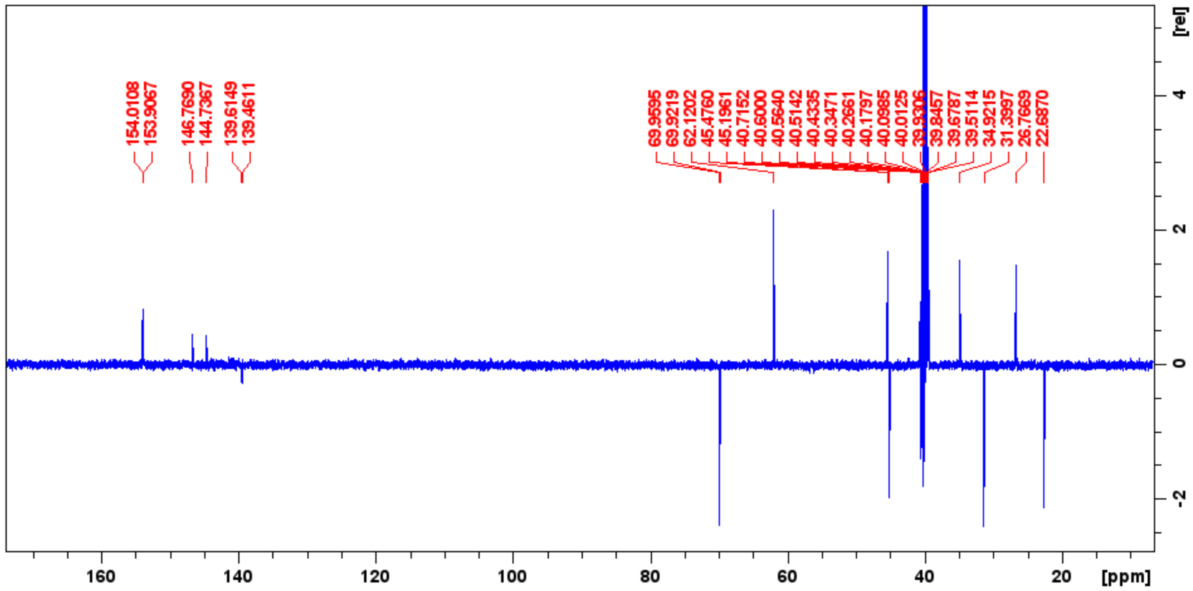

S19: ^{1}H- and ^{13}C-NMR of compound (3*S*,3a*R*,6*R*,7a*R*)-3-(((2-chloro-5-fluoropyrimidin-4-yl)amino)methyl)-6-methyloctahydrobenzofuran-3-ol (**19e**)

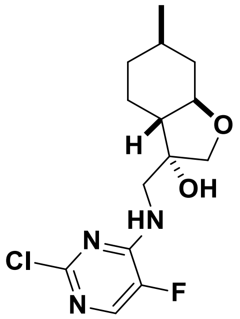

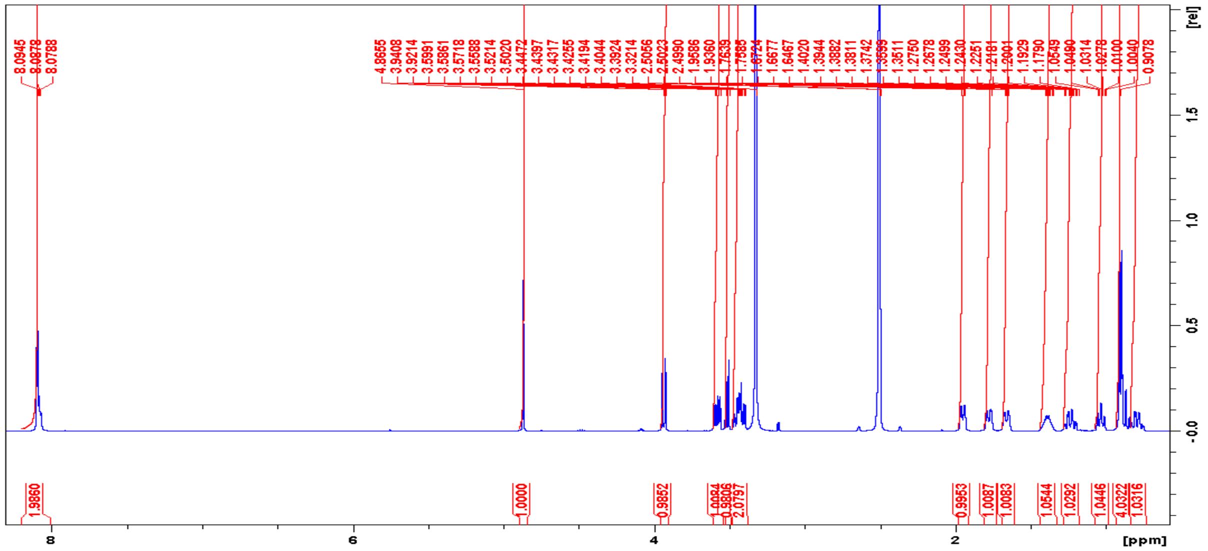

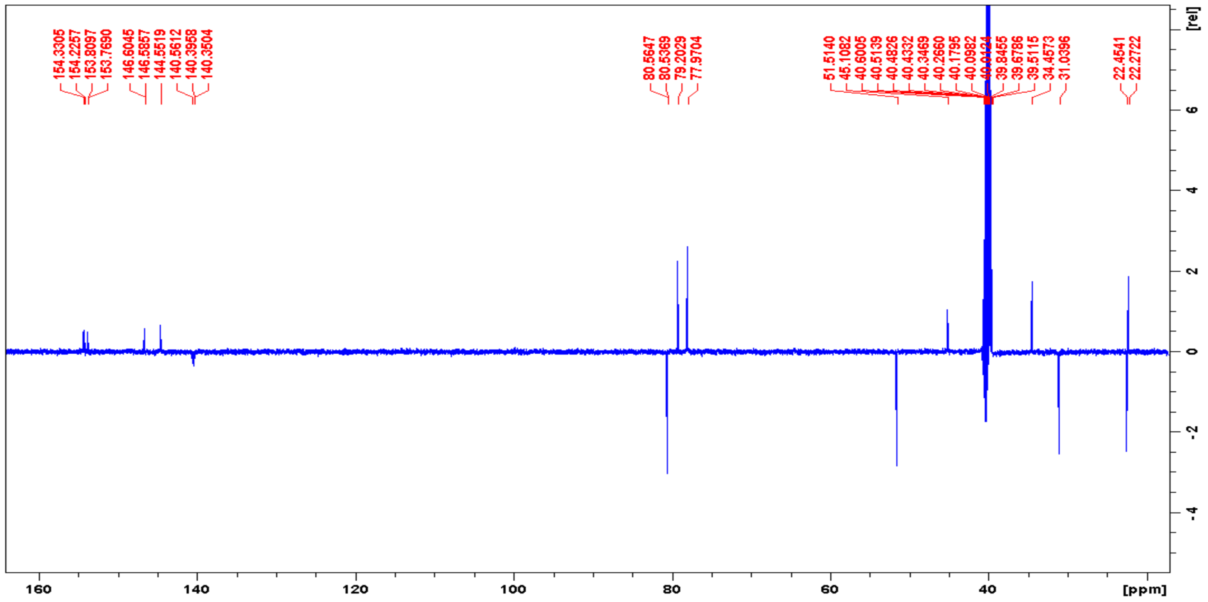

S20: ^{1}H- and ^{13}C-NMR of compound (3*R*,3a*R*,6*R*,7a*R*)-3-(((2-chloro-5-fluoropyrimidin-4-yl)amino)methyl)-6-methyloctahydrobenzofuran-3-ol (**19f**)

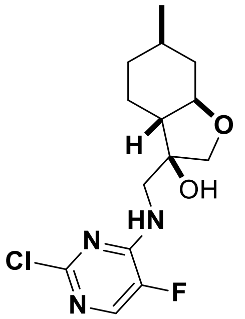

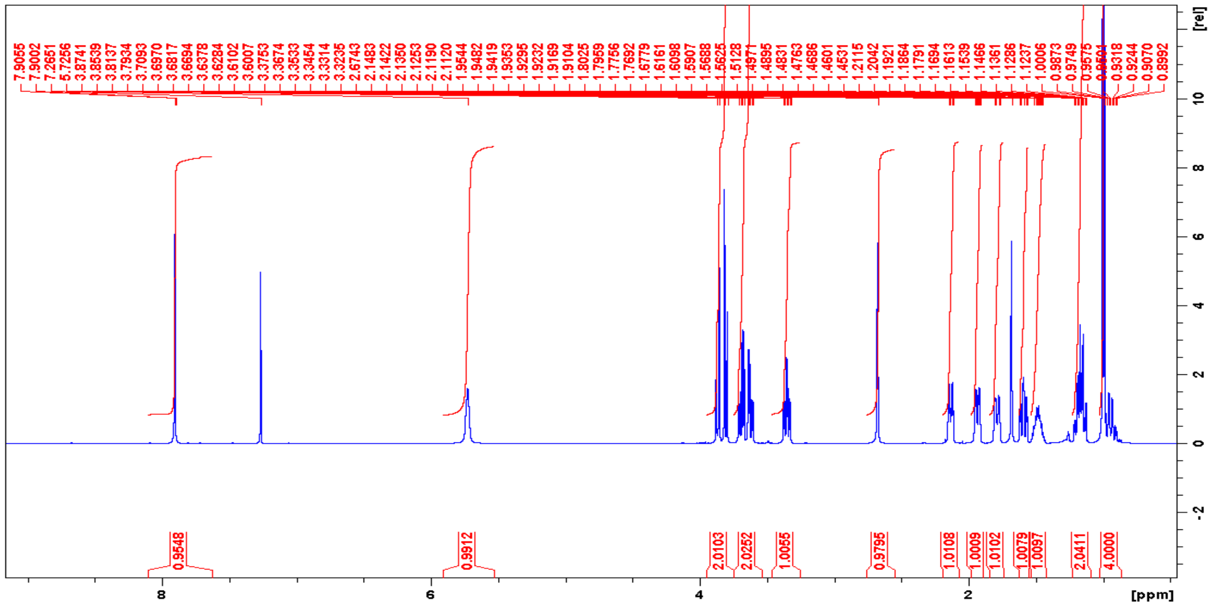

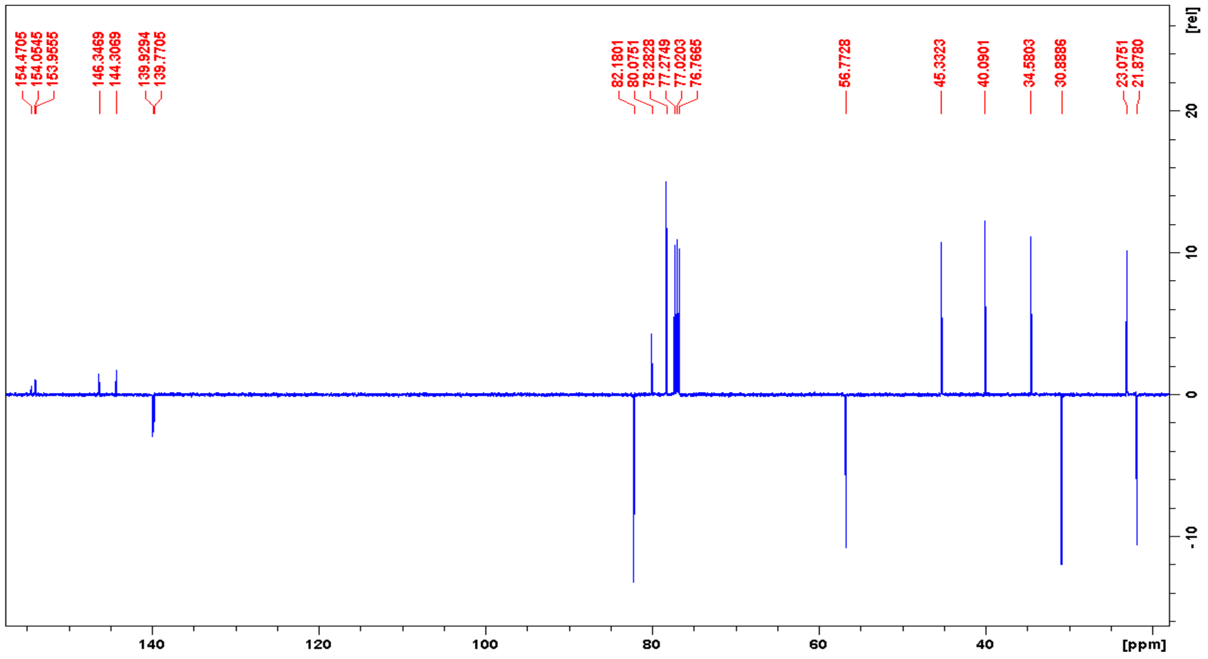

S21: ^{1}H- and ^{13}C-NMR of compound (1*S*,2*R*,5*R*)-2-((*S*)-1-((2-chloro-5-fluoropyrimidin-4-yl)amino)-2-hydroxypropan-2-yl)-5-methylcyclohexanol (**19g**)

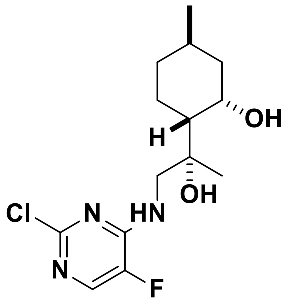

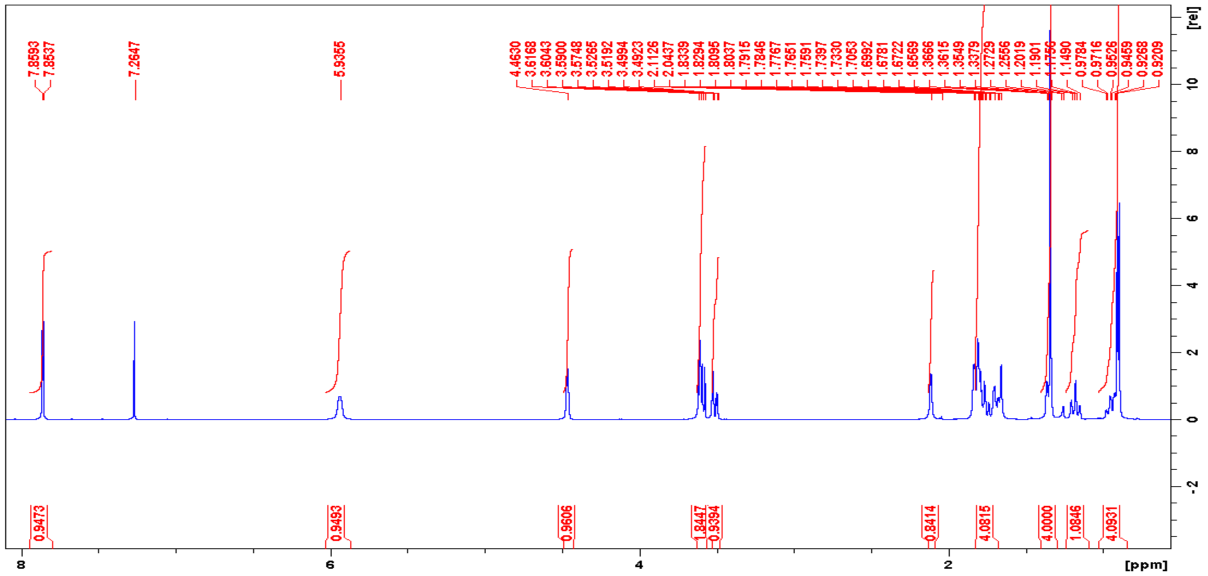

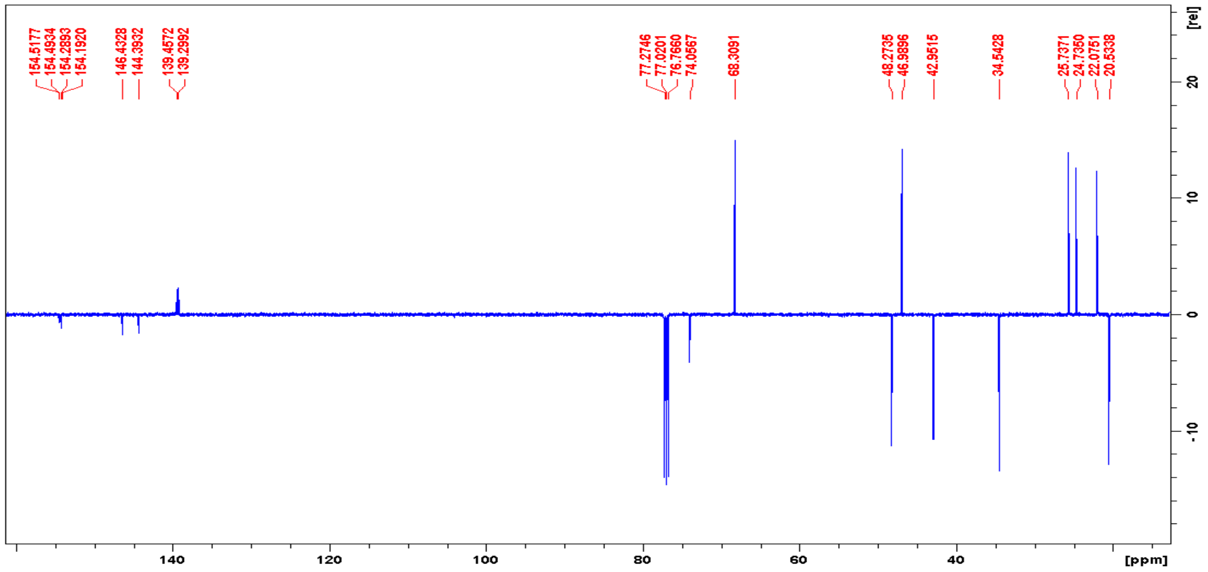

S22: ^{1}H- and ^{13}C-NMR of compound (1*S*,2*S*,5*R*)-2-((*S*)-1-((2-chloro-5-fluoropyrimidin-4-yl)amino)-3-hydroxypropan-2-yl)-5-methylcyclohexanol (**19h**)

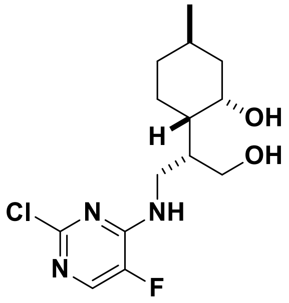

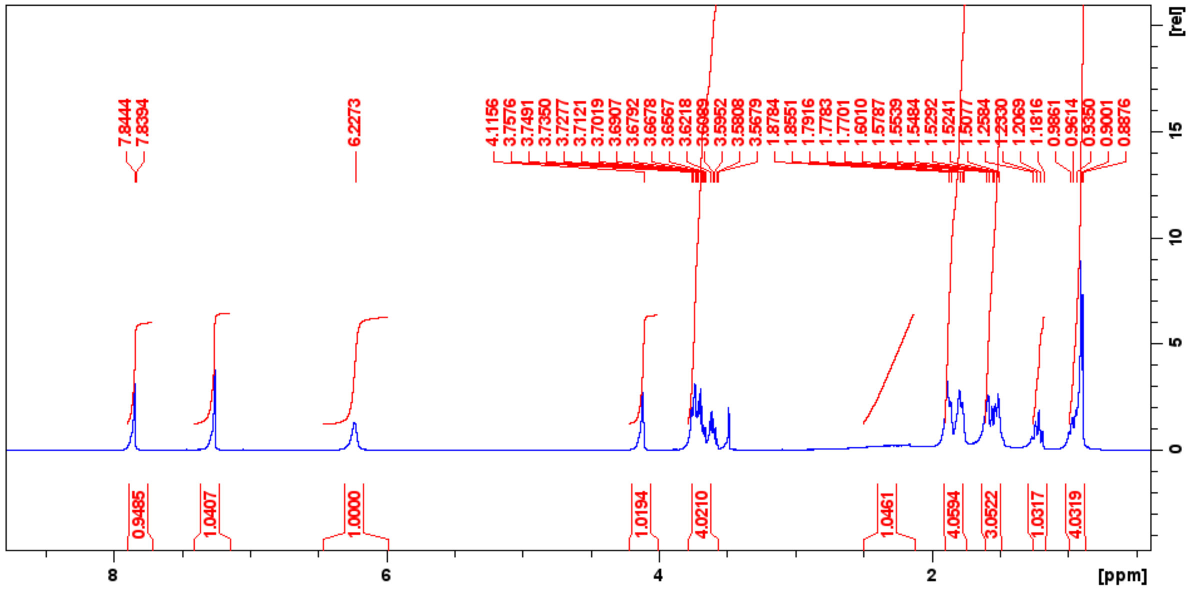

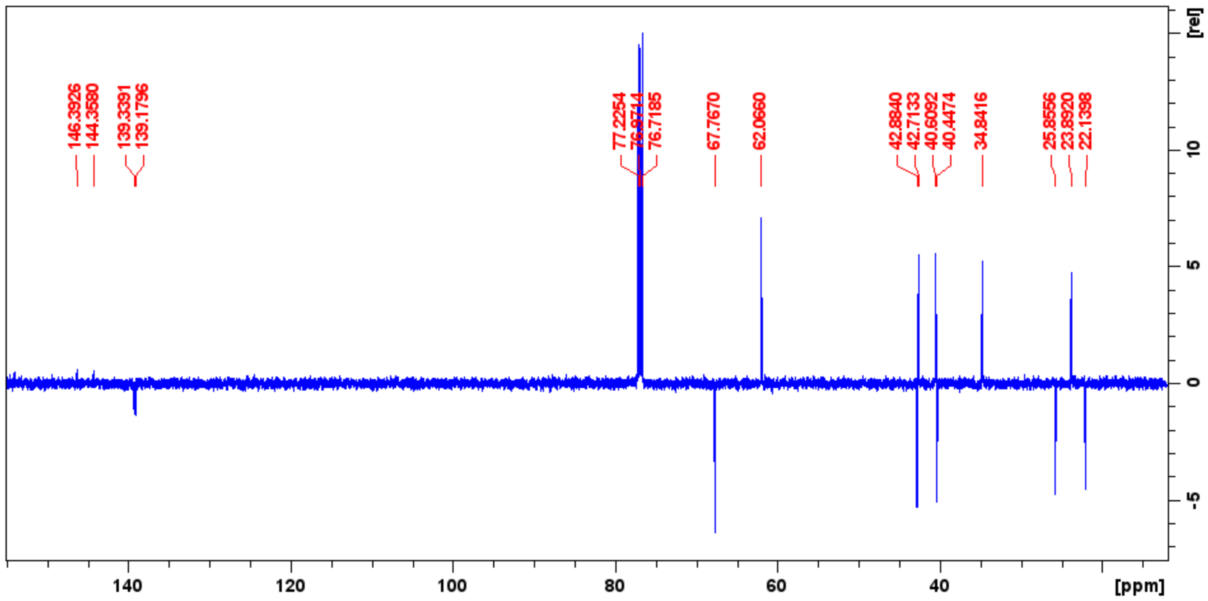

S23: ^{1}H- and ^{13}C-NMR of compound (3*R*,3a*R*,6*R*,7a*S*)-3-(((2-chloro-5-fluoropyrimidin-4-yl)amino)methyl)-6-methyloctahydrobenzofuran-3-ol (**19i**)

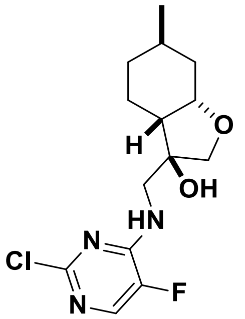

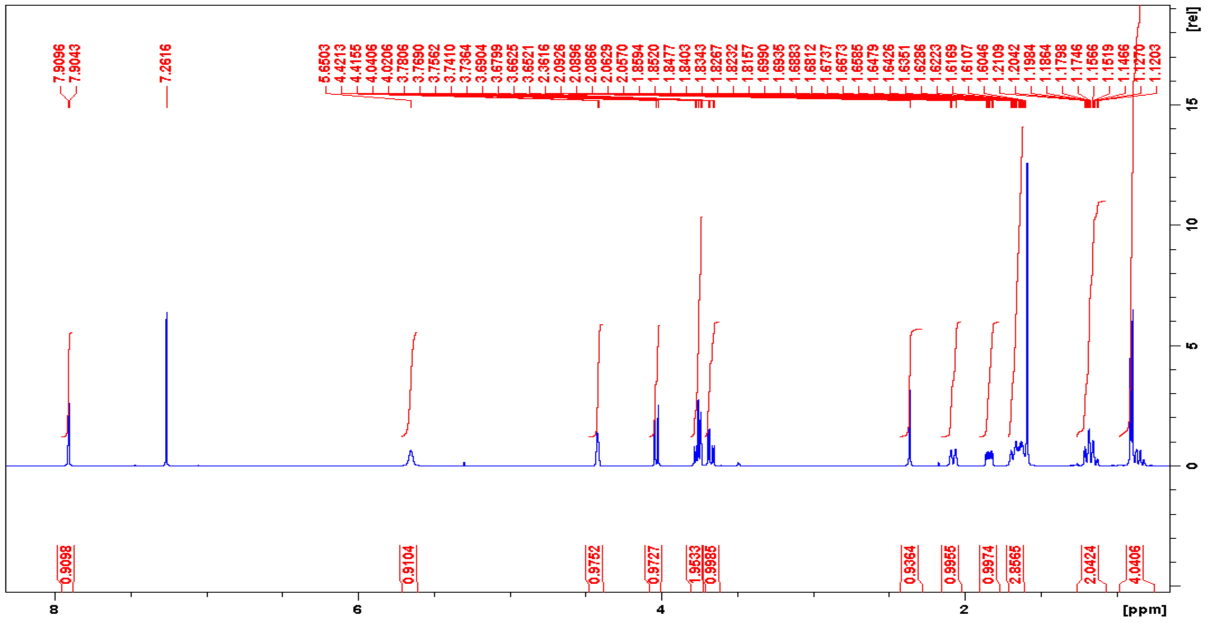

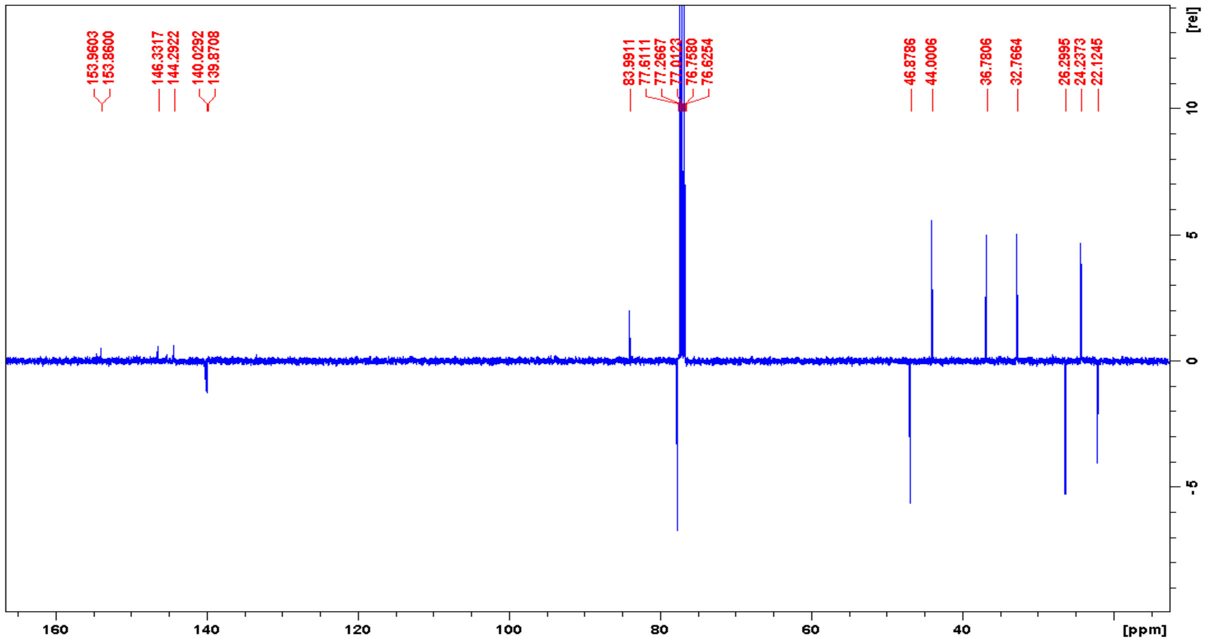

S24: ^{1}H- and ^{13}C-NMR of compound *(1R,2R,5R)-2-((S)-1-((5-fluoro-2-((4-(trifluoromethyl)phenyl)amino)pyrimidin-4-yl)amino)-2-hydroxypropan-2-yl)-5-methylcyclohexan-1-ol hydrochloride* (**20a**)

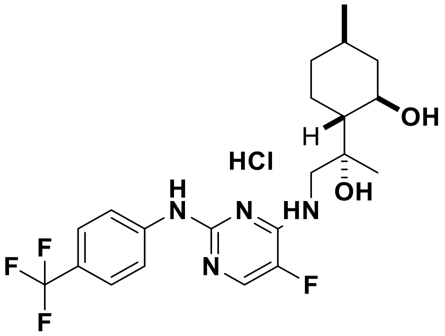

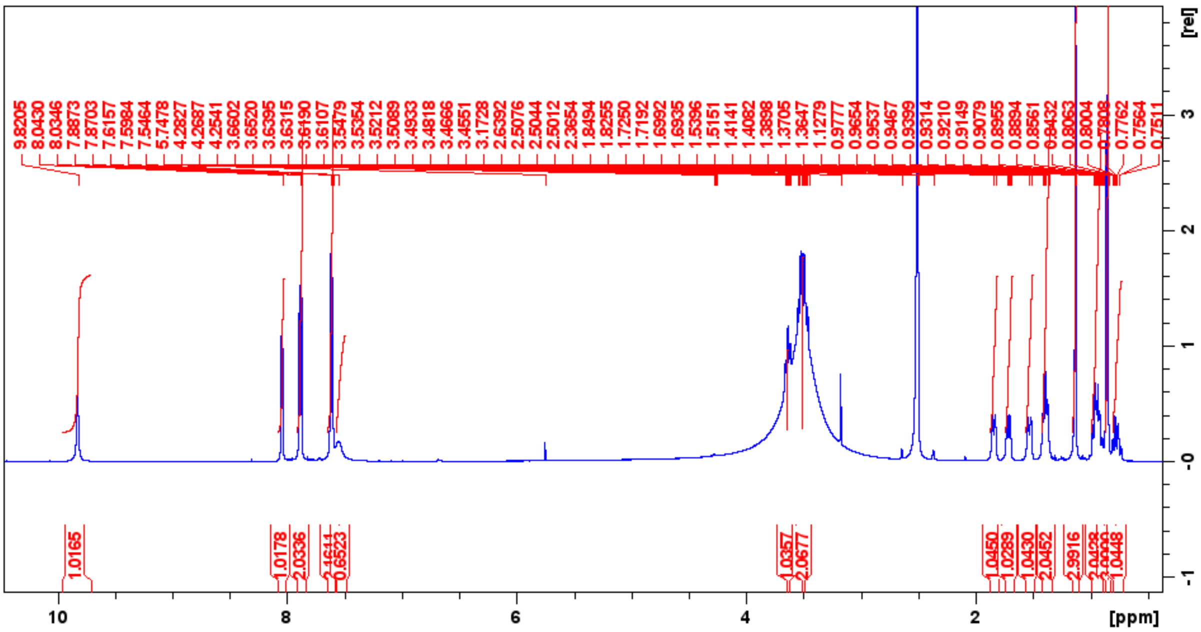

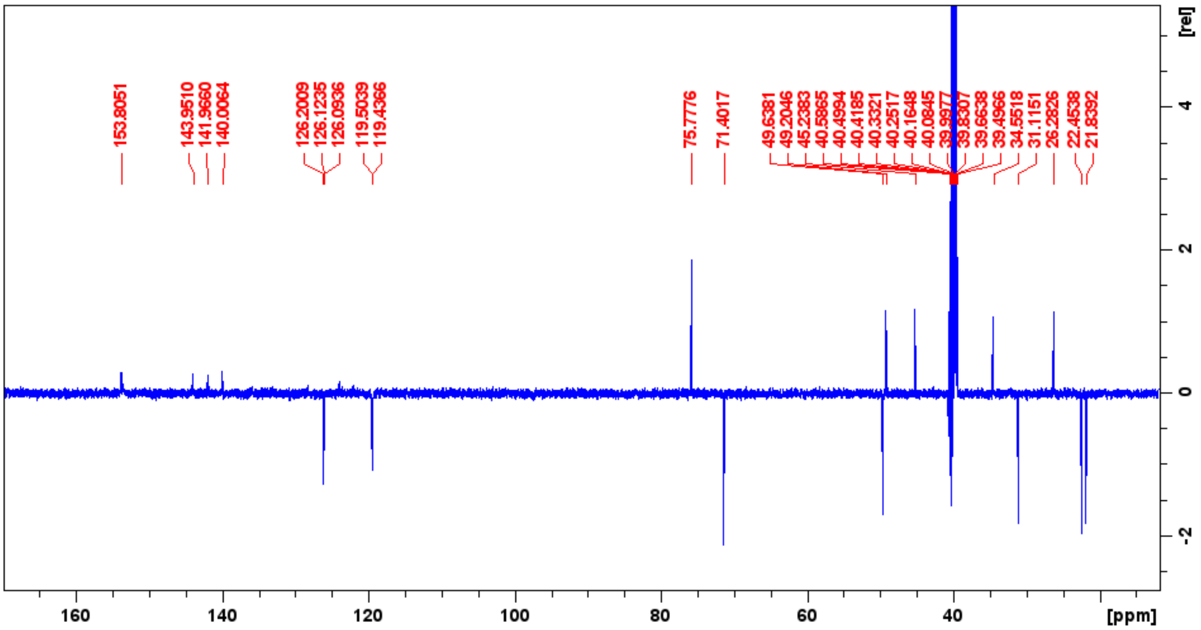

S25: ^{1}H- and ^{13}C-NMR of compound *(1R,2R,5R)-2-((R)-1-((5-fluoro-2-((4-(trifluoromethyl)phenyl)amino)pyrimidin-4-yl)amino)-2-hydroxypropan-2-yl)-5-methylcyclohexan-1-ol hydrochloride* (**20b**)

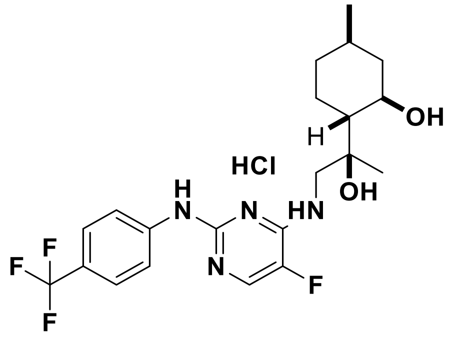

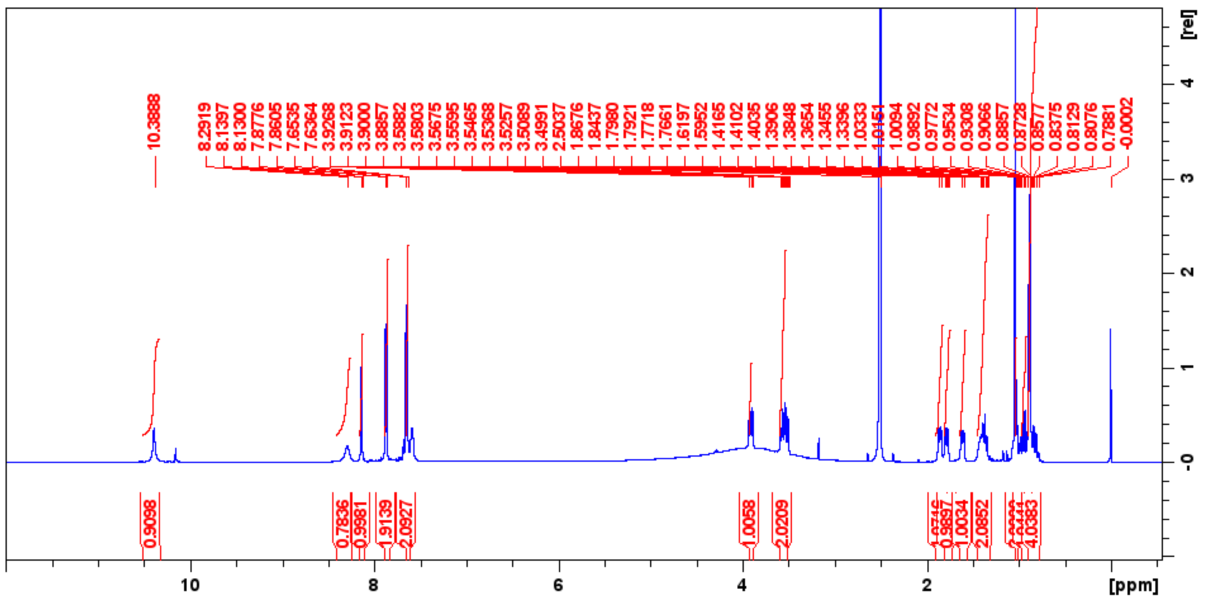

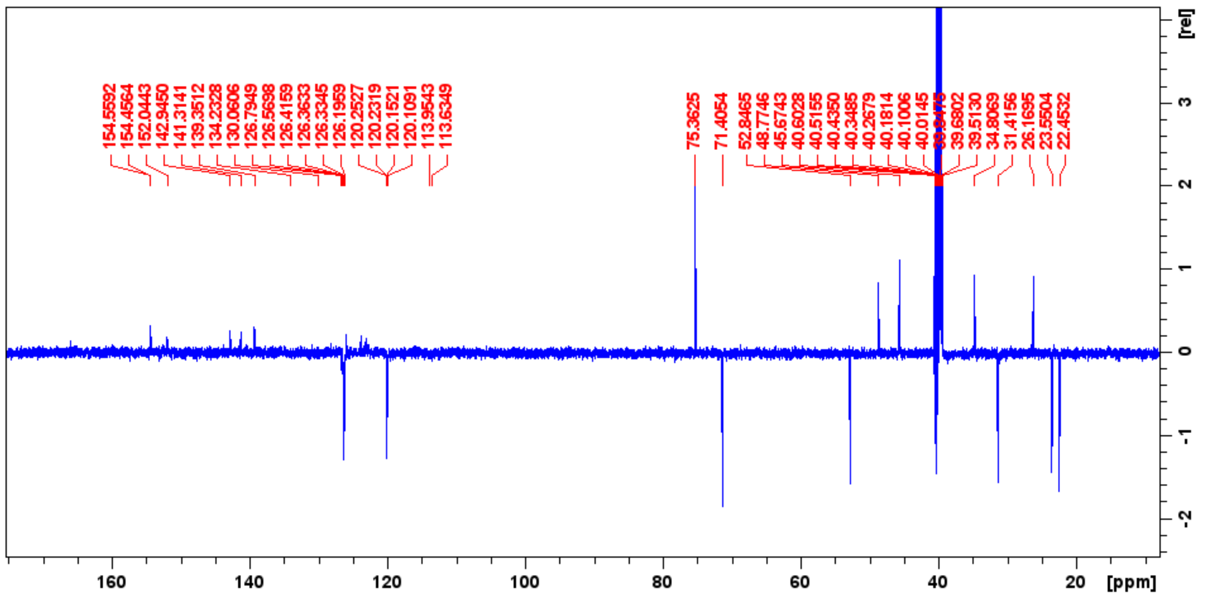

S26: ^{1}H- and ^{13}C-NMR of compound *(S)-3-((5-fluoro-2-((4-(trifluoromethyl)phenyl)amino)pyrimidin-4-yl)amino)-2-((1R,2R,4R)-2-hydroxy-4-methylcyclohexyl)propane-1,2-diol hydrochloride* (**20c**)

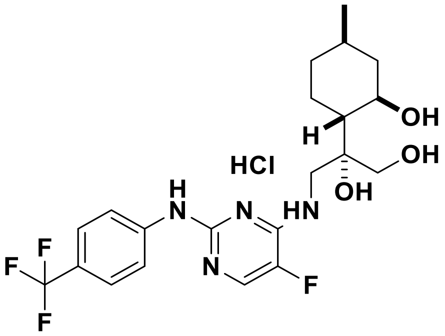

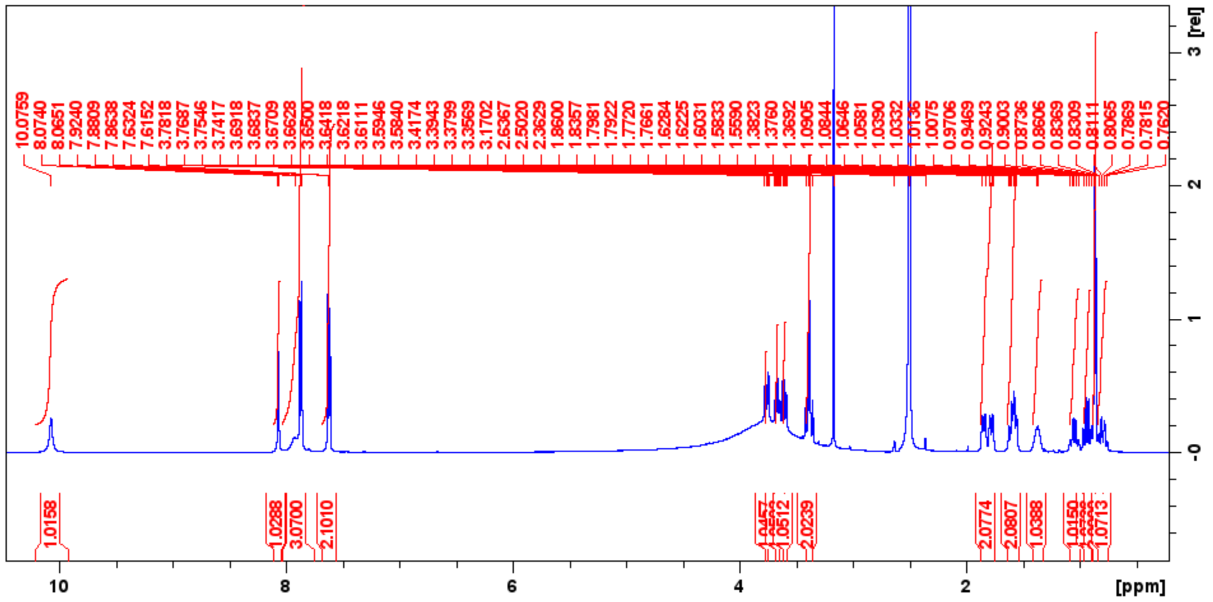

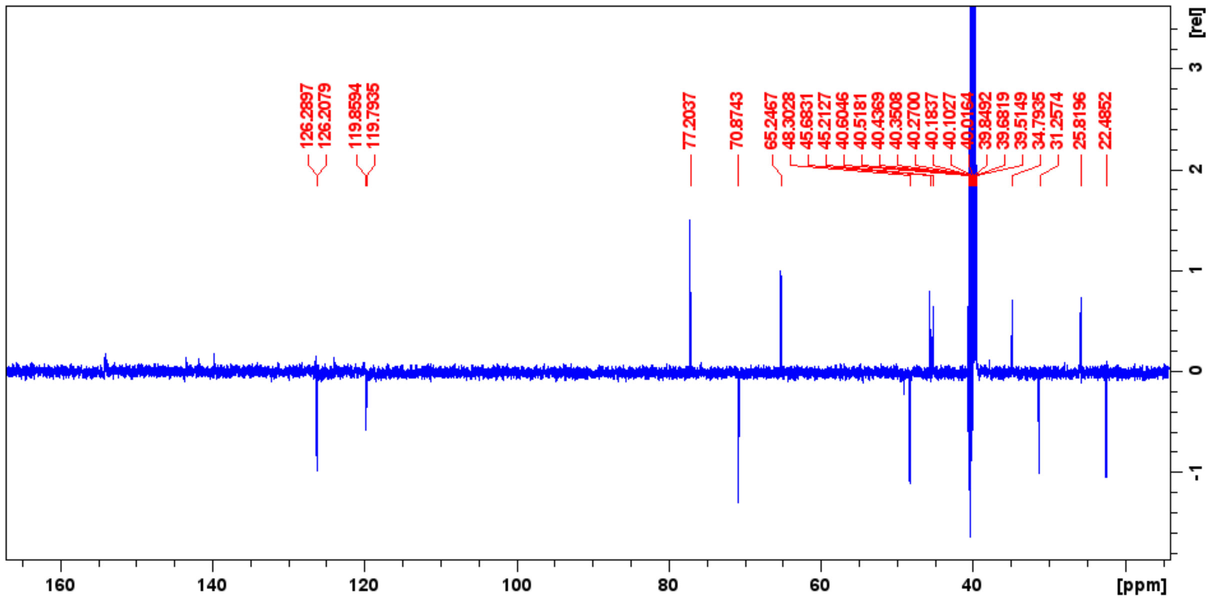

S27: ¹H- and ¹³C-NMR of compound *(1R,2S,5R)-2-((R)-1-((5-fluoro-2-((4-(trifluoromethyl)phenyl)amino)pyrimidin-4-yl)amino)-3-hydroxypropan-2-yl)-5-methylcyclohexan-1-ol hydrochloride* (**20d**)

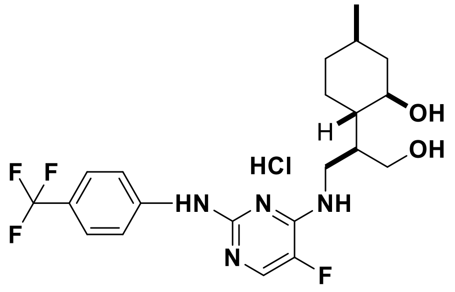

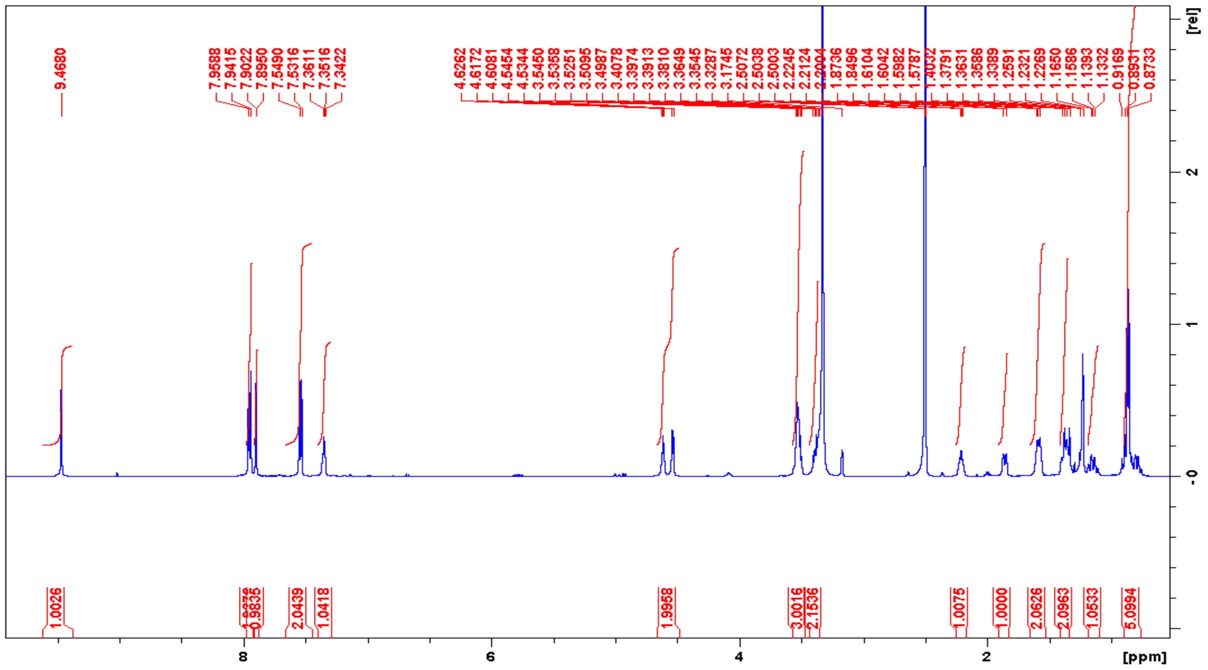

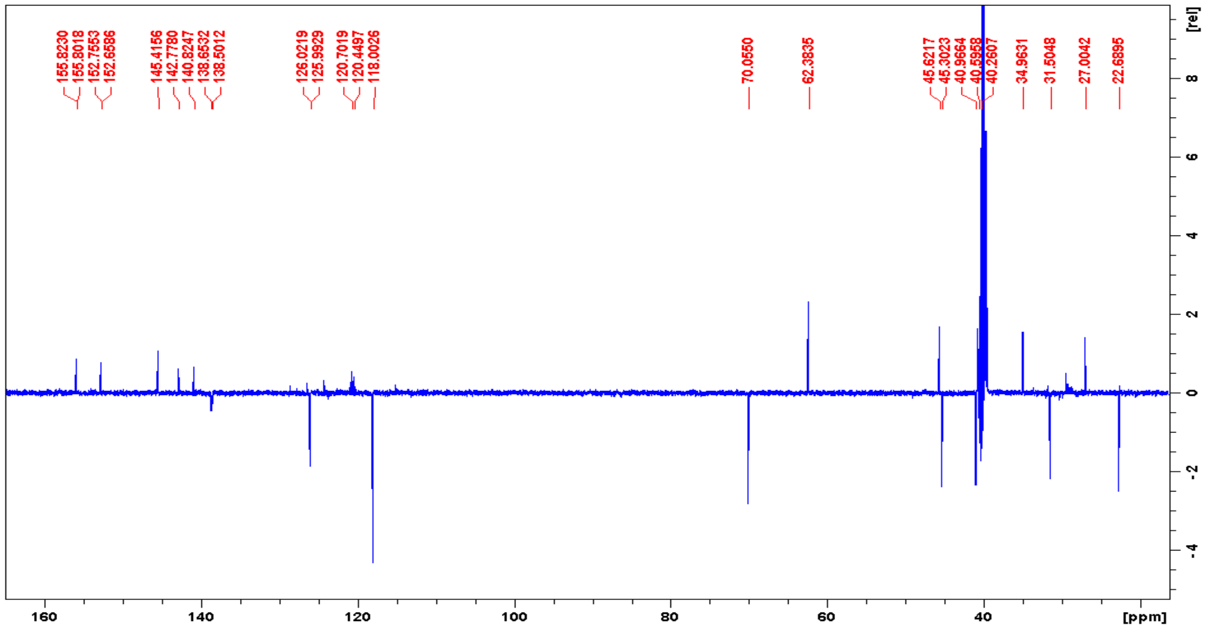

S28: ^{1}H- and ^{13}C-NMR of compound *(3S,3aR,6R,7aR)-3-(((5-fluoro-2-((4-(trifluoromethyl)phenyl)amino)pyrimidin-4-yl)amino)methyl)-6-methyloctahydrobenzofuran-3-ol hydrochloride* (**20e**)

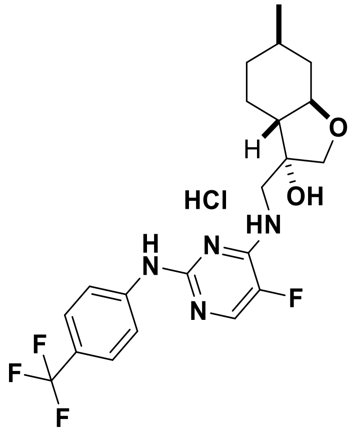

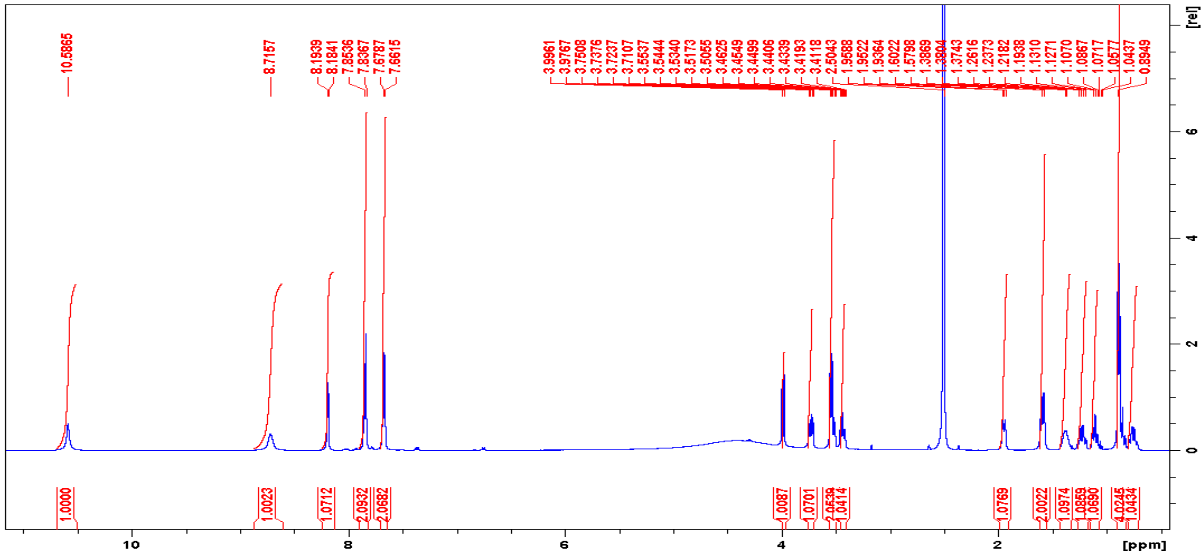

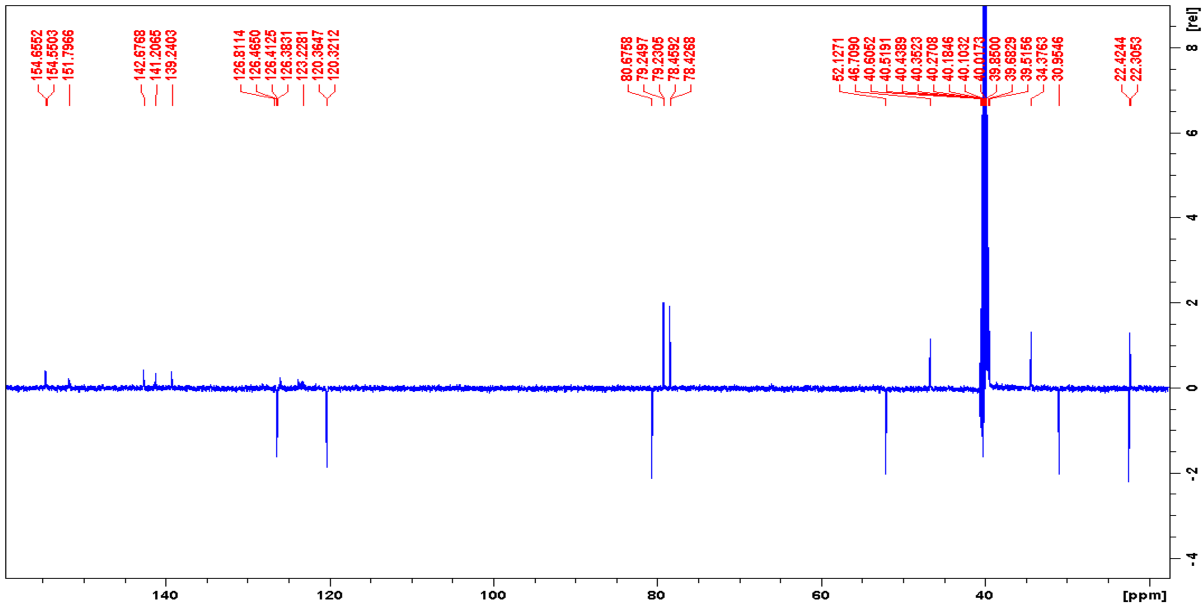

S29: ^{1}H- and ^{13}C-NMR of compound *(3R,3aR,6R,7aR)-3-(((5-fluoro-2-((4-(trifluoromethyl)phenyl)amino)pyrimidin-4-yl)amino)methyl)-6-methyloctahydrobenzofuran-3-ol hydrochloride* (**20f**)

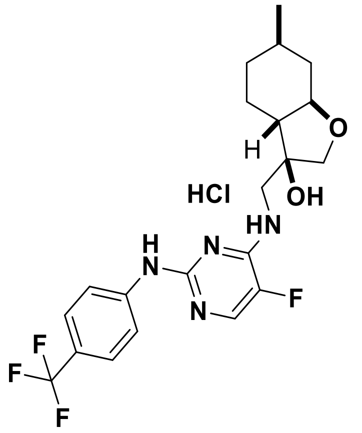

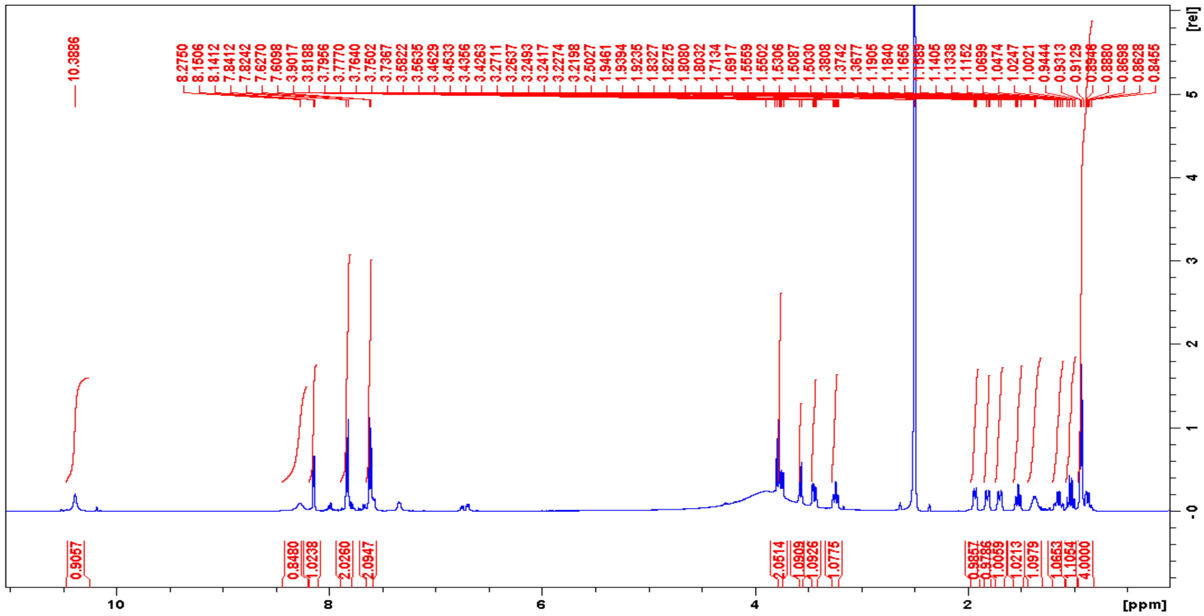

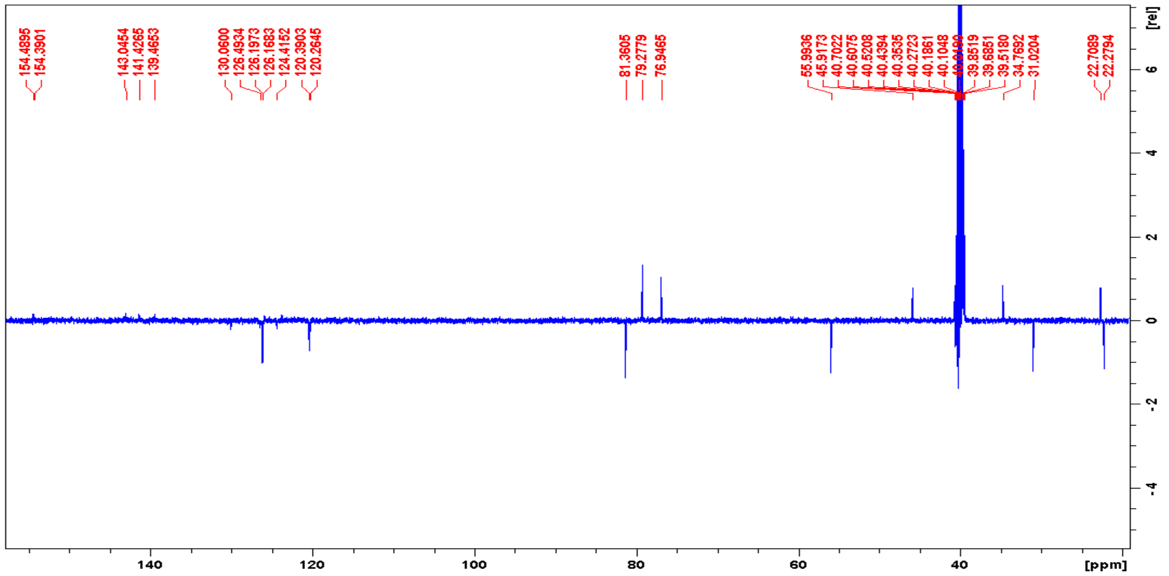

S30: ^{1}H- and ^{13}C-NMR of compound *(1S,2R,5R)-2-((S)-1-((5-fluoro-2-((4-(trifluoromethyl)phenyl)amino)pyrimidin-4-yl)amino)-2-hydroxypropan-2-yl)-5-methylcyclohexan-1-ol hydrochloride* (**20g**)

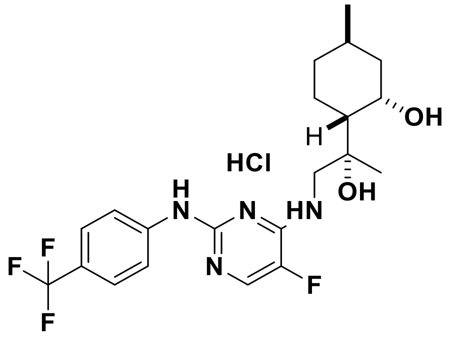

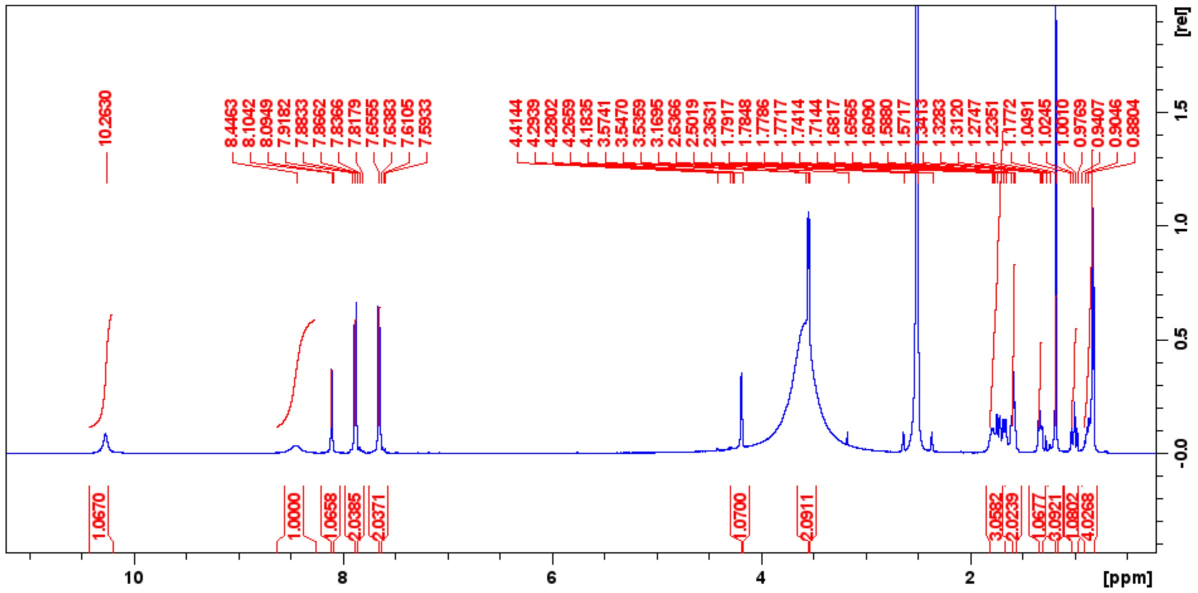

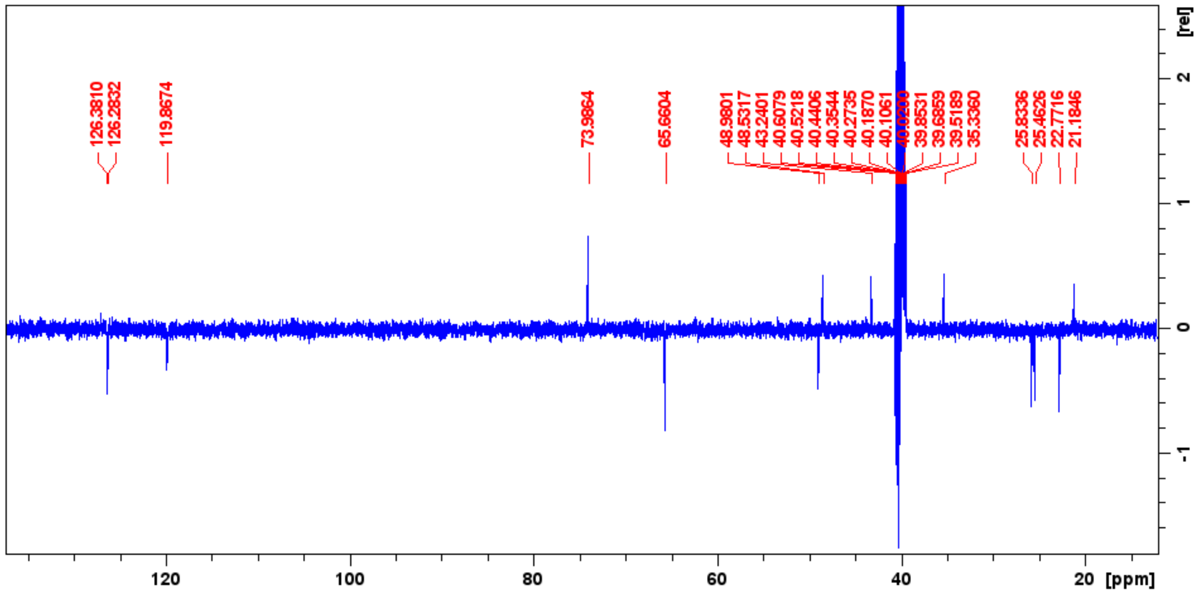

S31: ^{1}H- and ^{13}C-NMR of compound *(1S,2S,5R)-2-((S)-1-((5-fluoro-2-((4-(trifluoromethyl)phenyl)amino)pyrimidin-4-yl)amino)-3-hydroxypropan-2-yl)-5-methylcyclohexan-1-ol hydrochloride* (**20h**)

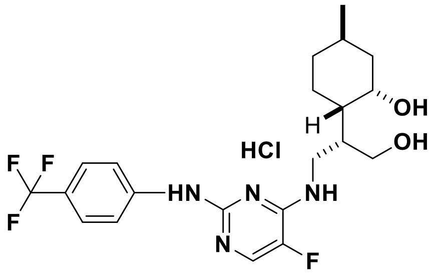

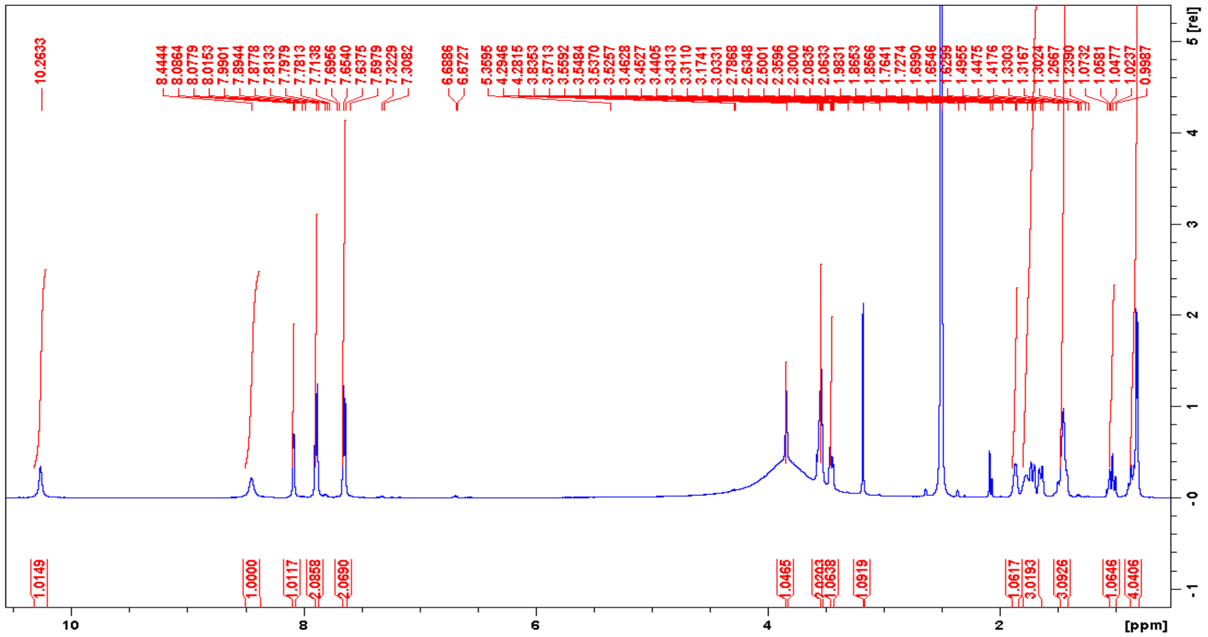

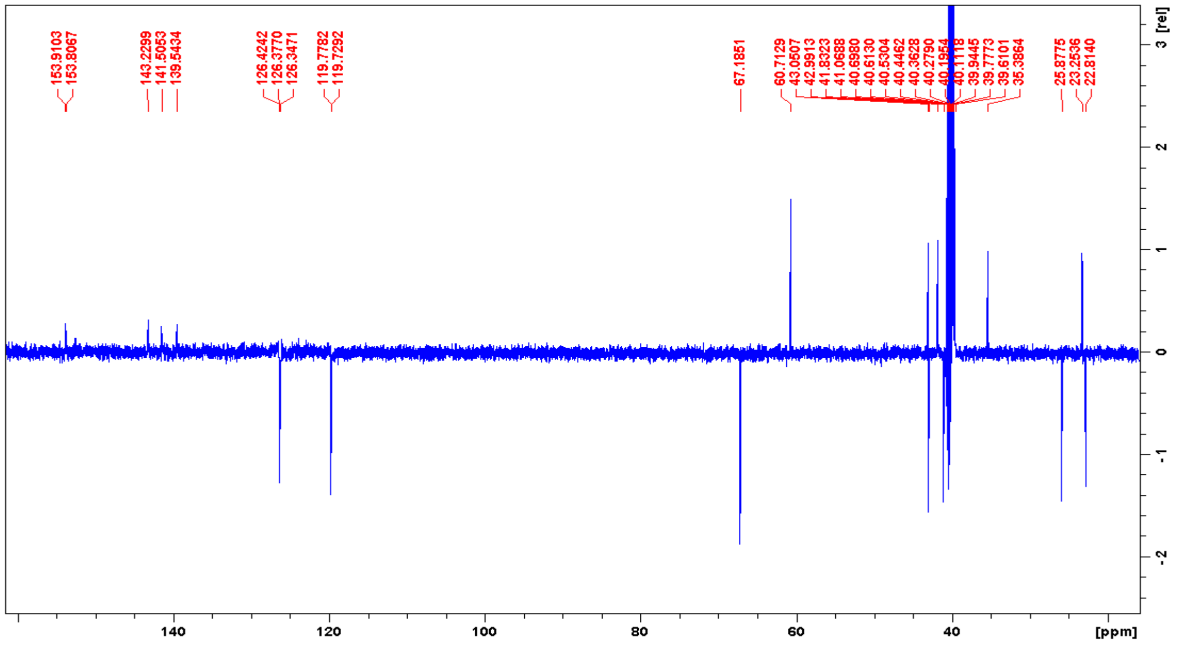

S32: ^{1}H- and ^{13}C-NMR of compound *(3R,3aR,6R,7aS)-3-(((5-fluoro-2-((4-(trifluoromethyl)phenyl)amino)pyrimidin-4-yl)amino)methyl)-6-methyloctahydrobenzofuran-3-ol hydrochloride* (**20i**)

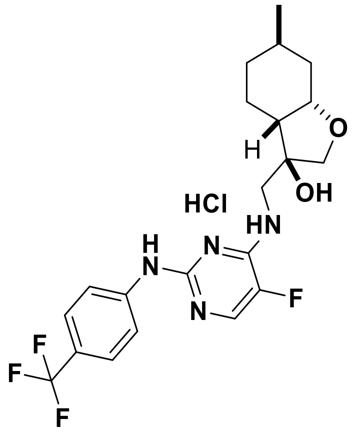

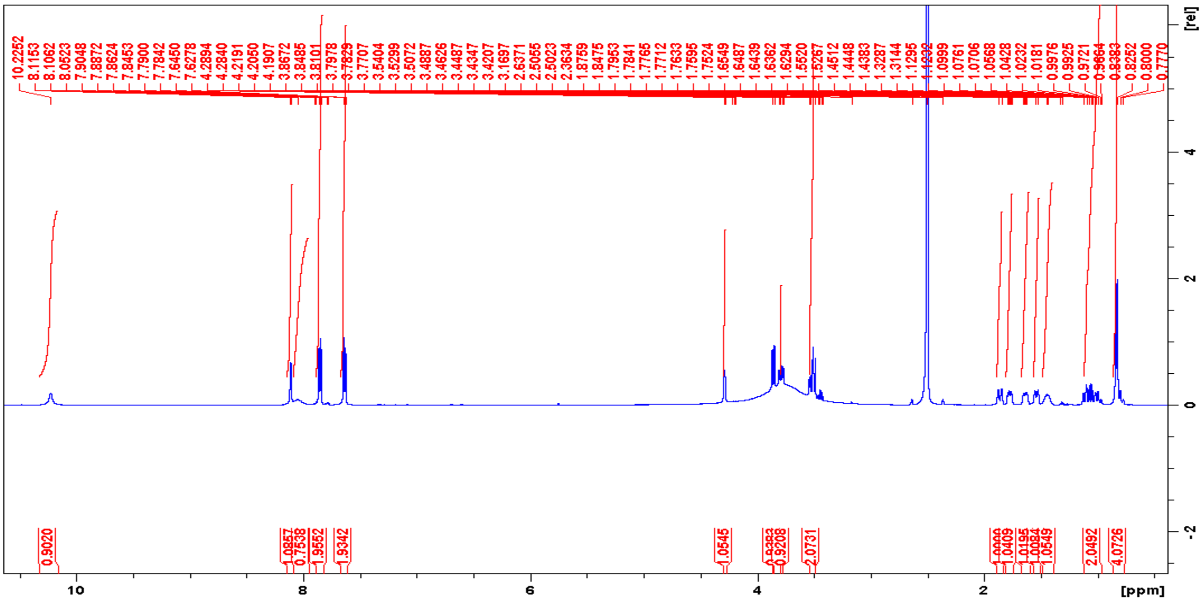

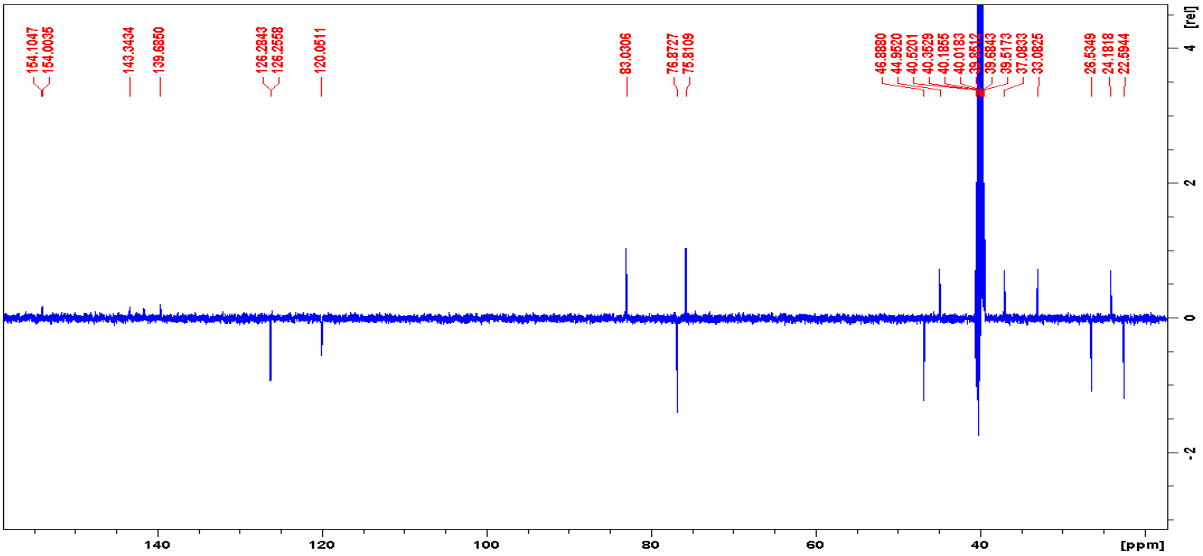

S33: ^{1}H- and ^{13}C-NMR of compound (1*R*,2*R*,5*R*)-2-((*S*)-1-((2,5-dichloropyrimidin-4-yl)amino)-2-hydroxypropan-2-yl)-5-methylcyclohexanol (**21a**)

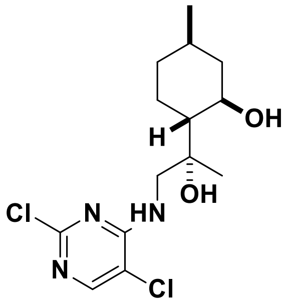

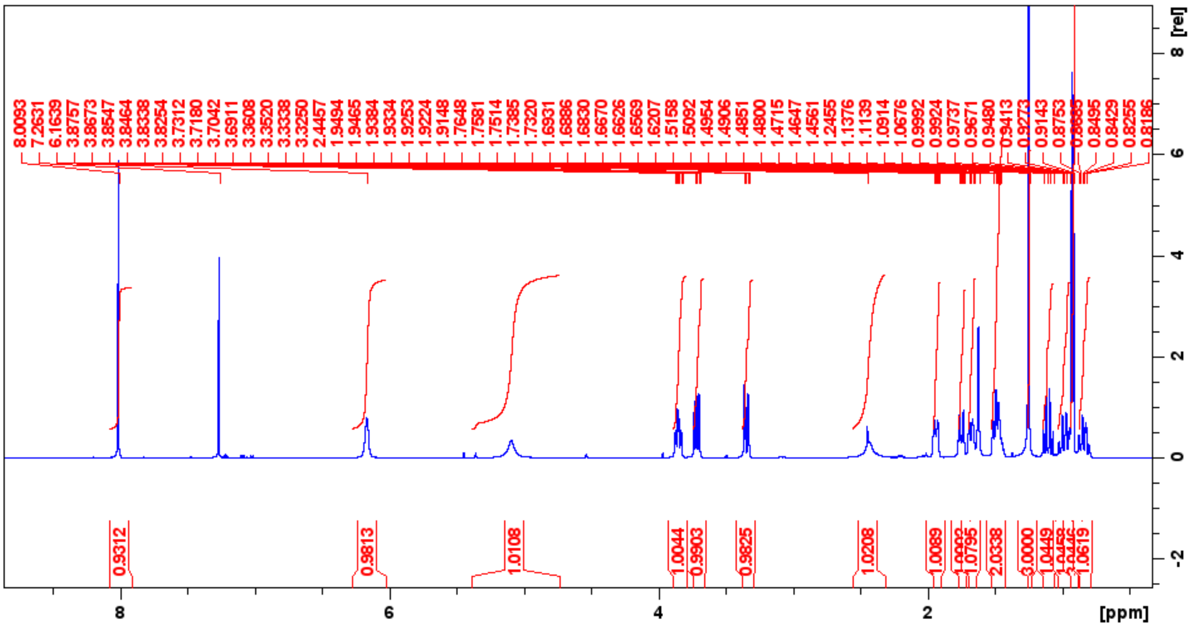

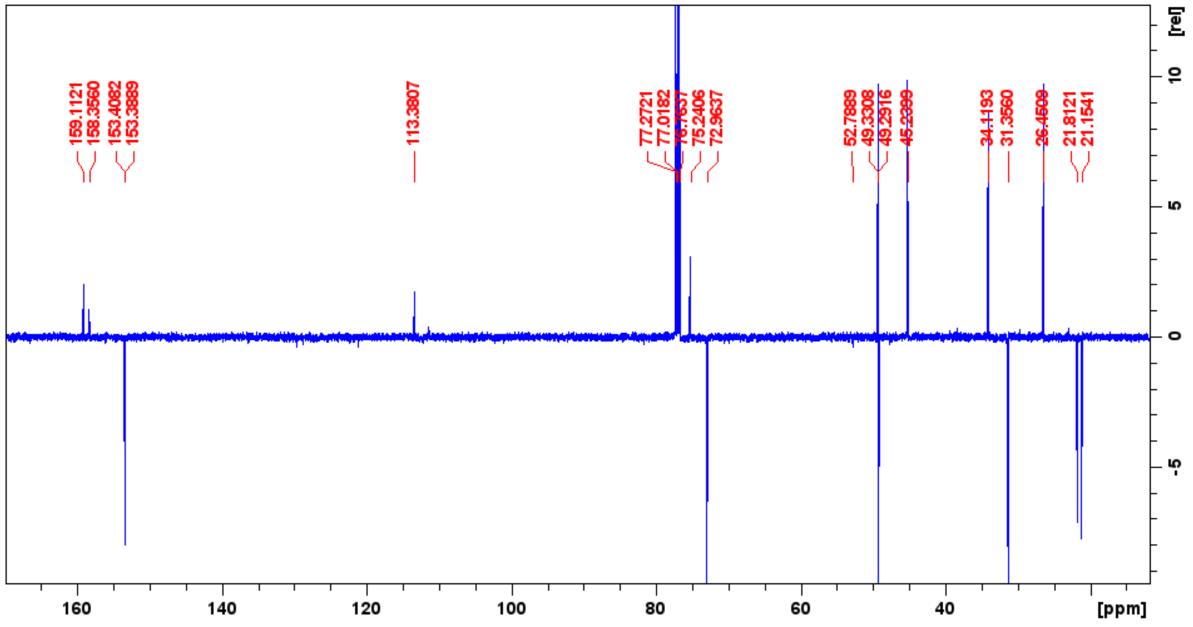

S34: ^{1}H- and ^{13}C-NMR of compound (1*R*,2*R*,5*R*)-2-((*R*)-1-((2,5-dichloropyrimidin-4-yl)amino)-2-hydroxypropan-2-yl)-5-methylcyclohexanol (**21b**)

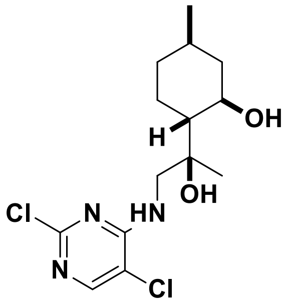

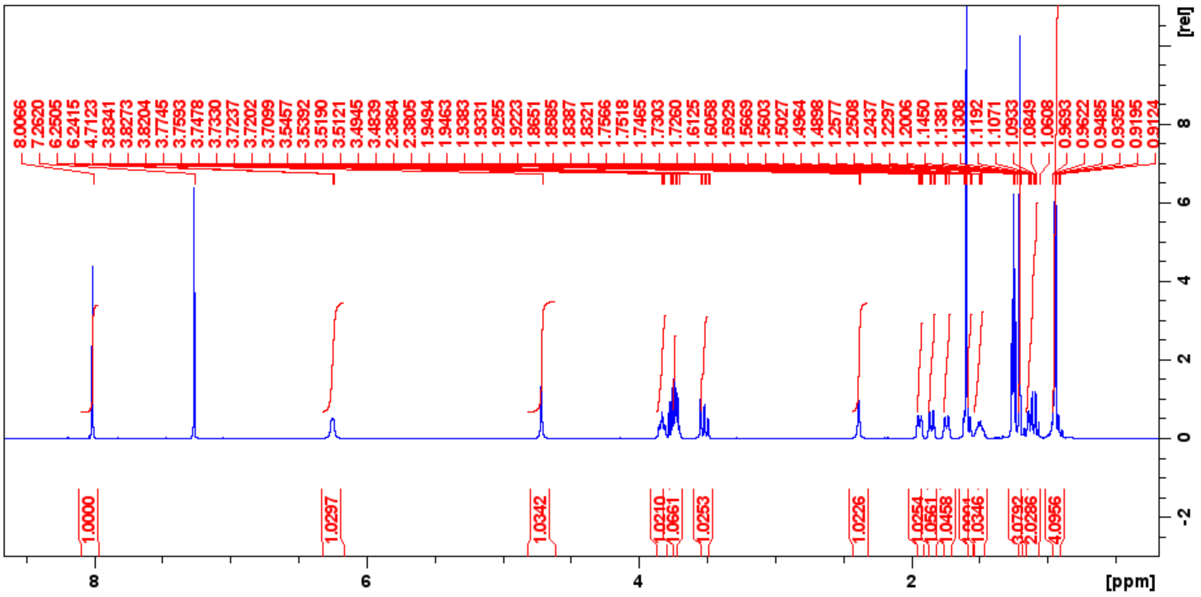

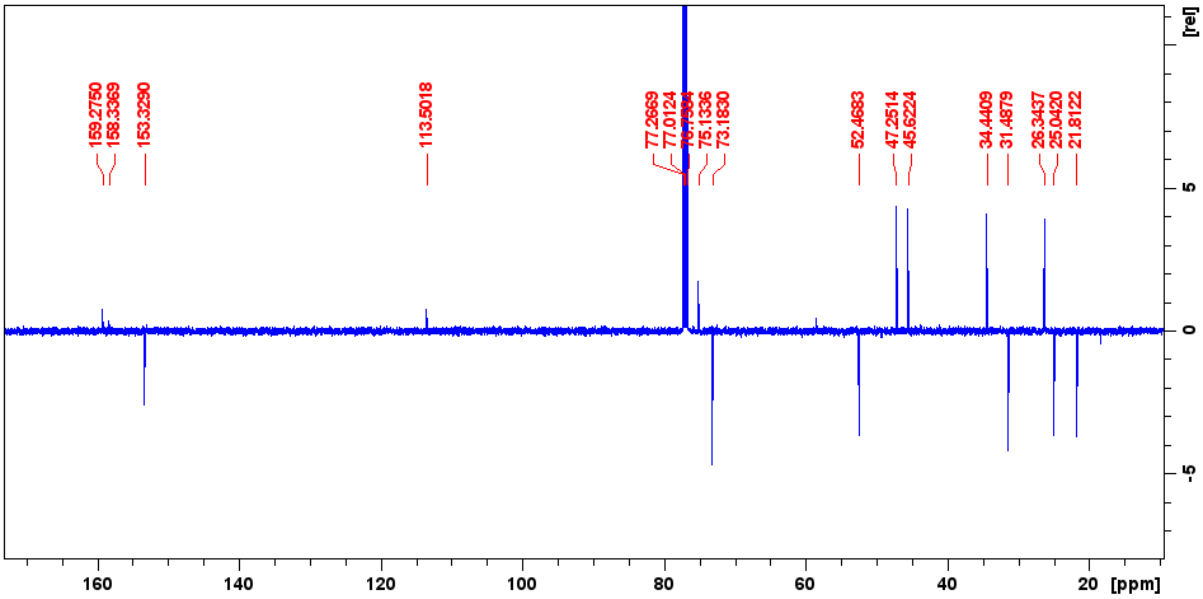

S35: ^{1}H- and ^{13}C-NMR of compound (*S*)-3-((2,5-Dichloropyrimidin-4-yl)amino)-2-((1*R*,2*R*,4*R*)-2-hydroxy-4-methylcyclohexyl)propane-1,2-diol (**21c**)

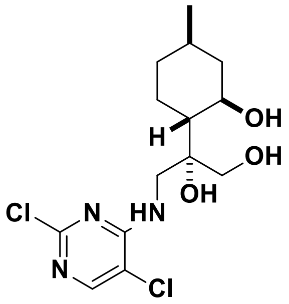

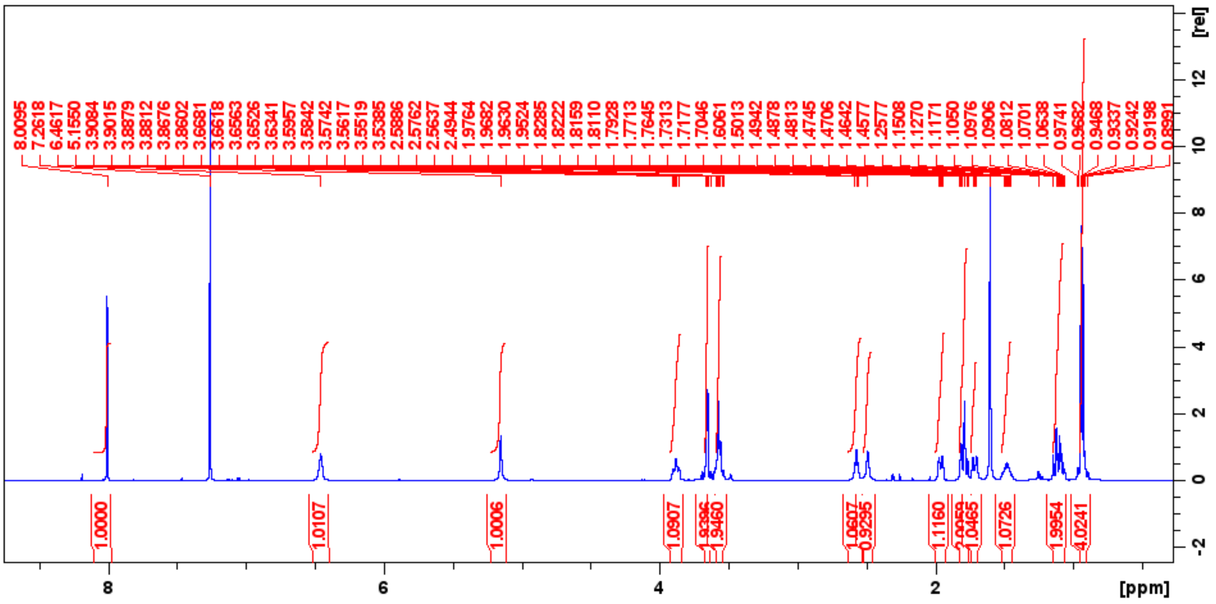

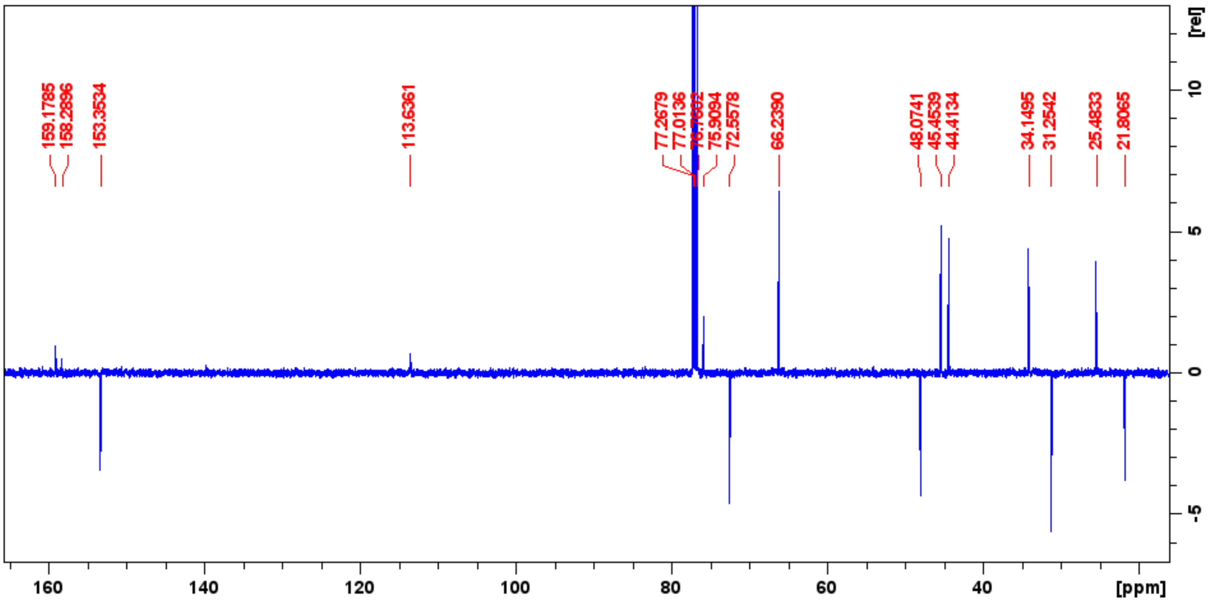

S36: ^{1}H- and ^{13}C-NMR of compound (1*R*,2*S*,5*R*)-2-((*R*)-1-((2,5-dichloropyrimidin-4-yl)amino)-3-hydroxypropan-2-yl)-5-methylcyclohexanol (**21d**)

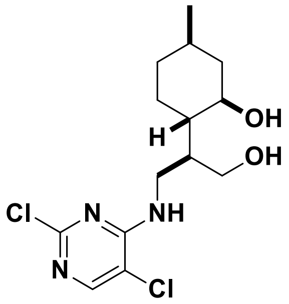

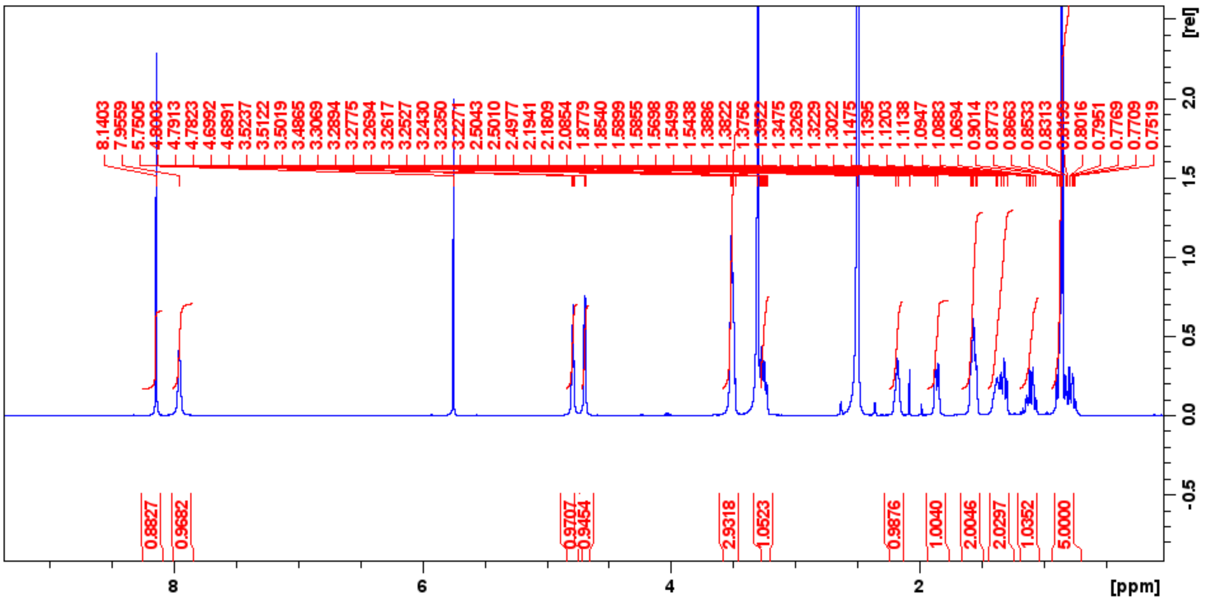

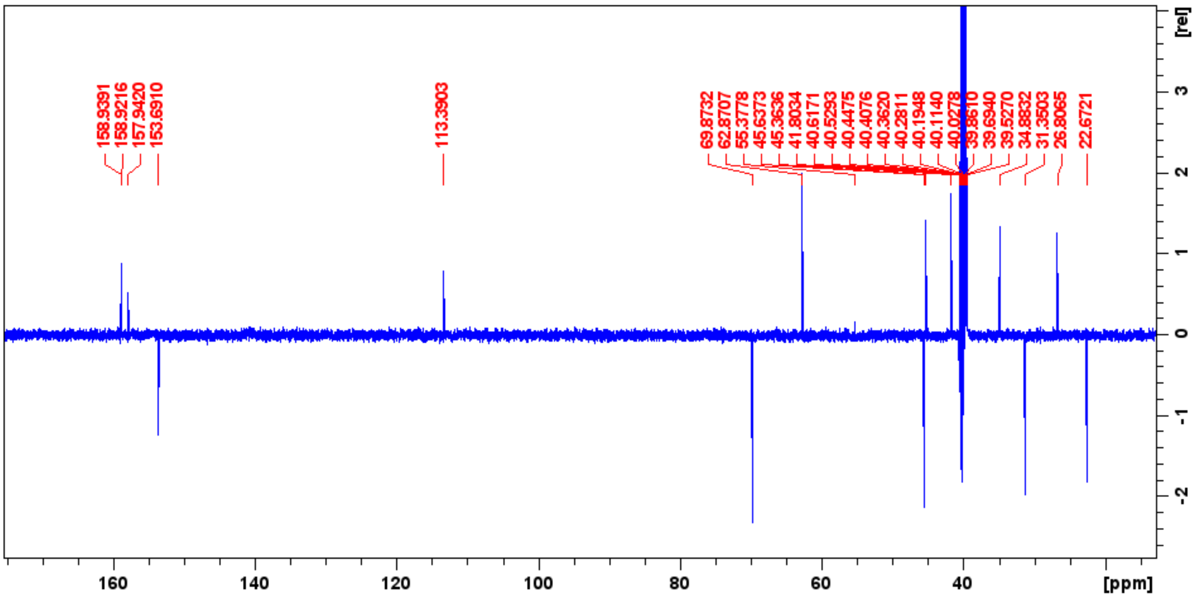

S37: ^{1}H- and ^{13}C-NMR of compound (3*S*,3a*R*,6*R*,7a*R*)-3-(((2,5-dichloropyrimidin-4-yl)amino)methyl)-6-methyloctahydrobenzofuran-3-ol (**21e**)

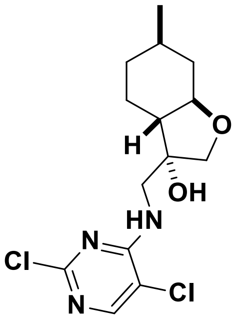

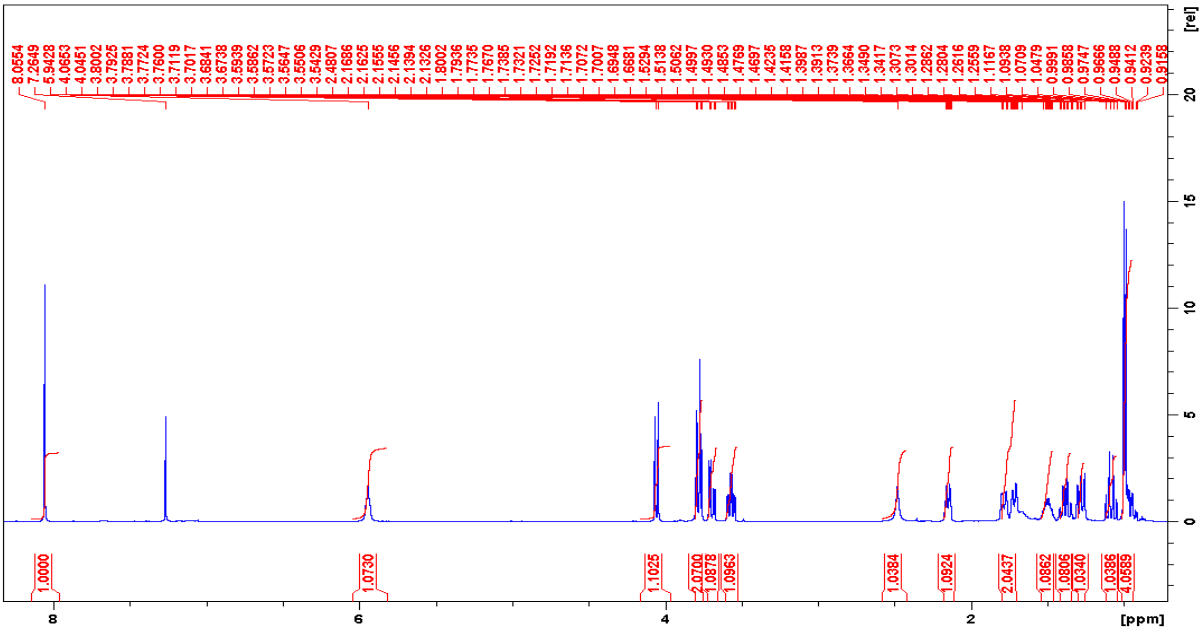

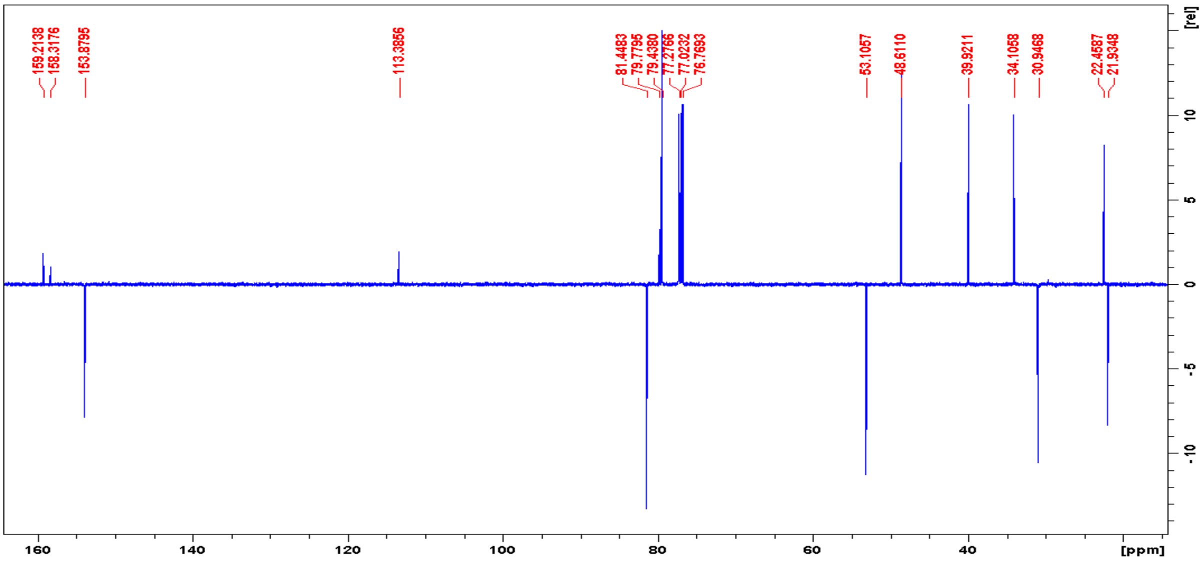

S38: ^{1}H- and ^{13}C-NMR of compound (1*S*,2*R*,5*R*)-2-((*S*)-1-((2,5-dichloropyrimidin-4-yl)amino)-2-hydroxypropan-2-yl)-5-methylcyclohexanol (**21f**)

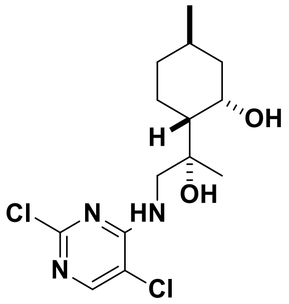

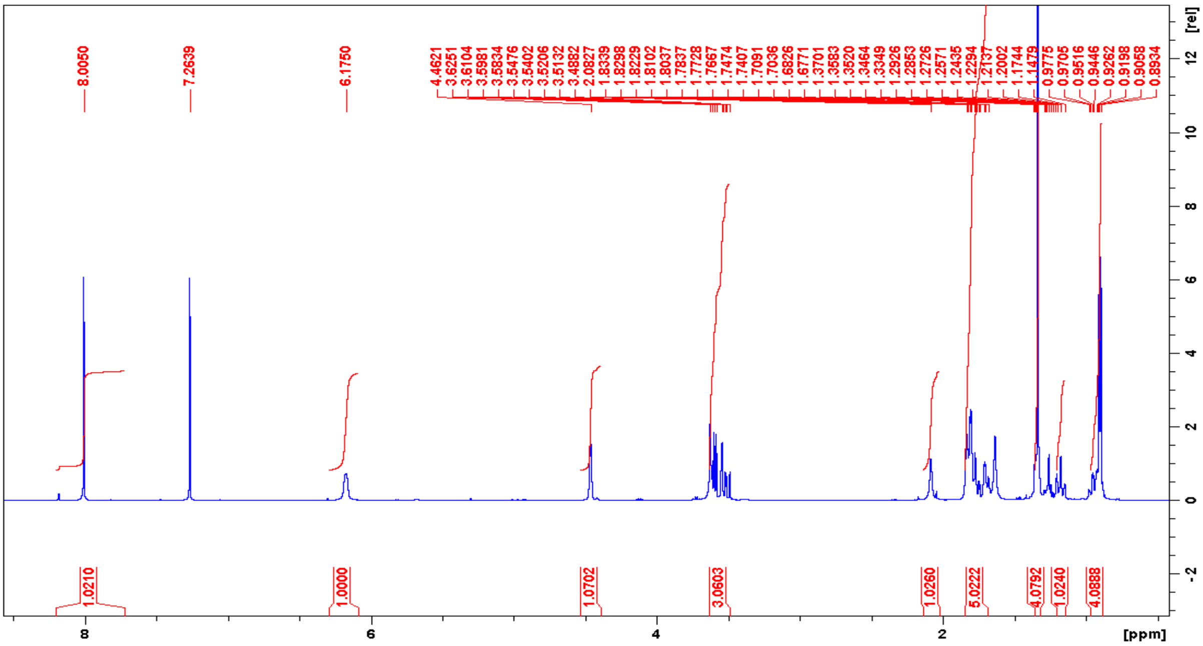

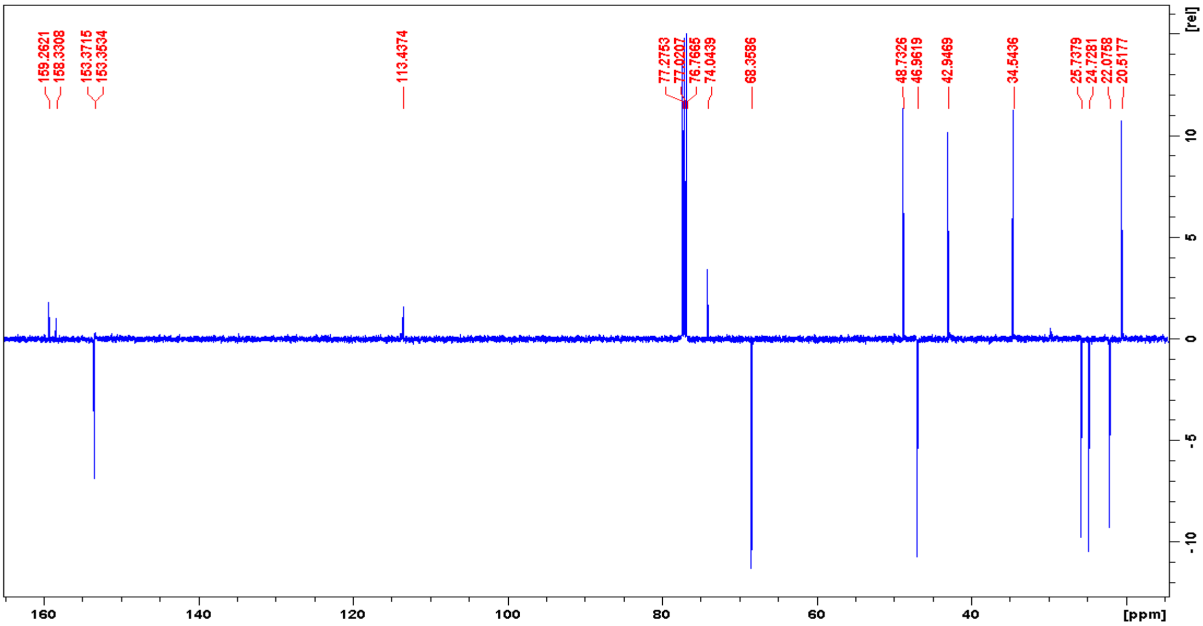

S39: ^{1}H- and ^{13}C-NMR of compound (1*S*,2*S*,5*R*)-2-((*S*)-1-((2,5-dichloropyrimidin-4-yl)amino)-3-hydroxypropan-2-yl)-5-methylcyclohexanol (**21g**)

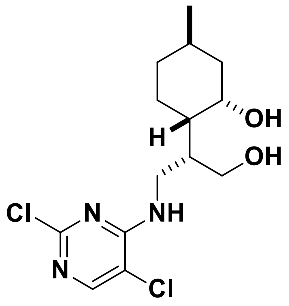

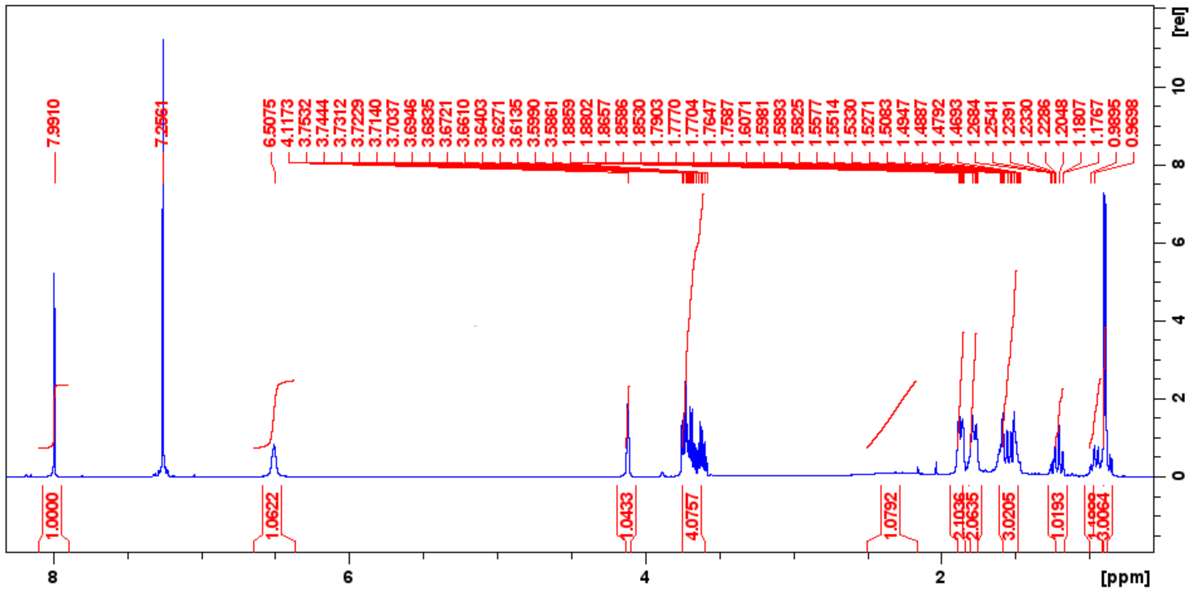

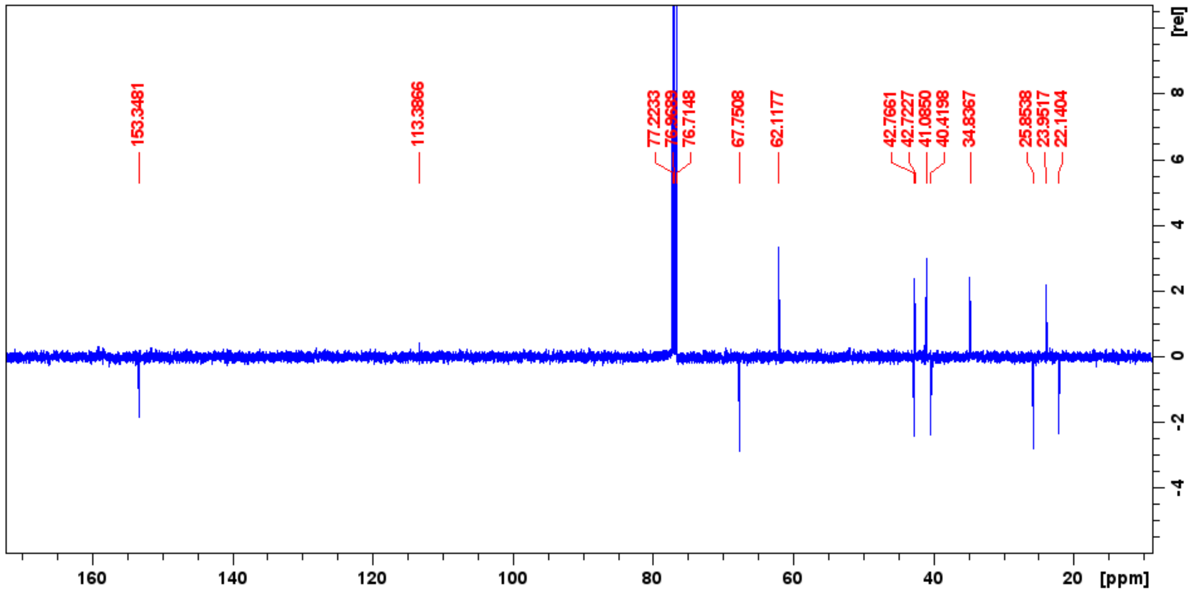

S40: ^{1}H- and ^{13}C-NMR of compound (3*R*,3a*R*,6*R*,7a*S*)-3-(((2,5-dichloropyrimidin-4-yl)amino)methyl)-6-methyloctahydrobenzofuran-3-ol (**21h**)

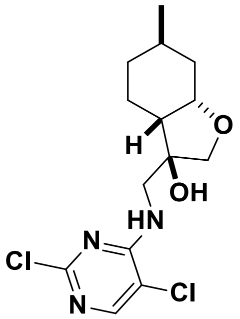

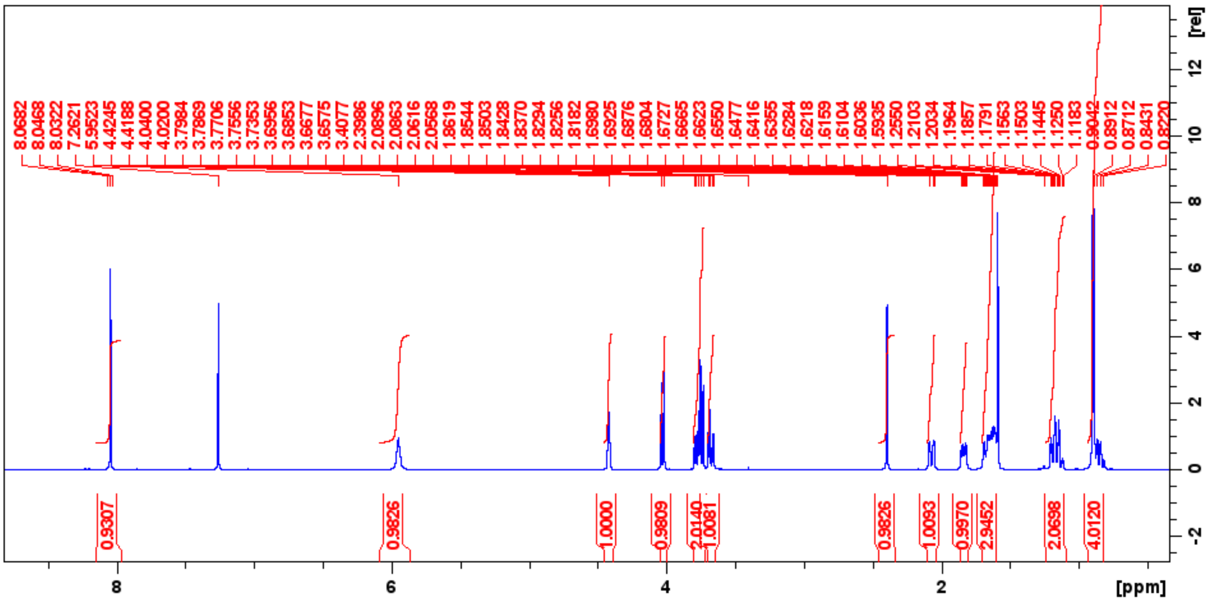

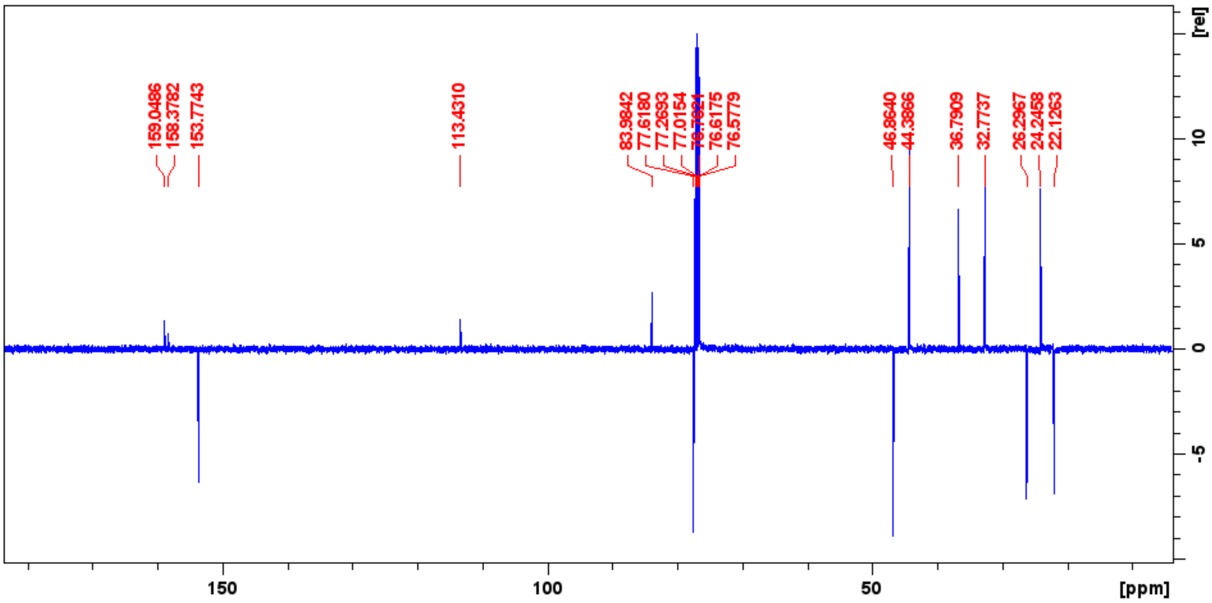

S41: ^{1}H- and ^{13}C-NMR of compound *(1R,2R,5R)-2-((S)-1-((5-chloro-2-((4-(trifluoromethyl)phenyl)amino)pyrimidin-4-yl)amino)-2-hydroxypropan-2-yl)-5-methylcyclohexan-1-ol hydrochloride* (**22a**)

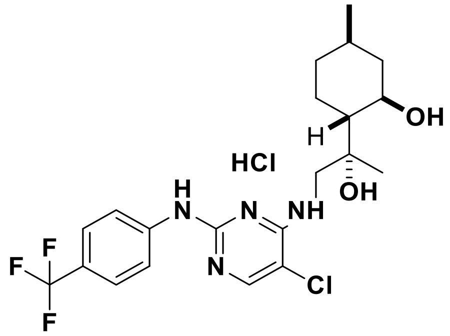

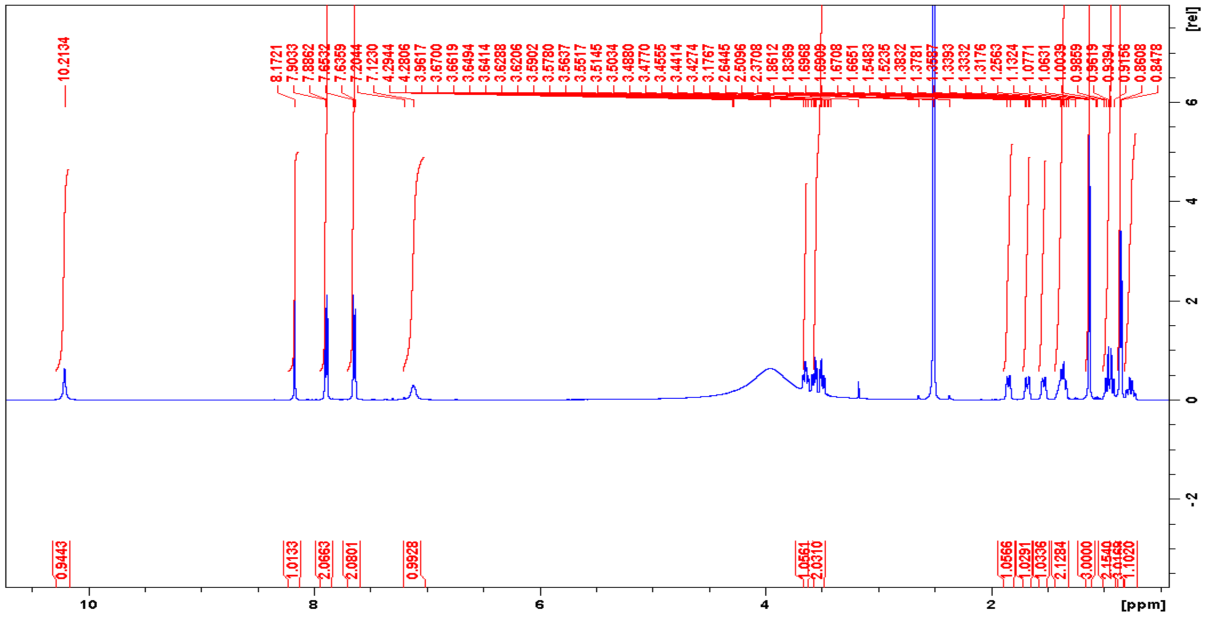

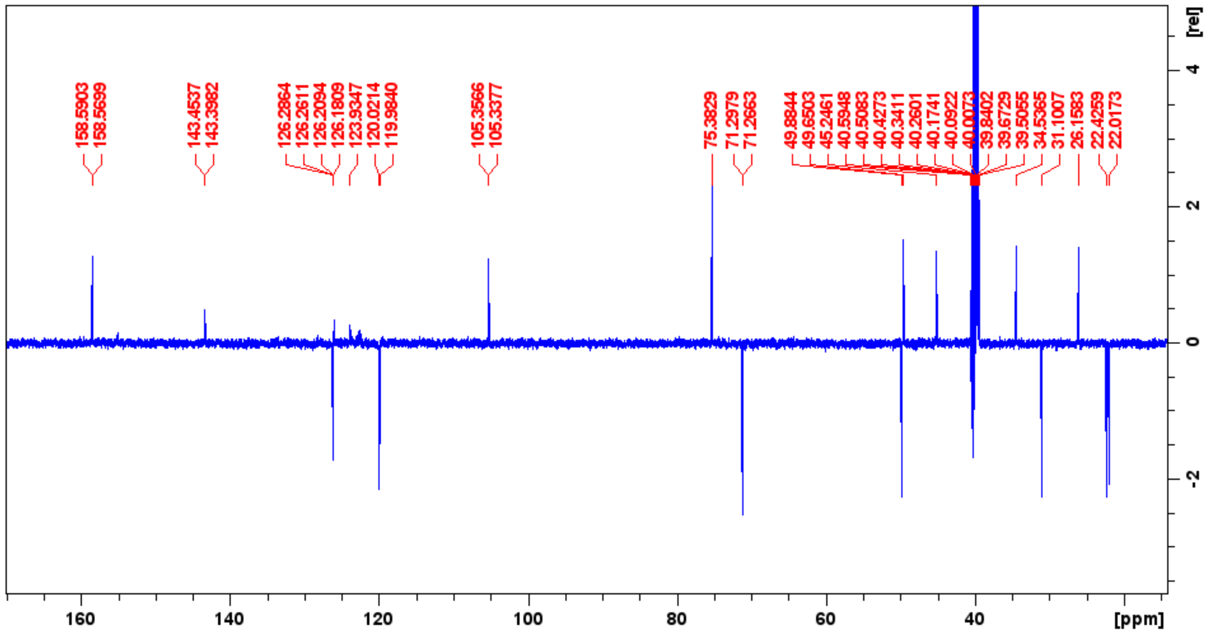

S42: ^{1}H- and ^{13}C-NMR of compound *((1R,2R,5R)-2-((R)-1-((5-chloro-2-((4-(trifluoromethyl)phenyl)amino)pyrimidin-4-yl)amino)-2-hydroxypropan-2-yl)-5-methylcyclohexan-1-ol hydrochloride* (**22b**)

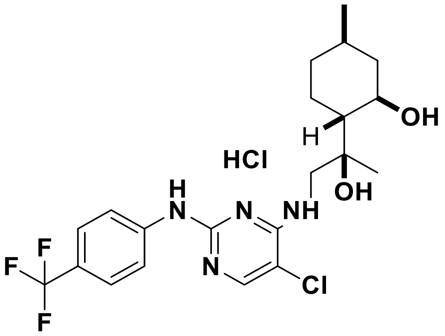

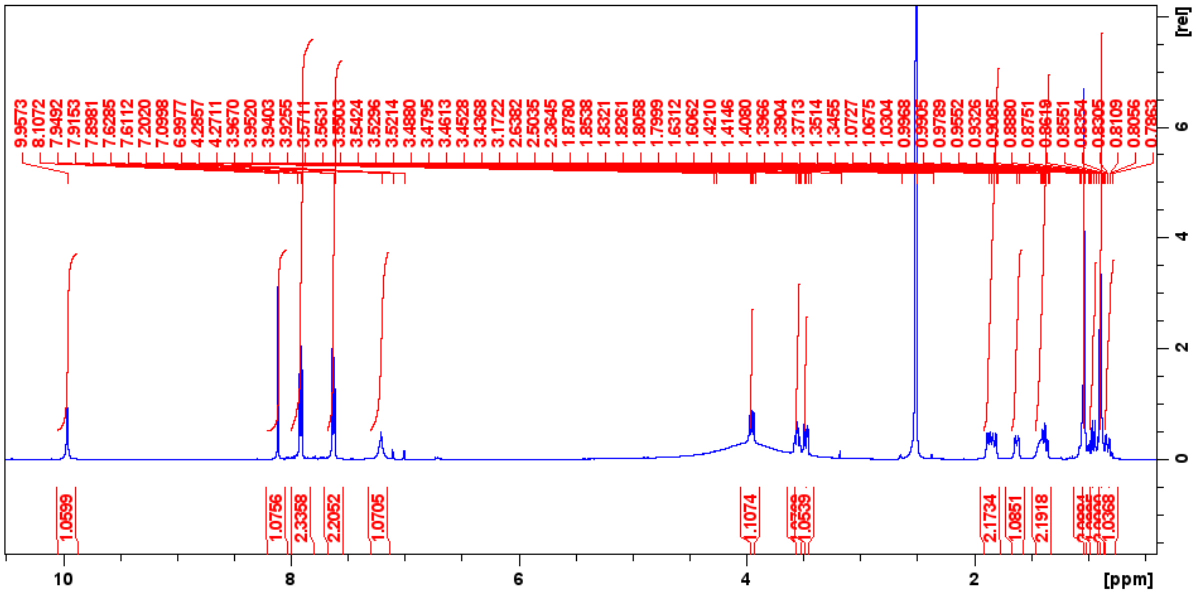

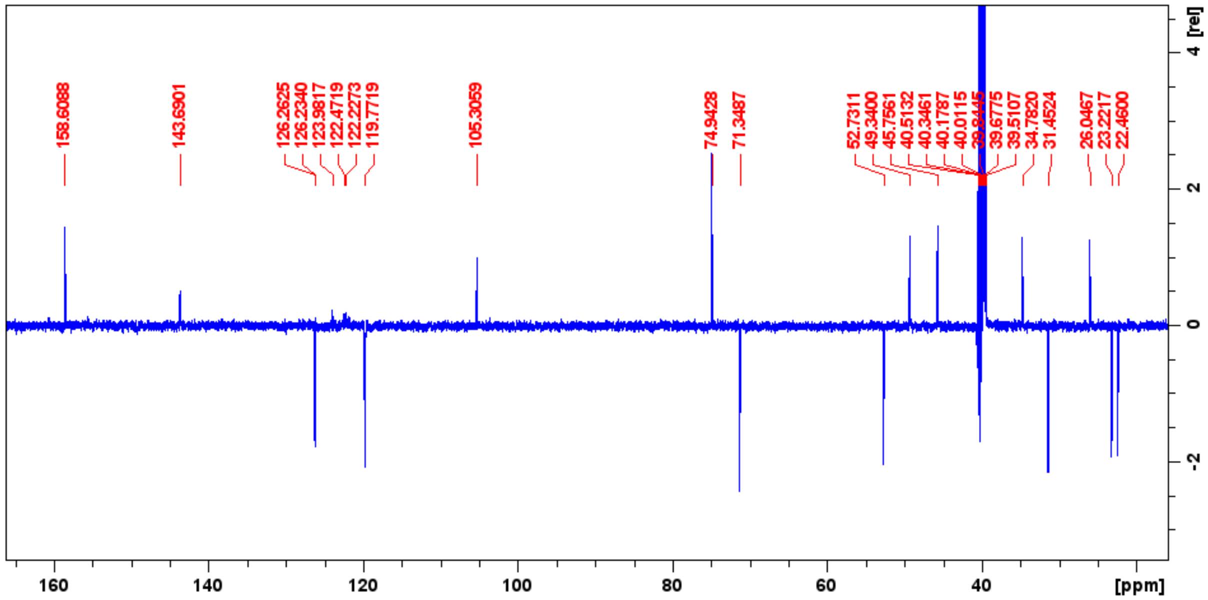

S33: ^{1}H- and ^{13}C-NMR of compound *(S)-3-((5-chloro-2-((4-(trifluoromethyl)phenyl)amino)pyrimidin-4-yl)amino)-2-((1R,2R,4R)-2-hydroxy-4-methylcyclohexyl)propane-1,2-diol hydrochloride* (**22c**)

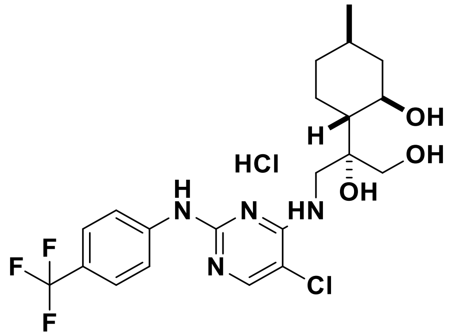

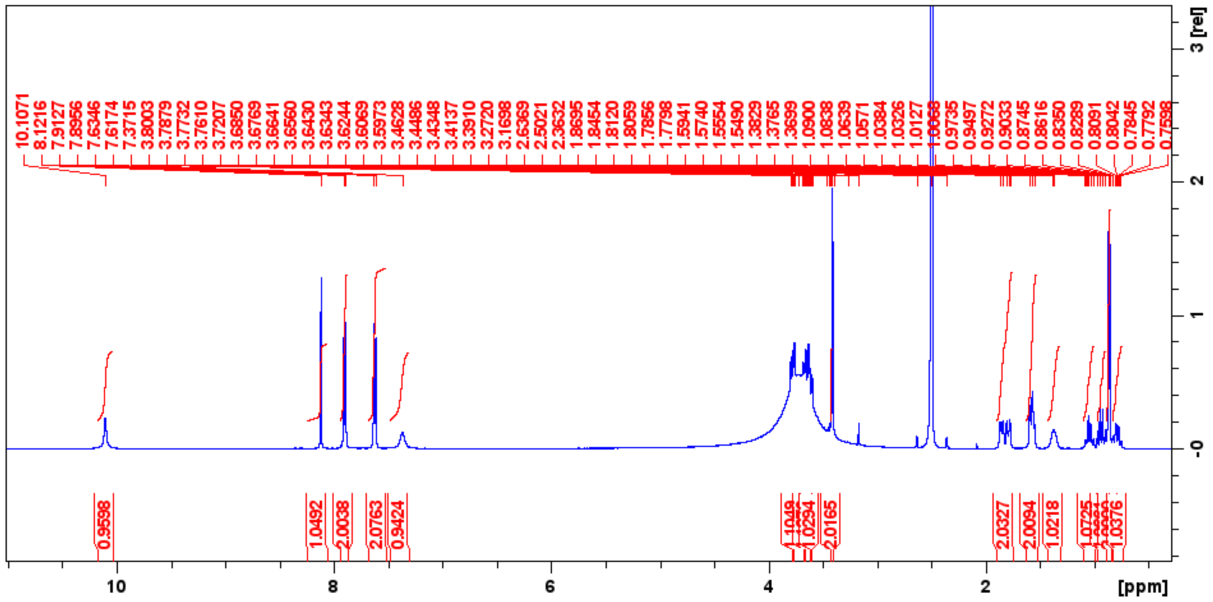

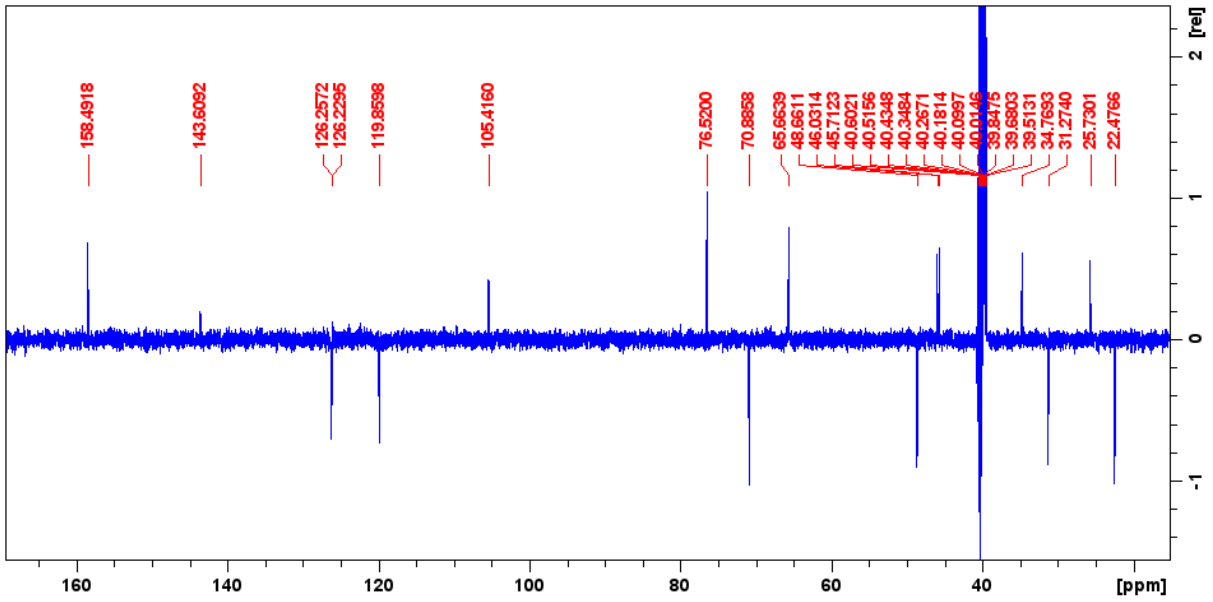

S34: ^{1}H- and ^{13}C-NMR of compound *(1R,2S,5R)-2-((R)-1-((5-chloro-2-((4-(trifluoromethyl)phenyl)amino)pyrimidin-4-yl)amino)-3-hydroxypropan-2-yl)-5-methylcyclohexan-1-ol hydrochloride* (**22d**)

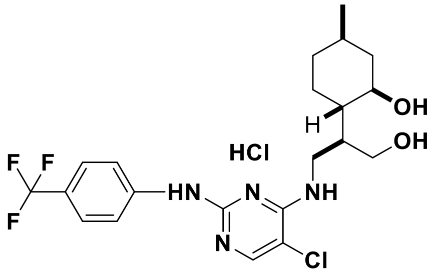

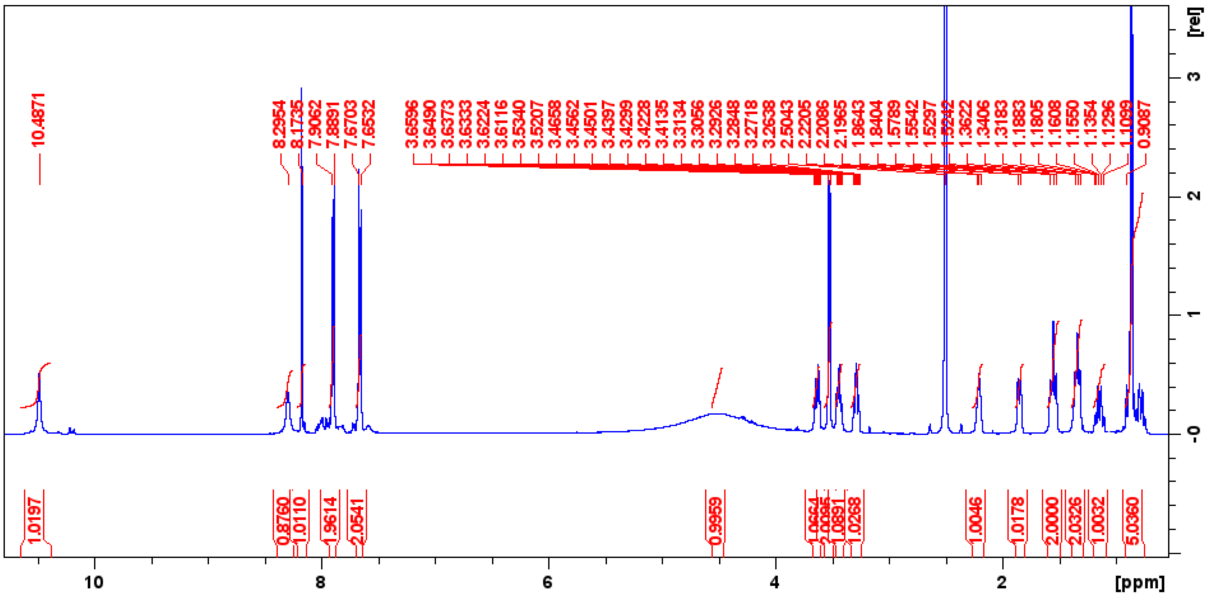

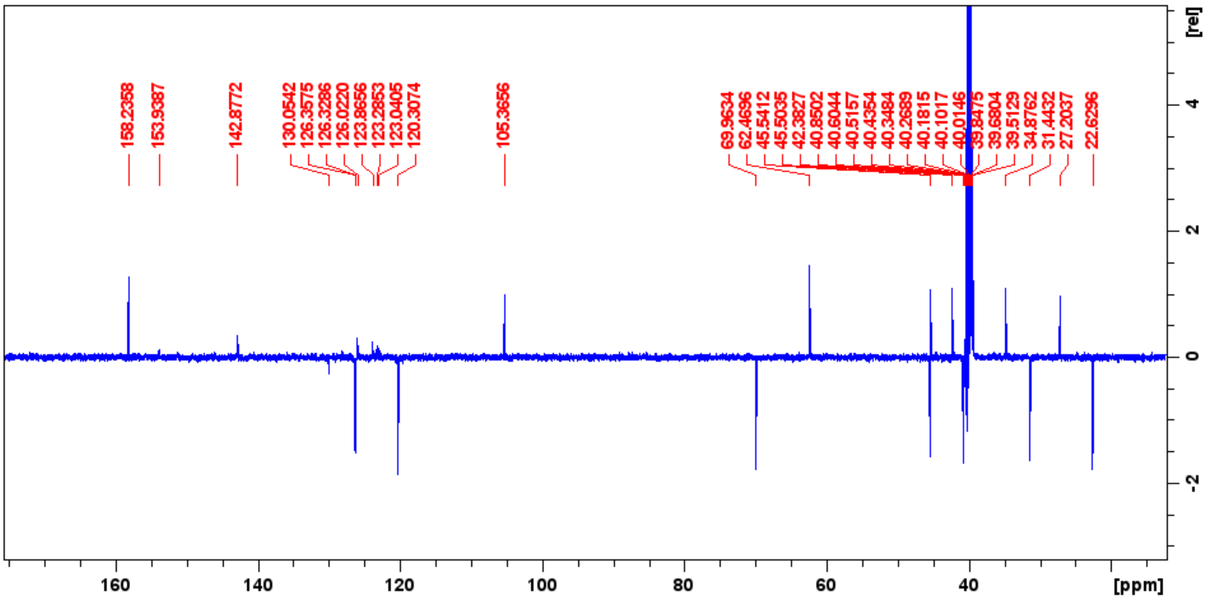

S35: ^{1}H- and ^{13}C-NMR of compound *(3S,3aR,6R,7aR)-3-(((5-chloro-2-((4-(trifluoromethyl)phenyl)amino)pyrimidin-4-yl)amino)methyl)-6-methyloctahydrobenzofuran-3-ol hydrochloride* (**22e**)

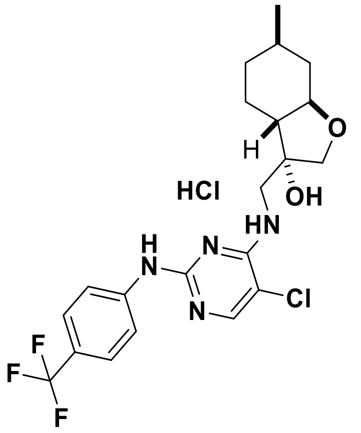

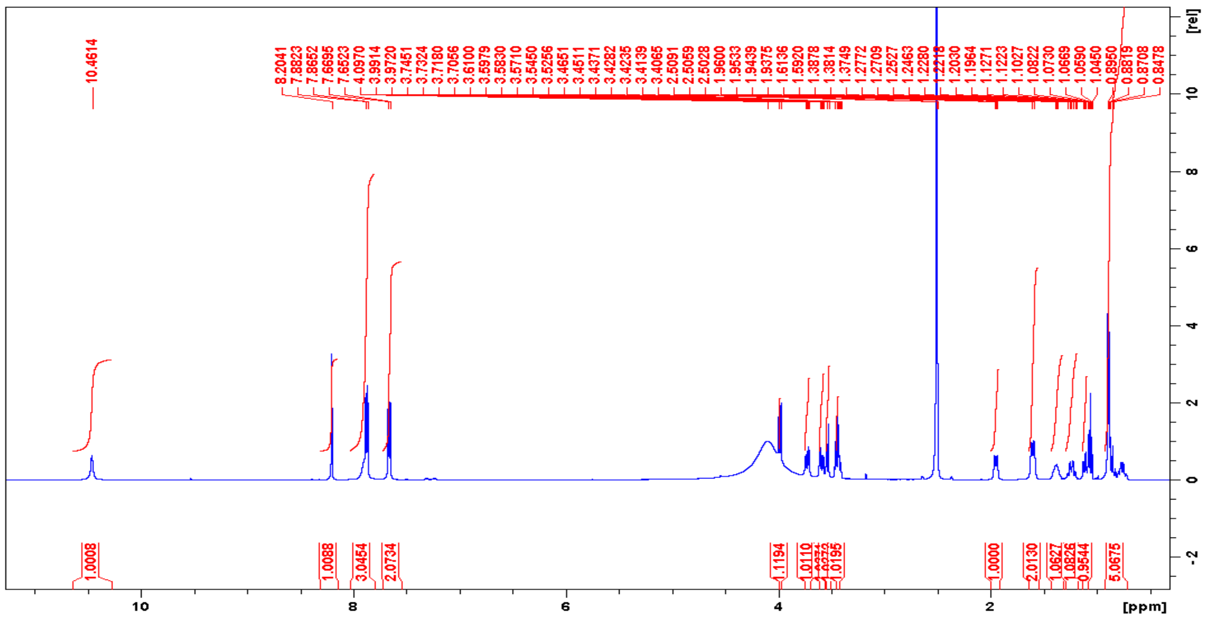

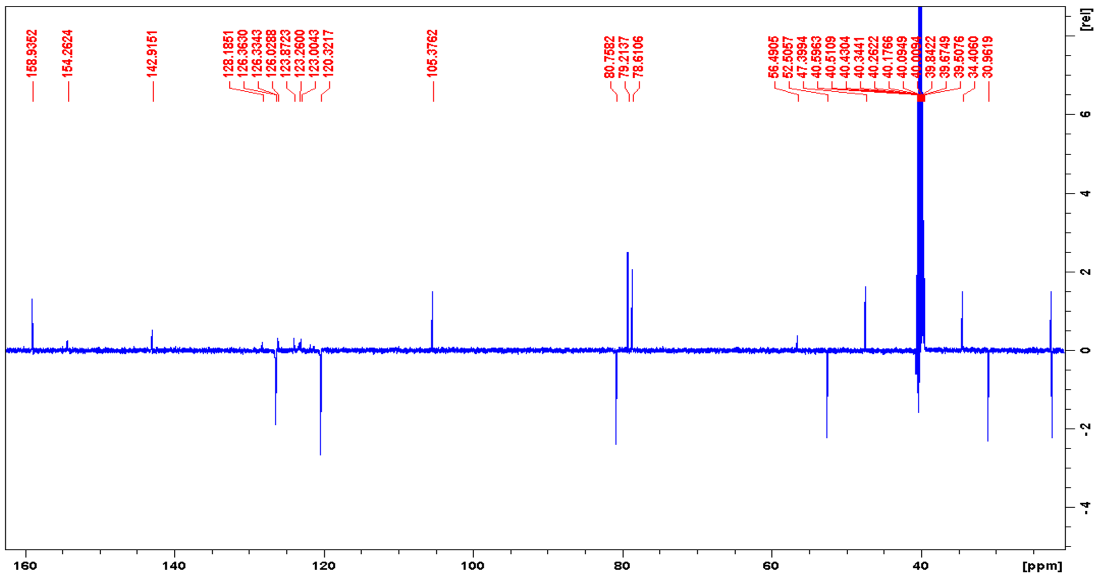

S36: ^{1}H- and ^{13}C-NMR of compound *(1S,2R,5R)-2-((S)-1-((5-chloro-2-((4-(trifluoromethyl)phenyl)amino)pyrimidin-4-yl)amino)-2-hydroxypropan-2-yl)-5-methylcyclohexan-1-ol hydrochloride* (**22f**)

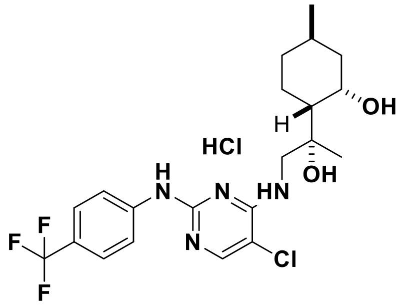

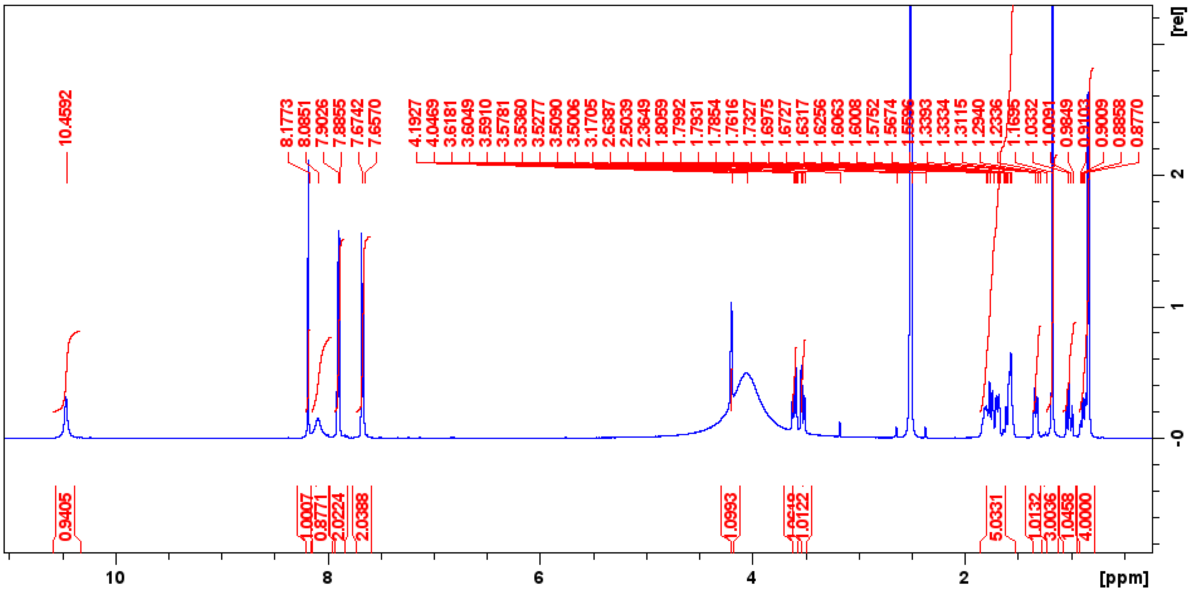

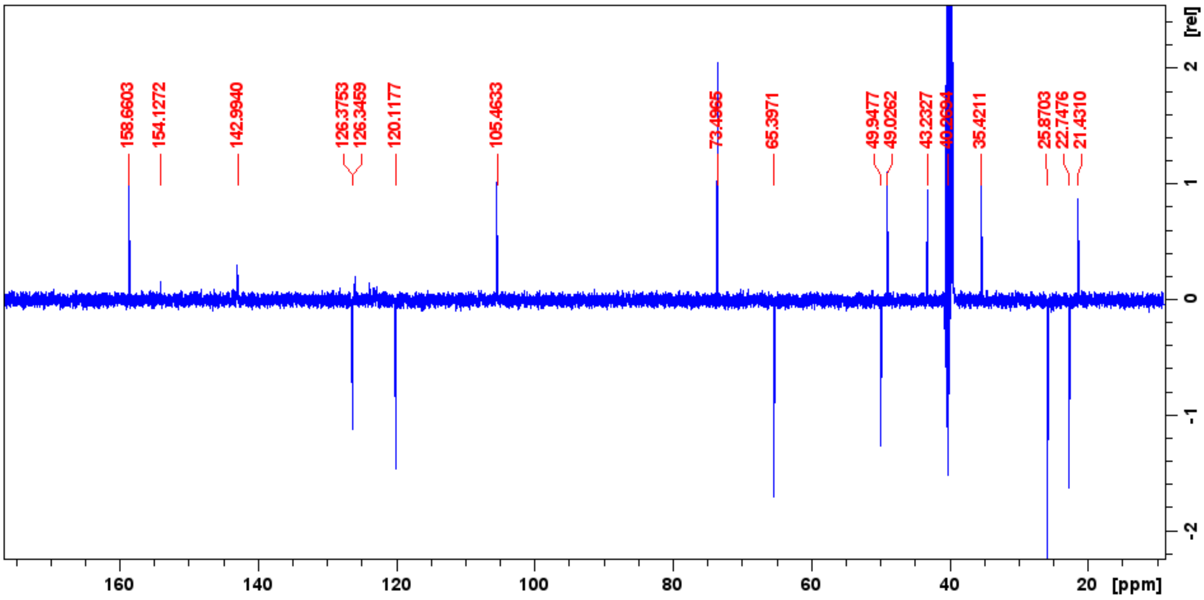

S37: ^{1}H- and ^{13}C-NMR of compound *(1S,2S,5R)-2-((S)-1-((5-chloro-2-((4-(trifluoromethyl)phenyl)amino)pyrimidin-4-yl)amino)-3-hydroxypropan-2-yl)-5-methylcyclohexan-1-ol hydrochloride* (**22g**)

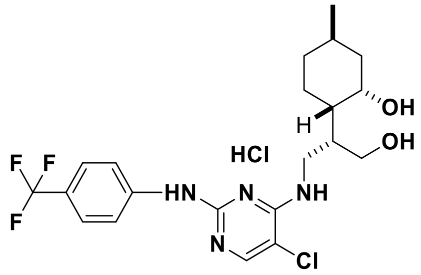

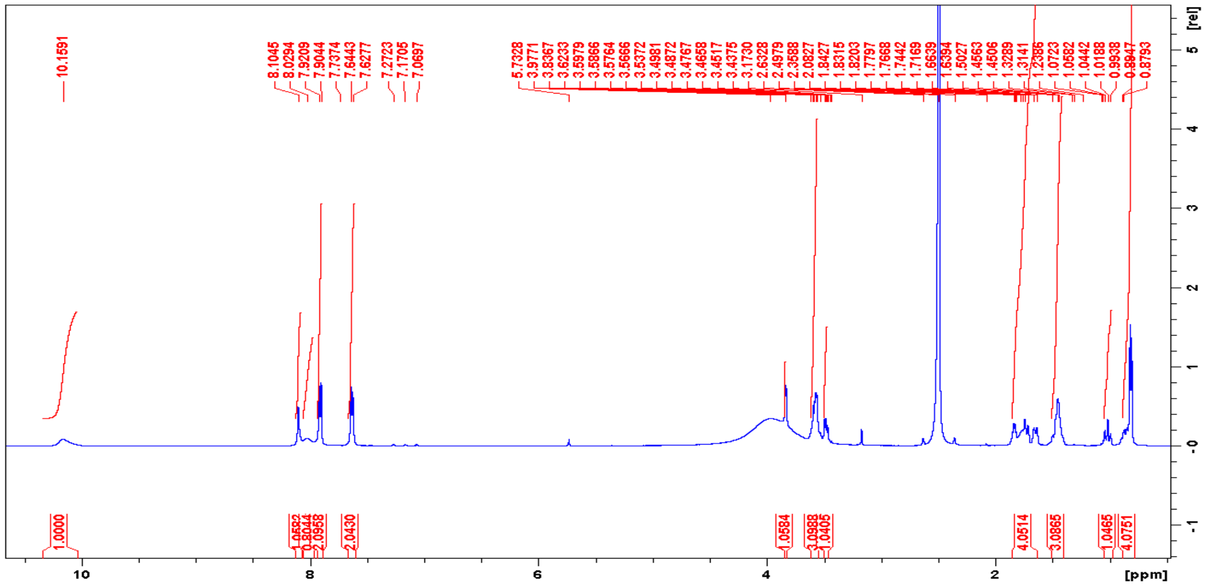

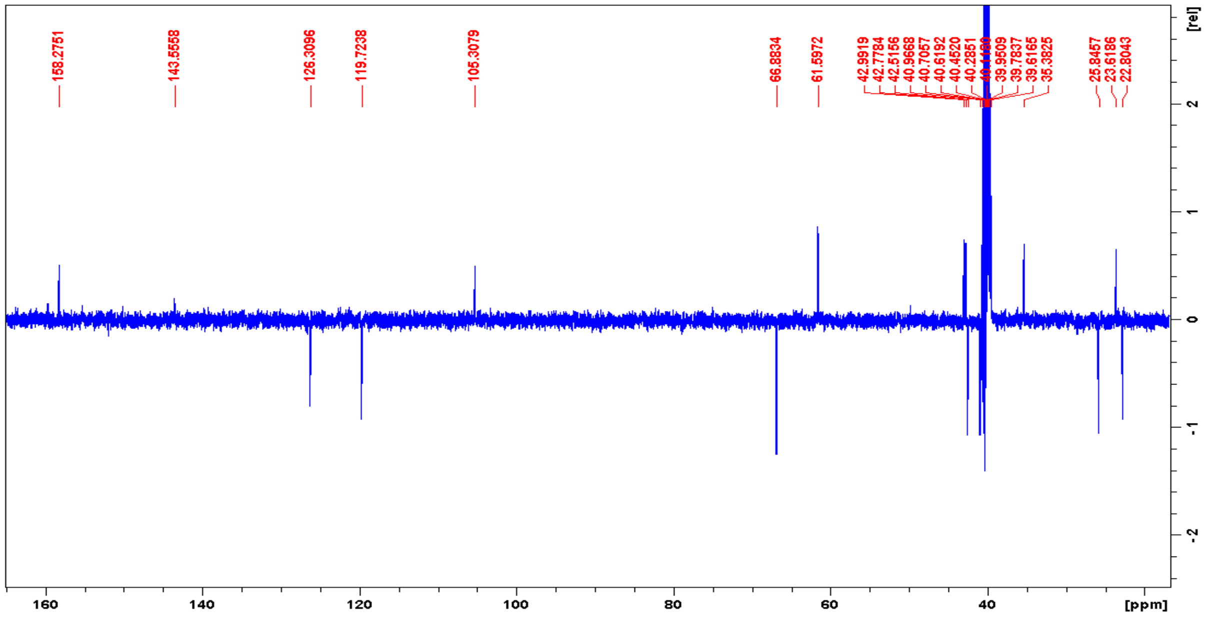

S38: ^{1}H- and ^{13}C-NMR of compound *(3R,3aR,6R,7aS)-3-(((5-chloro-2-((4-(trifluoromethyl)phenyl)amino)pyrimidin-4-yl)amino)methyl)-6-methyloctahydrobenzofuran-3-ol hydrochloride* (**22h**)

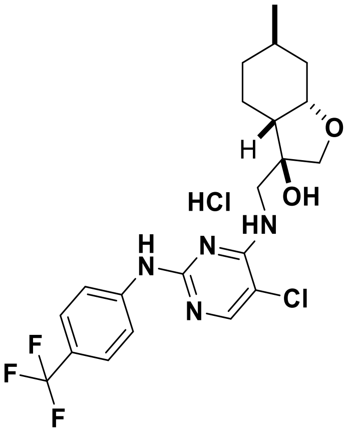

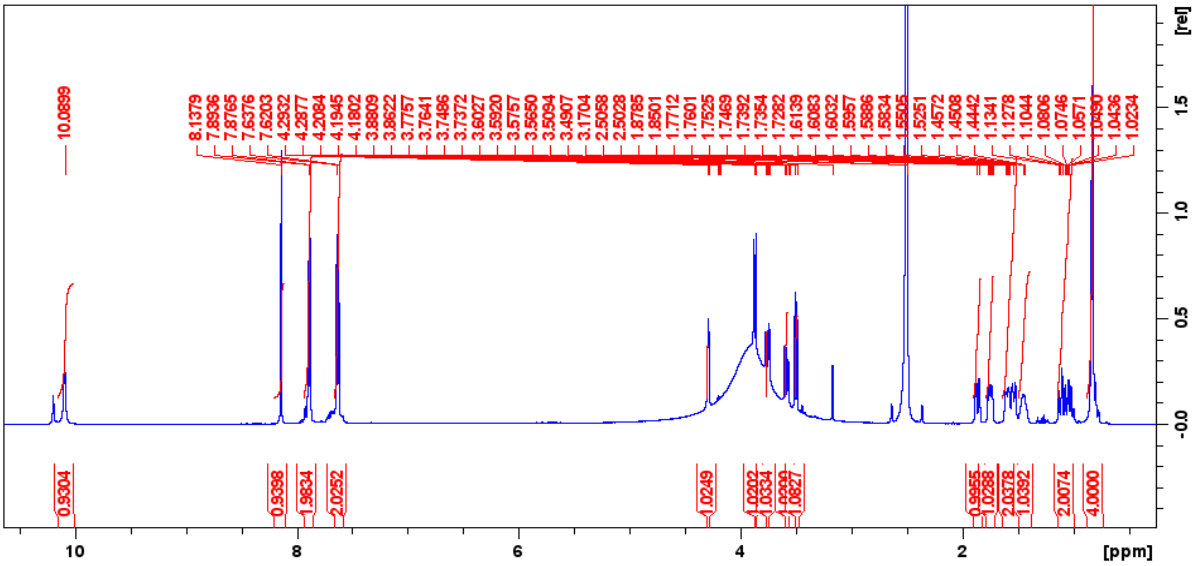

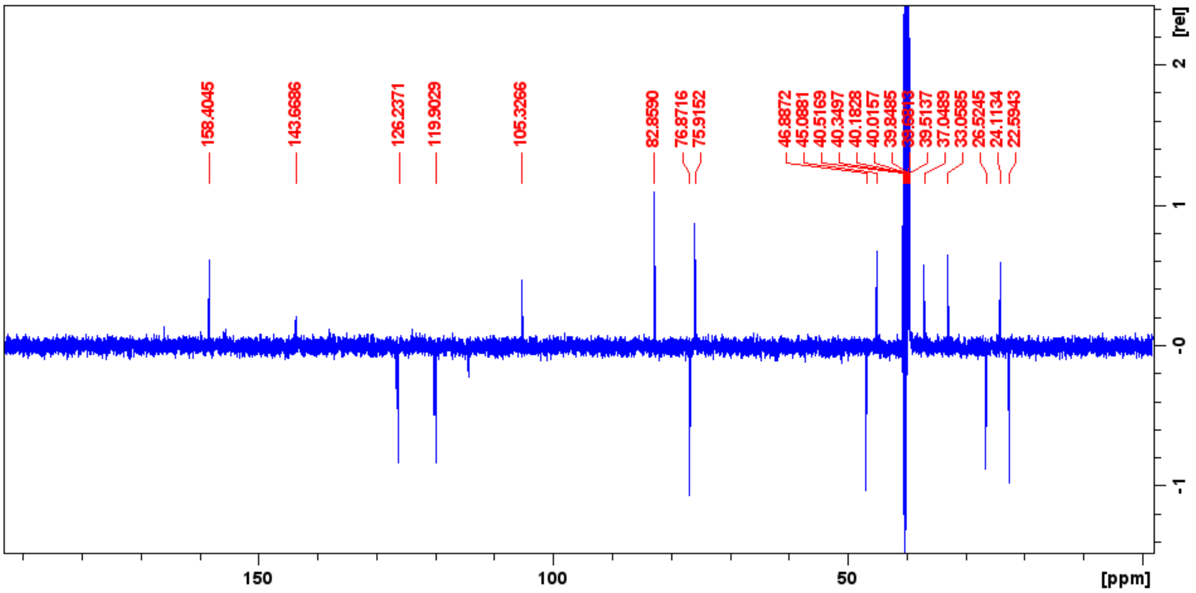

S39: ^{1}H- and ^{13}C-NMR of compound (1*R*,2*R*,5*R*)-2-((*S*)-1-((5-amino-6-chloropyrimidin-4-yl)amino)-2-hydroxypropan-2-yl)-5-methylcyclohexanol (**23a**)

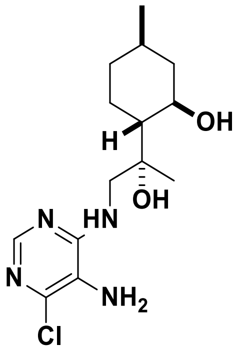

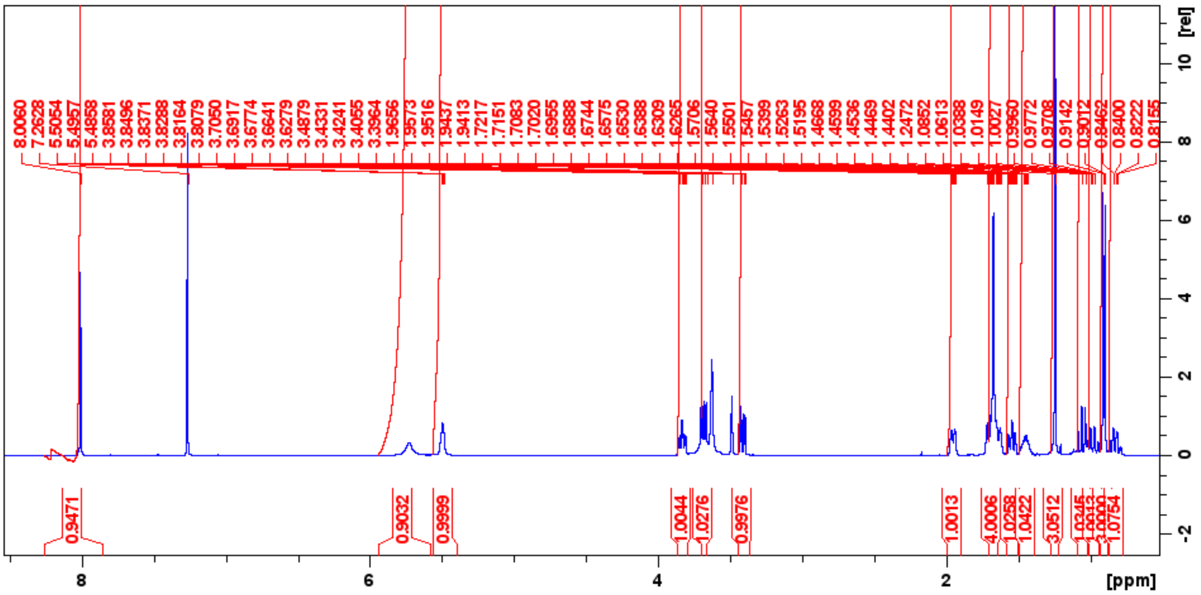

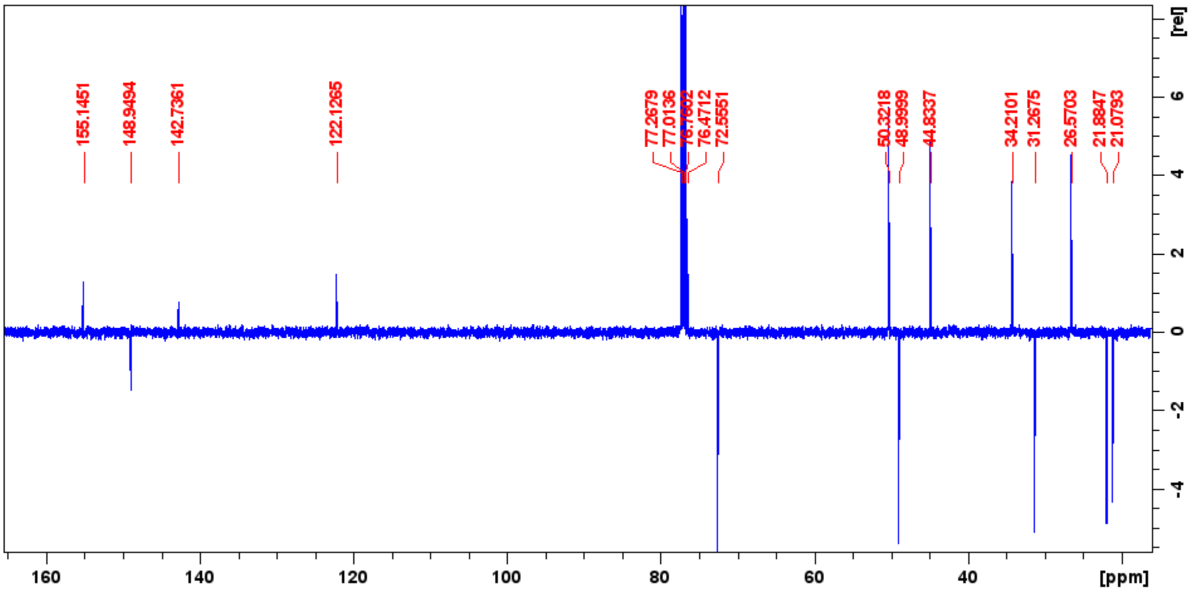

S40: ^{1}H- and ^{13}C-NMR of compound (1*R*,2*R*,5*R*)-2-((*R*)-1-((5-amino-6-chloropyrimidin-4-yl)amino)-2-hydroxypropan-2-yl)-5-methylcyclohexanol (**23b**)

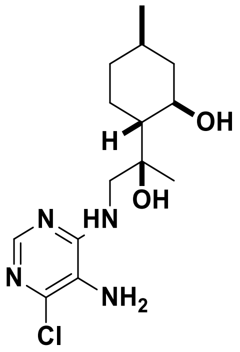

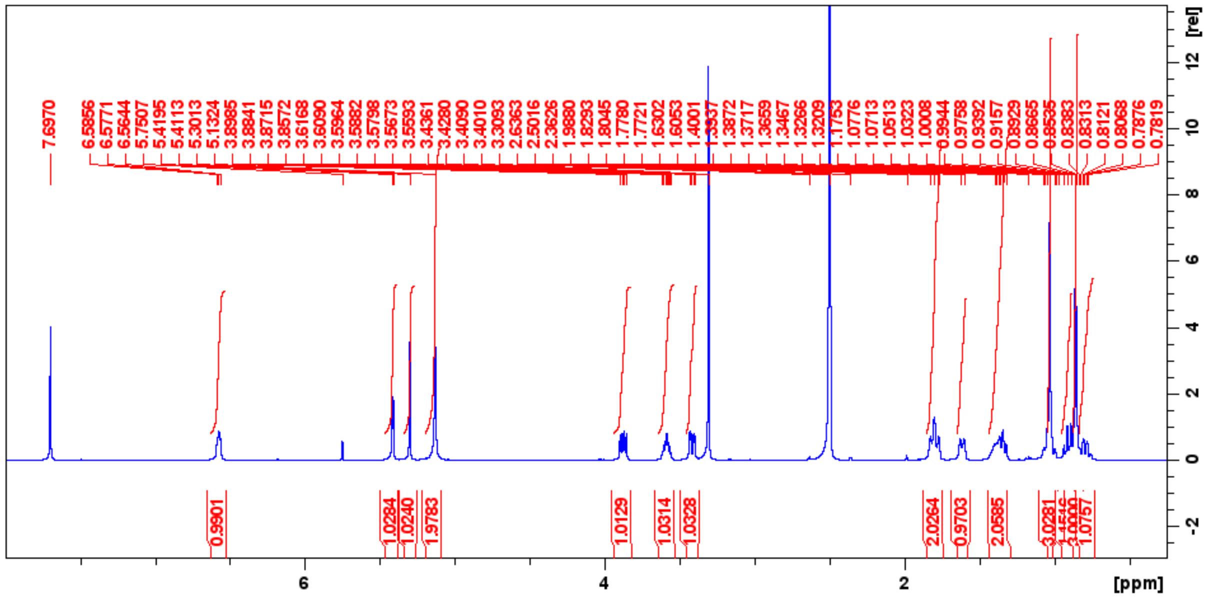

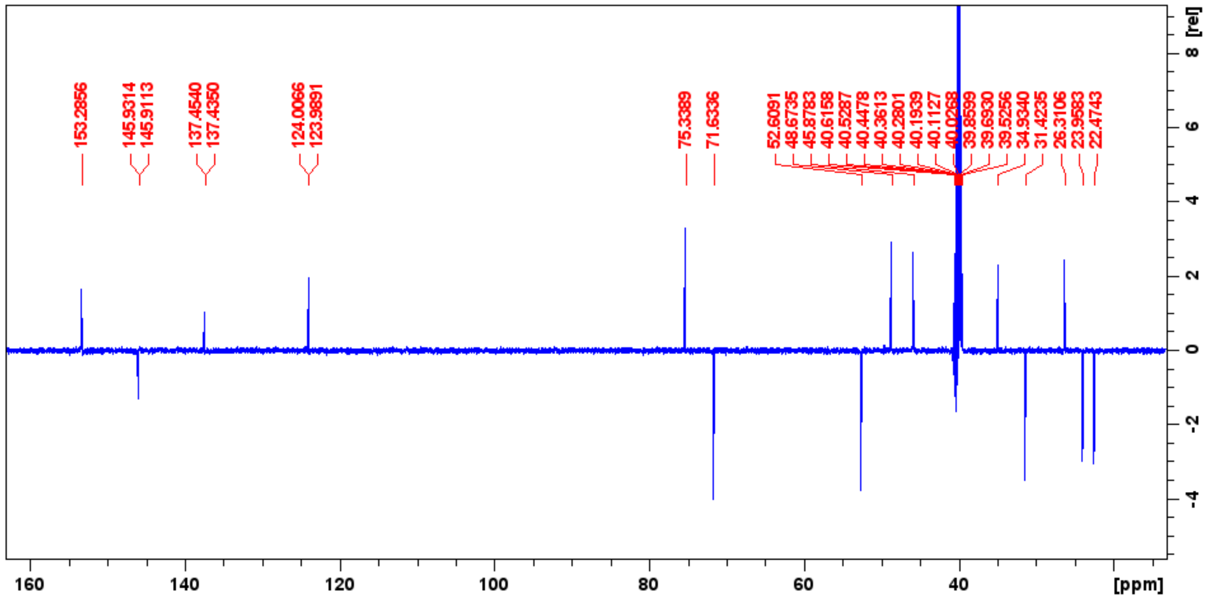

S41: ¹H- and ¹³C-NMR of compound (*S*)-3-((5-amino-6-chloropyrimidin-4-yl)amino)-2-((1*R*,2*R*,4*R*)-2-hydroxy-4-methylcyclohexyl)propane-1,2-diol (**23c**)

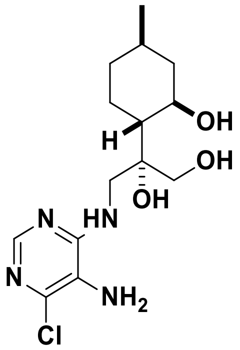

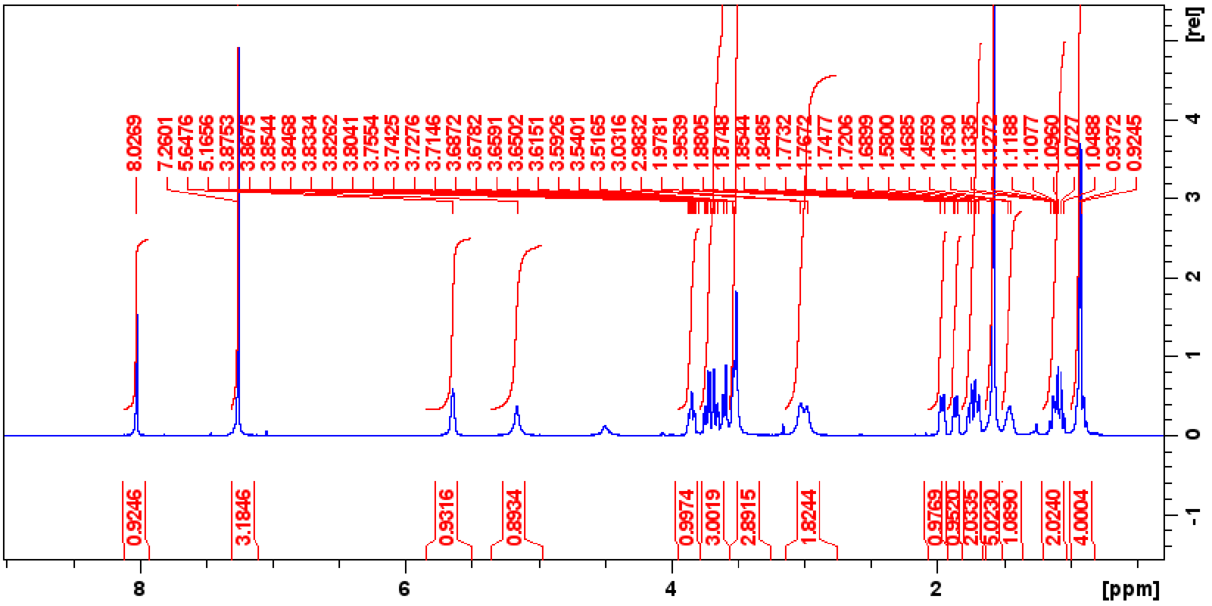

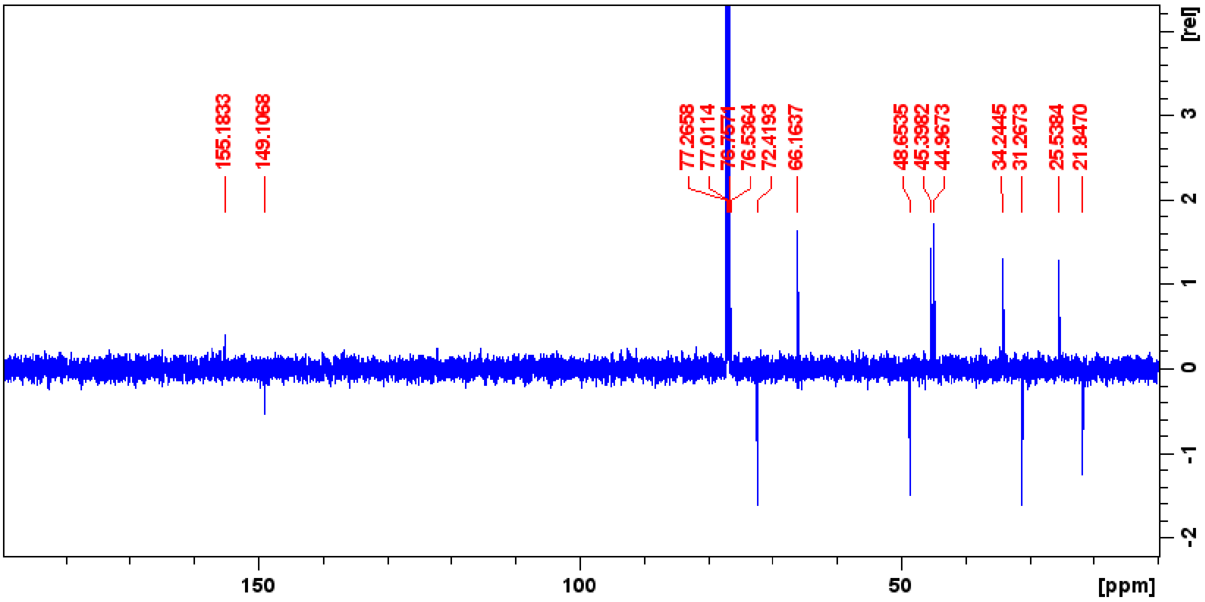

S42: ^{1}H- and ^{13}C-NMR of compound (1*R*,2*S*,5*R*)-2-((*R*)-1-((5-amino-6-chloropyrimidin-4-yl)amino)-3-hydroxypropan-2-yl)-5-methylcyclohexanol (**23d**)

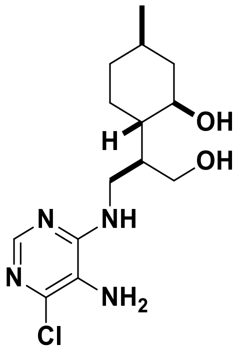

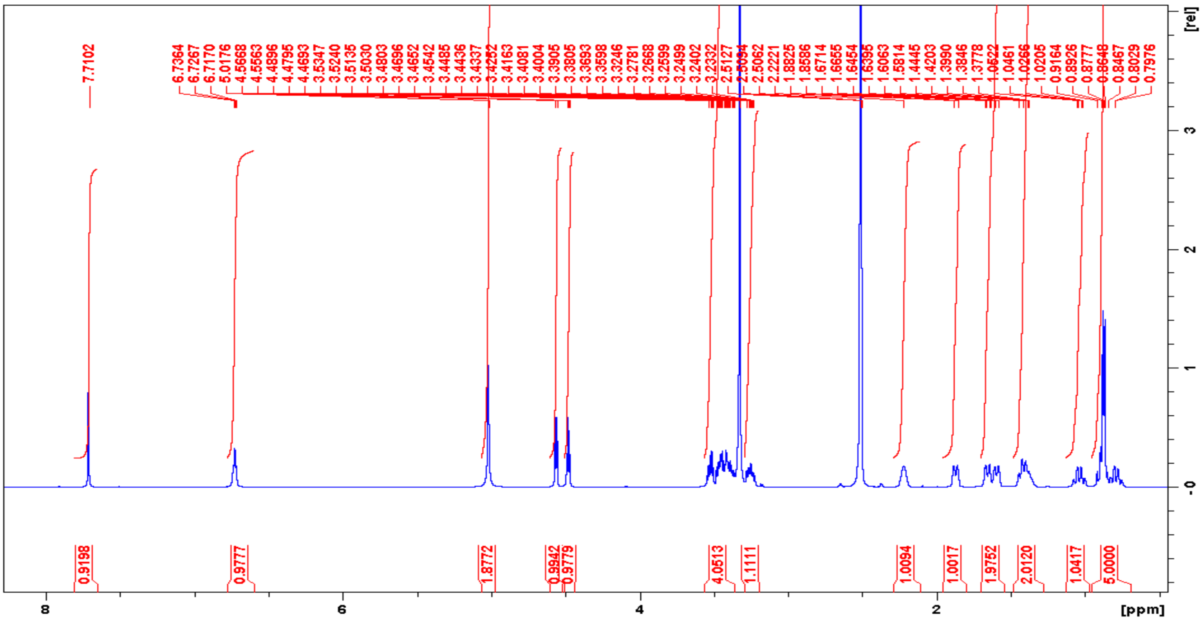

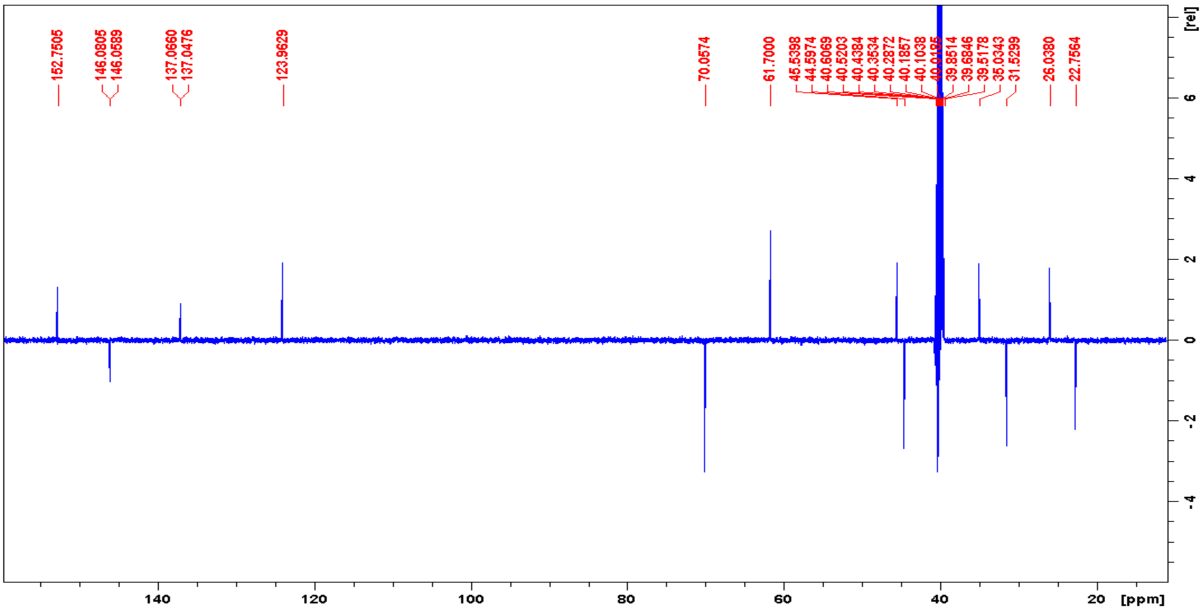

S43: ^{1}H- and ^{13}C-NMR of compound (3*S*,3a*R*,6*R*,7a*R*)-3-(((5-amino-6-chloropyrimidin-4-yl)amino)methyl)-6-methyloctahydrobenzofuran-3-ol (**23e**)

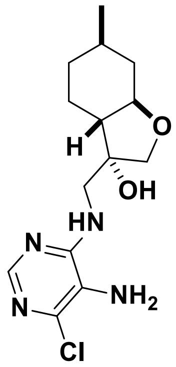

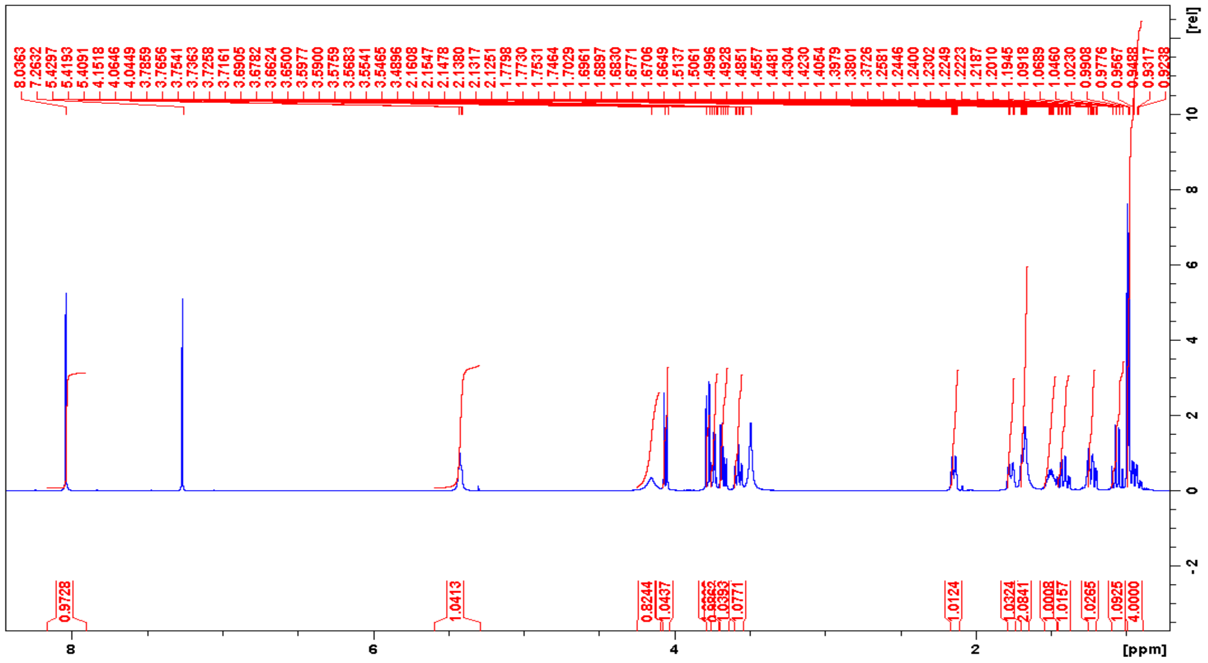

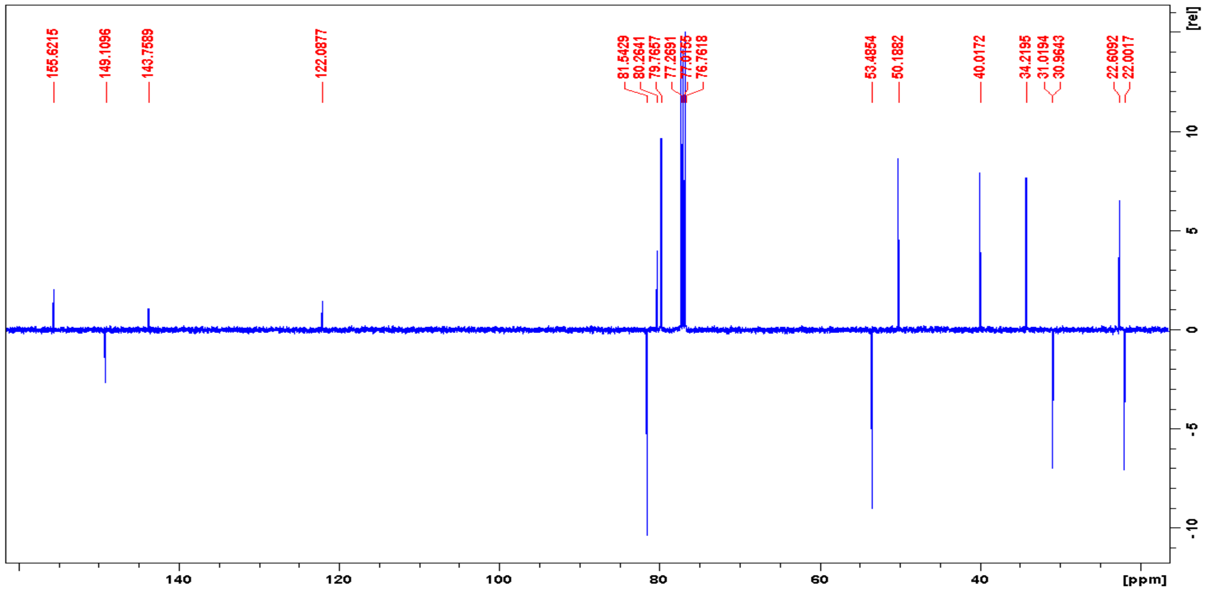

S44: ^{1}H- and ^{13}C-NMR of compound (1*S*,2*R*,5*R*)-2-((*S*)-1-((5-Amino-6-chloropyrimidin-4-yl)amino)-2-hydroxypropan-2-yl)-5-methylcyclohexanol (**23f**)

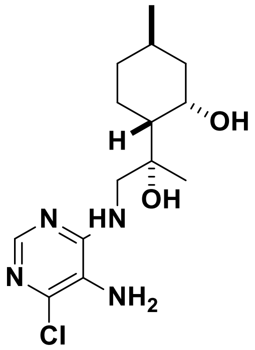

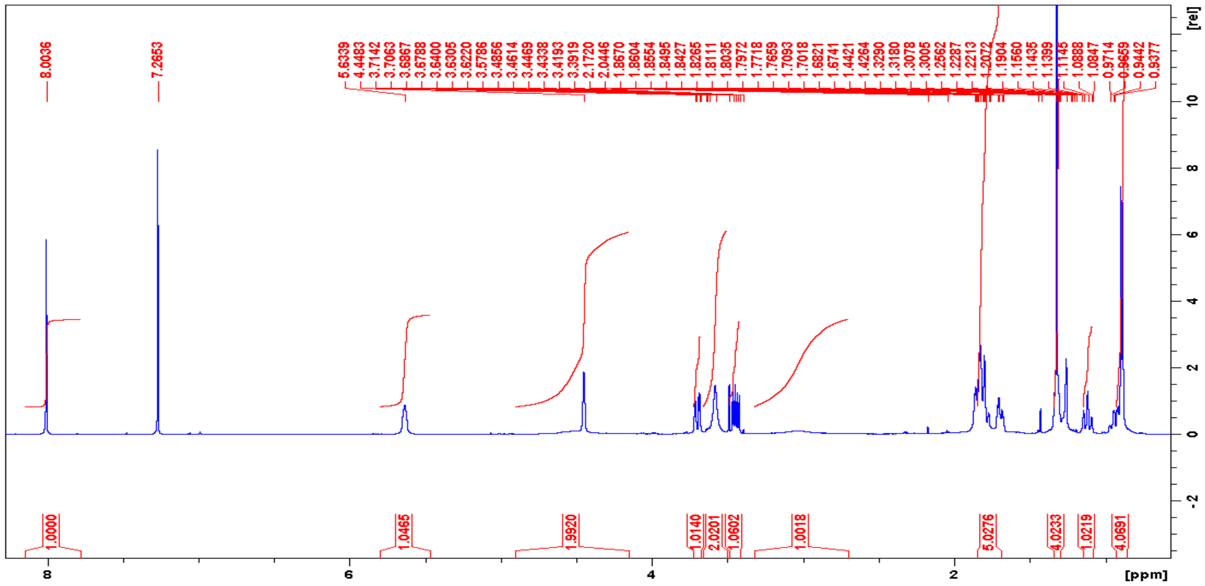

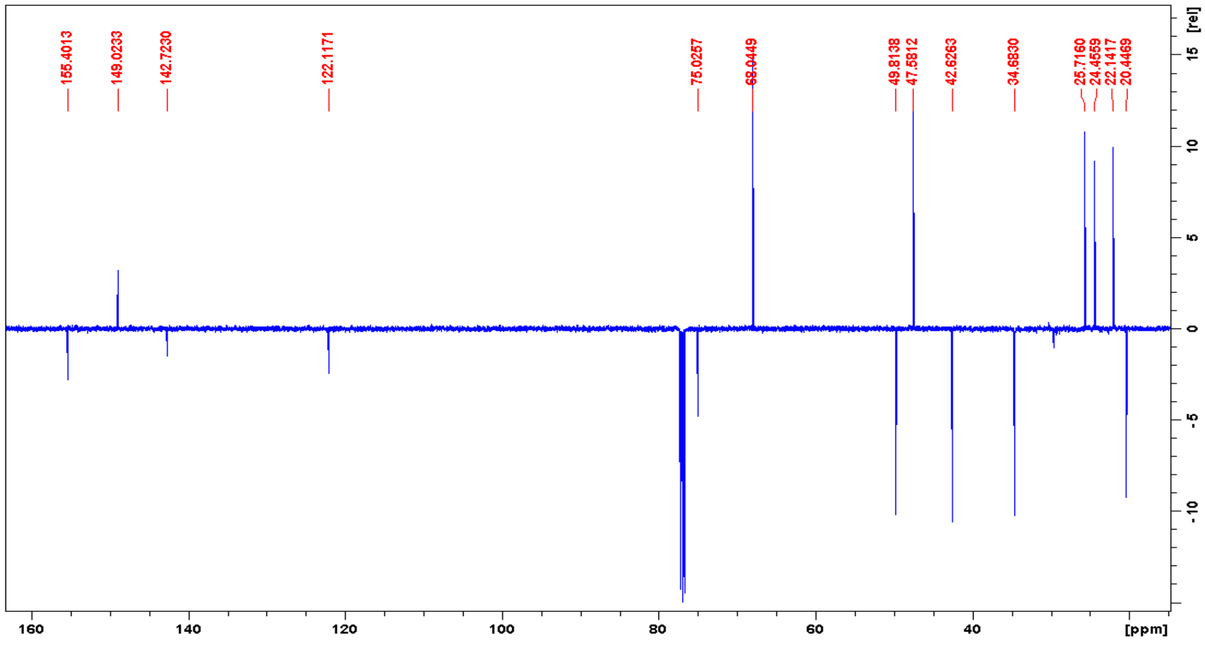

S45: ^{1}H- and ^{13}C-NMR of compound (1*S*,2*S*,5*R*)-2-((*S*)-1-((5-amino-6-chloropyrimidin-4-yl)amino)-3-hydroxypropan-2-yl)-5-methylcyclohexanol (**23g**)

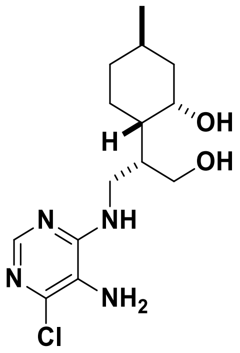

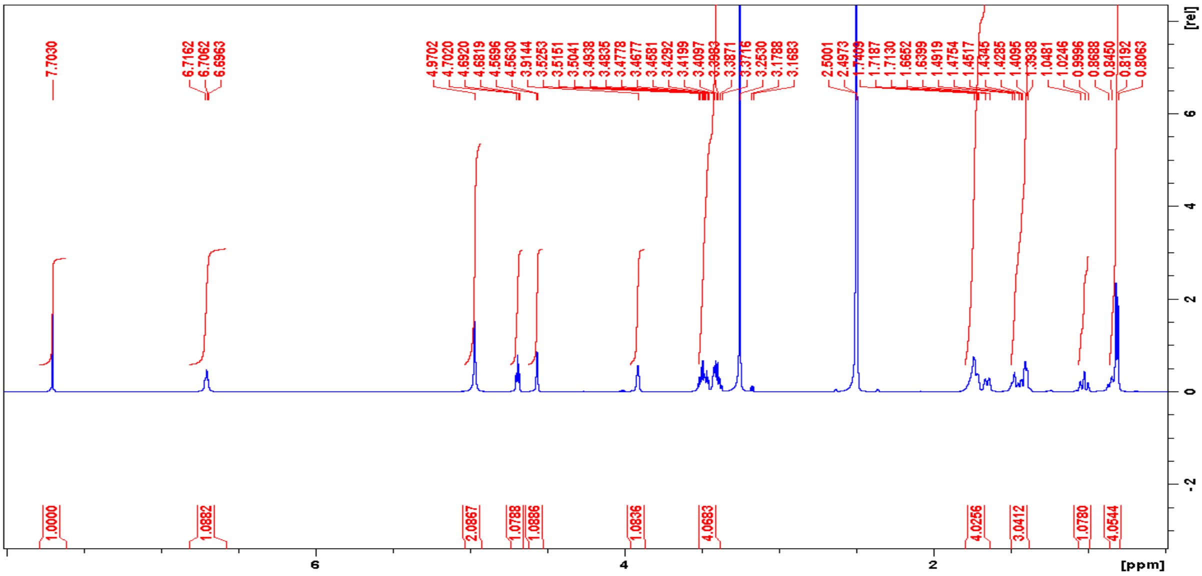

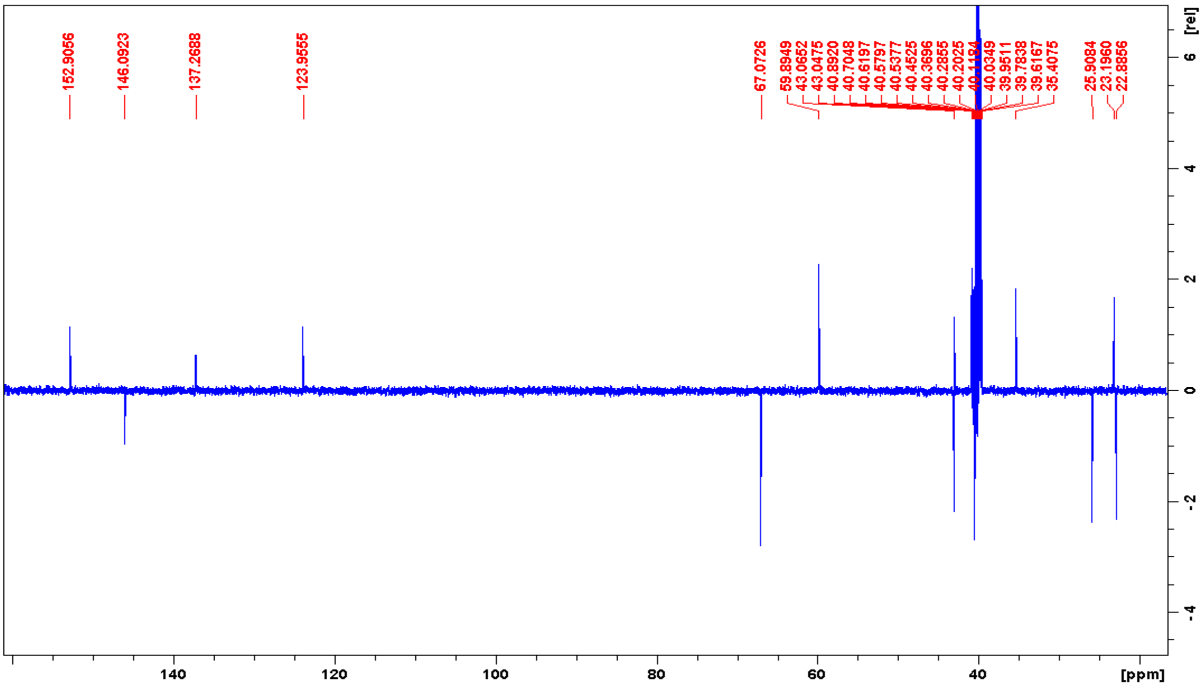

S46: ^{1}H- and ^{13}C-NMR of compound (3*R*,3a*R*,6*R*,7a*S*)-3-(((5-amino-6-chloropyrimidin-4-yl)amino)methyl)-6-methyloctahydrobenzofuran-3-ol (**23h**)

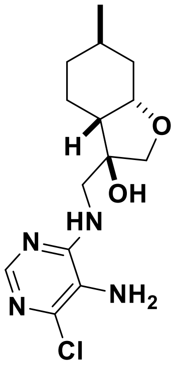

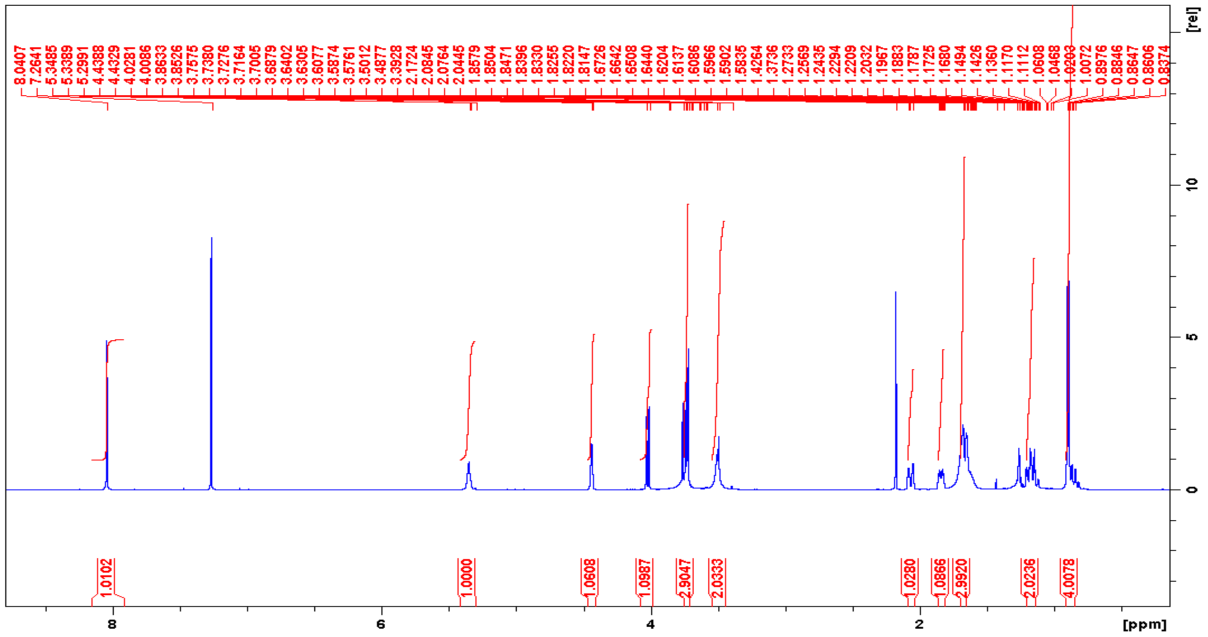

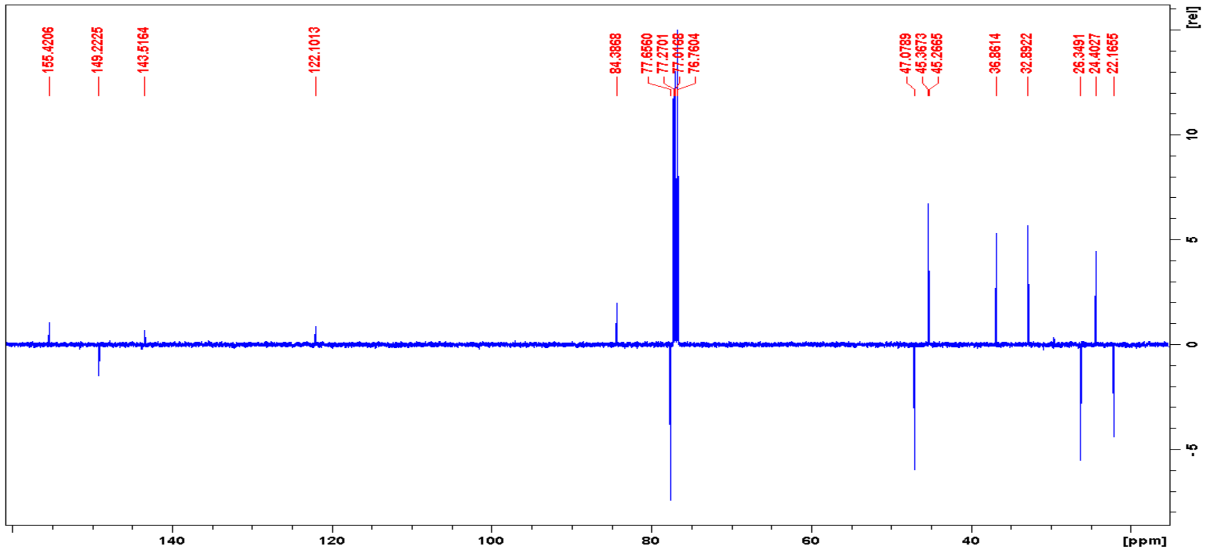

4. Docking study

According to the validation study, the RMSD values showed that the crystal structure 4DEE (ADP bound to Aurora A active site) is valid and could be confidingly used for docking study, where the RMSD values of all ADP generated conformations were less than 2 A° (Table 2).

Table 2. RMSD values of ADP generated conformations

Entry	RMSD (A)
4DEE 1	0.8808
4DEE 2	1.9663
4DEE 3	0.9532
4DEE 4	0.8146
4DEE 5	0.8178
4DEE 6	1.1551
4DEE 7	1.1506
4DEE 8	0.8264
4DEE 9	0.9767
4DEE 10	0.9962

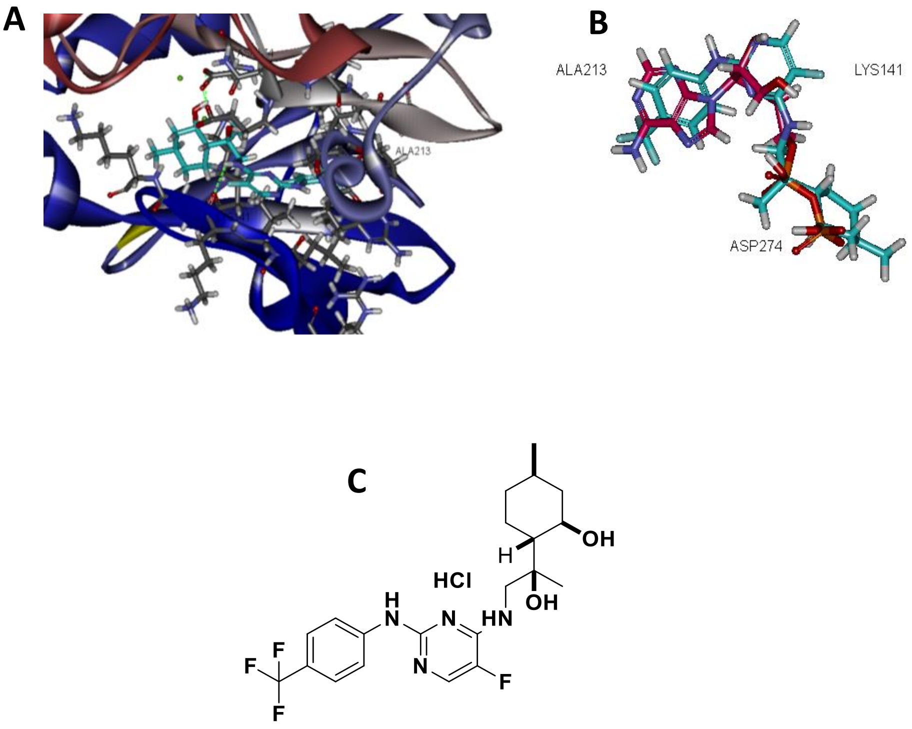

Compound 20b

Figure 1. A: Compound **20b** bound to Aurora A active site (PDB entry 4DEE), B: Overlayof compound **20b** (carbons shown blue) and ADP (carbons shown Red), C: Structure of the compound **20b**.

4.1. Determination of Molecular properties of 20b

Based upon the truth that oral bioavailability is highly affected by the molecular properties of the drug molecule, we have calculated the molecular properties of compound **20b** by using Accelrys Discovery Studio 2.5 software. The Lipinski's rules of five shows that the absorption of a ligand is higher when: molecular weight less than 500 D, calculated log P value less than

5, no more than 5 hydrogen bond donor (HBD) groups, no more than 10 hydrogen bond acceptor (HBA) groups, and no more than 10 rotatable bonds.

As shown in table (3), it is obvious that compound **20b** in accordance with Lipinski's rules of five, so it might have good oral bioavailability.

Table 3. Molecular properties of compound **20b**

Compound	ALogP	Molecular Weight	Nun. HBA	Num. HBD	Num. Rotable bounds
**20b**	3.41	442.45	6	4	7

4.2. In-Silico ADMET analysis

ADMET refers to absorption, distribution, metabolism, excretion and toxicity properties for a molecule. They were predicted for compound **20b** by using ADMET descriptors in Accelrys Discovery studio 2.5. There are six mathematical models are used to quantitatively predict properties of a set of compounds. These models contain: aqueous solubility (predict solubility in water at 25ºC, blood brain barrier (BBB) penetration, cytochrome P450 (CYP450) 2D6 inhibition, hepatotoxicity, human intestinal absorption (HIA) and plasma protein binding.[11] An ADMET model was also generated to predict the human intestinal absorption (HIA) and Blood Brain Barrier (BBB) penetration of tested compound. The model includes 95 and 99% confidence ellipses in the ADMET_PSA_2D and ADMET_ALogP98 plan as shown in figure (2).

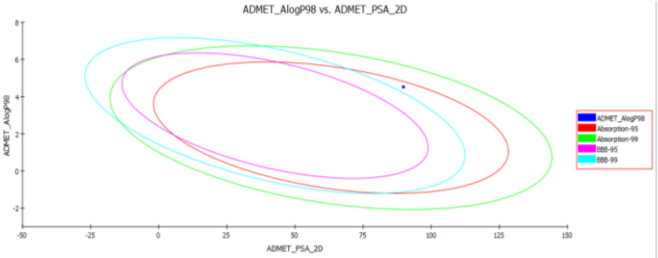

Figure 2. Plot of Polar Surface Area (PSA) vs. LogP for a standard and test set showing the 95% and 99% confidence limit ellipses corresponding to the Blood Brain Barrier and Intestinal Absorption models.

Table 4. In silico ADMET properties of **20b**

Compond	In silico ADMET properties							
	BBB level	Absorption level	Solubility level	Hepato-toxicity	CYP2D6 inhibition	PPB level	AlogP98	PSA 2D
20b	3 (low)	0 (good)	1 (possible)	0 (non-toxic)	0 (non-inhibitor)	1 (>90%)	4.52	89.77

As shown in Table 4, all these values are within the standard range shown in figure (2), which confirm that this compound has good absorption through human intestinal. Additionally, it exhibits low ability to penetrate blood brain barrier (BBB).

4. References

[1] M. G. Rigamonti, F. G. Gatti, *Beilstein J. Org. Chem.* **2015**, *11*, 2117–2124.

[2] T. M. Le, T. Huynh, G. Endre, A. Szekeres, F. Fülöp, Z. Szakonyi, *RSC Adv.* **2020**, *10*, 38468–38477.

[3] T. M. Le, T. Szilasi, B. Volford, A. Szekeres, F. Fülöp, Z. Szakonyi, *Int. J. Mol. Sci.* **2019**, *20*, 4050.

[4] F. Z. Bamou, T. M. Le, B. Volford, A. Szekeres, Z. Szakonyi, *Molecules* **2019**, *25*, 21.

[5] T. Arunkumar, A. F. Ebby, G. Narendrakumar, *Res. J. Pharm. Technol.* **2017**, *10*, 2497–2500.

[6] Z. A. Khdar, F. Sliman, M. Kousara, *Res. J. Pharm. Technol.* **2019**, *12*, 5413–5423.

[7] T. Mosmann, *J. Immunol. Methods* **1983**, *65*, 55–63.

[8] C. Marth, F. Landoni, S. Mahner, M. McCormack, A. Gonzalez-Martin, N. Colombo, *Ann. Oncol.* **2017**, *28*, iv72–iv83.

[9] J. A. Ledermann, F. A. Raja, C. Fotopoulou, A. Gonzalez-Martin, N. Colombo, C. Sessa, *Ann. Oncol.* **2013**, *24*, vi24–vi32.

[10] A. Gennari, F. André, C. H. Barrios, J. Cortés, E. de Azambuja, A. DeMichele, R. Dent, D. Fenlon, J. Gligorov, S. A. Hurvitz, S.-A. Im, D. Krug, W. G. Kunz, S. Loi, F. Penault-Llorca, J. Ricke, M. Robson, H. S. Rugo, C. Saura, P. Schmid, C. F. Singer, T. Spanic, S. M. Tolaney, N. C. Turner, G. Curigliano, S. Loibl, S. Paluch-Shimon, N. Harbeck, *Ann. Oncol.* **2021**, *32*, 1475–1495.

[11] R. Chelamalla, A. Makula, *Asian J. Res. Chem.* **2017**, *10*, 331.
